# The Minderoo-Monaco Commission on Plastics and Human Health

**DOI:** 10.5334/aogh.4056

**Published:** 2023-03-21

**Authors:** Philip J. Landrigan, Hervé Raps, Maureen Cropper, Caroline Bald, Manuel Brunner, Elvia Maya Canonizado, Dominic Charles, Thomas C. Chiles, Mary J. Donohue, Judith Enck, Patrick Fenichel, Lora E. Fleming, Christine Ferrier-Pages, Richard Fordham, Aleksandra Gozt, Carly Griffin, Mark E. Hahn, Budi Haryanto, Richard Hixson, Hannah Ianelli, Bryan D. James, Pushpam Kumar, Amalia Laborde, Kara Lavender Law, Keith Martin, Jenna Mu, Yannick Mulders, Adetoun Mustapha, Jia Niu, Sabine Pahl, Yongjoon Park, Maria-Luiza Pedrotti, Jordan Avery Pitt, Mathuros Ruchirawat, Bhedita Jaya Seewoo, Margaret Spring, John J. Stegeman, William Suk, Christos Symeonides, Hideshige Takada, Richard C. Thompson, Andrea Vicini, Zhanyun Wang, Ella Whitman, David Wirth, Megan Wolff, Aroub K. Yousuf, Sarah Dunlop

**Affiliations:** 1 Global Observatory on Planetary Health, Boston College, Chestnut Hill, MA, US; 2 Centre Scientifique de Monaco, Medical Biology Department, MC; 3 Economics Department, University of Maryland, College Park, US; 4 Minderoo Foundation, AU; 5 Monterey Bay Aquarium, US; 6 Biology Department, Boston College, US; 7 University of Hawai’i Sea Grant College Program, US; 8 Beyond Plastics, Bennington College, US; 9 Université Côte d’Azur; 10 Centre Hospitalier, Universitaire de Nice, FR; 11 European Centre for Environment and Human Health, University of Exeter Medical School, UK; 12 Centre Scientifique de Monaco, Marine Biology Department, MC; 13 Norwich Medical School, University of East Anglia, UK; 14 Biology Department, Woods Hole Oceanographic Institution, US; 15 Woods Hole Center for Oceans and Human Health, US; 16 Department of Environmental Health, Universitas Indonesia, ID; 17 Research Center for Climate Change, Universitas Indonesia, ID; 18 College of Medicine and Health, University of Exeter, UK; 19 Department of Marine Chemistry and Geochemistry, Woods Hole Oceanographic Institution; 20 Department of Biology, Woods Hole Oceanographic Institution, US; 21 United Nations Environment Programme, KE; 22 Department of Toxicology, School of Medicine, University of the Republic, UY; 23 Sea Education Association, US; 24 Consortium of Universities for Global Health, US; 25 Nigerian Institute of Medical Research, Lagos, Nigeria; 26 Lead City University, NG; 27 Department of Chemistry, Boston College, US; 28 University of Vienna, Austria; 29 University of Plymouth, UK; 30 University of Massachusetts Amherst, US; 31 Laboratoire d’Océanographie de Villefranche sur mer (LOV), Sorbonne Université, FR; 32 Chulabhorn Research Institute (CRI), TH; 33 School of Biological Sciences, The University of Western Australia, AU; 34 Biology Department and Woods Hole Center for Oceans and Human Health, Woods Hole Oceanographic Institution, US; 35 Superfund Research Program, National Institutes of Health, National Institute of Environmental Health Sciences, US; 36 Laboratory of Organic Geochemistry (LOG), Tokyo University of Agriculture and Technology, JP; 37 International Marine Litter Research Unit, University of Plymouth, UK; 38 Theology Department, Boston College, US; 39 Technology and Society Laboratory, WEmpa-Swiss Federal Laboratories for Materials and Technology, CH; 40 Boston College Law School, US

**Keywords:** plastic life cycle, human health, ocean health, microplastics, plastic additives, environmental health

## Abstract

**Background:**

Plastics have conveyed great benefits to humanity and made possible some of the most significant advances of modern civilization in fields as diverse as medicine, electronics, aerospace, construction, food packaging, and sports. It is now clear, however, that plastics are also responsible for significant harms to human health, the economy, and the earth’s environment. These harms occur at every stage of the plastic life cycle, from extraction of the coal, oil, and gas that are its main feedstocks through to ultimate disposal into the environment. The extent of these harms not been systematically assessed, their magnitude not fully quantified, and their economic costs not comprehensively counted.

**Goals:**

The goals of this Minderoo-Monaco Commission on Plastics and Human Health are to comprehensively examine plastics’ impacts across their life cycle on: (1) human health and well-being; (2) the global environment, especially the ocean; (3) the economy; and (4) vulnerable populations—the poor, minorities, and the world’s children. On the basis of this examination, the Commission offers science-based recommendations designed to support development of a Global Plastics Treaty, protect human health, and save lives.

**Report Structure:**

This Commission report contains seven Sections. Following an Introduction, Section 2 presents a narrative review of the processes involved in plastic production, use, and disposal and notes the hazards to human health and the environment associated with each of these stages. Section 3 describes plastics’ impacts on the ocean and notes the potential for plastic in the ocean to enter the marine food web and result in human exposure. Section 4 details plastics’ impacts on human health. Section 5 presents a first-order estimate of plastics’ health-related economic costs. Section 6 examines the intersection between plastic, social inequity, and environmental injustice. Section 7 presents the Commission’s findings and recommendations.

**Plastics:**

Plastics are complex, highly heterogeneous, synthetic chemical materials. Over 98% of plastics are produced from fossil carbon- coal, oil and gas. Plastics are comprised of a carbon-based polymer backbone and thousands of additional chemicals that are incorporated into polymers to convey specific properties such as color, flexibility, stability, water repellence, flame retardation, and ultraviolet resistance. Many of these added chemicals are highly toxic. They include carcinogens, neurotoxicants and endocrine disruptors such as phthalates, bisphenols, per- and poly-fluoroalkyl substances (PFAS), brominated flame retardants, and organophosphate flame retardants. They are integral components of plastic and are responsible for many of plastics’ harms to human health and the environment.

Global plastic production has increased almost exponentially since World War II, and in this time more than 8,300 megatons (Mt) of plastic have been manufactured. Annual production volume has grown from under 2 Mt in 1950 to 460 Mt in 2019, a 230-fold increase, and is on track to triple by 2060. More than half of all plastic ever made has been produced since 2002. Single-use plastics account for 35–40% of current plastic production and represent the most rapidly growing segment of plastic manufacture.

Explosive recent growth in plastics production reflects a deliberate pivot by the integrated multinational fossil-carbon corporations that produce coal, oil and gas and that also manufacture plastics. These corporations are reducing their production of fossil fuels and increasing plastics manufacture. The two principal factors responsible for this pivot are decreasing global demand for carbon-based fuels due to increases in ‘green’ energy, and massive expansion of oil and gas production due to fracking.

Plastic manufacture is energy-intensive and contributes significantly to climate change. At present, plastic production is responsible for an estimated 3.7% of global greenhouse gas emissions, more than the contribution of Brazil. This fraction is projected to increase to 4.5% by 2060 if current trends continue unchecked.

**Plastic Life Cycle:**

The plastic life cycle has three phases: production, use, and disposal. In production, carbon feedstocks—coal, gas, and oil—are transformed through energy-intensive, catalytic processes into a vast array of products. Plastic use occurs in every aspect of modern life and results in widespread human exposure to the chemicals contained in plastic. Single-use plastics constitute the largest portion of current use, followed by synthetic fibers and construction.

Plastic disposal is highly inefficient, with recovery and recycling rates below 10% globally. The result is that an estimated 22 Mt of plastic waste enters the environment each year, much of it single-use plastic and are added to the more than 6 gigatons of plastic waste that have accumulated since 1950. Strategies for disposal of plastic waste include controlled and uncontrolled landfilling, open burning, thermal conversion, and export. Vast quantities of plastic waste are exported each year from high-income to low-income countries, where it accumulates in landfills, pollutes air and water, degrades vital ecosystems, befouls beaches and estuaries, and harms human health—environmental injustice on a global scale. Plastic-laden e-waste is particularly problematic.

**Environmental Findings:**

Plastics and plastic-associated chemicals are responsible for widespread pollution. They contaminate aquatic (marine and freshwater), terrestrial, and atmospheric environments globally. The ocean is the ultimate destination for much plastic, and plastics are found throughout the ocean, including coastal regions, the sea surface, the deep sea, and polar sea ice. Many plastics appear to resist breakdown in the ocean and could persist in the global environment for decades. Macro- and micro-plastic particles have been identified in hundreds of marine species in all major taxa, including species consumed by humans. Trophic transfer of microplastic particles and the chemicals within them has been demonstrated. Although microplastic particles themselves (>10 µm) appear not to undergo biomagnification, hydrophobic plastic-associated chemicals bioaccumulate in marine animals and biomagnify in marine food webs. The amounts and fates of smaller microplastic and nanoplastic particles (MNPs <10 µm) in aquatic environments are poorly understood, but the potential for harm is worrying given their mobility in biological systems. Adverse environmental impacts of plastic pollution occur at multiple levels from molecular and biochemical to population and ecosystem. MNP contamination of seafood results in direct, though not well quantified, human exposure to plastics and plastic-associated chemicals. Marine plastic pollution endangers the ocean ecosystems upon which all humanity depends for food, oxygen, livelihood, and well-being.

**Human Health Findings:**

Coal miners, oil workers and gas field workers who extract fossil carbon feedstocks for plastic production suffer increased mortality from traumatic injury, coal workers’ pneumoconiosis, silicosis, cardiovascular disease, chronic obstructive pulmonary disease, and lung cancer. Plastic production workers are at increased risk of leukemia, lymphoma, hepatic angiosarcoma, brain cancer, breast cancer, mesothelioma, neurotoxic injury, and decreased fertility. Workers producing plastic textiles die of bladder cancer, lung cancer, mesothelioma, and interstitial lung disease at increased rates. Plastic recycling workers have increased rates of cardiovascular disease, toxic metal poisoning, neuropathy, and lung cancer. Residents of “fenceline” communities adjacent to plastic production and waste disposal sites experience increased risks of premature birth, low birth weight, asthma, childhood leukemia, cardiovascular disease, chronic obstructive pulmonary disease, and lung cancer.

During use and also in disposal, plastics release toxic chemicals including additives and residual monomers into the environment and into people. National biomonitoring surveys in the USA document population-wide exposures to these chemicals. Plastic additives disrupt endocrine function and increase risk for premature births, neurodevelopmental disorders, male reproductive birth defects, infertility, obesity, cardiovascular disease, renal disease, and cancers. Chemical-laden MNPs formed through the environmental degradation of plastic waste can enter living organisms, including humans. Emerging, albeit still incomplete evidence indicates that MNPs may cause toxicity due to their physical and toxicological effects as well as by acting as vectors that transport toxic chemicals and bacterial pathogens into tissues and cells.

Infants in the womb and young children are two populations at particularly high risk of plastic-related health effects. Because of the exquisite sensitivity of early development to hazardous chemicals and children’s unique patterns of exposure, plastic-associated exposures are linked to increased risks of prematurity, stillbirth, low birth weight, birth defects of the reproductive organs, neurodevelopmental impairment, impaired lung growth, and childhood cancer. Early-life exposures to plastic-associated chemicals also increase the risk of multiple non-communicable diseases later in life.

**Economic Findings:**

Plastic’s harms to human health result in significant economic costs. We estimate that in 2015 the health-related costs of plastic production exceeded $250 billion (2015 Int$) globally, and that in the USA alone the health costs of disease and disability caused by the plastic-associated chemicals PBDE, BPA and DEHP exceeded $920 billion (2015 Int$). Plastic production results in greenhouse gas (GHG) emissions equivalent to 1.96 gigatons of carbon dioxide (CO_2_e) annually. Using the US Environmental Protection Agency’s (EPA) social cost of carbon metric, we estimate the annual costs of these GHG emissions to be $341 billion (2015 Int$).

These costs, large as they are, almost certainly underestimate the full economic losses resulting from plastics’ negative impacts on human health and the global environment. All of plastics’ economic costs—and also its social costs—are externalized by the petrochemical and plastic manufacturing industry and are borne by citizens, taxpayers, and governments in countries around the world without compensation.

**Social Justice Findings:**

The adverse effects of plastics and plastic pollution on human health, the economy and the environment are not evenly distributed. They disproportionately affect poor, disempowered, and marginalized populations such as workers, racial and ethnic minorities, “fenceline” communities, Indigenous groups, women, and children, all of whom had little to do with creating the current plastics crisis and lack the political influence or the resources to address it. Plastics’ harmful impacts across its life cycle are most keenly felt in the Global South, in small island states, and in disenfranchised areas in the Global North. Social and environmental justice (SEJ) principles require reversal of these inequitable burdens to ensure that no group bears a disproportionate share of plastics’ negative impacts and that those who benefit economically from plastic bear their fair share of its currently externalized costs.

**Conclusions:**

It is now clear that current patterns of plastic production, use, and disposal are not sustainable and are responsible for significant harms to human health, the environment, and the economy as well as for deep societal injustices.

The main driver of these worsening harms is an almost exponential and still accelerating increase in global plastic production. Plastics’ harms are further magnified by low rates of recovery and recycling and by the long persistence of plastic waste in the environment.

The thousands of chemicals in plastics—monomers, additives, processing agents, and non-intentionally added substances—include amongst their number known human carcinogens, endocrine disruptors, neurotoxicants, and persistent organic pollutants. These chemicals are responsible for many of plastics’ known harms to human and planetary health. The chemicals leach out of plastics, enter the environment, cause pollution, and result in human exposure and disease. All efforts to reduce plastics’ hazards must address the hazards of plastic-associated chemicals.

**Recommendations:**

To protect human and planetary health, especially the health of vulnerable and at-risk populations, and put the world on track to end plastic pollution by 2040, this Commission supports urgent adoption by the world’s nations of a strong and comprehensive Global Plastics Treaty in accord with the mandate set forth in the March 2022 resolution of the United Nations Environment Assembly (UNEA).

International measures such as a Global Plastics Treaty are needed to curb plastic production and pollution, because the harms to human health and the environment caused by plastics, plastic-associated chemicals and plastic waste transcend national boundaries, are planetary in their scale, and have disproportionate impacts on the health and well-being of people in the world’s poorest nations. Effective implementation of the Global Plastics Treaty will require that international action be coordinated and complemented by interventions at the national, regional, and local levels.

This Commission urges that a cap on global plastic production with targets, timetables, and national contributions be a central provision of the Global Plastics Treaty. We recommend inclusion of the following additional provisions:

- The Treaty needs to extend beyond microplastics and marine litter to include all of the many thousands of chemicals incorporated into plastics.
- The Treaty needs to include a provision banning or severely restricting manufacture and use of unnecessary, avoidable, and problematic plastic items, especially single-use items such as manufactured plastic microbeads.
- The Treaty needs to include requirements on extended producer responsibility (EPR) that make fossil carbon producers, plastic producers, and the manufacturers of plastic products legally and financially responsible for the safety and end-of-life management of all the materials they produce and sell.
- The Treaty needs to mandate reductions in the chemical complexity of plastic products; health-protective standards for plastics and plastic additives; a requirement for use of sustainable non-toxic materials; full disclosure of all components; and traceability of components. International cooperation will be essential to implementing and enforcing these standards.
- The Treaty needs to include SEJ remedies at each stage of the plastic life cycle designed to fill gaps in community knowledge and advance both distributional and procedural equity.

This Commission encourages inclusion in the Global Plastic Treaty of a provision calling for exploration of listing at least some plastic polymers as persistent organic pollutants (POPs) under the Stockholm Convention.

This Commission encourages a strong interface between the Global Plastics Treaty and the Basel and London Conventions to enhance management of hazardous plastic waste and slow current massive exports of plastic waste into the world’s least-developed countries.

This Commission recommends the creation of a Permanent Science Policy Advisory Body to guide the Treaty’s implementation. The main priorities of this Body would be to guide Member States and other stakeholders in evaluating which solutions are most effective in reducing plastic consumption, enhancing plastic waste recovery and recycling, and curbing the generation of plastic waste. This Body could also assess trade-offs among these solutions and evaluate safer alternatives to current plastics. It could monitor the transnational export of plastic waste. It could coordinate robust oceanic-, land-, and air-based MNP monitoring programs.

This Commission recommends urgent investment by national governments in research into solutions to the global plastic crisis. This research will need to determine which solutions are most effective and cost-effective in the context of particular countries and assess the risks and benefits of proposed solutions. Oceanographic and environmental research is needed to better measure concentrations and impacts of plastics <10 µm and understand their distribution and fate in the global environment. Biomedical research is needed to elucidate the human health impacts of plastics, especially MNPs.

**Summary:**

This Commission finds that plastics are both a boon to humanity and a stealth threat to human and planetary health. Plastics convey enormous benefits, but current linear patterns of plastic production, use, and disposal that pay little attention to sustainable design or safe materials and a near absence of recovery, reuse, and recycling are responsible for grave harms to health, widespread environmental damage, great economic costs, and deep societal injustices. These harms are rapidly worsening.

While there remain gaps in knowledge about plastics’ harms and uncertainties about their full magnitude, the evidence available today demonstrates unequivocally that these impacts are great and that they will increase in severity in the absence of urgent and effective intervention at global scale. Manufacture and use of essential plastics may continue. However, reckless increases in plastic production, and especially increases in the manufacture of an ever-increasing array of unnecessary single-use plastic products, need to be curbed.

Global intervention against the plastic crisis is needed now because the costs of failure to act will be immense.

## Section 1—Introduction

Plastic is the signature material of our age. It has contributed to improvements in human health, extensions in longevity, and growth of the global economy. It has supported some of the most significant advances of modern civilization in fields as diverse as construction, electronics, aerospace, and medicine. Medical breakthroughs that could never have occurred in the absence of plastic include intravenous tubing, oropharyngeal airways, flexible endoscopes, and artificial heart valves. Plastics are used in food packaging and in the manufacture of furniture, toys, clothing, and athletic goods.

It is now clear, though, that the benefits provided by plastics have come at great cost to human health, the environment, and the economy. These harms have been suspected for decades [1,2], and their great magnitude is now becoming increasingly apparent. The main driver of these harms has been massive increases in plastic production from under 2 megatons (Mt) per year in 1950 to more than 400 Mt today [3,4,5,6]. Half of all plastic ever produced has been made since 2002. Sharp increases in plastic production coupled with very low rates of recovery and recycling—less than 10% globally—have led to the accumulation of over 6 gigatons (Gt) of plastic waste in the earth’s environment and wide-scale human exposure to plastics’ and plastic-associated chemicals [7,8]. Plastic manufacture significantly contributes to climate change and is at present responsible for about 3.7% of greenhouse gas (GHG) emissions [9,10,11,12,13], a contribution that is projected to increase to 4.5% by 2060 if current trends continue unchecked [14]. The volume of plastic and plastic-associated chemicals in the earth’s environment has become so great as to exceed global capacity for assessment, monitoring, and response and may be approaching a point where it could irreversibly damage the planet’s support systems [15,16].

The ocean has been badly damaged by plastics [17,18,19,20,21,22,23]. An estimated 4.8–12.7 Mt of plastic waste entered the marine environment from the land in 2010 alone [24]. Macroplastics (the bottles, barrels, packaging materials, and fishing gear that litter beaches, kill marine animals, and accumulate in vast mid-ocean gyres) are the most visible component of ocean plastic pollution. An additional quantity of plastic debris in the ocean consists of chemical-laden microplastic and nanoplastic particles (MNPs) and fibers, mostly formed through the degradation of plastic waste. Many plastics appear able to resist degradation and could persist in the environment for many decades [17].

Plastic is responsible for disease, disability, and premature death at every stage of its life cycle, from extraction of the coal, oil, and gas that are its feedstocks, through transport, manufacture, refining, consumption, recycling, combustion, and disposal into the environment [25,26]. Coal miners and oil field and fracking workers suffer high rates of injury, traumatic death, lung disease, cardiovascular disease, and cancer [27,28]. Plastic production workers suffer high rates of cancer and lung disease. Residents of “fenceline” communities—those adjacent to plastic manufacturing plants—experience high rates of premature birth, low birth weight, childhood leukemia, asthma, chronic obstructive pulmonary disease, cardiovascular disease, vehicular injuries, and mental health problems [29,30,31,32,33]. Plastics’ harms fall disproportionately on the world’s poorest and most vulnerable people. Children are especially susceptible [33].

Research into plastics’ health impacts has been fragmented and has not comprehensively examined these impacts across the plastic life cycle [27,28,29,30,31,32]. Until recently, most studies of plastics’ effects have appeared in agency publications [18,22] or in oceanographic and environmental journals, where they are not often seen by physicians or public health professionals. Plastics’ contributions to the global burden of disease have not been quantified. The health-related economic costs of plastic production, use, and disposal have not been counted.

### The Minderoo-Monaco Commission on Plastics and Human Health

The enormous and inadequately charted scale of plastics’ effects on human health and the economy, and our recognition that these impacts will worsen inexorably if current trends continue unchecked, led us to form the Minderoo-Monaco Commission on Plastics and Human Health [35]. This interdisciplinary Commission is composed of scientists, clinicians, and policy analysts from around the world. It is coordinated by the Global Observatory on Planetary Health at Boston College.

This Commission is committed to educating physicians, nurses, public health workers, the press, civil society, and the global public about the full magnitude of plastics’ hazards.

An additional goal is to inform the work of policy makers, government leaders, and international organizations as they strive to fulfill the urgent call of the United Nations Environment Assembly (UNEA) to curb plastic pollution and its unsustainable impacts by negotiating a legally binding Global Plastics Treaty [36,37].

The Commission presents a comprehensive analysis of plastics’ impacts on human health across its life cycle. We examine direct health impacts, including those caused by chemicals used in plastics, as well as indirect health effects mediated through plastics’ damage to the terrestrial, freshwater, and marine ecosystems. We estimate plastics’ health-related economic costs and construct a framework to support further expansion of these economic analyses [38]. We consider the ethical and moral implications of the unending production, consumption, and disposal of plastics, which fall disproportionately on the poor and disadvantaged, especially in the Global South [39,40]. We identify knowledge gaps and research needs [41].

The Commission offers science-based solutions to slow the pace of plastic production, reduce the accumulation of plastic waste, and protect human and planetary health. Our strongest recommendation is that pursuant to the 2022 mandate of the UNEA, the world’s nations develop and implement a strong, comprehensive, and legally binding Global Plastics Treaty that ensures urgent action and effective interventions across the entire life cycle of plastics [36,42]. It is essential that this Treaty covers all components of plastics, including its myriad chemicals, and that it includes measures to protect the health and well-being of the vulnerable and at-risk populations most seriously harmed by the current patterns of plastic production, use, and disposal [43].

This Commission recommends that a central provision of the Global Plastics Treaty should be a global cap on plastic production guided by targets and timetables and supported by national commitments. Our additional recommendations speak to the need to eliminate unnecessary uses of plastics, especially single-use plastics [44]; the need for plastic manufacturers to take full financial responsibility for their products across their life cycle—extended producer responsibility (EPR) [45]; the need for reductions in the complexity of plastics; the need for health-protective standards for plastic-associated chemicals; and the possibility of listing plastics as persistent organic pollutants (POPs) under the Stockholm Convention.

While gaps remain in knowledge about plastics’ harms to human health and the global environment, the evidence available today unequivocally shows that these harms are already great and indicates that they will increase in magnitude and worsen in severity in the absence of urgent intervention [34]. The manufacture and use of essential plastics may continue, but increases in plastic production, especially endless increases in the manufacture of an ever-increasing array of unnecessary single-use plastic products, need to be curbed.

Key ingredients for future successes in curbing plastic production, reducing plastic waste, and protecting human and planetary health are an educated and engaged citizenry; champions from medicine, science, public health, and sustainable development; strengthened international agencies; and courageous, visionary, and ethically grounded leaders at every level of government who heed the science and act responsibly to protect human health and preserve the earth, our common home [46].

## Section 2—The Plastic Life Cycle and Its Hazards to Human Health

### Introduction

*Plastic pollution is one of the great environmental challenges of the 21st century, causing wide-ranging damage to ecosystems and human health* [14].

**Figure 2.1:**
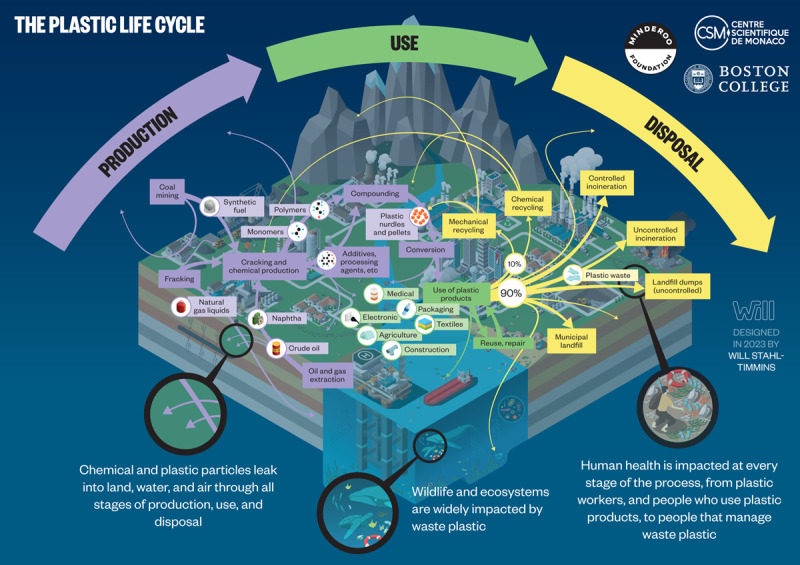
**The plastic life cycle.** The plastic life cycle is long and complex spanning multiple countries. There are three major phases. During production, carbon feedstocks – derived 99% from coal, oil and gas – are transformed through energy-intensive, catalytic processes into a vast array of products. Plastic is used in virtually every aspect of modern life and has provided many benefits. Single-use plastic constitutes the largest market share followed by synthetic fibers, building and construction, transport, electrical, agriculture and medical. Recycling is minimal. Disposal involves landfilling as well as controlled and uncontrolled burning. Plastic-laden e-waste is particularly problematic. Transnational environmental leakage of chemicals and plastic waste occurs throughout the life cycle resulting in extensive pollution and health hazards. *Credit*: Designed in 2022 by Will Stahl-Timmins.

The plastic life cycle is long, complex, and far from circular and encompasses three major phases: production, use, and disposal (Figure 2.1) [47,48]. Plastic production spans multiple countries and continents [12,49], resulting in extensive transboundary pollution and externalization of adverse environmental and human health impacts across national borders [50,51]. Box 2.1 describes our plastic age.

**Box 2.1 Our Plastic Age: From Bright Dawn to Current Reality.**

Plastics are lightweight, versatile, durable, and inexpensive materials that have transformed our daily lives to an extent unimaginable when the first plastics were developed in the 19th century. By the early 20th century, the wide range of potential applications and benefits of plastics were rapidly becoming apparent [53]. The authors of a Pelican reference book of the time offered the following thoughts on plastics’ future:

*It is a world free from moth and rust, and full of color, a world largely built of synthetic materials made from the most universally distributed substances, a world in which nations are more and more independent of localized naturalized resources, a world in which man, like a magician, makes what he wants for almost every need out of what is beneath and around him [**54**]*.

**Figure 2.2:**
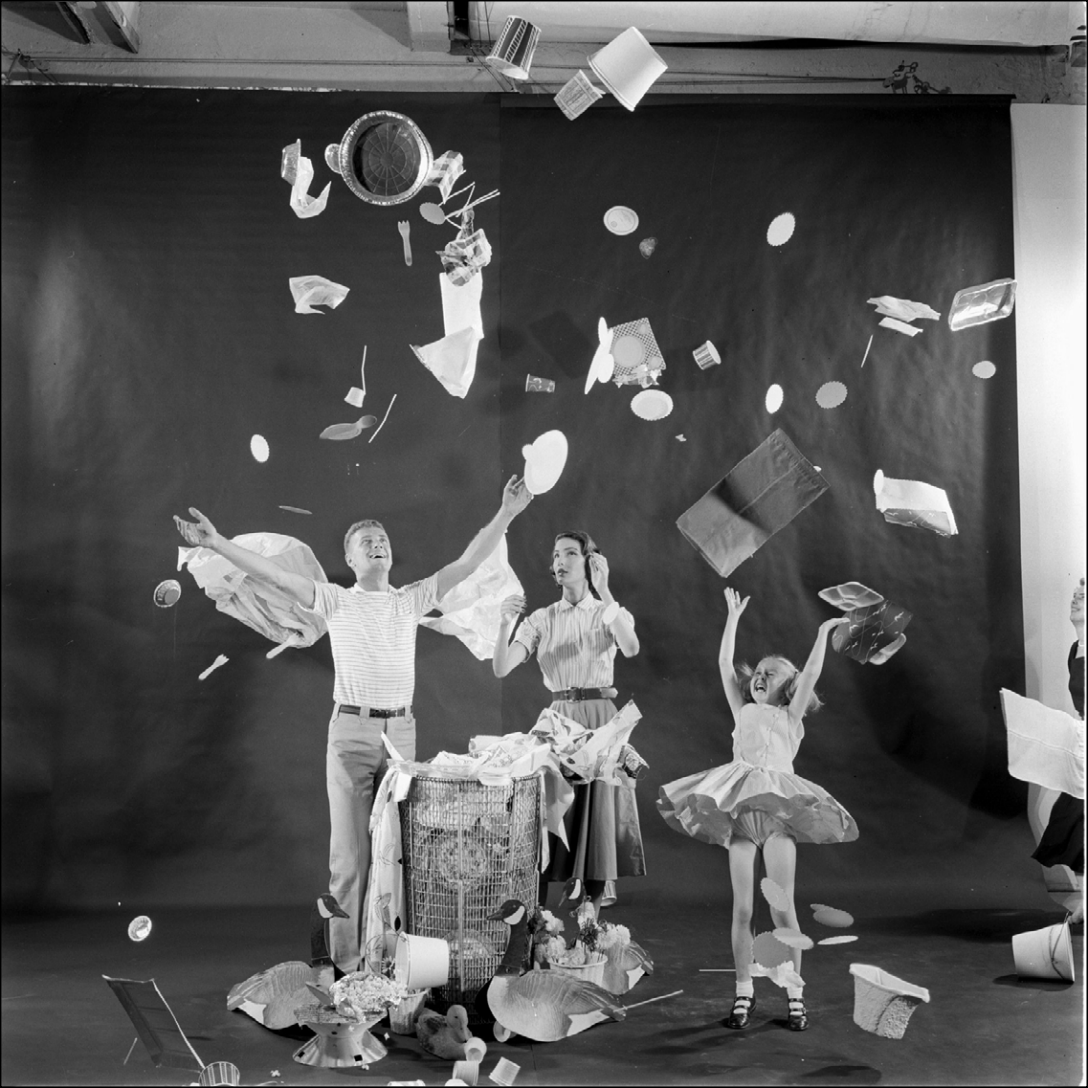
**Throwaway Living – Disposable items cut down household chores.** A family tossing single-use products through the air illustrating how society has turned into a disposable society with throwaway products, New York, NY, July 7 1955 (Life Magazine August 1^st^, 1955). *Credit*: Peter Stackpole/The LIFE Picture Collection/Shutterstock.

By the 1950s, all of the main synthetic plastics in use today, including polyvinyl chloride, polypropylene, polyethylene, polyethylene terephthalate, and polystyrene, had been formulated [55]. Greatly reduced costs were associated with their mass production and led to a shift in plastic use away from predominantly durable goods to the dawn of a new era—disposable living—where for our convenience items made from a range of materials, including plastics, could be used just once, with the environmental costs of this practice being externalized by both producer and consumer. At that time, annual global plastics production was around 5 Mt, and while the concept of throwaway living was inherently wasteful, the quantities of plastic waste produced were still quite small [53]. Indeed, in 1955, *Life* magazine featured a compelling image with an article titled “Throwaway Living: Disposable Items Cut Down Household Chores” (Figure 2.2).

The world’s population has tripled since the 1950s, but production of plastics has increased over 70-fold in the same period. It now exceeds 460 Mt per year, with 35%–40% being the production of single-use items. This increase in production coupled with the popularity of single-use items provides a pathway facilitating the rapid transfer of nonrenewable fossil oil and gas reserves into short-lived products and thence to highly persistent waste that is rapidly accumulating in both managed systems and as litter in the natural environment [2,56]. In short, our plastic age is causing unprecedented damage; it is depleting natural resources, impacting economies, and negatively affecting human health [57,58].

So substantial are the effects of plastic production, use, and disposal that they are considered, alongside climate change and habitat destruction, as substantive negative drivers of the *Anthropocene*—a geological period during which human activity is the dominant influence on climate and the environment [59].

It is clear that our current linear use of a fossil carbon resource, via plastics, to waste is no longer sustainable. If we are to continue to benefit from the opportunities offered by plastics, current practices need to change [57,58]. Levels of concern are so substantive that alongside conventions on biodiversity and climate, the United Nations has recently voted to support a Global Treaty on Plastics, UNEA 5.2.

What then are the solutions? To the present, our understanding of solutions has been largely limited to the waste hierarchy advocated on the first Earth Day in 1970 of the three *R*’s—*reduce, reuse*, and *recycle*—alongside a few other more recent *R*’s, like *redesign* [60] and *refuse* [61].

For plastics, *reduce* appears the most promising of these solutions, especially given the health threats and need for precaution. However, given the many societal and environmental benefits conveyed by plastic [56,62], there will be a limit to the number of plastic products we can reduce by elimination. For example, single-use packaging is often highlighted as the epitome of wastefulness. Yet, that same packaging protects products and extends the life of food and drink, thus reducing food and other wastage, which is another major environmental challenge [56,62].

To retain the benefits offered by our plastic age, without the largely unintended side effects of the linear business model developed in the 1950s, we rapidly need to understand how to use plastics more responsibly. We need granular evidence on which solutions will be most effective in which contexts [60].

- Where will reduction—for example, by restricting production—work, and where might this increase usage of other materials?
- How can we reduce health risks and toxic exposure?
- Where will deposit return be appropriate?
- Which products should be designed for circularity?
- Can recyclability be increased by redesign?
- How do we engage consumers to change?
- What polices and legal frameworks are needed?
- How do outcomes vary between nations?
- What are the costs, and who will carry them?
- What are the consequences of these changes on employment, health, and the environment?

Plastic pollution is a wicked problem, and we are now at a crossroads of opportunity to address it. UNEA 5.2 is an immense achievement [60]. Delivering on its ambition will require robust evidence from transdisciplinary research and stakeholder collaborations to indicate the most, and the least, appropriate interventions. While we need to act with urgency, as we move toward solutions, it is important to recognize that robust scientific evidence and evaluation will be just as critical as it was in raising awareness of the issue itself [60].

Success in the next chapter of our plastic age rests with us all—the public, governments, international organizations, and industry.

In the 75 years since large-scale production began in the aftermath of World War II, more than 8,300 Mt of plastic have been manufactured [3]. Annual production has grown from under 2 Mt in 1950 to 460 Mt in 2019 [4,5]. Plastic production is on track to treble by 2060 [14].

In this Section, we describe the plastic life cycle and the hazards that plastics pose to human health at each phase of this life cycle. We define *health hazards* as factors that increase the risk of an adverse health outcome following exposure [52]. Detailed descriptions of the resulting adverse health outcomes and some of their underlying mechanisms are presented in Section 4.

### Production

#### Production—Markets and market trends

Plastics are high-volume, highly heterogeneous manufactured materials based on chemical polymers. They are versatile, useful, durable, and low cost. More than 8 Gt (8 billion tonnes) of plastics have been produced over the past seven decades. Annual production has grown from 2 Mt in 1950 to 460 Mt in 2019, a 230-fold increase [5].

In the past two decades, global plastic production has increased almost exponentially, with an average annual growth rate since 2012 of 4.6% [63]. More than half of the 8.3 Gt of plastic produced since 1950 have been manufactured since 2002 [64].

**Figure 2.3:**
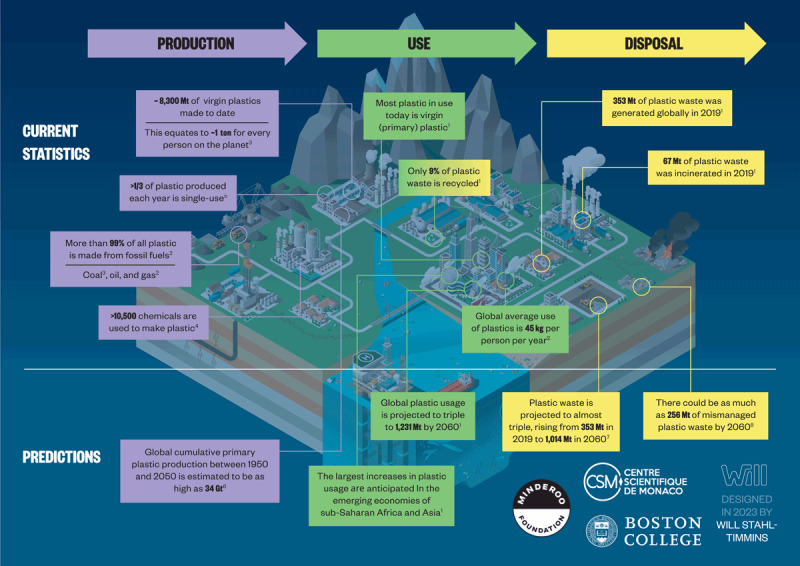
**Plastic life cycle: Production and market predictions.** Large volumes of plastic have been made since production started in 1950s with continuing predicted increases. Mt, Megatons; Gt, Gigatons. **References**: ^[1]^(Organisation for Economic Co-operation and Development (OECD), 2022a); ^[2]^(European Environment Agency (EEA), 2021); ^[3]^(Cabernard *et al.*, 2022); ^[4]^(Wiesinger, Wang and Hellweg, 2021); ^[5]^(Charles, Kimman and Saran, 2021); ^[6]^(Geyer, 2020); ^[7]^(Organisation for Economic Co-operation and Development (OECD), 2022b); ^[8]^(Lebreton and Andrady, 2019). *Credit*: Designed in 2022 by Will Stahl-Timmins.

The Organisation for Economic Co-operation and Development (OECD) predicts that global plastic use will continue to increase from 460 Mt in 2019 to 1,231 Mt in 2060 [14]. Overall production and market trends are summarized in Figure 2.3.

Single-use and short-lived plastics account for 35%–40% of current plastic production [65], and this fraction is growing very rapidly. The production of single-use plastics is predicted to increase by 30% (70 Mt) between 2021 and 2025 [65].

Estimates of plastic consumption vary by region and product type. China and the US account for 20% and 18%, respectively, of current plastic demand [5]. Annual consumption of single-use plastics varies widely, for example, being 50–60 kg per person in Australia and the US; 30–45 kg in South Korea, the UK, Japan, and Saudi Arabia; and 5–30 kg in Germany, Thailand, Turkey, and India [65]. The largest future increases in plastic use are anticipated to occur in the emerging economies of sub-Saharan Africa and Asia [5].

The main driver of recent explosive increases in plastic production and the manufacture of single-use plastics is a massive pivot in investment by multinational fossil carbon corporations. These vertically integrated companies, which produce coal, oil, and gas as well as plastics and petrochemicals, are pivoting away from fossil fuel production in response to growing demand for renewable energy while also expanding their capacity for polymer production [13,65,66]. The companies principally responsible for this pivot include Sinopec (+36% growth in plastic production), ExxonMobil (+35%), and PetroChina (+38%), with even greater expansions being anticipated for Russian-owned SIBUR (+240%), Oman Oil Refineries and Petroleum (+269%), and Indian HPLC-Mittal (+343%) [65]. An additional driver of recent increases in plastic production is growing demand from emerging economies.

Only about 30% of the plastic produced since 1950 is still in use [3]. Most plastics used today are virgin plastics, as opposed to recycled plastics, with polypropylene (PP) (16%), fibers (13%), high-density polyethylene (HDPE) (12%), and low-density polyethylene (LDPE) (12%) predominating [5]. Of the virgin plastics produced globally in 2020, 52% were produced in Asia (32% in China and 3% in Japan), followed by North America (19%), Europe (17%), the Middle East and Africa (7%), and Latin America (4%) [5,12].

Plastic recycling rates are very low, about 9% globally [5]—much lower than recycling rates for glass (European Union [EU] ~75%), paper (EU ~70%), and aluminum (EU ~65%) [67,68,69]. The combination of rapid growth in the production of numerous different polymer types coupled with low recycling rates has resulted in the generation of enormous quantities of plastic waste. Cumulative global production of plastic waste since 1950 is estimated to be 5.8 Gt [3].

The quantity of plastic production can be contextualized by considering that plastic-carbon (83% of plastic mass, on average) equaled total carbon in global human biomass in 1962 (0.06 Pg-C, petagram = 10^15^ g) and, assuming current trends (i.e., cubic growth), is predicted to equal global blue carbon mass (14-Pg-C) in 2035 and total carbon in global bacterial biomass (70-Pg-C) by 2095 [70,71].

The Stockholm Environment Institute has developed the concept of “planetary boundaries” to define a “safe operating space for humanity,” the conditions necessary for human societies to survive and thrive on earth. Plastics and chemicals are now included in this framework along with climate change and biodiversity loss [72]. The Stockholm Environment Institute has recently concluded that production and environmental dissemination of plastics and petrochemicals have increased so rapidly and uncontrollably that they have outstripped global capacity for assessment and monitoring. Risk is high that pollution by plastics and chemicals could—like climate change and biodiversity loss—lead to abrupt, nonlinear, and catastrophic disruption of the earth’s operating systems; destabilize modern societies; and endanger human survival [16].

#### Production—Fossil fuel extraction

##### Conventional

Fossil carbon—coal, oil, and natural gas—is the primary feedstock for more than 98% of global plastic production [12,66,73]. Counting use for both plastic feedstocks and for energy consumed during plastic production, coal contributed by far the largest proportion of this fossil carbon (67% in 2015) compared to oil (23%) and natural gas (10%) [13].

Global estimates of the total amount of fossil fuel used to make plastic are, by contrast, difficult to derive because of flexibility in consumption of feedstocks by the petrochemical industry [74], a lack of detailed data on this aspect of industrial consumption, and regional differences in the mix of fossil fuels used to generate energy versus other products [13,74]. However, it was estimated that, in 2015, 3.8% of the fossil carbon was used globally, both as feedstock and energy, to produce plastic [13]. Similarly, the World Economic Forum estimated that plastics’ share of global oil consumption was 6% in 2014 and is predicted to increase to 20% by 2050 [7,74]. For the US, the Energy Information Administration’s Short-Term Energy Outlook has forecast continuing increases in crude oil production from 12.3 million barrels/day in 2019 to 12.8 million barrels/day in 2024 [75]. Similarly, a report from British Petroleum predicts robust increases in the noncombusted use of oil, gas, and coal driven by “particularly strong growth in plastics, despite increasing regulation on the use of plastics [76].”

Coal production has significant environmental impacts. Virtually all the elements of the periodic table occur in coal, and 20 are present in quantities sufficient to be of environmental concern, namely arsenic, cadmium, chromium, mercury, lead, selenium, boron, fluorine, manganese, molybdenum, nickel, beryllium, copper, thorium, uranium, vanadium, zinc, barium, cobalt, and antimony [77].

On- and offshore oil production generates significant air pollution as a result of emissions from drilling equipment, hydrocarbons escaping from wells, flaring of natural gas, emissions from transport vehicles [78], and intentional use of chemicals [79,80]. Workers face significant occupational risks, including fire and explosions, particularly during offshore drilling operations that are vulnerable to blowouts and involve handling heavy equipment [78]. Contaminants similar to those used in hydraulic fracturing, or fracking, are found in water produced during the oil extraction process and include benzene, toluene, ethylbenzene, and xylene (BTEX) as well as toxic metal(loid)s such as arsenic, cadmium, chromium, and mercury [78]. The oil industry also uses millions of tons of barium sulfate as a densifying additive in drilling fluids to seal the space around the drill bit [81], which can be solubilized by other common chemicals, thereby creating hazardous waste [78]. PFAS are used for a wide range of applications in the mining and petroleum industry, including foaming and antifoaming, oil field mapping, and as wetting agents and surfactants in equipment such as pipelines, seals, conveyor belts, and electrical insulation [82].

In the Niger delta, oil production and gas flaring from over 100 wells has resulted in extensive water pollution, largely as a result of oil spills [83]. Analyses at nine sites revealed widespread toxic metal contamination with substantial exposures of populations at all ages [83]. The drilling rig failure of British Petroleum’s *Deepwater Horizon* in 2010 in the Gulf of Mexico resulted in the deaths of 11 workers and the largest oil spill in US history; 4.9 million barrels of crude oil were released over three months [84]. There were also multiple human health impacts, including large quantities of dispersants and their aerosolization being associated with toxicological effects such as obesogenicity and illness as well as potential increases in harmful algal blooms, mercury exposure, and pathogenic *Vibrio* bacteria. Due to their heavy reliance on natural resources, Gulf communities were vulnerable to high levels of disruption to their livelihoods, including contaminated fish and shellfish, psychological impacts, and institutional distrust [84].

Natural gas production is associated with multiple health hazards. Natural gas, largely methane and ethane, can be extracted using conventional or unconventional well pads. Conventional methods extract gas from *permeable* reservoirs (e.g., sandstone) by moving gas to the surface without the need to pump. In contrast, unconventional methods extract gas from shale reservoirs, which have *low permeability*, through horizontal drilling combined with staged hydraulic fracturing. Compared to unconventional gas extraction, conventional gas wells have resulted in considerably greater releases of methane to the environment per unit production [85]. The risk of fire and explosion is a potential occupational health and safety hazard for both conventional and unconventional gas wells [86].

While the majority of research on health hazards related to natural gas extraction has been focused on unconventional wells, multiple contaminants have also been detected in groundwater sites near conventional gas production regions. For example, a unique suite of contaminants (e.g., BTEX) was detected in a potable drinking water well from a well pad in a conventional gas region [87]. Also, high levels of endocrine-disrupting activities were detected in the samples from this well pad, including antagonist activities for androgen, glucocorticoid, and progesterone receptors and significant agonist activity for estrogen receptors [88].

##### Unconventional

Unconventional oil and gas extraction, termed *fracking*, engineering jargon for hydraulic fracturing, is a technology used to recover large volumes of hydrocarbons (oil and gas) trapped in fine-grained, porous, low-permeable rock formations such as shale [89]. Industrial fracking opens the natural fault lines and fissures in these geologic formations using a large volume of fracturing fluid (also called “slick water”) that is injected into the rock at high pressure along with proppants (gritty material with uniformly sized particles like sand) to hold the fissures open [89].

Fracking is a chemical-intensive procedure [90]. Slick water includes chemical additives such as polyacrylamide and oxidizers (e.g., ammonium, potassium sodium salt of peroxydisulfate) to reduce viscosity, biocides to prevent degradation of polymer-containing fluids, and hydrochloric acid to clean the bores after fracking, as well as an array of other chemicals with numerous functions [89,91]. Although chemical additives make up a small percentage of fracking fluid by volume (0.5%–2%), the massive quantity of fluid required to crack shale leads to the use of vast amounts of chemicals. An average injection of fracking fluid can total approximately 18,500 kg of additives per frack per well [92]. The chemicals used in fracking are not only abundant but also diverse. Over 1,000 chemicals have been identified in fracking fluids and/or wastewater [90], many of which are known or suspected carcinogens [93].

The fracking process generates multiple hazards, including airborne emissions of methane and other pollutants, groundwater contamination, induced seismicity, and the flammable and explosive nature of the extracted oils [89,94]. In the US alone, producers have drilled over 52,000 shale gas wells. Failures of well casings, leakage from aboveground storage, emissions from gas-processing equipment, and the large number of heavy transport vehicles moving through previously isolated rural communities have been endemic, contributing to environmental contamination and human exposure [95].

###### Air pollution

Fracking releases large volumes of air pollutants from over a dozen processes and sources [94]. Air pollutants commonly emitted from fracking include particulate matter (PM) and volatile organic compounds (VOCs) [95], especially methane [96]. Air pollutants can be emitted through direct releases, such as venting and flaring; from “fugitive” leaks; or from other junctures in the fracking infrastructure, such as wastewater pits, dehydrators, and pipelines [97]. Air pollutants can also originate from the volatilization of fracture fluid components and from proppant injection. A 2011 study noted that 37% of chemicals used in US operations could escape to ambient air [93]. An examination of material safety data sheets and chemical databases such as TOXNET indicates that fracking chemicals are associated with a range of adverse health impacts, including respiratory disease; injury to the eyes, skin, sensory organs, and gastrointestinal (GI) tract; cardiovascular disease; and kidney and liver damage. Eighty-one percent of the fracking chemicals used have the potential to damage the brain and nervous system [93].

Air pollutants emitted from fracking operations can travel great distances [94]. For example, an assessment of air quality 1.1 km from a fracking site in western Colorado over the course of a year before as well as during and after its development and operation [98] detected a wide range of chemicals in every sample during the study, including VOCs (methane, ethane, propane, and toluene) and carbonyls (formaldehyde and acetaldehyde). Chemicals with the highest concentrations (in order) across the sampling period were methane, methylene chloride, methanol, ethanol, acetone, and propane [98]. Concentrations were highest during initial drilling and did not increase during fracking [98], but 30 of the detected chemicals affect the endocrine system. Concentrations of fracking-related air pollutants are also highest in proximity to fracking sites, and residents living within 0.8 km of wells are at greater risk for adverse health effects than residents living farther away [99].

Volatile hydrocarbons emitted from fracking operations can be photochemically oxidized in the atmosphere in the presence of nitrogen oxides (NO_x_) to form ground-level ozone. Ozone concentrations in several drilling regions have exceeded the current eight-hour national ambient air quality standard (less than 71 parts per billion) set by the US Environmental Protection Agency (EPA) [100] and have been shown to extend into nearby residential communities [31,94]. Ozone levels above the air quality standard are considered harmful to human health, and both short- and long-term exposures to ozone are associated with adverse health effects, including preterm birth [101] and increased respiratory [102], cause-specific [103], and all-cause [104] mortality.

###### Water pollution

From 2012 to 2014, 116 billion liters of water were used annually in the US alone to extract shale gas compared to 66 billion liters/year for unconventional oil extraction [105]. Once the slick water and proppant are injected and as the fracking process proceeds, a large proportion of this water returns to the surface, as “flowback” water; this mainly consists of hydraulic fracturing fluids and “formation water” (i.e., naturally occurring water from rock formations such as shale), which is associated with high rates of oil and gas production. With time, the fracturing fluids are depleted, and oil and gas production decreases; now the “produced” water is composed almost entirely of formation water [105]. Because they are contaminated with VOCs and other organic compounds, neither flowback nor produced water can be returned to drinking water supplies [106]. A systematic evaluation of 1,021 chemicals identified in fracking fluids and/or wastewater for potential reproductive and developmental toxicity found that data were available for only 24% of these chemicals, 65% of which presented potential adverse reproductive and developmental effects [90]. For example, BTEX, which are VOCs and known hazardous toxins, have been detected in wastewater from shale oil and gas fields in concentrations ranging from 96.7 μg/L to 9 mg/L [107]. Fracking wastewater also contains contaminants such as radionuclides (e.g., radium and radon) and metal(loid)s (e.g., cadmium, copper, zinc, chromium, lead, arsenic), which are drawn from the shale formation and flow to the surface in quantities capable of presenting significant hazards [108,109,110].

In theory, wastewater from the fracking process can be safely contained in tanks, disposal wells, and pits. In actual practice, however, this water and its chemical additives, toxic metals, and radioactive isotopes escape into local surface water and groundwater supplies to cause extensive contamination [106]. Spills and leaks into the environment can occur at every point in the fracking process—from blowouts, storms, and flooding events; unlined wastewater evaporation pits; accidents during transportation of chemicals and wastewater; outflows during mixing, pumping, and at numerous points during active fracking; and mechanical failure, all enabling seepage [110,111]. Toxic pollutants, including benzene and other hydrocarbons, have been detected in surface and groundwater [95] near fracking sites, and higher levels of estrogenic, antiestrogenic, or antiandrogenic activities have been reported in surface water and groundwater from a drilling-dense region of Colorado compared to reference sites [87].

#### Production—Transport of oil and natural gas

Following its extraction, crude oil and natural gas are moved from the wellhead to the refinery and beyond using various means of maritime, pipeline, rail, and road transportation. Factors such as cost, speed, and distance largely determine the mode of transport.

##### Maritime transport

Due to their low cost and relative safety, tankers are among the most viable options for the transport of large volumes of crude oil and natural gas (liquefied petroleum gas, liquefied natural gas (LNG), and compressed natural gas), as well as their products and derivatives [112]. Tankers are used to transport 50%–60% of the world’s crude oil supply and are thus indispensable for the international oil trade [113]. As a proportion of international maritime trade, tankers have increased steadily over the past 40 years [114]. The development of ultra-large crude oil carriers has almost doubled deadweight tonnage from 0.337 billion in 1980 to 0.601 billion in 2020 [115]. Barges are also used to transport smaller quantities of crude oil and natural gas on inland waterways, and they are used together with tugboats for bulk transport to and from remote locations, such as offshore drilling rigs [116].

Maritime transport of crude oil and natural gas is hazardous, with diverse and far-reaching environmental and human health consequences when operations go awry. Between 1997 and 2007, there were 24 major oil spills, with releases ranging from, for example, 37,000 tons (*Exxon Valdez*, 1989) to 287,000 tons (*Atlantic Empress*, 1979) and shoreline impacts from 1 km to 3,000 km [117]. Oil spills threaten the lives of crew members and can also result in economic loss and resource damage, along with serious negative consequences for affected aquatic and terrestrial ecosystems and coastal communities [118,119,120,121,122]. The effects of seven major oil tanker spills on human health between 1989 (*Exxon Valdez*, Alaska) and 2002 (*Prestige*, Spain) have been reviewed. Epidemiological studies have found both acute and chronic health impacts [123]. Acute toxicity was seen among individuals involved in the cleanup and included headaches, sore throats and eyes, and respiratory symptoms, all known toxicological effects of oil. Genotoxicity assessment revealed DNA damage. Long-term adverse neurological effects, including stress and generalized anxiety disorder, were also reported [123]. The fraction of the oil involved in these spills that was destined for plastic manufacture is unknown.

The physical properties of LNG determine its hazards, which include fire and explosion, vapor clouds, rollover, freezing liquid, rapid phase transition, and pool fire [124]. Hazards to the health of individuals on board LNG carriers include gas leaks (which can result in asphyxiation), cryogenic burns, and confined space hazards. LNG spillages can ignite, resulting in pool fires, which can generate damaging levels of thermal radiation that threaten the surrounding environment [125] and release combustion products that are hazardous to human health, including smoke, carbon monoxide, and carbon dioxide (CO_2_), among others [126].

##### Pipelines

Pipelines require significant outlay to construct because they are fitted with multiple valves, pump/compressor stations, communications systems, and meters, but once built, they are one of the most efficient, cost-effective, and safest means of moving crude oil, petroleum products, and natural gas across vast distances [127]. Offshore pipelines are typically more expensive and difficult to build than land-based pipelines, which can be installed either aboveground or belowground [128]. Currently, the most extensive pipeline network is in the US [127]. In 2013, approximately 61,000 and 320,000 miles (that is, ~99,000 and 515,000 km) of the US pipeline network was dedicated to the transport of crude oil and gas, respectively [129].

Pipelines have a lower spill incident and fatality rate per Gt-miles of oil transported compared to other means used to transport crude oil. However, pipeline failures, deteriorating infrastructure, human error, and natural disasters can all result in major pipeline breaches, which can have severe and long-lasting impacts on public health, the environment, and regional economies [130,131]. Gas pipelines are also associated with their own unique hazards. In addition to accidents and unintended leaking (fugitive emissions), intended releases (blowdowns) are used to relieve pressure, with plumes reaching 30–60 meters into the air. Although little is known of their full chemical content, these emissions are known to include methane, ethane, benzene (a known human carcinogen), toluene, xylene, 1,3-butadiene, and other compounds that are either known or suspected endocrine disruptors [132]. Bayesian modeling and simulation suggest causal effects between pipeline emissions and both thyroid cancer and leukemia [133].

Mechanical failure of welded seams, external mechanical damage, and severe internal corrosion of gas transmission pipelines also occur and result in explosions, fires, and the formation of large craters [134]. Sudden ground shifts caused by rapid thawing and freezing, even in mild climates, can induce severe subterranean forces [135]. In Massachusetts, in 2018, a series of explosions and fires resulted from the overpressurization by an order of magnitude of a low-pressure pipeline; causation was attributed to lack of hazard analysis and safety review [136]. In sub-Saharan Africa, gas explosions are largely unreported, but a 2014 review revealed 28 separate pipeline explosions that killed 1,756 people (injuries were seldom reported in Nigeria, Kenya, Ghana, Sierra Leone, and Tanzania), with the most common causes of the explosions being intentional via theft, vandalism, or military activity [137].

##### Rail transport

Rail transport is used to transport crude oil and natural gas to refineries as well as refined products to downstream destinations, particularly when existing pipeline capacities are insufficient or do not link to required locations [138,139,140]. Rail has traditionally been considered a safe and efficient means of transporting oil [139]. However, increased use of railroads for the transportation of crude oil and a spate of highly publicized railroad accidents have revived concerns about the safety of transportation of crude oil by rail and its potential impacts on the environment [138,141]. Derailment is of particular concern, as it may result in oil spills that release a large amount of environmentally harmful and flammable materials, which in turn may cause fires or explosions, environmental degradation, property damage, injury, or loss of life, as well as potential long-term health and psychosocial impacts [142,143,144].

##### Road transport

Crude oil, natural gas, and refined petroleum products are also transported via roads. Although the highly distributed nature of road transport and the many different fossil carbon products that are transported make attribution to plastic production challenging, the proportion of fossil carbon feedstocks used to make plastic is predicted to increase, for example, to 20% of global oil consumption by 2050 [7]. In general, truck drivers are regularly exposed to numerous occupational hazards, including whole body vibrations, noise, climatic factors, ergonomic hazards, and psychosocial hazards, such as stress, fatigue, and poor lifestyle habits (e.g., cigarette smoking, poor diet, lack of exercise) [145]. They are also exposed to PM, nitrous oxide, and carbon monoxide from diesel exhaust fumes. Traffic accidents also pose the risk of injury, permanent disability, or death [145].

India lacks crude oil resources, yet it is the third-largest global consumer of petroleum and the fourth-largest refiner [146]. However, India’s pipeline network is limited (16,226 km), and, as a consequence, the petroleum and chemical supply chain is almost entirely dependent on road transport [146]. Of the 705 road accidents reported between 2014 and 2019, 8% involved chemicals, the remainder being fuel; health hazards were not reported [146]. India also has a large plastics industry, with 30,000 processing units and 2,000 exporters, that is currently worth US$37.8 billion and is projected to increase to US$126 billion in the next four to five years [147]. For this sector, improvements are needed to improve safety and reduce hazards.

#### Production—Chemical and petrochemical industry

Plastics are highly complex materials. Thousands of chemicals are used to manufacture many different polymers and enhance production and processing; thousands more are added to the polymer matrix to provide properties such as color, flexibility, or fire resistance and to enhance production processes [8]. Additional chemicals are inadvertently included (nonintentionally added substances [NIAS]; see Box 2.4) in the manufacture of plastics or are formed and released during its use [148,149], both of which result in the presence of environmental contaminants in the final product [150,151].

The chemical and petrochemical industry is the second-largest global manufacturing industry [152]. Global chemical production has increased 50-fold since 1950, with projections of a tripling between 2010 and 2050 [153]. It is estimated that over 235,000 chemicals with individual Chemical Abstracts Service Registry Numbers (or CAS numbers) have been registered in national or regional industrial chemical inventories, with a further 120,000 registered without revealing their assigned CAS numbers [36].

Plastics are one part of the complex industrial chemical web that uses 927 Mt per year of fossil fuel–based feedstocks (natural gas, natural gas liquids, naphtha, coal, and refinery feedstocks) to produce 820 Mt per year of chemical products [154]. Plastics make up the majority of these products (40%), comprising thermoplastics (222 Mt, 27%) and thermoset plastics, fibers, and elastomers (107 Mt, 13%). The remainder include nitrogen-based fertilizers (274 Mt, 33%); solvents, additives, and explosives (107 Mt, 13%); and other categories (109 Mt, 13%) [154].

Environmental impacts from oil refineries include toxic air and water emissions, releases of chemicals, hazardous waste disposal, and thermal and noise pollution. Hazardous air pollutants include BTEX and n-heptane, while hydrocarbons, sulfur dioxide (SO_2_), and PM contribute to acid rain [78]. In the US, oil refineries are major industrial sources of SO_2_ and VOC emissions [78]. Impacts extend beyond the refineries. For example, in some US states, oil and gas wastewater is used to deice roads and control road dust. This wastewater can contain salt, radium, toxic metals, and organic contaminants, which runoff and likely reach surface water, groundwater, and water treatment plants [155].

##### Fossil fuel feedstocks for plastic manufacture

Oil, gas, and coal are chemically transformed to provide the feedstocks for plastic manufacture. These feedstocks are naphtha, a product of crude oil refinement; natural gas liquids, a product of natural gas [73,156]; and syngas, a mixture of carbon monoxide and hydrogen produced from gasified coal, which is then converted to methanol [157]. The predominant components of these feedstocks are ethane, methane, and propane, saturated hydrocarbons containing single bonds, also called alkanes.

In the next stage of plastic production, large chemical plants termed “crackers” (catalytic cracking plants) are used to convert alkanes into olefins (also called alkenes), unsaturated hydrocarbons containing one pair of carbon atoms linked by a double bond. Cracking involves different technologies, depending on the feedstock. Natural gas liquids and naphtha are thermally cracked, i.e., broken down into small molecules at a high temperature [158]. By contrast, catalytic processes are used to convert coal-derived methanol into olefins [157]. Cracking also produces other petrochemicals used by the plastics industry, including benzene, toluene, and xylene (as solvents and raw materials); butadiene (used to make synthetic rubber); and styrene monomer [159].

An abundance of cheap oil and gas from fracking, coupled with large stocks of underutilized coal, and declining markets for hydrocarbon fuels are driving massive investment in fossil fuel–based plastic production in the US, China, the Middle East, and Europe [73]. The US Energy Information Administration reports current total monthly dry shale gas production to be 80 billion cubic feet per day [160]. Production of Appalachian natural gas liquids, including ethane for plastic production, is projected to grow faster than any other region in the US over the next 30 years [161]. Because construction costs for ethane cracking plants are very high—US$1 billion or more per facility and US$6 billion for one cracker in Pennsylvania [162]—their build-out will lock in plastic manufacture for many decades to come [73].

Cracking facilities produce multiple airborne pollutants. They generate substantial quantities of CO_2_, thus contributing to climate change. They also release methane, carbon monoxide, nitrogen dioxide, sulfur dioxide, VOCs, and particulate pollutants (PM_10_ and PM_2.5_) [159]. Steam cracking produces hydrogen [159], but given that the feedstocks are derived from natural gas, the hydrogen is not “green” but rather is termed “blue.” [163,164]

##### Monomers

Monomers are small molecules that are polymerized (see “Polymerization, Compounding, and Conversion” below) to form polymers. Ethylene and propylene are two of the main monomers used in the production of the polyolefins polyethylene (PE) and PP, which are the highest volume industrial polymers [73]. Many other monomers are used to make other plastics [8,165]. Many monomers are produced in very large quantities. Thus, an estimated 6 Mt of bisphenol A (BPA), a nonolefinic monomer used to make polycarbonate (PC) and epoxy resins, was produced in 2021 alone [166].

Production volumes of monomers are expected to grow, with an anticipated compound annual growth rate of 6% for BPA dominating within the Asia-Pacific region [166]. BPA’s primary use (95%) is as a monomer in the production of polycarbonate plastics and epoxy resins [167]. Ethylene is used to make PE (~32% of global plastic production), but it is also an important starting material for the synthesis of other plastic monomers, such as vinyl chloride, styrene, and ethylene glycol. The second highest volume monomer for industrial plastic production is propylene, which is used to make PP (23%). Other plastic monomers include vinyl chloride, which is used to make polyvinyl chloride (PVC, 16%); styrene, which is used to make polystyrene (PS, 7%); and ethylene glycol and terephthalic acid, which is used to make polyethylene terephthalate (PET, 7%) [73].

A comprehensive classification of the environmental and health hazards of the monomers used in production of the major classes of plastics has been developed [165]. This classification reflects the intrinsic hazardous properties of the monomers but does not consider the extent of human exposure. The classification is based on EU Classification, Labelling and Packaging criteria, and it ranks monomers on a five-point log scale that considers several properties, including carcinogenicity, reproductive toxicity, respiratory effects, specific organ toxicity, ozone depleting, explosive hazard, and oxidizing hazard. On this scale, monomers are categorized variously as *Phase Out* (Level V), *Risk Reduction* (Level IV), and *Acute Toxicity* (Level III). The most hazardous monomers according to this particular classification criteria include acrylamide, ethylene oxide, propylene oxide, 1,3-butadiene, and vinyl chloride [165].

Hazard ranking of 55 plastic polymers with annual production volumes exceeding 10,000 tons found that 16 were Level V (e.g., polyurethane [PUR] foam, plasticized and rigid PVC, and high impact PS) and 15 Level IV (e.g., phenol formaldehyde resin, unsaturated polyester, and PC—with phosgene). Additional polymers with high-production volumes ranked as hazardous include PVC (37 Mt), PUR (9 Mt), and the styrenic polymer acrylonitrile butadiene styrene [165]. The least hazardous included PP, polyvinyl acetate, HDPE, and LDPE [165].

##### Processing aids and additives

Over 10,500 different chemicals are used to make plastic [8]. In addition to monomers (24%), Wiesinger et al. describe two other broad chemical classes used to manufacture plastics—processing aids and additives [8]. Processing aids (39%) include antistatic, blowing, foaming, cross-linking, and curing agents as well as catalysts, heat stabilizers, antifoaming agents, lubricants, and solvents. Additives constitute the majority of the 10,500 (55%) chemicals incorporated into plastics, and they impart a wide range of functional properties, including durability (antioxidants, light stabilizers, heat stabilizers, biocides, and flame retardants), strength (glass fibers and carbon fibers), and flexibility (plasticizers). It should be noted that antioxidants protect oxidative degradation of plastic [168] and can be hazardous, while other antioxidants are used as food preservatives and are not considered as hazardous [169]. Additives also include colorants (pigments and soluble azo colorants) and fillers (mica, talc, kaolin, clay, calcium carbonate, and barium sulfate), among numerous other functions (e.g., impact modifiers, odor agents, antistatic agents, lubricants, slip agents) [8,170]. It should be noted that the same additive can have more than one function and that approximately 30% of the ~10,500 chemicals added to plastics are uncategorizable due to lack of information [8].

Based on EU, US, and Nordic data, approximately 4,000 of these ~10,500 chemicals are considered to be high-production volume chemicals (that is, annual production exceeds 1,000 tons). Determining how much of this production can be attributed to plastics is difficult because these substances are used in other applications and many production volumes are considered proprietary information [8]. The amounts of additives in plastic products vary widely, with plasticizers being the most predominant (10%–70% wt/wt, especially in PVC), followed by flame retardants (0.7%–25% wt/wt), antioxidants (0.05%–3% wt/wt), and ultraviolet light (UV) stabilizers (0.05%–3% wt/wt) (Table 1 in Hahladakis et al., 2018 [170]). On a weight basis, fillers represent greater than 50% of all additives used, followed by plasticizers, reinforcing agents, flame retardants, and coloring agents [171].

**Figure 2.4:**
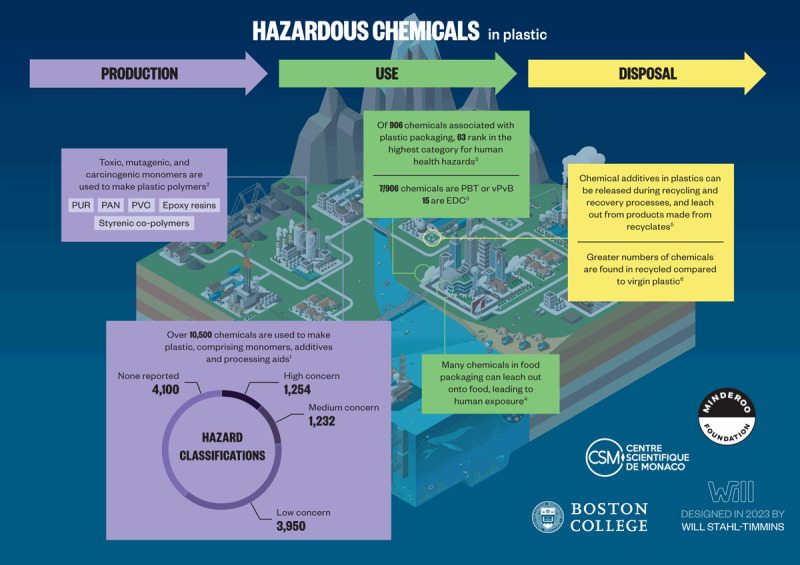
**A multitude of hazardous chemicals are used, present, and released across all stages of the plastic life cycle.** PUR, polyurethane; PAN, polyacrylonitrile; PVC, polyvinyl chloride; PBT, persistent, bioaccumulative and toxic; vPvB, very persistent/very bioaccumulative; EDCs, endocrine disrupting chemicals. **References**: ^[1]^(Wiesinger, Wang and Hellweg, 2021); ^[2]^(Lithner, Larsson and Dave, 2011); ^[3]^(Groh *et al.*, 2019); ^[4]^(Food Packaging Forum Foundation, 2022); ^[5]^(Hahladakis *et al.*, 2018); ^[6]^(Lowe *et al.*, 2021). *Credit*: Designed in 2022 by Will Stahl-Timmins.

Rating systems are used to assess the hazards associated with additives and other chemicals used to make plastic. Commonly used criteria in these ratings include persistence, bioaccumulation potential, and toxicity (PBT); carcinogenic, mutagenic, and reproductive toxicity; endocrine disruption; chronic aquatic toxicity; and specific target organ toxicity upon repeated exposure [132,172,173,174]. Assessments under EU Registration, Evaluation, Authorization, and Restriction of Chemicals (REACH) authorization lists [175] also feature candidate lists for substances of very high concern [176], PBT chemicals [177], and endocrine disruptors [178]. Reported hazard classifications in the regulatory databases are available for ~6,400 (61%) of the ~10,500 known plastic-associated chemicals, while the other 4,100 substances (39%) lack classifications in the databases accessed and are therefore considered as having unknown levels of concern [8]. Of the 6,400 substances with hazard classifications, 3,950 (37%) are of a low level of concern, and 2,486 (24%) meet one or more of the hazard criteria under EU REACH and are identified as substances of potential concern. For those with potential concern, 1,254 (12%) are also high-production volume chemicals and are therefore classified as having a high level of concern, and 1,232 (12%) have a medium level of concern. An overview of hazardous chemicals in plastics is given in Figure 2.4.

##### Selected plastic additives

Given the large number and diverse array of plastic additives and other chemicals used to make plastics [8,165,170], we include summary information in the following subsection on human health hazards for two classes of plastic additives (flame retardants and plasticizers) that were selected on the basis that they are commonly used; are included in human biomonitoring programs, such as the National Health and Examination Survey (NHANES); and among all of the many additives are the best studied. We also included three types of stabilizers (heat, antioxidant, and UV) on the basis that this is an emerging field of research.

###### Flame retardants

Flame retardants comprise a range of chemical groups added to plastics to prevent or slow ignition and combustion. Organohalide flame retardants incorporate chlorine, bromine, or fluorine [179]. BFRs are the most widely used of the halogenated flame retardants [180,181], but other groups, such as polychlorinated biphenyls (PCBs), have also been widely used [182], although they were also used in heat exchangers in electrical capacitors and transformers [183]. BFRs have been in use since the 1970s [181,184] in industrial materials, electronics, and textiles and are also used in substantial quantities in the manufacture of plastic cabinets for televisions, personal computers, and small appliances as well as in PUR cushions for chairs and sofas [184].

Polybrominated biphenyls (PBBs) were the first generation of flame retardants. Their production was discontinued in 1976 because of their toxicity to humans and animals [185]. PBB replacements (“regrettable substitutions,” see Section 4) include polybrominated diphenyl ethers (PBDEs). Because these compounds are PBT, they are mostly restricted under the Stockholm Convention and have been restricted in the US since 2004 [186]. Other halogenated flame retardants, including decabromodiphenyl ethane, hexabromocyclododecane (HBCDD), tetrabromobisphenol A, PCBs, short-chain chlorinated paraffins, the PBB hexabromobiphenyl, PBDEs (including tetra-, penta-, hexa-, hepta-, and decabromodiphenyl ethers and HBCDD are each internationally recognized and regulated as POPs under the Stockholm Convention. Medium-chain chlorinated paraffins are proposed for listing [187].

Organophosphates are another class of flame retardants and have been increasingly used in recent years [188]. Organophosphate esters (OPEs) were introduced in response to concerns about the environmental and health toxicity of BFRs and chlorinated flame retardants [188]. Five main groups of OPEs are commonly used as flame retardants: organophosphates, organophosphonates, organophosphinates, organophosphine oxide, and organophosphites [189]. OPEs may also include various degrees of halogenation [190]. Some OPEs are used as plasticizers in floor polishes, coatings, engineering thermoplastics, and epoxy resins [191]. Volatile OPEs (tributylphosphate, triethyl phosphate, tri(2-chlorethyl)phosphate) are easily released from plastic products into the air, soil, dust, sediments, surface water, and biological media [192]. Humans can be exposed to OPEs through inhalation, ingestion, or dermal contact with dust, air, or water that contains OPEs [192]. An assessment of in-home exposure of 21 mother-toddler pairs to OPFRs found levels of three of five OPE metabolites tested in the urine of both members of virtually all pairs (100%, 98%, and 96%) [180]. OPE metabolite levels in the mothers’ urine were significantly correlated with levels in children’s urine, suggesting similar exposure routes or maternal-child transfer (e.g., transplacentally or via breast milk). In children, predictors of hand-mouth exposure (e.g., less frequent hand washing) were associated with elevated OPEs levels, emphasizing the particular vulnerability of children to exposure to environmental contaminants.

###### Plasticizers

Plasticizers are chemical substances added to polymers to aid processing and increase flexibility [193,194]. Plasticizers help to make plastics softer, more pliable, and more durable [195]. Although as many as 30,000 chemicals have been identified or proposed for use as plasticizers [193], only about 100 plasticizers are commercially produced worldwide, with half of these being commercially important [193]. In 2018, the plasticizers market was estimated to be worth around US$14 million and is projected to reach US$16.7 million by 2024 [196]. Ortho-phthalate diesters comprise up to 85% of the total plasticizer market, of which di(2-ethylhexyl) phthalate (DEHP) and diethyl phthalate are the most common examples [197]. As a specific example, ~97% of DEHP’s use is as a plasticizer, with the remainder (~3–5%) being used as a solvent (i.e., nonplasticizer) in personal care products, such as perfumes and cosmetics [198,199]. Approximately 90% of all plasticizers are used to manufacture flexible PVC [193,196], which is used in products such as food packaging, children’s toys, furniture, medical devices, and adhesives [197,200,201,202].

Like many additives, plasticizers are typically not covalently bound to polymers [194]. Consequently, they can be released from polymer products through a number of pathways, including volatilization to air, leaching into liquids, abrasion of polymer products, and direct diffusion from the polymer to dust on the polymer surface, and contaminate the environment, where they may pose a risk to human health [195]. Some phthalate plasticizers are hazardous [197] and are carcinogenic [203,204] neurodevelopmental toxicants [205] and endocrine disruptors [206,207]. Unlike flame retardants, phthalates are metabolized and excreted and are not biomagnified in the food chain [208]. Diet is therefore not a major route of exposure that occurs instead through other routes such as direct contact with consumer products [200,201,202]. Given their environmental ubiquity and adverse health effects, the use of six major phthalates has been restricted in consumer products, including cosmetics and children’s clothing and toys, and medical devices in several countries in Europe as well as in Japan and the US [209].

Alternative plasticizers to ortho-phthalates have been developed [195] and currently include adipate esters (e.g., di[2-ethylhexyl] adipate [DEHA]); sebacate esters; mono- and dibenzoate esters; cyclohexane dicarboxylic acid esters (e.g., di-isononyl cyclohexane-1,2-dicarboxylate [DINCH]); glycerol esters (including acetylated glycerides); phosphate esters; terephthalate esters (e.g., di[2-ethylhexyl] terephthalate); trimellitates (e.g., trioctyl trimellitate) and biobased alternatives, such as citrate esters (e.g., acetyl tributyl citrate [ATBC]); and epoxidized vegetable oils [195,209]. The extent to which these substances have been implemented varies, with some having been utilized for several decades and others only entering the market more recently [195].

While alternative plasticizers are claimed to be safer than regulated phthalate plasticizers [209], few were tested for safety or toxicity prior to their commercial introduction. Like ortho-phthalates, they have the potential to leach out from plastic products, as they are typically not covalently bound to polymers [197]. These compounds have potential to pollute the environment and threaten human health, and concerns have been raised about regrettable substitution [197,210,211,212]. See Section 4.

###### Stabilizers

Stabilizers are a class of additives that protect plastics from degradation by oxidation, ozone, heat, light (including UV), and bacterial attack [213]. They are incorporated into plastics during processing as well as in final plastic product formulation, and some stabilizers have multiple roles. The EU European Chemicals Agency’s (ECHA) “Mapping Exercise—Plastic Additives Initiative,” completed in 2018, classified substances registered under REACH at above 100 metric tons per annum [214] by function and lists stabilizer chemicals under “light stabilizers” (N = 17), “heat stabilizers” (N = 27), “antioxidants” (N = 26), and “other stabilizers” (N = 22). We follow this categorization here. Biocide stabilizers were not included here because they were not a focus of the ECHA mapping exercise.

For the purpose of hazard assessment, stabilizers are best categorized by chemical class. “Harmonized hazard scores” were estimated for six groups of stabilizer chemicals [213] based on a number of sources, including classification, labeling, and packaging for (1) environmental, (2) human health hazards, or (3) endocrine disruption (based on REACH, United Nations Environment Programme [UNEP], World Health Organization [WHO]); and/or PBT properties; and/or very persistent/very bioaccumulative properties (based on EU classification) [213]. Each of the six stabilizer groups listed (tin, organophosphite, hindered phenol, benzophenone, benzotriazole, and “other”) had high hazard scores on at least one of the above three criteria [213].

###### UV stabilizers

*Benzophenones* (BzPs) were initially used as preservatives in paints and varnishes and were introduced as sunscreens in the 1950s [215]. BzPs are also used in cosmetics and other personal care products [215,216]. Their ability to prevent UV light from damaging scents and colors allows products such as inks, perfumes, and soaps to be stored in clear packaging (glass or plastic) that would otherwise need to be opaque. BzP UV filters are added to plastics and textiles as UV blockers to prevent photodegradation [216,217].

BzP concentrations of 7,426 ng/g in plastic waste from car recycling plants (vehicle “fluff,” namely scrap consisting of, e.g., plastic parts, textiles, seals, and tire fragments) and of 4,637 ng/g in e-waste (plastic cable granulates and plastic residue) have been reported [216]. Partition experiments show that different BzPs (e.g., BzP-1, BzP-2, BzP-3) leach in varying amounts from these products [216]. BzPs have also been found in environmental samples, such as wastewater [216] and indoor dust [218]. They have also been widely detected in wastewater treatment plants, sludge, fresh water, and sediments, with BzP-3 predominating, likely reflecting its use in both commercial and industrial products [219].

*Benzotriazoles* are another class of high-volume chemicals used as UV stabilizers in plastics as well as in a wide range of other products, such as detergents, dry cleaning equipment, and deicing agents [220]. Although the Japanese government banned the use of UV-320 in 2007, replacement benzotriazoles, such as UV-326, UV-327, and UV-328, are still widely used [221]. Indeed, in 2022, POPs Review Committee concluded that UV-328 fulfilled the screening criteria for inclusion in the Stockholm Convention [222].

Benzotriazoles have been shown to be present in plastic bottle caps, food packaging, and shopping bags [221]. The FCCmigex database identifies at least nine benzotriazoles where migration or extraction has been demonstrated from plastic food contact materials (galaxolide and the phenolic benzotriazoles UV-P, UV-P, UV-234, UV-326, UV-327, UV-328, UV-329, and UV-360, with 1–13 entries each) [223]. Benzotriazoles are highly lipophilic, bioaccumulative, and persistent in the environment and have been detected in human blood, breast milk, and urine [221].

*Other UV stabilizers* include triazine UV absorbers [224]; metal chelates, such as nickel phenolates; and hindered amine free radical scavengers [225]. These are less studied, but the FCCmigex database identifies at least two triazine UV absorbers where migration or extraction has been demonstrated from plastic food contact materials (UV-1164 and UV-1577, with one entry each), as well as at least one hindered amine (CAS number 42774-15-2, a tetramethylpiperidine-based hindered amine) [223]. Eleven different triazine UV stabilizers have recently been detected in human breast milk alongside benzotriazoles and BzPs [226].

###### Antioxidants

Antioxidants in plastics include hindered phenols [227], secondary aromatic amines [228], organophosphite esters [229], nonylphenols [230], and organosulfur compounds (thioethers) [231].

*Hindered phenol antioxidants* include butylated hydroxytoluene (BHT), which is used in a large number of products, including food, cosmetics, pharmaceuticals, and fuels as well as plastics and rubbers [232]. Exposure is primarily via diet and occupationally via inhalation in workers handling BHT. Its metabolite (BHT-acid) has been detected in 98% of samples in the German Environmental Specimen Bank, with median levels being slightly higher in women than men [232].

*Nonylphenols* are used as antioxidants (and plasticizers) in various resins. Concern about the endocrine-disrupting properties of nonylphenols led to increasing concern for human health, particularly if used in food contact materials. In fact, migration of nonylphenols from bottles into the water they contained was shown for HDPE and PVC bottles and caps [230].

###### Heat stabilizers

Heat stabilizers are essential for durable products, particularly PVC, with long service lives, as they prevent chain reactions leading to decomposition when products are heated [233]. Metal compounds that contain, for example, lead, cadmium, or tin are often used as heat stabilizers because they prevent the chain reactions and also prevent product deterioration due to sunlight exposure [233].

*Organotins* are widely used as heat stabilizers as well as color control in PVC, with different types of stabilizers being used for different unplasticized (e.g., pipes, fittings, sheet) or plasticized (e.g., cable covering, flooring wall covering, medical footwear, food packaging) products [233]. Organotin compounds are also widely used as antifouling agents in marine paint [234,235]. They are found extensively in the environment and in humans [236].

#### Polymerization, compounding, and conversion

All plastics are based on polymers—large molecules formed by the joining of monomers. During polymerization, mixtures of different monomers are often used to make different polymers. For example, flexible PUR foam is made from propylene oxide (58 wt%), ethylene oxide (7 wt%) and toluene-diisocyanate (29 wt%) monomers; epoxy resin is made from BPA (45% wt%), epichlorhydrin (37 wt%) and 4,4′-methyleneadianiline (18 wt%). Additional chemicals used in polymerization processes include initiators, catalysts, and solvents. Virgin plastics are typically produced in granular or pellet form [156], also called nurdles [237].

**Figure 2.5:**
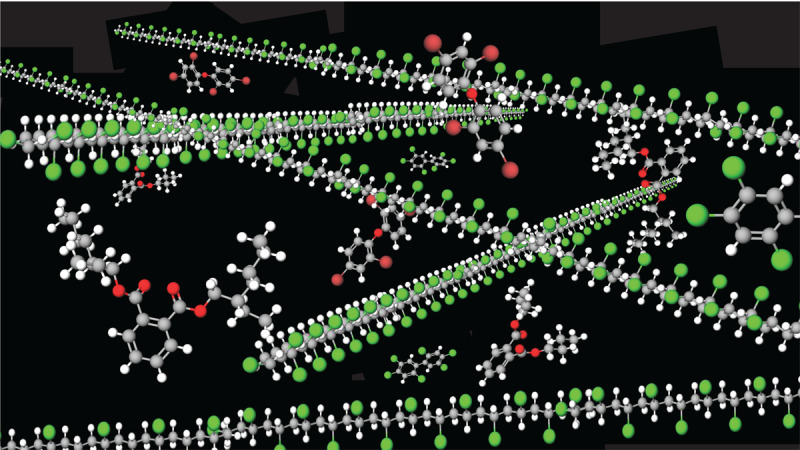
**Plastic is a complex chemical mixture of inter-twined polymers comprising multiple monomeric units joined by carbon-carbon bonds and multiple chemicals added to enhance production and impart properties such as flexibility, strength and durability.** Straight chains are polyvinyl chloride (PVC) polymers. Additives depicted are di(2-ethylhexyl) phthalate (DEHP), di-n-butyl phthalate (DnBP), a polybrominated diphenyl ether congener (PBDE-47) and a polychlorinated biphenyl congener (PCB-153). *Credit*: Manuel Brunner, co-author.

Once formed, polymers are *compounded* with additives and processing agents to produce the properties required for specific plastic products [156]. During compounding, multiple additional chemicals, including additives and processing agents (see above), are inserted into the polymers (Figure 2.5) to instill specific properties, such as color, stability, flexibility, water repellence, and UV resistance. During *conversion*, this mixture is converted into products using various mechanical processes, such as film extrusion, sheet extrusion, extrusion coating, blow molding, foam molding (involving expansion), injection molding, raffia, and PET conversion. Processing also involves heat and pressure to mold plastics into required shapes. Once cooled, products are trimmed, ground, drilled, and sanded and then assembled and painted. Different polymer types are used to make different categories of products. For single-use plastics, approximately 90% are made from five main polymer types, namely PP, HDPE, LDPE, linear-LDPE, and PET [65,238]. The remaining single-use plastics are made of polymers such as PS, PVC, and polyamide (PA) [65].

There are two broad categories of plastic: thermoplastics and thermosets. Thermoplastics can be softened repeatedly by heating and then hardened by cooling, thus allowing reshaping. Thermoplastics include HDPE, LDPE, linear-LDPE, PP, PC, PVC and PS [239,240]. By contrast, thermosets are plastics that change their chemical nature once heated and cannot subsequently be remelted or reshaped. Thermosetting plastics include PUR and a large number of resins, such as epoxies, melamine, acrylic, unsaturated polyesters, and vinyl esters [239].

### Use

#### Nondurable/single-use and durable plastic

Plastic products can be classified into nondurable, which includes single-use plastic, and durable plastics. Nondurable and single-use plastics, both flexible and rigid, have lifetime usages of three to six months and up to three years, but they may be designed to be “disposable” and thus are typically used only once [3,47]. In 2019, the Global Plastic Waste Makers Index estimated that a total of 376 Mt of 14 discrete polymer types were converted into a similar volume of single-use and durable products globally [65].

##### Single-use plastics—Consumer and institutional products

Single-use plastics accounted for 133 Mt, or 35%, of the 376 Mt of plastic produced in 2019 [65]. Of these 133 Mt, 99% (130 Mt) went into packaging. The remainder (3 Mt) was used to make single-use consumer and institutional products [65], such as disposable food service ware, kitchenware, household and institutional refuse bags, and personal care items [241]. Other sources confirm that packaging is by far the major application of single-use plastic in Europe (40%) [12,239]. In the US, an analysis of plastic flows in 2017 showed that 27% of all plastic is used for packaging (27%; Table 2 in Heller et al., 2020 [47]).

##### Durable plastic

Durable plastics accounted for the remaining 65% (243 Mt) of the polymers produced in 2019. These materials were used to make durable products in a number of consumer and industrial sectors [65], including synthetic fibers (66 Mt, 17.5% of total polymers), building and construction (62 Mt, 16.4% of total polymers), transport (16 Mt, 4% of total polymers), electrical and electronics (16 Mt, 4% of total polymers) and “other” (83 Mt, 22% of total polymers) for agriculture, homewares and furniture, and large industrial containers [65].

#### Use—Major sectors

##### Synthetic fibers

A substantial proportion of plastics is used to make synthetic fibers, for example, polyester and nylon, which is used in clothing, furniture upholstery and other household goods, such as carpets and curtains [12,242]. Global production of natural animal and plant fibers has grown slowly over the last 30 years, whereas synthetic fiber production has increased to almost 65 Mt per year [242]. Currently, synthetic fibers constitute almost two-thirds of all textile fibers produced globally, dominated by polyester [12].

Processes used to convert raw polymers to finished fiber-based products, such as dyeing, impregnating, coating, and plasticizing, involve multiple hazardous chemicals [243]. Azo dyes are made of aromatic amines, which are highly carcinogenic as well as genotoxic and allergenic [244]. Aromatic amines were among the first synthetic chemicals to be produced and were documented as early as 1895 to cause cancer of the bladder [245]. Disperse dyes (small polar dye molecules) used to stain synthetic fibers have been reported to be the most common causes of textile allergy [246,247].

Quinoline and its derivatives are extensively used in the manufacture of dyes and have also been detected in synthetic textiles, with some being skin irritants and probable human carcinogens [248]. Benzothiazoles and benzotriazoles are found in azo dyes [220] and have been reported in clothing with printed graphics, including socks and infant bodysuits [249]. As well as being dermal sensitizers, these chemicals are genotoxic and can act as endocrine disruptors [249].

Synthetic textiles with PVC prints, which contain high levels of phthalates, are common, especially in children’s clothing. Phthalate concentrations (as sum of all phthalates) ranging from 1.4 mg/kg to 200,000 mg/kg (20% by sample weight) have been reported in non-peer-reviewed work by Greenpeace [250]. The dominant phthalates found in this investigation were DEHP, butyl benzyl phthalate (BBP), di-isononyl phthalate (DINP), and diheptyl phthalate. BPA and its analog bisphenol S (BPS) have been detected in synthetic textiles, including those marketed for infants, at concentrations of 15–366 ng/g [251]. It is unknown to what extent these chemicals are taken up by infants via, for example, mouthing. We note, however, that the draft European recommendation for tolerable daily phthalate intake is 0.4 ng/kg [252]. Similarly, phthalates in concentrations above EU limits (i.e., 0.1% of mass: European Commission Regulation No. 552/2009) have been reported in a range of infant products, including nylon sheets, cot mattresses, and diaper changing mats [253].

In addition to dyes, a number of metal(loid)s are found in some synthetic fabrics, including high concentrations of chromium, copper, and aluminum in polyester and high concentrations of nickel and iron in nylon. Concentrations of these metal(loid)s, such as chromium, lead, and nickel, have been found to exceed recommended limits in some clothing samples tested [248].

Some synthetic textiles are treated with formaldehyde-releasing compounds and resins to prevent creasing. Formaldehyde, a known carcinogen, has been detected in clothing items at concentrations 40 times higher than specified by international textile regulations [254]. More recently, durable-press fabrics are being produced that release less formaldehyde.

Some synthetic textiles are treated with metal nanoparticles, which act as antimicrobial (silver) or UV absorption (titanium) agents. However, product disclosure is largely lacking, and the forms and concentrations in which these metals are precent are unknown. Nevertheless, dermal exposure is hypothesized to be a likely exposure route [248].

Exposure to airborne microplastics (MPs) during manufacture of synthetic textiles is an occupational risk, although natural fibers such as cotton also carry health risks [255].

##### Building and construction

Building and construction is the third-largest market sector for plastics after packaging and textiles and is the least visible [12]. Industry specifications require durability over decades as well as strength and flame resistance. A wide range of thermoplastics (e.g., PC glazing) and thermosets (e.g., PUR foam insulation) meet these standards and are thus widely used in the building and construction industry. Plastics offer some specific advantages over traditional building materials because they are resistant to heat transfer and moisture diffusion, and do not undergo metallic corrosion or microbial degradation. They can also be molded into different shapes, come in a range of colors and textures, and require minimal or no painting [256]. Products include pipes for supply water and sewage removal; electrical insulation; temperature insulation; waterproof membranes in walls, ceilings, window frames, and window profiles; and flooring [256].

PVC is the most commonly used plastic in the building and construction sector and accounts for almost half (43%) of the plastic used in Europe [12], with construction accounting for 75% of the European PVC market [257]. Global PVC demand was 35 Mt in 2007 and has grown since.

A range of heat stabilizers are used in both rigid and flexible PVC products (670,000 tons globally in 2006), including lead-based, mixed-metal stabilizers (e.g., calcium-zinc, barium-zinc) and tin-based stabilizers [257]. (Stabilizers are discussed in detail in the “Selected plastic additives” section.) The organotin compounds used in PVC as heat stabilizers represent approximately two-thirds of global organotin consumption [258]. A Greenpeace study of PVC flooring revealed concentrations of 330–48,800 µg/kg for monobutyltin, 37,700–569,000 µg/kg for dibutyltin, and 128–17,940 µg/kg for tributyltin [259].

Phthalate plasticizers (580,000 tons globally in 2006) are also a major constituent in certain PVC building materials [257]. PVC is widely used in flooring because it is inexpensive and easy to clean, making it highly practicable in kitchens, bathrooms, and children’s playrooms [259]. Flexible PVC is commonly plasticized with phthalates, historically DEHP and more recently DINP [260]. Analysis of floor samples in 2000 showed DINP concentrations of 4.7%–15.8% mass/mass in all five samples tested and BBP concentrations of 1.6%–5.0% mass/mass in three of five samples [259]. Phthalates are not bound to the polymer matrix and are therefore leachable. Phthalates vaporize from linoleum flooring to air and have been detected in suspended air particles and sedimented dust in homes as well as in water after cleaning plastic floors [259].

Pregnant women living in homes with PVC flooring have significantly higher urinary levels of the BBP metabolite (mono-benzyl phthalate) than pregnant women living in homes made with other flooring materials [261]. Exposure to PVC flooring in early life, including during pregnancy, is related to the incidence of childhood asthma at 10 years of age [262]. As another example of leaching from PVC products, MP shedding occurs inside water pipes and acts as a vector for the release of dibutyl phthalate [263].

Sources of data on alternative building materials include the Pharos and HomeFree databases from the Healthy Building Network [264].

##### Transport, electrical, and electronic

The transport, electronic, and electrical sectors have been revolutionized by the transition from metals to plastic materials reinforced with glass or carbon fibers. Such reinforced plastics (also referred to as “polymer matrix composites”) are associated with an array of functional properties, including high durability, strength, dimensional stability, thermal resistance (>300°C), and the ability to dampen mechanical vibration. They do not require anticorrosion painting. All of these properties result in their being highly versatile and widely used within these sectors [263,265,266].

Reinforced plastics made from both thermoplastic and thermosetting polymers are highly amenable to 3D printing, which enables their use in multiple applications, including electronics, printed circuit boards, aerospace, and biomedicine [266].

Reinforced plastics are approximately one-third lighter than metal alloys, and products from them can be molded, therefore requiring far fewer parts [265]. Because of their light weight and durability, they have significantly improved performance in every mode of road, air, and marine transport [267]. The Boeing 787 Dreamliner is made of 50% plastics, with the remainder being metal alloys, steel, and other materials. Efficiencies are gained not only in production but also during operation. For example, a 600-fleet airline with an average capacity of 200 seats per plane saves 1.9 million liters of fuel and 4.5 million kg of CO_2_ per year due to the use of reinforced plastics in aircraft construction [265]. Hazards to human health during use of reinforced plastic products are as yet unknown although MP shedding from these materials is known to occur [268].

##### Agriculture

Plastics are widely used within the agriculture sector. Macroplastics are commonly used in greenhouses, sheds, covering films and nets, irrigation, implements, twine, netting, storage equipment, and mulch; in addition, pesticides and fertilizers contained in MNPs are used for slow release and targeted delivery [269]. Plastics also enter soils as a result of waste mismanagement and from sewage-derived fertilizer [270]. It has been estimated that plastics constitute 0.1%–0.6% of soil in Swiss floodplain soils [271].

Plastics in agriculture are a source of several chemical pollutants and thus a soil contaminant. Chemicals of concern that leach into soil from plastics include phthalates and residual monomers such as BPA [269]. Metal(loid)s (cadmium, chromium, copper, zinc, nickel, lead, and arsenic) have also been detected in soils and vegetables grown in plastic shed production systems in China [272]. A study in Spain estimated plastic waste to be almost 250,000 kg over 1,500 hectares per year [273]. A recent project using satellite imaging and artificial intelligence revealed that agricultural plastic is a major component of land-based plastic waste [274].

Plastics in soils can alter soil formation, stability, and hydrology and are likely to alter primary nutrient production and cycling [275]. Furthermore, under experimental conditions, MP infill in artificial sports turf has been observed to reduce plant growth, possibly due to the leaching of additives [276]. A recent study using fluorescently labeled PS beads under controlled laboratory conditions revealed their uptake by wheat and lettuce roots, with transfer to the epidermis and xylem [276,277]. Nanoplastic (NP) particles made from PS applied at high concentrations (g/kg or mg/L) have been found to trigger mild reactions in plants resulting in increased root stress. It is not yet known whether environmental MNPs are also taken up into plants from soil under natural conditions, nor whether or to what extent they may bioaccumulate through the food chain.

Soil can also be polluted by chemicals (e.g., metalloids, polycyclic aromatic hydrocarbons (PAHs), VOCs, PCBs, PBDEs, polychlorinated dibenzo-*p*-dioxins (PCDDs), “dioxin-like” compounds) released during the production and disposal of plastics [278,279,280]. Analysis of farmland soils in China has revealed that flame retardants, including OPFRs and BFRs such as PBDEs, are widelydetected [281].

Plastic packaging for animal feedstock has been found to contain several bisphenol compounds, with BPA being the predominant form, and evidence of leaching of these compounds from the products [282]. Microplastics have also been detected in animal feedstock such as fish meal and soybean meal [283].

##### Medical

###### Single-use and durable

Modern medicine has been transformed by plastic. Quantifying plastic use in medicine is challenging, but procurement data and waste audits provide a high-level overview of consumption of products used in medicine such as the following:

- Disposable gloves, intravenous (IV) bags, protective clothing, drapes, blue wrap, nappies, incontinence wear, bed pads, wipes, sharps and various other containers, trays, and packaging;
- Critical medical devices that come into contact with the vascular system and other sterile tissues (e.g., intravenous administration systems, syringes, connectors, tubing); and
- Nonmedical items, including bags (waste, body, specimen, laundry, and patient property), food and drink containers, and cleaning items [284].

A 2021 audit across five hospitals in Europe showed that plastics comprise 70% of sanitary waste and 34% of general waste [284]. Plastic waste has been recognized as a substantial footprint in the health care sector, and various procurement programs that aim to replace products with nonplastic alternatives are underway [285]. See Box 2.2 for more information about the contributions of the health care sector to plastic waste production. The COVID-19 pandemic has resulted in large increases (approximately 350%–370%) in plastic waste. It has been reported that, in one day, care for the inhabitants of Wuhan, China (population ~11 million people) generated 200 tons of medical waste, a volume four times greater than the capacity of dedicatedincinerators [286].

**Box 2.2 Contributions of the Health Care Sector to Plastic Waste Production.**

The origin of the phrase *primum non nocere* (first, do no harm) is uncertain. Although likely to be hundreds rather than thousands of years old [299], it certainly predates invention of the synthetic plastics so widely used in health care today [300].

Global demand for the manufacture of plastic medical disposables, including personal protective equipment and medical devices, more than doubled between 2005 and 2020 [301]. Drivers of this increase include concerns of infection control, which have driven a transition to single-use items, and a culture of defensive medicine that has led clinicians to be overcautious.

Prior to the COVID-19 pandemic, plastic medical waste accounted for 23% of the total waste produced by the UK National Health Service (NHS) [284], with an average of 3.4 kg of single-use materials utilized every day when treating a patient with severe sepsis [302]. Between April 2019 and March 2020, NHS England generated 624,000 tons of waste, 53% of which was incinerated, 29% recycled, 11% alternatively processed, and 7% sent directly to the landfill [303]. The COVID-19 pandemic resulted in an increased demand for single-use products [286,304], demonstrating the vulnerability of health care systems reliant on single-use rather than reusable equipment [305]. Present legislation related to the management of medical waste and lack of clarity on recyclability leads to the majority being incinerated, whether used or not [303,306].

With climate change predicted to cause approximately 250,000 additional deaths per year from malnutrition, malaria, diarrhea, and heat stress, with estimated direct health costs of US$2billion to US$4 billion per year by 2030 [307], the health care profession needs to review its use of “planet-harming technologies and products which fuel its own workload.” Yet, when the concept of “first, do no harm” is applied to a specific patient, clinical tunnel vision can take over. Thoughts of product origin, cost, and planetary or distant community harm are forgotten, as clinicians’ main concerns become protecting the health of their patients, their colleagues, and themselves. Health care systems need to take a broader view and be cognizant of the wider consequences of decisions they make as to which products to use and their potential for reuse.

There have been some limited successes in reducing demand for specific items, e.g., nitrile gloves [308]. Larger reductions in health care’s plastic footprint will, however, require a systems-level approach [285] to reverse years of culture and procurement practices, now made worse by the COVID-19 pandemic.

Fortunately, the profession is becoming increasingly aware that resource extraction and the manufacture, use, and disposal of plastic medical devices cause harm to both people and the planet. This heightened awareness now needs to translate to a full assessment of options, balancing impacts of single-use items with the chemical and energy use needed to decontaminate reusable equipment. Health care systems, including the UK NHS, need to urgently reexamine the products used to provide care, and legislation that hinders progress needs to be reviewed. Up to 50% of health care’s plastic waste arises from packaging [284], which offers the greatest opportunity for change.

Health care services have a vested interest in reducing their impact on the ecosystem; otherwise, they will become increasingly burdened through climate-related acute and chronic ill health [307], which will increase demand for the very products that are harming the environment. In addition, there are presently many unanswered questions about the direct and indirect impacts of the MPs found in human blood, breast milk, and lungs.

While macroplastic health care–associated pollution can be seen and sorted in the health care system, it is essential not to forget sources of equally important MPs. For example, 3.5% (9.5 billion miles a year) of all road travel in England is linked to NHS activity [309], which results in significant amounts of MPs generated through tire and brake pad wear. These MPs contribute to particulate air pollution and are washed into storm drains and thereafter the ocean [310]. Reusable textiles are a source of plastic microfibers produced during washing that are then discharged into hospital wastewater [311,312].

Health care can not only drive change through its considerable buying power but also through the narratives its actions generate. The NHS has 80,000 suppliers, and through communication, it can ensure that these companies understand the need for change. This health care provider-supplier collaborative approach can be replicated globally, help drive positive actions, improve health and well-being, and reduce the impact of providing health care on the planet.

Considerations for health care systems, organizations, and professionals to help reduce plastic waste production include the following:

- Investing in health prevention and keeping people healthy to reduce demand for consumables.
- When health care intervention is planned, ensuring patients are as fit as possible to reduce the incidence of postintervention complications.
- Understanding that decisions and actions are not benign: every piece of single-use equipment used, every patient treated, and every mile traveled has an environmental and human health impact.
- Insisting or mandating that health care goods are supplied in clearly labeled recyclable packaging.
- Reprocessing equipment (e.g., cardiology and laparoscopic) whenever possible.
- Utilizing buying power; rewarding and collaborating with suppliers who are the forefront of reducing the impact of their products, packaging, and delivery miles on the environment.
- Remaining open-minded and considering investments in new technologies for processing medical device waste.

Toxic chemical additives are found in a wide range of plastic-based medical supplies [287], and medical supplies are an important source of human exposure to phthalates, BPA, and PFAS (e.g., see Table 4 in [288]). Phthalates can account for 30%–40% of medical-use plastics by weight [287]. Phthalates are used in medications to control GI drug delivery. Parenteral nutrition enhances leaching from plastic medical products such as blood bags, endotracheal tubes, and cardiopulmonary bypass machines [289]. Leaching of DEHP from PVC-based medical equipment made more than 40 years ago has been described [290]. A 1982 study revealed DEHP metabolites in nonuremic psoriatic patients and in uremic patients undergoing hemodialysis or cardiac bypass surgery [291]. A more recent study on serum and urine samples from 35 adult intensive care unit patients also revealed exposure to DEHP, and to a lesser extent BPA, with patients on hemofiltration, extracorporeal membrane oxygenation, or both showing higher levels (100–1,000 times) than the general population [292].

A 2005–2006 audit of DEHP in medical devices in a large neonatal intensive care unit (NICU) via website searches and phone interviews found that 10 of 21 (48%) devices were DEHP-free and that gaps exist with respect to alternatives to both the PVC polymer and alternative plasticizers [293]. A two-center (97 neonates) study examining PVC medical devices used for infusion therapies, parenteral nutrition, blood transfusion, and respiratory ventilation found urinary DEHP, di-(2-ethylhexyl) terephthalate, and tri-(2-ethylhexyl)trimellitate metabolites in exposed patients [294]. Larger exposures were found for DEHP compared to di-(2-ethylhexyl) terephthalate (5–10 times) and tri-(2-ethylhexyl)trimellitate (57–228 times) [294].

Both phthalates and BPA have been detected in NICU patients, and phthalates are associated with increased risk for cholestasis, necrotizing enterocolitis, and bronchopulmonary dysplasia in newborns [295,296]. In very low birth weight infants, in a single-center study, serendipitous replacement of IV fluids with a DEHP-free formulation over a two-year period resulted in the near elimination of neonatal hypertension, an effect that was reversed when the original brand of DEHP-containing IV fluids was reintroduced [297]. This finding is consistent with broader epidemiological evidence of an association between phthalate exposure and increased blood pressure in children [298].

###### Iatrogenic exposure

An additional emerging route of iatrogenic exposure to plastics is through novel nanoparticle drug delivery systems. These systems promise a number of potential benefits in drug delivery, including transport of medications that may otherwise require complex solvents, targeted delivery or release, and sustained release [313,314,315]. Those synthetic polymer technologies that have made it onto the pharmaceutical marketplace have largely been based on saturated aliphatic polyesters, such as polylactic acid (PLA), polyethylene glycol, or a combination of a saturated aliphatic polyester and polyethylene glycol in block copolymer micelles [314,316], expected to be fully metabolized and excreted [313,314]. There are however a much more diverse range of polymers and nanoparticle technologies in the experimental and developmental phases, beyond the scope of this report, with hydrogel and dendrimer applications being particular areas of focus [313,314,315].

### Disposal

*The current plastics life cycle is far from circular* [5].

Disposal is the third main component of the plastic life cycle, after production and use. With continuing year-to-year increases in global plastic production, especially in the production of single-use plastics, and continuing increases in the accumulation of plastic waste, the issue of waste disposal has become increasingly prominent.

Reduce, reuse, and recycle are the three traditional components of waste management programs. While these strategies have proven highly effective for paper, cardboard, glass, and aluminum, especially when they are coupled with economic incentives such as container deposit fees, they have largely failed for plastic, where recovery and recycling rates are below 10% globally.

The result of continuing near exponential increases in plastic production coupled with very low rates of recycling is that each year an estimated 22 Mt of plastic waste enters the environment. Global generation of plastic waste has more than doubled in the past two decades, from 156 Mt in 2000 to 353 Mt in 2019 [5]. For single-use plastics, 131 Mt was produced in 2019, increasing to 137 Mt in 2021, with predictions of 148 Mt in 2027 [317]. A total of more than 6 Gt of plastic waste have accumulated worldwide since 1950.

Strategies currently used for disposal of plastic waste include controlled and uncontrolled landfilling, open burning, thermal conversion, and export. Of the plastic waste produced globally in 2019, almost half was disposed of in sanitary landfills, 19% was incinerated, 9% was recycled, and 22% was discarded into uncontrolled dumpsites, burned in open pits, or leaked to the environment [5]. In the US, an analysis of plastic flows in 2017 showed that 8% was recycled, 14% was combusted, 76% was landfilled [47].

Vast quantities of plastic waste are exported each year from high-income to low-income countries, where it accumulates in landfills, pollutes air and water, degrades vital ecosystems, befouls beaches and estuaries, and harms human health—environmental injustice on a global scale. Plastic-laden e-waste is particularly problematic.

If current trends in plastic production continue unchecked, it is anticipated under a business-as-usual scenario that the dominant means of plastic waste disposal will continue to be landfilling, and by 2060, the volume of mismanaged plastic waste produced each year could triple to 155–256 Mt each year [14]. A disproportionately high fraction of this future waste is expected to be produced in Africa and Asia [318].

#### Recycling

Recycling is an important component of modern waste management systems, which encourage collection of waste by assigning it an economic value, thereby reducing mismanagement and leakage and displacing primary production. Sectors like glass (EU ~75%), paper (EU ~70%), and aluminum (EU ~65%) packaging have high recovery and recycling rates in many modern economies [67,68,69]. Such efficient waste recovery systems are an important step toward a more circular, less wasteful, and less polluting economy.

Plastic recycling, by contrast, accounts for less than 10% of the new plastic produced globally each year [5]. For single-use plastic, closed-loop recycling (also referred to as “on-par” recycling, for example, bottle-to-bottle recycling) is even lower, being 1% from recycled feedstocks in 2019 and 2% in 2021, with predictions of 3% in 2027 [317].

Current plastic recycling rates by region are US ~5%, Middle East and Africa ~5%, China ~13%, India ~14%, and Europe ~14% [319]. The amount of recycled plastic is increasing somewhat, having quadrupled over the past two decades from 6.8 Mt in 2000 to 29.1 Mt in 2019 [5], but recycling rates are still too low to displace primary production. Thus, virgin plastic production and use continue to soar, and plastic waste continues to accumulate in ever-growing amounts [3,14,320].

Factors that contribute to the global failure to recycle more substantial quantities of plastic are the sheer volume of plastics; unfavorable economics (the high cost of collection, waste transport, and recycling vs. cheap virgin plastic); the wide variety of resins, polymers, multilayer, and composite plastic products; and plastics’ chemical heterogeneity [8,48]. Additional impediments are the extensive use of plastic materials of close-to-zero material value (e.g., small-format packaging such as sachets) and the decrease in plastic quality after recycling (or *downcycling*). A particularly intractable problem is the inclusion in plastic products of multiple toxic chemicals, such as phthalates, BPA, flame retardants, and heavy metals. The presence of these materials in recycled plastic limits its use in consumer products with high potential for human exposure, such as food packaging.

##### Mechanical, physical, and chemical recycling technologies

Several technologies have been developed for recycling plastics and include both mechanical and chemical technologies. Plastic waste is also burned in waste-to-energy facilities, euphemistically termed *thermal recycling* or *pyrolysis*, to produce heat or energy [321].

Plastic recycling processes may have negative environmental impacts, such as the generation of air pollution and toxic ash [322]. Exposures to these waste products are associated with health impacts in recycling workers and in residents of nearby “fenceline” communities [323].

###### Mechanical recycling

Mechanical recycling is a process in which plastic waste is sorted by polymer, shredded, washed, and melted into plastic granulates. This process is suited for thermoplastic materials (e.g., PET, PE, PP, PVC) but generally cannot be applied for thermosets like epoxy resins and most PURs.

Mechanical recycling can be divided into closed-loop or open-loop recycling [324]. Today, closed-loop mechanical recycling is largely limited to PET bottles (bottle-to-bottle) in waste systems where the bottles are collected in a separate waste stream. The majority of mechanical recycling is an open-loop process in which the resulting plastic granulate is of lower quality than the input material. Multiple factors contribute to the lower quality of plastic recycled via this technique. They include polymer degradation during extrusion, cross-contamination with undesired polymers, incomplete removal of odors and colorants, and unknown additive composition—notably the potential inclusion of toxic additives—in the final granulate used for product manufacture. As a result, most mechanically recycled plastics do not meet standards for such uses as food contact materials, which means the plastic is downcycled to products with lower specifications [322]. To overcome the poorer material properties of recycled plastic, mechanical recycling often involves enrichment with additives or virgin polymer.

Depending on the plastic type, melting during the extrusion process can emit toxic chemicals into the workplace air, including VOCs (e.g., vinyl chloride, styrene, formaldehyde, benzene) and PAHs [325,326]. Melting plastic pellets recycled from waste plastic releases additives such as phthalates and VOCs in considerably higher quantities than are released from melting virgin plastic pellets [327]. These toxic chemicals are also being released during the granulation step of mechanical recycling [328]. Workers employed in mechanical recycling operations can be directly exposed to carcinogenic metal(loid)s such as arsenic, cadmium, and chromium present in recycled plastic pellets via skin contact and through inhalation of contaminated airborne dust [329].

Mechanical recycling is described in engineering jargon as a mature technology with a technology readiness level of 8–9 (TRL 1–9: 1 = observation of a new phenomenon (science); 9 = proven application in real-life scenarios). However, mechanical recycling can be improved with advanced sorting technologies, i.e., cleaner feedstock; better pretreatment methods, like a hot washing step; and postprocessing, such as deodorization [330].

###### Physical recycling (purification)

Physical recycling (often referred to as *purification*) is a process in which sorted plastic is treated with solvents to selectively dissolve one or multiple types of polymers [322]. Solvent treatment is followed by a series of steps intended to remove contaminants and additives and by selective precipitation of target polymers. The product is a purified polymer precipitate that can be extruded and compounded to produce close-to-virgin plastic. Physical recycling has gained interest in recent years because it has the potential to address complex plastic waste that is almost impossible to recycle mechanically.

Because it conserves plastic’s material properties, physical recycling is regarded as a closed-loop process. The physical recycling technology is, however, not mature at this time (TRL 4–7) and is currently only operating at small commercial scale (e.g., CreaSolv process in Indonesia).

###### Chemical recycling

Chemical recycling covers a wide range of processes [5]. The common element in all of them is that the chemical structure of polymers is changed. There are two major types of chemical recycling: depolymerization and conversion.

*Depolymerization* technology involves cleaving plastic polymers into their initial monomeric units, which can then be used in a polymerization process to yield virgin-like polymers. Depolymerization can be performed chemically (chemolysis/solvolysis), biologically (enzymolysis), or thermally (thermolysis). It is best suited for PET plastics (most common), PS, and PA plastics, but less so for polyolefins [322].

Depolymerization is regarded as a closed-loop process because additives and contaminants can be removed, and the resulting polymers are indistinguishable from their virgin counterparts. These polymers can then be used to make the same or different higher-value materials (higher than those resulting from mechanical recycling). This is called *upcycling*. Consequently, depolymerization is increasingly gaining traction in the circular waste economy, with multiple pilot and demonstration plants emerging (TRL 3–7) [322]. Its effectiveness at scale is unproven.

*Conversion* is a set of processes that involves breaking down plastic waste into small molecules that can then be used as chemical feedstock in petrochemical facilities. The resulting material can contain organic contaminants, toxic chemical additives, and NIAS [331]. Conversion processes include gasification (TRL 5–8), pyrolysis (TRL 3–9), and hydrothermal conversion (TRL 4–7), based on temperature, catalyst, and reaction medium:

- Gasification generally uses the highest temperatures, which leads to low molecular weight hydrocarbons and syngas as outputs.
- Pyrolysis and hydrothermal conversion are regarded as milder methods that can lead to a mix of low and higher molecular weight outputs, such as gasoline, naphtha, and BTEX.

Theoretically, all polymer types can be converted, and chemical recycling has the potential to remove contaminants, additives, and toxins from the plastic waste stream and under certain circumstances uses low-quality plastic (e.g., from downcycling) and mixed-plastic waste as feedstock.

In practice, however, conversion technologies, too, have a number of disadvantages:

- A certain degree of preprocessing and sorting of waste to ensure good process efficiencies and robust output is required;
- The products of conversion typically have to be fed into postprocessing operations such as separation, catalytic conversion, or steam cracking to yield useful chemical products or fuels;
- Only a fraction of the output from conversion technologies can directly be used to create new plastics (causing the circularity of conversion technologies for plastic recycling to come under debate [322]); and
- Polymers with heteroatoms (e.g., PET) have lower yields, and PVC can cause great challenges for process engineering (hydrochloric acid formation leads to reactor corrosion).

Occupational health hazards to workers in chemical recycling include exposures to toxic solvents (e.g., chloroform, xylene, *n*-hexane, cyclohexane), to airborne emissions from pyrolysis and gasification (e.g., styrene, hydrogen), and to hazardous waste (e.g., chars, tars) [323,332]. The pyrolysis process can result in the production and release of toxic chemicals such as VOCs, PAHs, PCBs, and PCDDs [326]. While adequate management of recycling plants and use of personal protective equipment can mitigate exposure to toxic chemicals [323], some pilot pyrolysis plants have not been equipped with all of the abatement systems intended for use in such facilities, resulting in the risk of occupational exposures of workers to noncondensable gases such as VOCs [333].

##### Recycled products

The ultimate goals of recycling are to curb plastic pollution, reduce production of new virgin plastic, and contribute to a circular economy in which recycled plastic is converted into new products. These aspirational goals are evident in such documents as the EU’s *Directive on Single-Use Plastic*, which requires all PET bottles to be made of 25% recycled plastic by 2025 [334].

The use of a large number of chemicals in plastics is, however, a persistent impediment to recycling and to achieving global plastic circularity goals. These hazardous chemicals have been shown to leach out of recycled plastic in larger quantities than from virgin plastic [335,336]. Chemical analysis of products (children’s toys, fabrics, tires, food contact materials, and construction materials) made from recycled plastics has shown higher numbers of flame retardants, fragrances, solvents, biocides, pesticides, and dyes compared to products made from virgin plastics, suggesting greater exposure hazards [331]; 14 of 20 chemicals with high toxicity scores (abundance, detection frequency, and bioactivity) had a higher prevalence in recycled plastics compared to virgin plastics [331].

A further problem is that restricted/banned chemicals such as legacy POPs may be unintentionally reintroduced into the market if outdated plastic products are included in the recycling waste stream [337]. For example, PBDEs and PBBs have been detected in children’s toys made from recycled plastic in several countries [338,339,340].

Another problem is that chemical contaminants may be introduced into recycled plastic from multiple sources during disposal, collection, and processing [151,341,342,343,344]. For example, analysis of food containers and plastic films made from recycled PET revealed metal(loid) contamination (average concentrations: cadmium 8.8 ppm; chromium 6.8 ppm; nickel 9.4 ppm; lead 0.2 ppm; antimony 8.3 ppm) [345]. Likewise, higher concentrations of antinomy and BPA have been detected in recycled compared to virgin plastic [336].

A systematic evidence map of chemical migration from recycled PET food and drink bottles showed that, of 193 chemicals examined, over 150 chemicals migrated from the polymer into food samples [336]. These included chemicals such as antinomy, acetaldehyde, and a number of endocrine-discrupting chemicals (EDCs). Eighteen of the 150 chemicals detected were found in concentrations above EU regulatory limits, with 109 and 113 being nonauthorized substances and NIAS, respectively [151,336].

##### E-waste

Discarded electronic products, or *e-waste*, include small and large household appliances, computers, mobile phones, lighting, tools, toys, sports equipment, medical devices and batteries, circuit boards, plastic casings, cathode-ray tubes, activated glass, and lead capacitors [346]. Approximately 20% of e-waste by weight is plastic [347].

Plastics commonly used in the manufacture of electronic equipment are acrylonitrile butadiene styrene, PS, PC, PVC, PE, and PP [348]. These materials are used for electrical and thermal insulation as well as for manufacture of intrinsically conducting polymers, screens, casings, cables, films, and machine parts [348]. Plastic additives in electronics vary in their amount and include antioxidants (~1%), heat and light stabilizers (up to ~5%), plasticizers (e.g., DEHP ~50% in films and cables), and colorants (~1%) as well as mold release agents, foaming agents, mineral fillings, and coupling agents [348]. More than 200 different types of flame retardants are used and can constitute up to 15% of the plastic in electronic products [348]. Flame retardants include chlorinated, brominated, phosphorus-based aluminum-trihydrate and its derived inorganic rehydrate compounds. Metals, either in plastics or from other sources, are also present [348]. All of these toxic additives are found in e-waste [348].

Because of its chemical complexity and toxicity, e-waste and e-waste recycling pose significant hazards to human health [349,350]. OPEs, toxic metals, and POPs, such as PCBs, BFRs, and PFAS, are released into the environment in e-waste recycling operations—both formal and informal [351,352,353]. Even in a formal e-waste recycling plant in Sweden, despite industrial hygiene improvements that successfully reduced occupational exposures, workers had higher serum PBDE concentrations than unexposed controls [354]. Similarly, in Canada, e-waste recycling workers have been shown to have higher concentrations of OPE metabolites in their urine and of PBDE and lead in their blood compared to glass recycling workers [355]. PBDE concentrations were associated with changes in thyroid function, and OPE metabolite concentrations were associated with changes in sex hormones [355].

The hazards of e-waste recycling are magnified in low-income and middle-income countries (LMICs), where much recycling occurs in the informal sector, emissions controls are few, and a substantial fraction of the workers are young children and women of child-bearing age. E-waste recycling in LMICs poses threats to the health of workers and residents of nearby communities [356,357,358]. In India, for example, the average concentrations of PAHs, phthalates, BPA, and toxic metals in soil were higher in informal e-waste recycling sites than in other dumpsites [359]. Analyses of air, dust, and soil close to e-waste recycling facilities in southeast China revealed higher concentrations of BFRs, POPs such as PCBs, polychlorinated dioxins and dibenzofurans (PCDD/Fs), perfluoroalkyls (from fluoropolymers), and PAHs (including pyrene and benzene derivatives) compared to control areas. Metal(loid)s (lead, chromium, cadmium, mercury, zinc, nickel, lithium, barium, and beryllium) were also detected in these samples [346].

Open burning of e-waste, such as plastic-covered cables to dispose of plastics and recover copper and other metals, is an especially hazardous practice [356,357,358]. Open burning of plastic-coated cables generates dense clouds of black smoke that can contain PAHs, dioxins, and VOCs such as benzene.

Exposures to workers, children, and pregnant women living within or near unregulated e-waste recycling sites have been associated with a range of negative health outcomes, including changes in thyroid function, altered gonadal hormone levels, adverse birth outcomes, and altered growth due to exposure during pregnancy (see systematic reviews [346,360]).

#### Incineration—Controlled and uncontrolled

Because they are organic carbon, plastics can be incinerated. Controlled combustion with sufficient oxygen at very high temperatures (>1,000°C) mainly produces water, CO_2_, and trace chemicals [361]. However, this idealized type of combustion requires well-resourced infrastructure, which is mostly lacking in the LMICs where much plastic incineration occurs. The consequence of increasing export of plastic waste from higher-income countries to LMICs is uncontrolled waste disposal using landfilling and open fires [362].

A systematic review of open burning of plastic waste, mainly in the Global South, examined emissions of eight hazardous substances, including BFRs, phthalates, dioxins and related compounds, BPA, PM, and PAHs [362]. The authors concluded that large quantities of mismanaged waste, including plastic, are threatening the health of ~2 billion people in LMICs, with highest risk to an estimated 11 million waste pickers who lack safe workplaces and protective equipment. Waste incineration, including plastic waste incineration, is estimated to account for 39% (approximately 334 million kg) of global atmospheric aerosol emissions [363]. These aerosolized materials eventually precipitate to accumulate in soils and sediments [362,364].

Mixed waste includes general household waste, tires, and agricultural and construction materials, much of which contains, or is made of, plastics. Uncontrolled incineration of mixed waste, either from landfill or backyard burning, releases a multitude of toxic substances, including PM, PAHs, PCDDs and related compounds, BFRs (bromophenols, HBCDD, PBBs), VOCs, and semi-VOCs [363,365]. Open burning of PVC plastic is particularly problematic [366]. During uncontrolled, low-temperature, open combustion, PVC acts as a chlorine donor, which leads to the substantial formation of dioxins and furans that can become airborne [366]. Estimates of the health impact of PCDDs vary in different regions. For example, PCDD release during open burning of municipal solid waste in India is associated with 0.1–0.2 excess cancer cases per 100,000 people, and co-incineration of waste and coal in Poland indicates excess cancer cases of 4.5–13.2/100,000 [362].

Additional toxic emissions from PVC incineration include carbon monoxide, hydrogen chloride, and PAHs. Burning of plastic waste is estimated to account for 39% of global PAH emissions [362]. PAHs are potent human carcinogens and have been estimated to contribute to 8.7 cases of cancer per one million people exposed [367].

BFRs are another hazard in plastic waste combustion. Although the manufacture of many of the most highly toxic of these compounds is now prohibited by the Stockholm Convention [187,368], these older BFRS can still reside in legacy plastics and thus enter the plastic waste stream [369]. Analysis of plastic waste, as well as virgin and recycled plastic, revealed a wide range of BFRs, including bromophenols, dibromophenols, hexabromocyclodecane stereoisomers, and PBDEs [369]. Acrylonitrile butadiene styrene, PS, and PE can have high concentrations of BFRs [370]. Incineration of plastic waste results in the release of gases, particulates, and ash and the formation of brominated dibenzo-*p*-dioxins and dibenzofurans [371,372]. Analyses of hair samples from populations near e-waste recycling sites indicate that younger people (aged 15–45 years) who were more likely to be involved in recycling and waste management had higher concentrations of brominated compounds than children or older adults [371,373]. PBDEs have been linked to reduced birth weight, type 2 diabetes, endometriosis, cardiovascular disease and cancers [26].

Toxic metals in combusted plastic waste include mercury, cadmium, lead, chromium, and nickel. Mercury and lead are neurotoxic, while chromium and nickel are carcinogenic [374]. Antimony is used as a synergist in the production of BFRs, and arsenic is used as a biocide [170]. Although these metal(loid)s do not tend to migrate from plastics during use, they are released into airborne and precipitated soot during plastic incineration [362].

Little is known of the fate during recycling and waste disposal of the multiple other chemicals incorporated into plastics. A study that examined open burning of plastic materials such as shopping bags, roadside trash, and landfill waste found elevated concentrations of two chemicals—1,3,5-triphenylbenzene and tris(2,4-di-tert-butylphenyl)phosphate)—that are not found in wildfire smoke [375]. A study of atmospheric aerosols from urban, rural, marine, and polar regions showed a positive correlation between 1,3,5-triphenylbenzene and BPA levels [376], a finding that suggests that open burning of plastic waste is a source of widespread atmospheric BPA [375]. In Europe, where incineration is controlled, atmospheric sources of BPA as well as plasticizers are thought to contribute to human exposure [377].

#### Waste-to-energy

Waste-to-energy facilities, euphemistically called *thermal recycling* facilities, convert plastic waste to energy. This conversion involves the production of a wide range of hazardous chemicals, including chlorine, hydrogen chloride and phosgene, hydrogen cyanide, and ammonia as well as formic acid, formaldehyde, benzene and its derivatives, phenol, and PCDD/Fs. Depending on the facility, substantial quantities of these chemicals will be released to the atmosphere [321]. The main sources of these toxic combustion products are PVC and condensation polymers such as PUR, PA, and phenyl-formaldehyde resins as well as chemicals in plastics. Waste-to-energy conversion also generates CO_2_.

Under controlled conditions, formal waste-to-energy plants may remove a large portion of these pollutants from their emissions. For example, in China, while the waste-to-energy incineration capacity increased by 150% from 2015 to 2020, the total emissions of toxic gases decreased by 42.46%–88.24% due to improvements in gas cleaning treatment [378]. However, co-incineration of fossil fuel with 15% of refuse-derived fuel (containing 35% plastic, 30% paper, 20% wood, and 15% textiles) in a cement plant in Spain has been shown to emit similar amounts of PM, PCDD/Fs, and metals compared to normal operations with 100% fossil fuel [379].

While there are limited investigations of the health impacts of exposure to waste-to-energy emissions, a recent systematic review [380] reported that depending on the use of sorted/unsorted waste and gas cleaning technology, waste-to-energy processes may be associated with increased cancer and noncancer risks [381].

#### Particulate matter

##### Incineration

When plastic is burned, airborne PM is released. PM consists of “black carbon” [382] in the form of char or ash, PM_10_, and PM_2.5_ [362]. It can contain heavy metals, VOCs, PAHs, and PCDD/Fs [383,384,385,386]. Plastic burning has been estimated to contribute to 6.8% of PM_2.5_ in Nanjing, China [387]; 13.4% of PM_2.5_ in Delhi, India [388]; and up to 3% and 15% of PM_2.5_ in Atlanta, Georgia, and Dhaka, Bangladesh, respectively [389].

Evidence suggests that PM solids together with PAHs may be more deleterious to health than PM alone [390]. Thus, PM_2.5_-bound PAH are both carcinogenic and mutagenic [77] and may be linked to immunological and developmental impairments and reproductive abnormalities [391].

##### Microplastics

Data are lacking on the contribution of MPs to airborne PM, and so respirable exposures in humans cannot be estimated reliably [171]. However, in an urban setting, MPs were found in all air samples, with deposition rates ranging from 575 to 1,008 MPs/m^2^/day, the majority (92%) of which were fibrous with 15 different petrochemical-based polymers being identified [392].

Nevertheless, airborne MPs have been identified as an emerging source of PM pollution [393,394]. MPs can be released into the atmosphere from a range of sources, including compost spreading, wastewater sludge, tires, textiles, and paint [393,394,395], and are potentially transported by wind currents. Several MPs have been detected in the atmosphere in various forms (fibers, fragments, film) and include PE, polyester, and PUR [396]. However, little is known about the extent to which people are exposed to airborne PM MPs, and further research is required to better understand the implications for human health.

#### Landfill

Plastic waste is projected to almost triple in volume globally by 2060, with half still being landfilled at that time and less than 20% recycled [14]. The amount of solid waste that enters landfills varies widely across countries [239,397]; for example, Switzerland recycles 25%, uses 75% for energy recovery, and has no landfills. Other European countries, by contrast, report 20% recycling, minimal energy recovery, and 80% landfill [239]. Formal landfills in Europe accumulated over 5.25 Gt of waste between 1995 and 2015, of which 5%–25% by weight is estimated to be plastic [398]. This contrasts with other global regions, which often lack formal landfill facilities [399].

Landfilling is associated with environmental pollution, including groundwater pollution due to the leaching of organic and inorganic substances contained in the waste, odor pollution from degradation products, and pollution of surface waters from runoffs [400]. Air pollution is an important negative impact of gas emissions from landfills, with the GHGs methane and CO_2_ predominating [397]. Fugitive aromatic compounds with different levels of dispersion are also emitted. These include BTEX and naphthalene. Analysis of concentration profiles emitted from the working face of municipal solid waste dumpsites in China showed wide variations. For example, toluene levels were up to 90 µg/m^3^ within 200 m and dropped to approximately 12 µg/m^3^ at 800 m; benzene levels were 12 µg/m^3^ within 200 m and dropped to approximately 2 µg/m^3^ at 800 m [401]. Potential carcinogenic risk zones were also calculated. The carcinogenic risk impact distances for benzene and ethylbenzene were 710 ± 121 m and 1,126 ± 138 m downwind of the landfill; cumulative carcinogenic risk distances were higher at 1,515 ± 205 m, and, for worst-case scenarios, the cumulative risk was estimated to persist at over 4 km from the landfill site [401]. Flame retardants (PBDEs) have also been detected in landfill [402].

### Leakage of Plastics to the Environment

**Figure 2.6:**
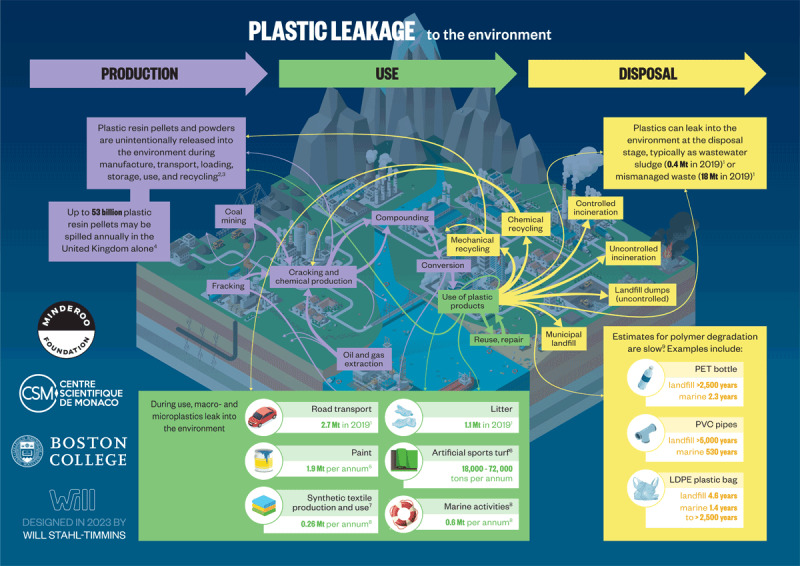
**Plastic life cycle: Plastic leakage.** Plastic and plastic-associated chemicals leak into the environment across all stages of the plastic life cycle. Chemical-laden macroplastics constitute the bulk of plastic leakage. Plastic polymers can take many years to degrade in the environment with the rate of degradation depending on many factors such as temperature, light and mechanical action.^[9]^ Mt, Megatons; PET, polyethylene terephthalate; PVC, polyvinyl chloride;LDPE, low-density polyethylene. **References**: ^[1]^(Organisation for Economic Co-operation and Development (OECD), 2022a); ^[2]^(Karlsson *et al.*, 2018); ^[3]^(Organisation for Economic Co-operation and Development (OECD), 2021); ^[4]^(Cole and Sherrington, 2016); ^[5]^(Paruta, Pucino and Boucher, 2022); ^[6]^(Hann *et al.*, 2018); ^[7]^(Periyasamy and Tehrani-Bagha, 2022); ^[8]^(Ryberg, Laurent and Hauschild, 2018); ^[9]^(Chamas *et al.*, 2020). *Credit*: Designed in 2022 by Will Stahl-Timmins.

Lack of end-of-life management for plastic products, particularly in LMICs, coupled with the economics of cheap virgin plastic versus expensive recycled plastic has resulted in the failure to recover most plastic-based materials and retain their economic value [65,403]. As a result, there is significant “leakage” of plastics throughout the plastic life cycle out of the economy and into the environment (Figure 2.6). In 2019, an estimated 22 Mt of plastics leaked into the environment, with macroplastics accounting for 88% and manufactured MPs for 12% [5].

Vast quantities of plastic waste from developed countries are exported to LMICs [404,405]. China was previously the largest destination for plastic waste, with an estimated 6.6 Mt of plastic waste imported in 2017 (~54% of total exported plastic waste) [405]. Following China’s January 2018 ban on the import of nearly all plastic waste [406], exports from developed countries shifted to Southeast Asian countries, including Thailand, Malaysia, Vietnam, and Indonesia as well as Turkey [49]. These countries often lack the infrastructure to properly manage plastic waste [5,399], thus increasing the likelihood of environmental leakage [407,408,409]. The volume of this leakage is estimated to have been 19.4 Mt in 2017 [399], of which 58% came from Asian countries, including 23% from China [5].

Other sources of macroplastic leakage include littering and marine activities. Littering is the second-largest contributor, with an estimated 1.1 Mt of leaked plastic attributed to littering in 2019 [5]. Littering mainly involves incorrect disposal by consumers of plastic products, most commonly single-use plastics such as plastic packaging, straws, and cutlery. Marine activities, including fishing, can lead to copious amounts of macroplastic leakage directly into the ocean [399], largely in the form of abandoned, lost, or otherwise discarded fishing gear (ALDFG) [410]. A recent study estimated that 75%–86% of floating plastic mass in the North Pacific Garbage Patch can be attributed to fishing activities [318].

#### Microplastics

Microplastics, or MPs, is a term used to denote plastic particles less than 5 mm in diameter. MPs are further classified into two categories: *primary MPs* and *secondary MPs* [5]. Primary MPs are plastic particles that have been manufactured to a small size and intentionally added to consumer products for cosmetic and biomedical purposes; they also include microfibers that are shed from synthetic materials. Primary MPs can leak into the environment from multiple sources across the plastic life cycle. Secondary MPs, on the other hand, are small plastic fragments that arise from the degradation (breakdown) of larger plastic items, particularly plastic litter, as a result of natural weathering processes after they have entered environment [5].

*Nurdles* are tiny plastic preproduction resin pellets from which plastic products are manufactured. Nurdles are also considered to be primary MPs due to their size and can be unintentionally released into the environment during their manufacture, land and maritime transport, loading, storage, conversion, and recycling [417,418]. These resin pellets have been found in rivers, waterways, and the ocean and are often seen on beaches along coastlines around the world [419]. Up to 53 billion nurdles may be spilled annually in the UK alone [420].

Microfibers are shed to the environment from synthetic textiles during production, use, and disposal [421]. It has been reported that by the fifth washing cycle, a total of 30,000–465,000 microfibers will have been released per square meter of synthetic fabric [422]. MPs are released into wastewater, the environment, and the human body from multiple sources during use, including abrasion of tires [423], paint (e.g., marine coatings [412]; see Box 2.3), road markings [424,425], and glitters [426]. Another source is microbeads that are intentionally added to rinse-off personal care products (e.g., face scrubs, toothpaste) [427]. Shed microfibers are found in the air [428], wastewater [429], rivers [430], and ocean [431,432].

**Box 2.3 Microplastics and Toxic Chemicals from Paint.**

It has become increasingly apparent that the total volume of marine microplastics (MPs) cannot be solely attributed to mismanaged consumer waste. Therefore, there must be other contributing sources [411].

Paint is increasingly recognized as an important source of marine MPs. Paint, including water-based acrylics, can contain up to 50% plastic, including polyurethanes, polyesters, polyacrylates, polystyrene, alkyls, and epoxy resins as well as additives, adhesion promoters, thickeners, antiskinning agents, and emulsifiers [412]. Antifouling paints contain high concentrations of hazardous inorganic additives as antifouling agents as well as an array of toxic metals, such as Cu^+^/Cu^2+^, tributyl tin^+^ (which was banned in 2008 [413] but is still circulating in legacy materials), Pb^2+^, and CrO_4_^2–^ [412].

Paint MPs and their potentially toxic additives are shed into the environment from ships and other marine megastructures, such as rigs, as well as from road markings and the external surfaces of buildings [414]. Ships have been reported to leave “skid marks” of MPs in the ocean consisting of high-density polyethylene, low-density polyethylene, polypropylene, polyvinyl chloride, polyethylene terephthalate, and polyurethane [411]. Intentional releases of paint MPs into the environment occur during power-abrasion cleaning of ships during dry dock maintenance [415]. To reduce paint shedding in dry dock operations, closed-loop vacuum blasting is being developed commercially as a capture technology [416].

An estimated 42 billion liters of paint are applied to marine megastructures globally each year [415]. With a 20-year life span, or 5% annual loss of marine paint, this equates to 2–3 Mt of paint MPs released to the ocean every year [415].

MPs can be generated in plastic use and during mechanical recycling of plastics, especially during cutting and shredding stages, and are released into the environment or into wastewater [433]. Additionally, MPs can enter the environment via leakage from controlled landfill sites where geomembrane liners fail. Analyses of leachates from active and closed municipal waste sites revealed PE and PP polymers as the predominant MPs, with size ranges of 100–1,000 µm [434]. Furthermore, unregulated incineration of solid waste can also result in the production and release of MPs as PM into the air and on land [418]. MPs can also be formed from degradation of already leaked land- and marine-based mismanaged macroplastic waste [418].

Airborne MPs can travel long distances via atmospheric transport to reach remote areas [435,436,437]. Airborne MPs and MPs deposited on land can be washed into the aquatic environment or into sewage systems via stormwater and/or surface runoff [418,438]. The deposition of airborne MPs in a coastal city has been estimated at 4,885 ± 1858 MNPs/m^2^/day (mean ± standard deviation; range: 82–12,159 MNPs/m^2^/day); the highest level at an urban rooftop correlated with coastal winds, suggesting that airborne MNPs may originate from wave action [439].

MPs detected in wastewater and sewage systems include PE, PP, PS, PA, PET, and PVC [440]. These materials can come from households and industrial activities [441]. Before being released into the aquatic environment, wastewater is treated in wastewater treatment plants. Varying amounts of MPs are removed in these facilities through preliminary, primary, secondary, and tertiary treatment [442], with 88% of MPs removed from wastewater by applying the first three treatment processes and 94% removed with more advanced tertiary treatments, such as reverse osmosis [443].

While the removal efficiency of MPs from wastewater is high, the residual concentration of up to 54 microparticles/L treated wastewater adds up to a significant total amount of MP leakage into the aquatic environment given the very large volume of urban wastewater [443]. Additionally, sewer systems that combine sewage and stormwater through a single pipe can be overloaded during heavy rainfalls [418], and in these circumstances, untreated wastewater, with all its MP content, is discharged directly into rivers and the ocean [444].

MPs removed from wastewater are concentrated in sewage sludge. If applied to the land as a fertilizer, this can be a major source of MPs to the environment, accounting for an estimated 0.66 Mt of all plastic leakage in 2019 [5]. A recent study reported that each gram of dry solid sewage sludge contains 0.01 g of MPs [445]. Due to its high organic and nutrient content, sewage sludge is commonly used as a sustainable soil conditioner or fertilizer on agricultural lands in many countries [446,447]. As a result of this circular process, MPs captured during wastewater treatment may accumulate in terrestrial environments [448] or enter aquatic environments via surface runoff or infiltration into groundwater [449]. More MPs are estimated to enter the soil from the use of wastewater sludge for agricultural purposes each year than MPs entering the ocean overall and freshwater sediments [447,450]. As many as 3,500 MP particles/kg of dry soil have been reported in samples of agricultural soils that were subject to 10 years of continuous sewage sludge disposal [451]. The presence of MPs in sewage sludge poses a threat to soil health and productivity [452,453,454] and could cause harm to soil-dwelling biota [455].

#### Nonintentionally added substances (NIAS)

*The production of intended chemicals results in the unintended production of by-products, transformation products … and impurities with little being known about their potential adverse effects either singly or, indeed as mixtures* [16].

Nonintentionally added substances, or NIAS, is a term that was first introduced in the food industry (Box 2.4). It refers to chemicals that are not intentionally added to foods or other materials, but they enter foods or other consumer goods in manufacturing or via contact materials such as food wrappings [148]. NIAS are now known to include an enormous variety of synthetic chemicals and may outnumber intentionally added substances in food products [456].

**Box 2.4 Nonintentionally Added Substances (NIAS) in Plastic Food Packaging.**

NIAS in plastic products include impurities present in raw materials and/or additives used during production, oligomers and other by-products of polymer production, degradation and transformation products, contaminants from machinery, and contaminants introduced in recycling [148,149,150]. Breakdown products are a major source of NIAS in plastic food contact materials [149], and one breakdown product, 2,4-di-*tert*-butylphenol, is among the most commonly detected chemicals in migration and extraction studies on plastic and multimaterial food contact products [457]. Oligomers are also common. Cyclical silicone (polydimethylsiloxane) oligomers are among the most commonly detected migrants from plastic and multimaterial food contact materials [457], although this may reflect bias due to available methodologies for detection or selection of materials studied. A systematic evidence map of food packaging plastics detected through published migration and extraction studies has recently been developed [148,223].

Migration of polyolefin oligomers into food from polypropylene food containers has recently been demonstrated during microwave heating [223,458]. Multilayer packaging offers an additional level of complexity, with transformation products that can arise from the polymer and additives in plastic layers as well as from adhesives bonding those layers. In a third important group, the contaminants, examples of such contaminants frequently detected in migration and extraction studies of plastic food contact materials, include the solvent toluene and antimony (a catalyst) [223,457].

Recycling can introduce additional NIAS into plastic due to contamination during use (e.g., chemicals from previously-packaged food), disposal (e.g., metals and persistent organic pollutants adsorbed from the environment), and/or recycling (e.g., monomers formed during melting) [150]. Typical recycling-related NIAS in plastic include oligomers, bisphenols, phthalates, and other additives in recycled plastics [148]. One example study of recycled polyethylene terephthalate bottles included detection of acetaldehyde, formaldehyde, 2-methyl-1,3-dioxolane, polyethylene terephthalate oligomers, toluene, xylenes, and cyclopentanone [151].

Migration of NIAS into food depends on concentration of the NIAS in the material, volatility, molecular weight, vapor pressure, hydrophilicity and lipophilicity, food contact surface area, time of contact, temperature, and nature of the food or food simulant (such as lipid content) [148,149,223]. In many cases, toxicity of these substances and their transformation products is unknown [456]. Transformation products may be more toxic than their progenitors [459].

Because NIAS migrating from food packaging plastics include a complex array of predicted and unpredicted substances, toxicological assessments based on classical chemical-by-chemical approaches fail to capture the potential hazards of these materials [149,456]. The inescapable conclusion is that we have insufficient understanding of the chemical and toxicological profiles of common consumer plastic materials, including food packaging, to evaluate consumer safety in use and environmental safety in waste [460].

Plastic-associated chemicals, such as many additives and NIAS, are present in plastic products as mobile and leachable components within the plastic matrix and have been shown to leach from everyday plastic products made from different polymer types [459]. Controlled aging of biopolymers such as PS, PP, PET, LDPE, and HDPE at 40°C over four weeks results in a progressive increase in the release of VOCs. Testing of MP debris collected from a beach led to the release of VOCs, including benzene, acrolein, propanal, methyl vinyl ketone, and methyl propenyl ketone, thus highlighting an additional invisible hazard of plastic pollution [461]. Both new PVC pellets and beached preproduction pellets/nurdles have been shown to leach PCBs and PAHs to seawater [462]. Additionally, beached PS pellets can degrade into styrene oligomers [463], which have been detected in sand samples from 26 countries [464]. In the same study, BPA was found in seawater samples [464].

Leaching of plastic-associated chemicals during use is specially problematic for food contact materials [170,457] because it can result in human exposure via ingestion—e.g., leaching from plastic baby bottles [465] or from PET food containers and drink bottles [336]. A recent systematic evidence map identified DEHP, dibutyl phthalate, BPA, DEHA, and 2,4-di-tert-butylphenol as the five most frequently detected plastic-associated chemicals leaching from food contact materials [457].

Inhalation is another route of exposure to plastic-associated chemicals released during use. For example, semi-VOCs such as PAHs, phthalates, organophosphates, and BFRs in household products (e.g., electronic devices, furniture, carpets) have been shown to vaporize into indoor air [466,467]. Human exposure to plastic-associated chemicals can also occur via the dermal route, as these chemicals leach from products in contact with skin, such as textiles [248] and personal care products [468,469].

#### Environmental degradation of plastic

Degradation of plastic in landfills releases polymer breakdown products [398], and different plastics produce different degradation products, which can include aldehydes and ketones from PE; hydrochloric acid from PVC; pentanes from PP; oligomers of styrene, ethyl benzene, phenol, and benzoic acid from PS; and acetaldehyde, ethylene, benzene, and biphenyl from PET with concentrations in the 0.1–7 mg/L range [398]. These pollutants can be released into air [470] or water [398,471].

Metal(oid)s, plastic additives, and constitutional monomers present in plastic waste can also be released into leachate [398]. For example, BPA concentrations in leachates from municipal waste disposal sites in tropical Asia ranged from sub µg/L to mg/L [472]. For this reason, landfill leachate is considered to be heavily polluted water that requires specialized physical, biological, and chemical treatment [473]. Rainfall on landfill sites, especially on landfills without impermeable bottom liners or protective top cover layers [474], results in dissolution of organic and inorganic pollutants into leachates, which then either seep into the soil and contaminate underground water systems [400] or enter runoffs and contaminate surface waters such as rivers [475,476]. High levels of PBDEs have been reported, for example, in groundwater near open dumpsites [477].

##### Abiotic pathways

Plastic polymers can be degraded in the environment by abiotic pathways such as physical fragmentation or chemically through photooxidation, hydrolytic cleavage, or thermal oxidation (discussed in Section 3). Degradation processes are complex and include depolymerization, chemical modification, and changes in physical properties (e.g., strength, surface strength, integrity). In an ideal scenario, they result in complete mineralization to CO_2_ and H_2_O. For large macroplastics, the most appropriate definition of degradation is overall loss of mass, whereas surface ablation is an important mechanism for small plastic pieces, especially in the marine environment [240].

Degradation rates vary with polymer type as well as the waste material’s physical properties, including size and shape, and polarity, as well as the environmental milieu (air, terrestrial, aquatic, and landfill) in which the waste resides and the milieu’s physical parameters, such as heat, light, temperature, oxygen levels, and pressure [240]. The complexity of degradation processes, as well as the different techniques used to measure it, are reflected in widely varying estimates of plastic degradation rates, with some media reports claiming that some plastic does not degrade at all [240].

Using surface degradation rates (μm per year), average half-lives of plastic items have been estimated at between 2.3 and 2,500 years, or more. Values of greater than 2,500 years were given for studies where no degradation had been detected within the study period. Mean degradation rates for single-use PET water bottles are reported to be over 2,500 years in landfills and 2.3 years in marine environments with acceleration by UV or heat. Degradation rates for PVC pipes were 5,000 years in landfills and 530 years in marine environments, and the degradation rates for LDPE plastic bags are 4.6 years in landfills and a range of 1.4 to more than 2,500 years in marine environments [240]. (See Section 3 for details.)

##### Biotic pathways

Interest in natural enzymatic polymer degradation by actinomycetes, algae, bacteria, and fungi has increased in recent years [478]. Microbial enzymes that degrade PUR, PE, PS, and nylon have been identified [478]. Their mechanisms of action include biodeterioration (surface fragmentation), biofragmentation (extracellular enzymes and free radicals), assimilation (active and passive transportation), and mineralization (end products being CO_2_, acetic acid, and lipids) (discussed in Section 3). Different factors can either enhance or inhibit biotic degradation. For example, the addition of bacterial nutrient sources such as starch or palmitic acid and oxidation with hydrochloric, sulfuric, or nitric acids can accelerate degradation, whereas plastic additives such as plasticizers and flame retardants can inhibit it [478].

The potential for developing engineered biotic degradation methods via various -omics approaches may be substantial [478]. However, scaling for commercial degradation is a challenge, and the extent to which biotic degradation is a viable mechanism for “cleanup” remains unknown. The impacts of this emerging technology on human health are unknown. (See Section 3 for details.)

#### Greenhouse gases

**Figure 2.7:**
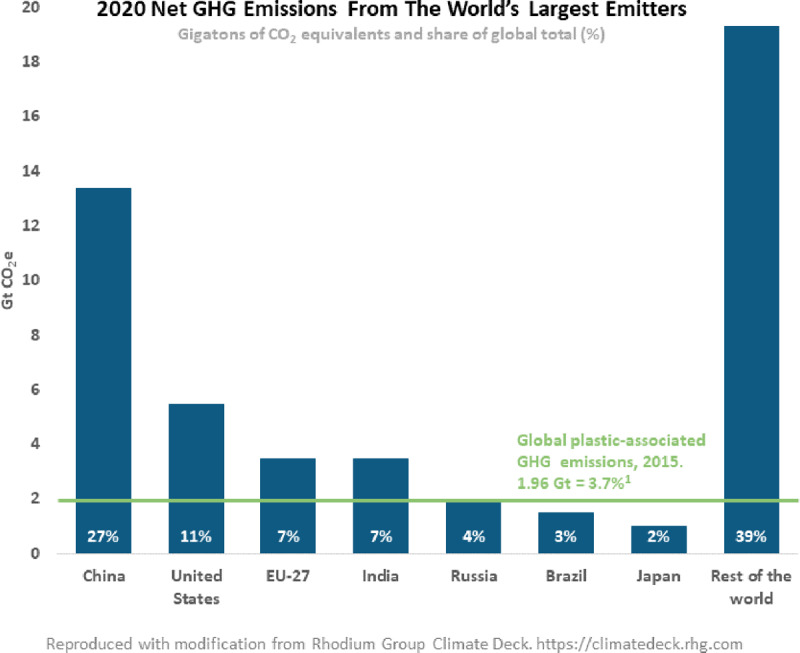
**2020 net greenhouse gas (GHG) emissions from the world’s largest emitters.** Gigatons (Gt) of carbon dioxide equivalents (CO_2_e), including land use, land-use change and forestry, and share of global total (%). In 2015, the annual emissions of CO_2_ and other greenhouse gas from plastics production was 1.96 Gt of CO_2_e, or 3.7% of total emissions (Cabernard *et al.*, 2022)^[1]^. *Source & Permissions*: Adapted from Rhodium Group ClimateDeck. https://rhg.com/research/preliminary-2020-global-greenhouse-gas-emissions-estimates/

Plastic is a contributor to global climate change [14,479,480,481]. GHGs are emitted at every stage of the plastic life cycle [482,483,484], starting with direct and fugitive emissions from the extraction and transportation of fossil fuel feedstocks, direct process emissions from energy-intensive chemical reactions in steam crackers, indirect emissions from energy conversion in the energy sector that facilitates polymerization and conversion, and finally emissions associated with end-of-life processes [479,482,484,485,486]. Combined, the total global plastic-associated GHG emissions are higher than the total net GHG emissions of most individual countries (see Figure 2.7).

By far, the largest proportion of these plastic-associated GHG emissions (90%) is attributed to plastic production [5]. In 2015, the annual emissions of CO_2_ and other GHG from plastics production amounted to 1.96 Gt of CO_2_ equivalents (CO_2_e) [13], with an estimate of 4.5% in 2019 [5]. GHG emissions from plastic are projected to more than double in volume by 2060 [14]. They will consume a significant proportion of the global carbon budget [479] and will undermine the ability of the global community to hold emissions within the targets set in international climate treaties [479,483,487]. In addition to increasing in absolute volume, CO_2_ emissions from plastic may be anticipated to account for an increasingly large proportion of global CO_2_ emissions in future years as emissions from combustion fossil carbon as fuel decreases.

Natural gas released to the atmosphere in gas extraction and transmission principally consists of methane and is an additional contributor to plastic’s total carbon footprint. As much as 4% of all natural gas produced by fracking is lost via leakage to the atmosphere from a combination of venting, flaring, and unintentional leaks [96]. These releases appear to have contributed to recent sharp increases in atmospheric methane and are responsible for a third of the total global increase in methane emissions over the past decade. Methane is a potent contributor to global warming, with a heat-trapping potential 30 times greater than that of CO_2_ over a 100-year span and 85 times greater over a 20-year span [96]. The expansion of shale gas and oil extraction will likely result in further increases in these releases [92].

GHG emissions resulting from the manufacture of plastics differ by polymer type. The largest emissions are associated with the production of polymer fibers used for textiles, followed by PP and LDPE [14]. Growth in plastics production in coal-based economies has resulted in doubling of the carbon footprint associated with plastic manufacture since 1995 [13]. Factors such as the efficiency, configuration, and service life of plant equipment likely influence GHG emissions from plastic production facilities [479].

End-of-life processes account for the remaining 10% of plastic’s GHG emissions, though these vary according to disposal method [14]. Incineration is the disposal method responsible for the largest GHG releases and accounts for approximately 70% of all end-of-life GHG emissions, followed by recycling and sanitary landfilling [5]. Recycling has the potential to reduce plastic’s overall GHG emissions through reduction of primary plastics production via substitution with secondary plastics [14].

### Conclusion

Hazards to human and planetary health occur at every stage of the plastic life cycle—production, use, and disposal. This Section of the Minderoo-Monaco Commission on Plastics and Human Health has summarized these hazards stage by stage.

A key finding that emerges from this analysis is that plastics’ hazards to human and environmental health extend far beyond the visible and now well-recognized hazards of beach litter and marine microplastic. They include the hazards of the many toxic chemicals incorporated into plastics, as well as MNPs, and also the contributions of plastic production to greenhouse gas emissions and global climate change.

In this Section of the report, we have built a framework and structure that we will follow in subsequent Sections, and especially in Section 4, where we will examine plastics’ impacts on human health at each stage of its life cycle.

## Section 3—Ocean Health

### Introduction

The ocean stabilizes Earth’s climate, provides essential ecosystem services, generates oxygen to the atmosphere, and produces protein to feed billions of people. Concern about the negative impacts of plastics (particularly macroplastics and plastic debris) in the ocean first arose in the 1960s with the finding that seabirds were ingesting substantial quantities [488]. In the 1970s, reports of MPs in the ocean and estuaries began to appear [1,489,490,491,492,493]. Concerns about the negative effects of chemicals added to plastics during manufacture resulted from the discovery that intravenous materials stored in PVC bags contained phthalates that had leached from the bags [494]. Ultimately, a personal account published in 2003 describing plastics floating in the North Pacific Ocean far from land, in the presumed “pristine” open ocean environment, raised alarm bells among environmentalists and the public [495]. Although this description of a “garbage patch” resulted in some misperceptions—namely, that of an immense floating island of plastic trash rather than the widespread dispersal of primarily tiny plastic particles—the report of sizeable amounts of plastic debris so far from land was shocking. Widespread plastic debris is, however, only a symptom of a larger problem. A year later, a seminal paper [2] describing MPs as small as 20 µm in beach sediments and plankton samples going back to the 1960s propelled this new subfield of environmental science, which grew with exceptional speed, as indicated by the number of scientific articles on plastic pollution in the ocean and the environment published in the 20 years since [496].

There are now >4,500 publications on the sources, occurrence, distribution, fate, and impacts of plastics (all types) in the ocean, and the implications for human health. Excellent summaries can be found in recent reports [23,497,498,499,500,501]. In preparing this Section of the Commission report, we drew information from these and other reviews [19,41,502,503,504,505,506,507,508,509,510,511,512,513,514,515,516,517,518] as well as from recent primary literature considering the issues of plastics and plastic-derived chemicals in the ocean, their impacts, and their potential to be transferred to humans. Despite the abundance of information, our understanding of the significance and potential for impacts of ocean plastics, including macroplastics but especially small MNPs, is fragmentary. Yet, given the continued growth in the manufacture of plastics, as detailed in Section 2, the amounts entering the ocean and the potential for harm are certain to increase.

Recognizing the great and growing magnitude of the plastic pollution problem, the ECHA recently concluded that, even in the face of incomplete scientific information, there is sufficient evidence of risk to recommend legal actions to reduce the inputs of MPs to the environment [519]. In addition, the world’s nations adopted a resolution in 2022 under the auspices of UNEA to establish a Global Plastics Treaty by the end of 2024 to end global plastic pollution [520]. Thus, as we move toward measures to address the plastics problem, we must continue to seek a greater understanding of the behavior and impacts of plastics in the ocean to better inform the search for solutions (Section 7).

The diversity and complexity of the materials that fall under the category “plastics” are major obstacles to a complete understanding of plastic’s behavior and impacts in the ocean. Thus, as noted in Section 2 and by others [8,20,43,521,522,523,524,525], plastics found in the environment (including the marine environment) collectively include an enormous variety of polymers, sizes (macroplastics and MNPs ranging over at least nine orders of magnitude), shapes, colors, added or sorbed chemicals (thousands), surface chemistries, and other features. Adding to the complexity are the physical, chemical, and biological transformations that occur after the plastics enter the environment [526]. The distribution, fate, and impacts of plastics are strongly influenced by their properties, which vary tremendously in a continuum across the range of features mentioned above. This complexity severely limits our ability to generalize from results of studies examining one or a few types of plastics. In particular, NPs have properties and behavior that are distinct from those of both MPs and engineered nanomaterials [527,528,529]. Indeed, it has been suggested that different types of plastics should be considered separate materials [525]. One promising approach to managing the complexity of plastics is through the use of continuous probability distributions of plastic properties, which focuses attention on the plastics that are most relevant for assessing exposure (including human exposure) and risk [522,524,530].

Plastics contaminate the environment on a global scale, occurring nearly ubiquitously in terrestrial, aquatic (marine and freshwater), atmospheric, and built environments. Here, we focus on plastics in aquatic environments (primarily the ocean), which have been extensively investigated and which have potential impacts on human health both directly (e.g., through consumption of seafood) and indirectly (e.g., through degradation of ecosystems). Readers interested in the presence, fate, and effects of plastics in terrestrial and atmospheric environments are referred to recent reviews of these topics [275,531,532,533,534].

In focusing on aquatic environments, especially the ocean, we diverge from the organizational structure of “production – use – disposal” that is used in other Sections of this report. We do not detail impacts from production of fossil carbon feedstocks in the ocean, although there are impacts associated with obtaining feedstocks in offshore oil production, as there are on land. Sections 2 and 4 detail human impacts from feedstock production. We recognize that overall, as with all oil produced, a small percentage of the oil produced offshore will be used in plastic production. Nonetheless, impacts of feedstock production on life in the ocean derive from spills during transport such as the Exxon Valdez or accidents such as Deepwater Horizon. Impacts of oil in the sea have been reviewed substantially for many years [535]. There may also be impacts of sounds from well operation or from tanker vessels [536,537,538]. The over-riding concerns with plastics in the ocean are more with distribution, degradation, impacts in the ocean, and also with how plastics in the ocean may impact humans.

### Distribution of Plastics in the Ocean

For nearly as long as plastics have been made, they have escaped into nature, including the marine environment. However, the rate of escape has increased markedly since the start of widespread mass production in the 1950s. Marine life has encountered plastic debris in the ocean at least as early as the mid-1960s when global plastic production was less than 2% of what it is today (See Section 2) [488].

Much of the scientific literature on plastics has reported on contamination of the marine environment—from shorelines and coastal environments to the open ocean, from the sea surface to the seafloor, from the tropics to the poles, and in association with increasing numbers of marine species. The most comprehensive datasets on the abundance and distribution of plastics in the marine environment come from beach cleanups [539] and sampling the ocean surface [540,541]. Ocean Conservancy has sponsored an annual International Coastal Cleanup for more than 30 years to remove trash from beaches worldwide and document the most commonly found items while simultaneously engaging and educating local communities about the problem. Scientific measurements of plastics in the ocean itself have mostly been conducted at the sea surface by towing nets designed to sample plankton (with mesh size typically ~0.2 mm or larger). The first reports of floating plastic particles were from plankton surveys in which this anthropogenic material was incidentally collected along with the plankton of interest [1,490].

Larger, highly visible floating plastic debris—macroplastic—has been quantified by visual surveys from ships or aircraft since the 1980s [542,543,544]. These very resource-intensive surveys are few, however. While remote sensing technologies offer promise to identify and quantify large floating plastic debris, such approaches are still under development [274,545,546]. Consequently, data on the number or mass abundance of floating plastics larger than centimeters in size are scarce.

For decades, scientists have encountered and measured large debris composed of plastics and other materials on the seafloor, from continental shelves to the deep sea [547]. As with floating MPs, data on these materials were initially collected by scientists trawling or imaging the seafloor for other scientific purposes (e.g., [548,549,550]). Given the substantial challenges of accessing the deep ocean, it is perhaps surprising that there are more scientific publications documenting large debris on the seafloor than that floating at the sea surface. Much of the seafloor literature reports ALDFG [551], such as traps and nets (e.g., [552,553]), or debris made of denser materials and/or consumer products that sank close to presumed coastline sources (e.g., [554,555]).

As public and scientific interest in MPs has grown, new methods have been employed to measure ever-smaller particles throughout the marine environment, including on beaches [556,557], in deep ocean sediments [558], deeper in the ocean water column [559,560,561], and even in sea ice [562,563]. Chemical characterization (fourier-transform infrared spectroscopy or Raman spectroscopy) is critical to confirming the identity of the smallest particles that can be isolated from environmental samples, including biota. Currently, there are no reliable methods to identify and quantify NP particles and sources in environmental samples, although there is some evidence of NP generation from laboratory weathering exposures [564] and colloidal material isolated from seawater [565]. It is likely that NP particles behave differently in the environment than larger particles of the same polymer composition [566], and much work remains to understand their abundance, distribution, and potential risks [527,528,529].

The scientific literature includes numerous reports of marine life, ranging from phytoplankton and zooplankton to large marine animals, interacting with plastic debris, especially through entanglement and ingestion (reviewed [505,567,568]; see section “Plastics in Aquatic Food Webs and Seafood” below for details). The diversity of plastics in terms of their particle size and morphology, their polymer and chemical (additive and sorbed) composition [8,20,522], as well as a lack of clarity on relevant environmental exposure rates, makes risk assessment for individual categories of plastic debris a complex and challenging task.

It is now clear that MPs contaminate every part of the marine environment globally. While variable in their regional concentrations, they are abundant and widespread in their global distribution. The level of plastic contamination in any particular location will be influenced not only by proximity to inputs or sources of plastics to that location but also by their transport in the atmosphere and ocean. This transboundary transport can render some geographies, such as remote islands [569,570] and polar regions [571,572], particularly vulnerable to the accumulation of plastic debris that may have originated thousands of kilometers away. The ocean may also be a source of MPs to coastal regions, through transport of sea spray from breaking waves [439,532,573]. Some have argued that discharges of synthetic chemicals, including plastics, onto land and into the ocean have reached a critical threshold that meets the criteria for planetary boundary threats [16,574], potentially with negative impacts on global health.

#### Sources

Although constrained by a lack of direct measurements of fluxes of plastics (of any size) to the ocean, it is presumed that most marine plastic debris is released to the environment on land [497], where most plastics are used, and then transported by water (e.g., rivers, streams, waves, and tides on beaches) or air (wind) to the ocean [23]. The first global estimate of the input of plastics from municipal solid waste generated on land to the ocean—4.8–12.7 megatons in 2010 [24]—was computed using country-level estimates of plastic waste generation and waste management practices reported by the World Bank [575]. These data were used to model the amount of plastic waste generated by populations in coastal regions that was not properly captured and contained in waste management systems and, therefore, available to enter the environment, including the ocean. This provided a first estimate of the scale of the global problem and resulted in a strong recommendation to further refine the estimate with more robust national data on plastic municipal solid waste generation and its management and environmental measurements of plastic waste.

The same basic modeling framework was subsequently adopted to estimate plastic waste input from land to inland waters that ultimately drain to the ocean [407,408], with subsequent refinements added to model the likelihood of river transport [409] taking into account the complexity of flow in watersheds [576]. Additional studies have used a similar framework to estimate the amount of plastic waste entering aquatic ecosystems globally (19–23 megatons in 2016 [577]; 9–14 megatons in 2016 [57]).

In addition to the broad assumptions built into these simple models, they did not always consider known activities that influence plastic waste handling and fate, such as burying, burning, illegal or unregulated dumping; unregulated or illegal discharges; informal waste collection (e.g., by waste pickers); and the international trade of waste. Further, until recently, few field data were available to anchor estimates of environmental fluxes of plastics, such as from land into rivers and rivers to the ocean. Although data are increasing, the methodologies used vary substantially according to study, the size of debris measured (large plastics vs. MPs), and the environmental matrix sampled (e.g., riverbank, water surface or sediments; river mouth or estuary). Estimating fluxes of plastic debris from beaches or shorelines to the ocean, including estuaries, is even more complex because, in contrast to rivers, fluxes may be erosional or depositional due to local bathymetry and coastline geometry, and they also vary with ocean tides. For these and other reasons, there is extremely high spatio-temporal variability in plastic flux measurements, and much work remains to constrain estimates of total plastic flux from land to ocean (Box 3.1).

**Box 3.1 How Much Plastic in the Marine Environment Originates From Single-Use Products?**

While it is possible to confirm the polymer identity of most microplastics, it is generally not possible to identify the object from which microplastic fragments were generated. However, most of the floating plastics collected in plankton nets are polyethylene and polypropylene [590], which are high-production polymers largely used in packaging and other single-use applications [591]. Item counts from decades of beach cleaning and beach surveys have consistently been dominated by categories of single-use items, including packaging, food and beverage service ware, and cigarette butts [23]. In 2017, for the first time, Ocean Conservancy’s Top Ten List of items most frequently collected in the International Coastal Cleanup were all composed of plastics, displacing non-plastic items such as paper bags, glass bottles, and aluminium cans [592].

Many additional sources of plastics to the ocean are not captured in estimates of leaked mismanaged waste from land. A presumably very large source is ALDFG and aquaculture gear, which has yet to be robustly estimated, even to an order of magnitude [551]. Immense amounts of debris of all material types are input to the ocean due to catastrophic events such as tsunamis, hurricanes, or floods [515,578,579]. Plastic debris is also lost during shipping, commercial, recreational, and other maritime activities [19,497,499,500].

MPs have additional sources and pathways to the marine environment. Sources include generation from large debris [580,581,582], release as a consequence of wear or abrasion of products while they are in use, such as tires [583,584,585,586] or clothing [587], and the direct release of small pieces of plastic used in applications such as cosmetics [496]. MPs from these sources may be carried to the ocean in wastewater [588] and stormwater outflows [589], as well as by atmospheric transport [531,532]. MPs can directly enter the ocean during use as industrial scrubbers or as paint from vessels or structures, for example, and plastic resin pellets, flakes, and powder (the “raw materials” of plastic products) can be lost during transport.

#### Transport

The three-dimensional hydrodynamic processes that transport debris bidirectionally between shorelines and the sea are complex and largely determined by local characteristics such as the shape of the shoreline, seafloor bathymetry, and local wind and sea conditions [515]. Once in the ocean, floating plastics are transported by the surface flow resulting from the interaction of processes including large-scale current systems that form ocean gyres [593], tides [594], surface waves [595], and vertical motion associated with convection [596], ocean turbulence [597,598], and frontal dynamics [599]. For larger debris that protrudes above the sea surface, surface winds also contribute to the direction and speed of transport (windage effect).

The highest concentrations of floating MPs collected in plankton nets in the open ocean have been measured in subtropical ocean gyres, consistent with theoretical ocean circulation models [600,601,602]. Very high surface concentrations have also been measured in the Mediterranean Sea’s semi-enclosed and highly populated basin [603,604,605], where surface flow is unidirectional into the basin. Albeit in lower concentrations, floating MPs have been detected throughout the open ocean, including in polar regions [571,572] and other regions remote from human populations. For small MPs (<100 um), atmospheric transport also carries particles over long distances where they may be deposited in the ocean far from land sources such as roads, agricultural fields, and population centers [531,532]. MPs also may be transported from the ocean back to land via coastal sea spray [573].

Even in the high accumulation zones of the subtropical ocean gyres, large spatiotemporal variability in surface plastic concentration is typically observed [606,607]. Variations in sampling methodology and conditions when sampling (e.g., wind speed, sea state) can explain some of this variability [608]. Still, the complex interaction of multiple physical processes such as those listed above and the diverse physical characteristics of the debris itself currently renders the prediction of “hotspots” of accumulation at small scales (tens of kilometers or less) extremely challenging.

There is clear evidence of the transport of plastic debris below the sea surface, but the factors involved are less well understood for all but the densest items, which sink relatively quickly close to their point of entry. Neutrally buoyant or slowly sinking particles could be carried long distances by typically slower-moving, mainly horizontal, deep ocean currents. Even for initially buoyant items, deep sea sediments are the presumed final sink for MPs and other debris in the ocean [560,609]. Laboratory studies and modelling studies have demonstrated the sinking of particles (even initially buoyant plastics) due to biotic processes such as biofilm formation [610,611,612], aggregation in marine snow (aggregate particles comprising organic material, detritus, fecal pellets and more, which continuously fall from upper regions of the oceans to the depths) [613], and fecal pellet formation after ingestion [614]. While MPs have been found in the deep water column [615] and in seafloor sediments [558], direct measurement of vertical MPs fluxes in marine snow has only recently been achieved [547,560].

In addition to biologically mediated processes, such as biofilm growth on plastic debris and incorporation of MPs into fecal pellets, there can also be direct biological transport of plastics by birds, fish, and other marine life encountering debris by ingestion or entanglement. Biological transport can occur between ocean and land, as in the case of seabirds foraging at sea to feed their chicks on land [616], or within the ocean when animals swim or migrate at one depth or across a range of depths. For example, mesopelagic fish that feed near the sea surface at night and migrate to depth during the day could transport MPs vertically through ingestion and egestion [617,618]. Not yet well understood, the relative effect of biological transport compared to physical transport may be quantitatively small but still biologically or ecologically important.

#### Mass Balance: The Case of the “Missing Plastics”

The concept of “missing” plastics in the ocean was first considered by Richard Thompson and co-authors in their 2004 article, “Lost at Sea: Where Is All the Plastic?” [2]. They reported an increase in the abundance of microscopic plastic particles in plankton samples collected since the 1960s in the northern North Atlantic, whereas previous ocean and beach samplings had not shown an increase in large plastic debris that might have been expected during a time when plastic production had greatly accelerated, and considering plastics’ persistence in the environment. They suggested that this increase in particles they dubbed “microplastics” (likely from the fragmentation of larger items) might explain the difference.

Since then, the concept of “missing plastics” has evolved to describe a quantitative mismatch between estimates of plastic mass input to the ocean (with presumed negligible outputs) and the estimated standing stock of plastics in major marine compartments or reservoirs [19]. This mass balance exercise is analogous to carbon budgeting carried out in the 1990s to understand the fate of anthropogenic CO_2_ released into the atmosphere [619].

The goal of an environmental mass balance analysis for plastics should not be to make precise estimations, because standing stock estimates cannot capture the complexity of time-dependent processes, including the fluxes between reservoirs and the physical and chemical transformations that occur within them. Instead, the mass balance approach is most useful to identify the major knowledge gaps in understanding the flow of plastics from sources to marine sinks. This knowledge is critical to inform other goals, such as exposure and risk assessment or hotspot identification for prevention or mitigation activities.

When Jambeck et al. [24] made the first estimate of plastic waste entering the ocean from land, they compared their estimate (4.8–12.7 megatons in a single year) to the only available standing stock estimates of plastic debris in the ocean, which reported the mass of plastics floating at the sea surface, mainly in sizes collected by plankton nets [600,601,602]. The estimated annual flux into the ocean was 10 to 1,000 times larger than the estimated mass of floating plastics. Further, because the input estimate represented only one of the presumed largest sources of plastic input to the marine environment, others of which include fishing and aquaculture gear and losses due to catastrophic events, it was likely an underestimate.

Later environmental assessments continued to demonstrate a large quantitative mismatch with the annual input estimated by Jambeck et al. [24], even when paired with numerical ocean models to better estimate surface plastic concentrations in unsampled regions (Table 3.1).

**Table 3.1:** **Estimates of the amounts of plastic debris floating at the ocean surface.** Global estimates range from 0.09% to 12% of the 4.8–12.7 million metric tons of plastic waste estimated to enter the ocean in [24] Regarding debris size, most plastics collected in plankton nets are millimeters in size or smaller, although many studies do not report particle size distribution. Here, “nominal” refers to all plastic debris collected in plankton nets, the majority of which is likely to be microplastic (<5 mm in size).


ESTIMATED MASS	MEASUREMENT METHOD	DEBRIS SIZE	REGION(# MEASUREMENTS)	STUDY

1,100 tons	Plankton net(0.335 mm mesh)	Microplastic (nominal)	Western North Atlantic Ocean (6,136)	(Law *et al.*, 2010) [600]

21,290 tons	Plankton net(0.335 mm mesh)	Microplastic (nominal)	Eastern North and South Pacific Ocean (2,529)	(Law *et al.*, 2014) [607]

6,350–31,750 tons	Plankton nets(0.2 mm–1 mm mesh)	Microplastic (nominal)	Global (3,070)	(Cózar *et al.*, 2014) [601]

66,140 tons	Plankton net(0.33 mm mesh)	0.33–200 mm	Global (680)	(Eriksen *et al.*, 2014) [602]

202,800 tons	Visual survey transects	>200 mm	Global (891)	(Eriksen *et al.*, 2014) [602]

93,000–236,000 tons	Plankton nets(0.15–3 mm mesh)	Microplastic (nominal)	Global (11,854)	(van Sebille *et al.*, 2015) [1561]

82,000–578,000 tons	Plankton nets & filtered continuous seawater intake(0.1–0.3 mm mesh)	Microplastic (nominal)	Global (8,209)	(Isobe *et al.*, 2021) [541]


Although one should not expect standing stock estimates from the sea surface alone, especially from a limited range of debris size, to equal an annual input estimate, the magnitude of the mismatch revealed a major knowledge gap in understanding plastic flows in the marine environment. Proposed explanations for this mismatch include:

1. Plastic debris, across the size spectrum (possibly nanometers to hundreds of meters), may reside in other marine reservoirs. This includes plastic debris larger or smaller than that typically collected in plankton nets floating at the sea surface; plastics suspended or sinking in the water column; and plastics residing on the seafloor, on coastlines, in sea ice, in marine life, or buried in seafloor sediments. Shorelines (beaches) and the seafloor have been proposed as major reservoirs of both macroplastic and MP debris, and many individual studies document plastics in these compartments around the globe (e.g., [620,621,622,623]). However, as both individual and comparative studies demonstrate, measured debris abundance can vary by orders of magnitude, even on short time scales [624,625]. Thus, extremely limited and highly variable data, with large uncertainties, have hindered a robust determination of the largest marine reservoirs for plastic debris.
2. Global estimates of plastic input to the ocean, or the environment more broadly, have been primarily model-based to date and are poorly constrained with environmental data. As discussed above, the first input estimate by Jambeck et al. [24] was derived from a model based on plastic waste generation and management data, with no direct flux estimates from land to the ocean. Subsequent estimates have used the same general framework to estimate the input of mismanaged waste into different receiving environments, adding increasing complexity and environmental data as it has become available. Perhaps not surprisingly, these estimates roughly agree that millions of metric tons of plastic waste leak into the environment annually (see Table 5.1 in [23]). However, a recent study by Weiss et al. [626] argued that these model-based input estimates may overestimate the flux of plastics into the ocean via rivers by several orders of magnitude. They estimated the global riverine flux of MPs after correcting methodological biases in previous studies arising from assumptions of average particle mass and particle size biases resulting from variable sampling methodologies. When they limited their analysis to riverine data collected comparably to that typically used in ocean inventories (that is, using similar plankton nets), they found that total human population levels in the river basin and river drainage intensity were better predictors of riverine MP fluxes than estimates of mismanaged waste. Although it is not feasible to measure the flux of plastics into the ocean via every pathway, this study demonstrates the current high level of uncertainty in input estimates, even to an order of magnitude.
3. The residence time of initially buoyant plastic debris at the sea surface is poorly known for all debris sizes. While some studies have argued that residence time could be as short as days to weeks in some cases [627], direct evidence of long-lived floating debris suggests residence times could be as long as many decades [621]. Removal times from the sea surface are likely to be strongly dependent on particle size, where the smallest particles (tens of µm in size, including microfibers [628]) could be removed much faster by photochemical degradation (see section Fate of Plastics in the Ocean, below) [629,630], turbulent mixing and dispersion [631,632,633], biofouling [610,612,634], and ingestion by organisms from plankton to large marine mammals. The size-dependent residence time remains poorly understood, as discussed in more detail below.

Although the evidence demonstrates the contamination of the marine environment by plastic debris at global scales, knowledge about the sources, accumulation zones, and sinks of plastic debris remains incomplete. This primarily stems from a gross under-sampling of the marine environment to date, a challenge difficult to overcome for multiple reasons:

- The ocean covers 72% of the Earth’s surface, can extend to more than 10,000 m in depth, and is generally difficult to access.
- Plastic debris ranges from nanometers (potentially) to tens or even hundreds of meters in size, requiring a suite of complementary approaches to measure the entire size spectrum.
- Especially for MNPs, chemical identification is required to confirm that particles are composed of synthetic polymers, at present an extremely resource-intensive task, and one that requires careful interpretation of results [635].
- Even for the best-sampled subset of plastic debris to date—MPs floating at the sea surface—the immense spatiotemporal variability characteristic of environmental measurements requires large data sets of consistent measurements across a variety of scales.

Facing these challenges head-on, new technologies, such as remote sensing of the sea surface from aircraft or satellites and automated particle identification techniques, are actively being developed to better understand the global distribution of large and small plastic marine debris. Advances in knowledge of the baseline distribution will be necessary to inform exposure and risk assessment of this global contamination and to assess the efficacy of mitigation activities.

### Fate of Plastics in the Ocean

**Figure 3.1:**
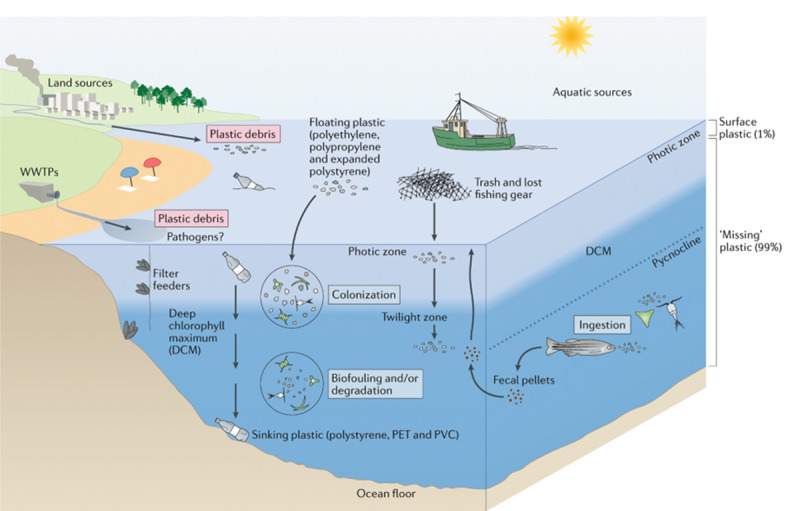
**Fate of plastic debris in the environment.** Diagram illustrating many of the possible pathways over the lifecycle of plastic litter on its journey from land to sea. Plastic debris enters the ocean through both aquatic (rivers, accidental escape at sea) and land-based sources (littering, escape from municipal waste management such as wastewater treatment plants (WWTPs)). Depending on the density of the plastic material, plastic items will remain afloat for a given part of their lifecycle or, as they become weighted down by biofouling, will begin to sink into the water column, ultimately to the ocean bottom. Changes in biofouling with depth may lead to depth oscillations (not shown) before particles end up on the seafloor (Kooi *et al.*, 2017; Rummel *et al.*, 2017; Royer *et al.*, 2021). Mechanical, photochemical and biological forces break down plastic debris into microplastics and nanoplastics that subsequently become incorporated into the marine food web. Organisms such as filter feeders may further concentrate these smaller particles, given their capacity to filter large volumes of water. Microorganisms begin to attach, colonizing plastic in the water within hours, and can include potentially harmful microorganisms, such as disease-causing pathogens. PET, polyethylene terephthalate; PVC, polyvinyl chloride. Figure reproduced from (Amaral-Zettler, Zettler and Mincer, 2020) [641], with permission from Springer Nature and the Copyright Clearance Center. The reproduced figure is not part of the open access license governing the current paper.

Once in the marine environment, plastics experience abiotic and biotic processes that transform their physical and chemical properties (Figure 3.1). These processes may include photochemical degradation from UV irradiation, hydrolysis, mechanical breakdown by wave action and interaction with sediments (abrasion), and biodegradation, principally by microbes. These processes can synergize or antagonize with one another to transform and degrade plastic. For most plastics, these are surface processes; thus, geometries with greater surface area to volume ratio enhance degradation [240]. A plastic product’s structure, properties, and composition (polymer and additives) [526], its geometry (e.g., particle, fiber, sheet) [636], and the local environmental conditions it encounters (e.g., sea surface vs. seafloor) together control the extent to which any one process contributes toward its degradation [240,637,638]. The integration of these processes defines a plastic product’s eventual lifetime in the marine environment.

It is apparent that, for most conventional plastics, the sum of these various processes does not result in mineralization of plastics to CO_2_ and water at a rate that could provide any meaningful mitigation of the increasing input of plastics to the environment [23,639,640]. Nevertheless, the environmental transformations of plastic are relevant for understanding their potential impacts on marine biota.

#### Abiotic Processes

For most plastics, abiotic processes dominate their degradation, particularly for polyolefins with carbon-carbon backbones (e.g., PE, PP, PS) [637]. For example, PS is largely resistant to microbial degradation because breaking the aromatic backbone is energetically unfavorable, but absorption of sunlight results in photochemical oxidation, producing CO_2_ and dissolved organic carbon [629]. Abiotic processes are categorized by their mode of action as either chemical (photochemical, hydrolytic) or mechanical (abrasion) transformations [642].

##### Photochemical

Photochemical processes (i.e., sunlight) transform plastics at the molecular and microstructural levels, manifesting as macroscopic changes. Under appropriate conditions, sunlight can degrade plastics much faster than previously thought, reducing estimated lifetimes from thousands of years to tens of years [629,643].

Photochemical degradation is the process by which light, principally UV radiation, initiates a series of chemical reactions in the plastics that lead to chain scission and crosslinking of the polymer matrix and the addition of new functional groups into the polymer backbone [225,630]. The rapid absorption of UV radiation by plastics limits photochemical degradation to the first 50 to 100 µm into the material, making photochemical degradation a surface-level transformation [644,645]. Photodegraded plastic is often identifiable in the field by its yellowed and cracked appearance [646,647,648].

Photochemical degradation depends on irradiation conditions (environmental dependencies) and the plastic type and microstructure (material dependencies). The amount of irradiation a plastic receives depends on its global location (latitude), local environment (on the beach, ocean surface, or position in the water column), and water clarity (e.g., the presence of other light scattering and absorbing materials) [240,637]. For photochemical processes to occur, the plastic must have unsaturated chromophoric groups that absorb UV radiation [225]. Several of the major plastics (e.g., PE, PP, PVC) do not have UV absorbing groups in their backbone (unlike PS, PET); however, internal (e.g., catalyst residues, unsaturated content, carbonyls) and external impurities (e.g., trace metals, sorbents, additives) can absorb UV [225].

Generally, photochemical degradation is a three-step process: i. initiation, ii. propagation, and iii. termination [649,650]. Initiation occurs when UV radiation is absorbed by the plastic and a radical is formed by breaking a C-H bond. The radical propagates in step two by reacting with oxygen to form a peroxy radical, which can become autocatalytic. The process terminates in step three when two radicals combine to yield inert products that result in chain-scission, branching, crosslinking, and incorporation of UV absorbing oxygenated and unsaturated functional groups. Specific polymer susceptibilities and photochemical reaction mechanisms have been reviewed [225,630,637,650].

Photochemical degradation results in several interrelated physical and chemical changes that span the molecular and microstructural levels of the material, which are relevant to plastics’ interaction with biological systems [650]. At the molecular level, chain-scission reduces the molecular weight of the polymer matrix, and oxidation introduces carbonyls and hydroxyls into the polymer matrix [225,637,650]. A decrease in molecular weight, in turn, reduces mechanical properties [651]. Oxidation makes the surface more hydrophilic [652]. Microstructurally, most plastics are semi-crystalline materials. Amorphous regions are more susceptible to degradation processes [650,653]. This leads to an increase in surface crystallinity. As a result, a mechanical mismatch between the more crystalline surface layer and the less crystalline underlying bulk polymer generates internal stress [654]. To relieve the stress, the surface of the polymer fractures, leading to increased surface roughness and thus increased surface area [655,656]. The collective changes in molecular and microstructural properties contribute to increased brittleness making the material more susceptible to fragmentation [651,657,658]. Along with physicochemical changes, photochemical degradation results in the release of additives [637], the formation of water-soluble products [526,629,643,659,660,661,662], the liberation of CO_2_ [526,629], and, importantly, the shedding of NPs [636,663,664,665,666,667].

Because of these changes in plastic properties, most plastics are modified with additives to impede photodegradation. Additives can include antioxidants (e.g., phenolics and phosphites), hindered-amine light stabilizers, their combination, and benzotriazole-type UV stabilizers (BUVSs), among others [649,668,669]. In contrast, prooxidants and photocatalysts can be incorporated to enhance the photochemical degradation of plastics [670]. Gaining traction is the use of titanium dioxide, a common white pigment and photocatalyst, which has been shown to accelerate photochemical degradation [526,542,671,672,673,674,675,676,677].

##### Thermal Degradation and Combustion

Thermal degradation and combustion of plastics are processes not often associated with marine plastics; however, recent work on ship fires and pyroplastics (see Box 3.2) has revealed that plastics altered by elevated temperature and burning enter the ocean [678,679]. In general, thermal degradation results in the same chemical and physical property changes as photochemical degradation [637,680]. The difference is at the initiation step; instead of UV light, it is thermal energy that leads to radical formation [637]. Combustion of plastics during incineration and open burning of waste results in air pollutants, ash, and charred remnants (Section 2) [362]. The remnants of burning (pyroplastics) have only recently been documented on beaches globally because of their camouflaged appearance, resembling rocks or other natural marine debris [678,679,681]. Owing to their recent discovery, little is known about how the transformations from burning impact plastics’ fate in the marine environment. During the recent M/V *X-Press Pearl* ship fire and plastic spill off the western coast of Sri Lanka, both unburnt plastic pellets and burnt plastics were released [678]. Though unfortunate, this spill provided an opportunity to study pyroplastics in the ocean. Recent evidence has shown that burnt plastics from this spill can be of diverse shape, size, and color (often darker shades), have increased brittleness, and contain soot and toxic, combustion-derived contaminants (e.g., PAHs and heavy metals) [237,678,682,683,684,685,686].

**Box 3.2 New Forms of Plastic in the Anthropocene.**

Abiotic and biotic degradation processes in the marine environment transform plastics physically and chemically into new forms, such as plastiglomerates, plasticrusts, pyroplastics, anthropoquinas, plastitars, and petroplastics, which have properties unlike those of the material that originally entered the ocean and are unique to the marine environment [681,707,708,709].

*Plastiglomerates*: multi-component composites of melted plastic, sand or sediment, volcanic rock, and organic matter, likely formed by the burning of plastic [710,711,712,713,714].

*Plasticrusts*: plastic encrusted on intertidal rocks with other debris often embedded within the plastic, likely formed following the wave-induced collision of plastic with rock outcrops [713,715,716,717].

*Pyroplastics*: burned or melted plastic with geogenic appearance, neutral color, and increased brittleness formed from the burning of plastic [678,679,684,713,714,716,718].

*Anthropoquinas*: anthropogenic material contained within sedimentary rock including plastic formed by the deposition of sediment containing plastic [719].

*Plastitars*: tar encrusted on intertidal rocks with embedded plastic and other debris, similar to plasticrusts [708,720].

*Petroplastics*: agglomerates of oil and microplastics [709,721].

These new forms of plastic debris have only recently been recognized in the environment, and there is uncertainty about their prevalence, distribution, fate, and overall impact on marine systems [681].

##### Hydrolytic Degradation

Hydrolytic degradation is the process by which water reacts to cleave chemical bonds in plastics, principally ester and amide bonds. Some polymers susceptible to hydrolysis include polyesters (e.g., PET, PLA, polycaprolactone, PC), PAs (e.g., nylons), and PUR [637,687,688,689]. The rate and extent of hydrolysis largely depend on temperature, salinity, and pH (environmental dependencies) and molecular weight and crystallinity (material dependencies) [688,689,690]. Hydrolytic degradation results in many of the same physical and chemical changes as photochemical degradation: reduction in molecular weight, new functional groups, increase in crystallinity, reduction in mechanical properties, increased brittleness, increased surface roughness, and increased hydrophilicity [658,687,690,691,692]. Similarly, along with changes in the plastic, other products are liberated, including additives, water-soluble products, and fragmented NPs [637,690]. The time required for hydrolytic degradation under marine conditions varies depending on the type of polymer [240,689,690].

##### Mechanical Degradation

Waves and other motion act to fragment plastics by abrasion with hard particulate (e.g., sand) and surfaces (e.g., rocks). Mechanical degradation works synergistically with chemical degradation processes. As chemical degradation embrittles plastic, mechanical degradation can more readily fragment the material.

#### Biotic Processes

Biotic degradation processes encompass plastic’s fragmentation, assimilation, and transformation by organisms (micro and macro). It is believed that abiotic processes can prime plastics for biotic degradation by reducing the length of the plastic’s polymer molecules and introducing labile functional groups [637,638,677,693,694]. Conversely, organisms or biological macromolecules can impede abiotic processes (e.g., biofilms screening UV light [695]). Biotic processes include chemical (enzymatic, hydrolytic, oxidative) and mechanical degradation. There is an important distinction between the *biodegradability* and the *biodegradation* of plastics. Biodegradability (sometimes referred to as *inherent biodegradability*) indicates the potential for a plastic to be degraded by some biological means [639]. ISO and ASTM standards for biodegradability can test for this intrinsic property [696]. In contrast, biodegradation is a system property describing microbial transformation of the plastic, the rate of which is dependent on the surrounding environmental conditions [639]. Thus, biodegradability can be thought of as a prerequisite for biodegradation to occur, but it says nothing about whether it occurs in a given environment, or the rate of transformation, which are the subject of biodegradation.

##### Biodegradation (Enzymatic, Hydrolytic, Oxidative)

Microbes are the organisms primarily responsible for the biodegradation of plastics in the marine environment. Microbes can degrade recalcitrant materials (e.g., lignin and oil); however, the rates of degradation are highly variable [697]. Microbes can degrade plastics hydrolytically, oxidatively, and enzymatically [637,638,640,698,699,700]. Together these processes lead to chain scission, oxidation, and new end groups [637,638]. Like their abiotic counterparts, biodegradation processes depend on temperature and pH (environmental dependencies) and on polymer type and crystallinity (material dependencies). However, biodegradation has additional requirements such as nutrient levels and the extent to which other, more labile carbon sources are available (environmental dependencies), the molecular weight of the plastic and size of the plastic particle (material dependencies), and whether microbes in the local community can degrade the given plastic (biological dependency) [640,698,699]. Degradation can occur extracellularly (e.g., through the secretion of enzymes) [640,698,699], or, if polymer chains or plastic particles are small enough to cross cellular membranes, they can be degraded intracellularly by cellular machinery and intracellular conditions [640,698,699].

In general, biodegradation requires chemical bonds and microstructural features that are cleavable, modifiable, and accessible by enzymes; chemical bonds that are hydrolytically cleavable; or chemical bonds that are susceptible to reactive oxygen species [637,638,640,698,699,700]. A recent metagenomic study examined microbial plastic-degrading enzymes in global soil and marine environments (67 locations at 3 depths across 8 oceans) [701]. Filtered environmental-hits were compared to those in the gut microbiome where plastic degrading species have not been reported. Overall, >30,000 enzyme-hits were detected. Approximately one quarter of organisms in the environmental microbiomes examined had a range of polymer-degrading enzyme-hits including enzymes capable of degrading PET, PVA, PUR, PET, and PE; phthalate-degrading enzyme-hits were also identified. Higher enzyme-hits were observed in more heavily plastic-polluted ocean locations where stratification with depth was also observed, reflecting depth-related variations in taxonomic richness. Proportions of enzyme-hits varied with location, with PUR-degrading enzymes being found in the ocean and not the soil, and twice as much PET-degrading enzymes in the soil as in the ocean [701].

Biodegradation results in many of the same physical and chemical changes as abiotic processes: reduction in molecular weight, new functional groups, increase in crystallinity, reduction in mechanical properties, increased brittleness, increased surface roughness, and increased hydrophilicity. Along with changes in the plastic, microbes may degrade organic additives, metabolize water-soluble products, and respire plastic carbon to CO_2_ [640,698,699]. It is important to emphasize that although microbial biodegradation of plastic in the ocean can occur, the rates are very low [23,639,640,641,701].

##### Mechanical Fragmentation by Marine Biota

Biological fragmentation of plastic results from the biting or chewing of plastics by organisms and the excretion or regurgitation of smaller secondary particles and fragments. This process has been observed for MPs ingested and excreted by a variety of organisms, including amphipods, arctic krill, rotifers, and seabirds [568,702,703,704].

#### Lifetimes

Plastic products entering the marine environment are heterogeneous, variable, and diverse in their properties. For instance, a bottle made of PET and another made of PP may functionally both serve the same purpose, but they will have different fates in the environment. Likewise, a clear PS cup and a Styrofoam (expanded PS) cup, though both are cups and chemically PS, can differ in molecular and microstructural properties and surface area to volume ratio resulting in different fates. From the descriptions above, it is evident that different plastic degradation processes can lead to similar transformation outcomes. Differences in environmental persistence and fate arise because each plastic product has different susceptibilities to any given degradation process, based on the product’s geometry, the local environment, and the type of plastic. Collectively, degradation processes create heterogeneity, variability, and diversity in the properties of the plastic and its degradation products. This translates into a continuum of lifetimes for a given plastic product, not a single number as often quoted by infographics [240,471]. Similarly, this heterogeneity can lead to the misrepresentation of plastic lifetimes without appropriate reporting of the material and environmental conditions. For instance, this is evidenced by the discrepancy between the lifetimes of PLA products in composting and marine conditions; under composting conditions, PLA readily degrades in months, while under marine conditions, it degrades over many years [689,705,706].

**Figure 3.2:**
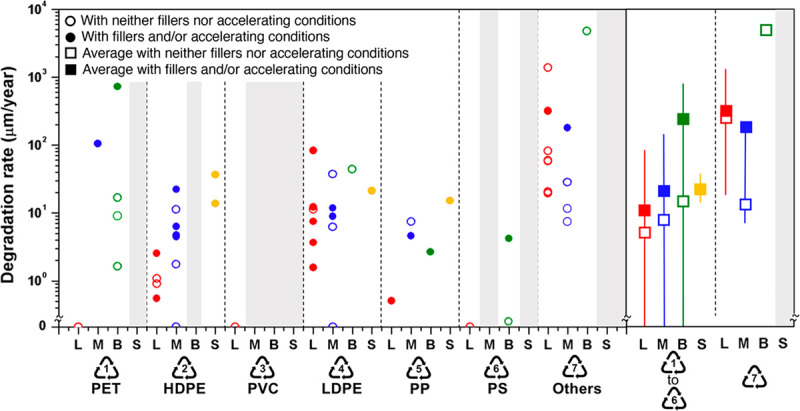
**Degradation rates for various plastics.** “Vertical columns represent different environmental conditions (L, landfill/compost/soil; M, marine; B, biological; S, sunlight) and plastic types (represented by their resin identification codes). Plastics type 7, “others”, corresponds to various nominally biodegradable plastics. The range and average value for plastic types 1–6 are shown on the right as lines and squares, respectively, as well as for biodegradable “others”. Data points representing degradation rates that were unmeasurably slow are shown on the x-axis. Gray columns represent combinations for which no data were found.” PET, polyethylene terephthalate; HDPE, high-density polyethylene; PVC, polyvinyl chloride; LDPE, low-density polyethylene; PP, polypropylene; PS, polystyrene. Figure caption and figure reprinted with permission from (Chamas *et al.*, 2020) [240] (CC BY 4.0).

The coupling of geometry, local environment, and plastic type makes determining plastics’ lifetimes in the environment challenging and onerous. Chamas et al. [240] proposed the “specific surface degradation rate” (µm/year) as a measure of plastic degradation (Figure 3.2). This term is a material-environment coupled property, which was calculated for the major plastics (PE, PET, PVC, PP, PS) and some biodegradable plastics (PLA, polyhydroxyalkanoate). The specific surface degradation rate can range from 0 to 1,400 µm/year depending on the combination of plastic type and environment [240]. Applying this parameter to real products, it was estimated that a PET single-use bottle and HDPE bottle in the marine environment with accelerants (e.g., sunlight, heat, microbes, additives) could have average half-lives of 2.3 years and 26 years, respectively [240]. The surface specific degradation rate only considers gross mass loss from a product but not the fate of the degradation products, which can include water-soluble products and MNPs.

Determining rates and lifetimes of plastic degradation in the marine environment remains an active area of research frustrated by the tremendous complexity and diversity of plastic products and marine environmental conditions, as well as the relatively easy transport of lightweight plastics by wind and water. Despite having a relatively well-developed theoretical understanding of plastic degradation mechanisms, our grasp of how those mechanisms manifest together as degradation rates and lifetimes in the marine environment remains nascent. Much more would be required to fully understand the long-term fate of plastics in the ocean; it is clear, however, that rates of input to the environment considerably exceed rates of degradation, leading to environmental accumulation [639].

### Plastics in Aquatic Food Webs and Seafood

#### Plastics in Marine Biota

Plastic-biota interactions in aquatic environments include entanglement, colonization, and ingestion. Entanglement in large plastic litter such as ALDFG is well known to impact megafauna such as marine mammals, turtles, seabirds, and some fishes; such interactions are usually lethal [505,567,722].

Ingestion of plastics is less visible but is widespread, and thus may be a more insidious threat to marine life. Plastic pieces and particles have been found in hundreds of marine and freshwater species from diverse environments around the world. The aquatic animal species ingesting plastic include a variety of invertebrates, fish, seabirds, turtles, and marine mammals [2,567,568,608,723,724,725,726,727,728,729,730,731,732,733]. Santos et al. [734] documented 1,565 species globally for which ingestion of plastics had been reported; most of these (82%) were marine species, which may, at least in part, reflect the greater effort devoted to investigating plastics in marine systems as opposed to other environments.

The probability and extent of ingestion of plastic particles is influenced by multiple factors [725,729], including encounter frequency (related to particle concentrations), the size of the particles relative to the normal food types [735,736,737], shape, color [523,738], and the presence of eco-coronas (layers of biological macromolecules adsorbed to the surface of MPs [507,739] or infochemicals (biochemicals that mediate communication among organisms) from biofilms [740,741,742,743,744]. Ingestion may be indiscriminate, but some suspension feeders are able to reject plastics in favor of their natural food [735,745,746,747].

Our current understanding regarding the ingestion of plastic particles by marine species has some limitations. One is that much of the data are for MPs that are in the GI tract, which does not represent internal concentrations in tissues [727,748]. Moreover, the MPs may often be excreted without adversely affecting the animal. The location of MPs mainly in GI tract has implications for the behavior of plastics in aquatic food webs (see below).

Another limitation is that most studies of plastics in biota in the field have measured only the larger sized MPs (>150 µm) [748]. Much less is known about the ingestion of NPs and small MPs [749,750], which includes the sizes most likely to undergo translocation from the GI tract into tissues [751]. The dearth of information about the ingestion of these smaller plastic particles by marine species is a major knowledge gap.

#### Plastics as Substrate: The Plastisphere

Marine microbes (bacteria, archaea, and single-celled eukaryotes such as diatoms and dinoflagellates) form the base of the marine food web, and so their interactions with plastic particles may help drive the trophic dynamics of MPs. Any hard surface immersed in an aquatic environment will readily acquire a biofilm—an “eco-corona”. Initial formation of an “eco-corona” [507,739] composed of biological macromolecules is driven by physico-chemical interactions with the surrounding water, and colonization by microorganisms typically follows within hours [612]. Over longer periods in freshwater and marine environments, succession to an assemblage of epifaunal microorganisms then follows over the following weeks to months. Plastics are no exception and readily become colonized by micro and macro biota. Microbial communities on plastic particles in the ocean have been named the *Plastisphere* [752]. A variety of microbial taxa have been found associated with plastics, including bacteria, archaea, diatoms, dinoflagellates, and fungi (reviewed [641,753]). These taxa include pathogens (e.g., certain *Vibrio* species) and species of diatoms, dinoflagellates, and cyanobacteria that are associated with harmful algal blooms [605,641,752,753,754]. The colonized MPs may thus serve as vectors to transport these organisms, as well as the genes they carry (e.g., antibiotic resistance genes [755]), over long distances, with potential impacts on humans and aquaculture.

In some instances, this “fouling” assemblage can overwhelm originally buoyant plastic items causing them to sink [610,612,756]. In addition, infochemicals such as dimethyl sulfide released from the microbial assemblage can attract planktivorous organisms and seabirds that normally use these chemical cues to indicate the location of food [740,741,742,743,744,757]. The presence of an eco-corona or microbial biofilm may promote consumption and internalization of plastic particles into cells [758].

#### Bioaccumulation, Trophic Transfer, and Biomagnification of Plastic Particles

The behavior of chemicals in marine food webs is governed in part by processes such as *bioaccumulation* (increased concentration of chemical during the lifespan of an organism), *trophic transfer* (movement of the chemical from one trophic level to another), and *biomagnification* (increased concentration with increasing trophic level). Plastic particles may potentially undergo the same processes (reviewed [748]), but there is some confusion and misunderstanding in the MP literature about these processes and the extent to which they occur for MPs and NPs [727,748,759]. It is important to distinguish between plastic particles and the chemicals (additives, sorbed pollutants) that they may carry. Many of the plastic-associated chemicals also have sources other than plastics and are well known to bioaccumulate and biomagnify in marine food webs. We focus here on the plastic particles themselves, for which these processes are not as well understood.

**Figure 3.3:**
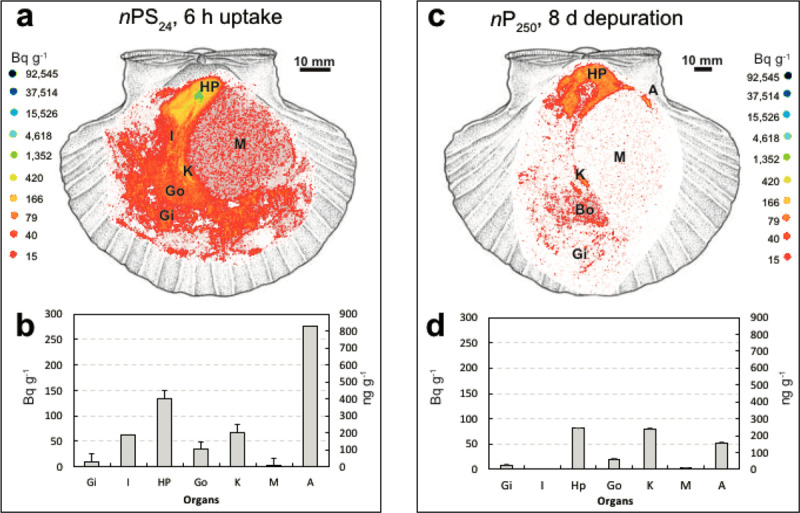
**Uptake and depuration of 24 nm polystyrene nanoparticles. (a)** Tissue distributions shown by Quantitative Whole Body Autoradiography (QWBA) in *Pecten maximus* after 6 h uptake with **(b)** quantification of radioactivity levels measured in tissues (left axis; Bq g^–1^, S/Nnorm; right axis ng g^–1^), **(c)** Tissue distributions shown by QWBA in *Pecten maximus* after 8 days of depuration, with **(d)** quantification after 8 days of depuration (left axis Bq g^–1^, S/Nnorm; right axis, ng g^–1^). Each bar represents the mean value measured in 3–6 different sections of a given individual. No bar = radioactivity < LOD. nPS_24_ and nP_250_ are spherical polystyrene nanoparticles with sizes of approximately 24 ± 13 and 248 ± 21 nm respectively. HP, Hepatopancreas; Gi, Gills; Go, Gonad; I, Intestine; K, Kidney; M, Muscle; A, Anus. Figure caption and figure reprinted from (Al-Sid-Cheikh *et al.*, 2018) [764] (CC BY 4.0).

Bioaccumulation of MNPs has been documented in some, but not all, laboratory and field studies involving aquatic species [748]. Bioaccumulation typically requires translocation from the GI tract into tissues. Smaller MNPs (particles <150 µm) have a greater potential for translocation [751]. There is evidence from field studies that ingested MPs can translocate into tissues [760,761,762], but in some cases the sizes of particles detected are larger than those seen to translocate experimentally or that can be explained by known mechanisms of transcellular and paracellular uptake [762]. Such conflicting results between laboratory and field studies (e.g., [762,763,764,765,766]) indicate that this is an area in need of further research. Although there is some evidence suggesting that NPs may persist in tissues, depuration of MPs and NPs often occurs within a few days [745,746,764,767,768]. For example, a study using radiolabeled NPs found size-dependent, rapid uptake into scallop tissues, followed by rapid depuration (Figure 3.3) [764]. A recent study suggested that less than 1% of NPs move from a fish GI tract to other tissues, and the accumulation there did not persist [769,770]. In general, the persistence of MPs and NPs in tissues—which is required for bioaccumulation and biomagnification to occur—is not yet fully understood. Insight into these processes may come from cross-disciplinary exchange with biomaterial scientists who study polymeric wear particles from orthopedic implants or nanoparticles for drug delivery. The migration and clearance of polymeric particles from orthopedic implants to other tissues in humans have been described [771,772,773,774,775,776].

There is abundant evidence for trophic transfer of plastic particles from prey to consumer [748]. However, trophic transfer does not necessarily lead to bioaccumulation or biomagnification in the consumer, at least for MPs, because much of the MP load is retained in the GI tract and rapidly excreted. In contrast to many POPs, for which biomagnification is well known, both experimental and field-based studies have indicated that MPs do not biomagnify [729,748,759,762,777,778]. In some cases, there is evidence of trophic *dilution*—a reduction in concentrations of MPs in higher trophic levels [760,778,779]. An important caveat is that because we do not yet have reliable methods for measuring NPs in animals, it remains possible that their behavior could be more similar to that of POPs.

#### Plastics in Seafood

As noted above, a wide range of organisms are known to ingest MPs; this includes a variety of seafood species, including fish, shellfish (mollusks and crustaceans), seaweeds, echinoderms, and cephalopods. Examination of seafood species prior to and at the point sale clearly indicates the potential for human exposure to MPs through consumption of MP-containing seafood [780,781,782]. The discussion that follows has been informed by several recent reviews that have compiled data on concentrations of MPs in seafood and provided estimates of human exposure [762,783,784,785,786,787,788,789,790].

MP particles are typically found at greatest concentration in the GI tract and with most species this is removed before consumption; exceptions include smaller fish (e.g., anchovies, sardines) and most bivalves, which are usually eaten whole. Several studies have reported higher MP concentrations in inedible vs. edible fractions of seafood [791,792,793]. However, the “inedible” fractions may be further processed for use as animal feed (including fish meal), providing additional opportunities for transfer to humans later in the food supply chain [790,794]. Studies examining the edible fraction of fish have often found MPs in the species examined [785,795,796,797,798,799]. The environment may also influence the MP burden in animals; e.g., benthic species have been found to contain more MP than pelagic species [796,800].

Most current knowledge about plastics in seafood is for the larger-sized MPs. For the smaller NPs the potential for transfer to humans is greater because of the greater potential of these particles to translocate from the GI tract to the circulatory system and onward to tissues throughout the organism, as noted earlier.

Despite the prominence of seafood in discussions of human exposure to MPs and NPs, there is need for caution in the interpretation of these findings, for several reasons. For MPs, the quantities found are typically quite low (1 or 2 pieces per individual fish or shellfish) [801]. In the context of total human exposure to MPs, seafood is only one of several sources, others of which (e.g., drinking water, inhaled air) may equal or exceed the exposure from seafood (Table 3.2) [783,788,802]. Moreover, in addition to the MPs in the tissues of seafood species themselves, there are several points in the pathway from harvest to consumption at which MPs may be introduced [790]. For example, MPs are produced through the use of cutting boards and cookware [803,804,805] and the quantity of MPs settling onto the food from the atmosphere during preparation and consumption is likely to exceed that in the seafood beingconsumed [802].

**Table 3.2:** **Estimates of microplastics consumption (annual particle intake per capita) from seafood versus other sources.** Numbers represent average or ranges of annual intake (particle numbers) per capita. For additional discussion of human exposure, see (Ramsperger *et al.*, 2022) [1198] and (Lusher and Covernton, 2022) [790].


	(DANOPOULOS *ET AL.*, 2020) [786]	(CATARINO *ET AL.*, 2018) [802]	(VAN CAUWENBERGHE AND JANSSEN, 2014) [767]	(HANTORO *ET AL.*, 2019) [784]	(BARBOZA *ET AL.*, 2020) [785]	(COX *ET AL.*, 2019) [783]	(DOMENECH AND MARCOS, 2021) [788]	(ZHANG *ET AL.*, 2020) [1562]

mollusks	2,067(0–27,825)	123–4,620	1,800–11,000	500–32,750				

Crustaceans	206–17,716			322–19,511				

Shellfish								0–1.3 × 10^4^

Fish	31–8,323			25–32,375	112–3,078			

Total seafood						17,448	22,000	

Fruits & vegetables							19 × 10^9^	

Bottled water						15,156	2.61 × 10^3^–3.96 × 10^10^	

Tap water						3,358		0–4.7 × 10^3^

Total water								0–2.8 × 10^10^

Salt						86	261	0–7.3 × 10^4^

Alcohol						294	26	

Honey						73		

Sugar						8,319		

Dust ingestion								100–1.9 × 10^4^

Air (inhaled)						46,501	2,160	

Indoor air								1.9 × 10^3^–1 × 10^5^

Outdoor air								0–3 × 10^7^


Overall, the evidence available does not implicate consumption of seafood as a major pathway for transfer of MP from the environment to humans. Two caveats are 1) the potential contribution from seafood may be greater for populations for whom seafood is a higher proportion of their diet and 2) without corrective action (Section 7), the relative contribution from seafood could increase in the future. The relative importance of seafood as a vector for transfer of NPs is less clear because particles of this size are extremely difficult to quantify in environmental samples. Filling the NP knowledge void should be a researchpriority.

### Plastic as a Vector of Chemicals

Plastics in marine environments may contain, or have accumulated, hundreds of chemicals classified as “additives” or “adsorbed” chemicals, respectively (see also Section 2) [8,43,472,806,807]. Thus, plastic litter in marine environments is a cocktail containing chemicals added during manufacture as well as those adsorbed from polluted water, including phthalates, PBDEs, BPA, PCBs, styrenes, PAHs, and metal(loid)s such as lead and nickel [808]. Some of these chemicals can be bioaccumulated upon their ingestion or uptake by marine organisms. The mechanisms and characteristics of MP-mediated bioaccumulation are different between adsorbed chemicals and additives.

A variety of anthropogenic chemicals have been found in association with plastics in the marine environment (Table 3.3). The importance of MPs as vectors of adsorbed chemicals to marine biota has been reviewed in several papers [21,509,809,810,811,812,813]. Mainly through equilibrium model calculations, the results suggest that the role of MPs as vectors of adsorbed chemicals is often minor compared to the accumulation of those chemicals from natural prey, especially in aquatic environments already highly contaminated by chemicals such as PCBs.

**Table 3.3:** **List of anthropogenic organic chemicals found adsorbed to plastic pellets.** *Representative references only. ** Higher brominated diphenyl ethers such as PBDE-209 excluded, because of possible contribution of additives compounded to pellets.*** “Drins” such as aldrin, dieldrin included.**** Potential contribution of additives compounded to pellets, especially sporadically high concentrations.


NAME OF CHEMICAL(S)	ABBREVIATIONS (WHERE APPLICABLE)	REFERENCES*

Polychlorinated biphenyls	PCBs	(Ogata *et al.*, 2009 [1316]; Taniguchi *et al.*, 2016 [1563]; Camacho *et al.*, 2019 [1564]; Yamashita *et al.*, 2019 [819]; Arias *et al.*, 2023 [1565])

Polybrominated diphenyl ethers**	PBDEs	(Taniguchi *et al.*, 2016 [1563]; Camacho *et al.*, 2019 [1564]; Pozo *et al.*, 2020 [1566]; Ohgaki *et al.*, 2021 [1567])

Polycyclic aromatic hydrocarbons	PAHs	(Taniguchi *et al.*, 2016 [1563]; Yeo *et al.*, 2017 [1568]; Camacho *et al.*, 2019 [1564]; Arias *et al.*, 2023 [1565])

Dichlorodipheyltrichloroethane and its metabolites	DDTs	(Ogata *et al.*, 2009 [1316]; Taniguchi *et al.*, 2016 [1563]; Camacho *et al.*, 2019 [1564]; Pozo *et al.*, 2020 [1566]; Arias *et al.*, 2023 [1565])

Hexachlorocyclohexanes	HCHs	(Ogata *et al.*, 2009 [1316]; Taniguchi *et al.*, 2016 [1563]; Camacho *et al.*, 2019 [1564]; Pozo *et al.*, 2020 [1566]; Arias *et al.*, 2023 [1565])

Cyclodiene pesticides***		(Taniguchi *et al.*, 2016 [1563]; Camacho *et al.*, 2019 [1564])

Chlordanes		(Taniguchi *et al.*, 2016 [1563])

Mirex		(Taniguchi *et al.*, 2016 [1563]; Camacho *et al.*, 2019 [1564])

Hexachlorobenzene	HCB	(Taniguchi *et al.*, 2016 [1563]; Camacho *et al.*, 2019 [1564]; Pozo *et al.*, 2020 [1566])

Pentachlorobenzene	PeCB	(Pozo *et al.*, 2020 [1566])

Benzotriazole-type UV stabilizers****	BUVSs	(Camacho *et al.*, 2019 [1564]; Karlsson *et al.*, 2021 [1569])

Organophosphorus flame retardants****	OPFRs	(Camacho *et al.*, 2019 [1564])

Triclosan****		*H. Takada, personal communication*

Sterols		*H. Takada, personal communication*

Hopanes		(Alidoust *et al.*, 2021 [1570])


Conversely, in remote areas with higher abundances of plastic, the accumulation of POPs or other sorbed chemicals from that plastic could be greater than accumulation from natural prey. Plastic from contaminated areas also may carry POPs to otherwise cleaner environments. MPs of mm-size are transported offshore by Stokes drift [814] and are sometimes found in remote islands [815], while µm-size MPs are settled and deposited in bottom sediments [816] through biofouling [610] and some other biological processes. Sorption of hydrophobic organic compounds to mm-size MP particles is not only a surface process but also involves diffusion of chemicals within the polymer matrix; for this reason, sorption/desorption takes time [817]. For example, it would take more than one year for a PCB congener with a *K*_ow_ ~7 (e.g., PCB-153) to reach equilibrium [818]. This is the reason that high concentrations of PCBs are sporadically found in mm-size MP (resin pellets) on the beaches of remote islands [819]. This means that mm-size MPs can transport sorbed chemicals from anthropogenically impacted areas to remote ecosystems.

**Figure 3.4:**
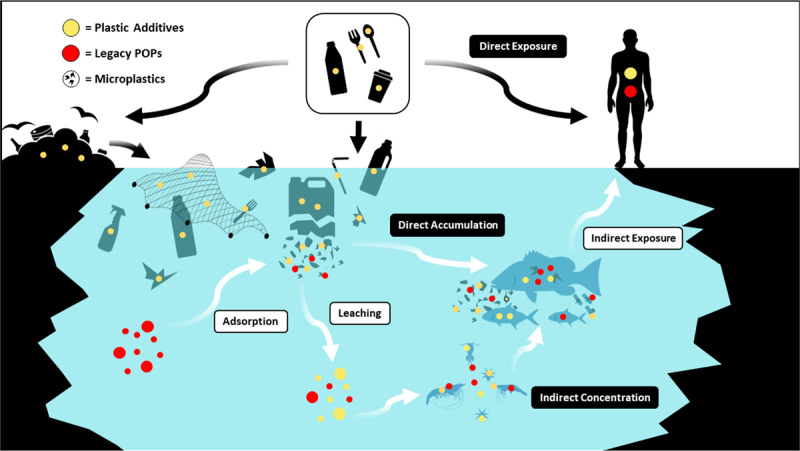
**Conceptual model of microplastic-mediated transfer of additives and POPs to marine animals and humans.** Conceptual model of microplastic-mediated transfer of additives and persistent organic pollutants (POPs) persistent to marine animals and humans. Plastic additives and legacy POPs accumulate in the ocean through leaching from waste virgin and recycled plastic; similarly, microplastics, which also contain additives, accumulate as a result of fragmentation. Plastic additives and legacy POPs can be adsorbed to microplastics. Humans can be directly exposed to plastic additives and legacy POPs from use of plastic products as well as indirectly via the food chain. Human exposure to microplastics via the food chain may also occur. *Credit*: Shige Takada and Manuel Brunner (co-authors).

The other group of chemicals found in plastics in marine environments are the additives, by-products of plastic production, and unreacted and polymer-degradation-derived monomers that are originally contained in plastic products [8]. Additives include plasticizers, antioxidants, UV stabilizers, flame retardants, and many others [8]. Many of these are EDCs and neurotoxicants [221]. (See also Section 4.) They have a wide range of hydrophobicity. Hydrophilic additives such as BPA can be directly transferred to humans (“*Direct Exposure*” in Figure 3.4) or leached into seawater. However, most of the additives are hydrophobic and they are retained in plastics and MPs in seawater [820].

Plastic ingestion can be considered as a kind of internal exposure to additives (“*Direct Accumulation*” in Figure 3.4). A key question is the extent to which the additives from ingested plastics transfer to the tissue of marine organisms. Because additives are compounded in the polymer matrix, they may not readily leach out of plastics. Hydrophobic additives are retained in MPs with minimal leaching into seawater [820]. When they are ingested by marine organisms, however, oily components in digestive fluid can facilitate the leaching of the hydrophobic additives [821,822]. Furthermore, as plastics become smaller through fragmentation, leaching of hydrophobic additives from plastics can be facilitated [823,824]. Laboratory experiments [820] and a semi-field exposure experiment [825] demonstrated that plastic additives can be transferred from ingested plastics to the tissue of organisms. Occurrence of the process was demonstrated through the detection of BUVSs and BFRs in globally collected seabird preen gland oil samples [826]. Furthermore, the bioaccumulation of plastic additives in the tissue of marine organisms and their connection to plastic ingestion has been demonstrated for whales [827], oysters [828], seabirds [829], and lanternfish [830]. Collectively, these observations demonstrate that plastics can be an important vector of additives to some marine organisms.

One impediment to interpreting these studies is the sporadic occurrence of specific additives (e.g., UV stabilizers and BFRs) in plastics [831,832] and marine organisms [821,829,833]. To connect detection of specific additives to ingestion of plastics, many observations are therefore necessary. Another difficulty is distinguishing plastic-mediated exposure to additives from prey-mediated exposure, as prey may already be contaminated with additives via indirect bioconcentration. In such scenarios (“*Indirect concentration*” in Figure 3.4), additives may leach out into seawater and organisms may concentrate the leached additives from seawater. The importance of this process has been demonstrated by exposure experiments involving moderately hydrophobic additives such as hexabromocyclodecanes (log *K*_ow_ ~ 6) [834] and BUVSs (log *K*_ow_ ~ 7) [820]. Bioaccumulation of moderately hydrophobic additives such as nonylphenol and BUVSs has been observed in a wide range of coastal organisms [835,836,837,838].

Highly hydrophobic additives such as decabrominated diphenyl ether (BDE-209, log *K*_ow_ ~ 12) may be directly accumulated from ingested plastics into tissues of organisms (*“Direct accumulation”* in Figure 3.4). However, excretion of MPs would reduce retention time and thus limit the release of such additives in the digestive tract. Consistent with this, in one study the proportion of bioaccumulated BDE209 was only a few percent of the total BDE-209 in the ingested plastics [825]. However, excreted MPs could possibly be re-ingested, until ultimately becoming unavailable through burial or other processes.

Various plastic additives have been detected in human adipose [839], blood [840], and urine [841,842,843,844]. This may occur via direct transfer through daily-use plastics, i.e., leaching of additives from food and beverage containers (“*Direct exposure*” in Figure 3.4) as well as through indirect exposure via consumption of seafood (Figure 3.4).

Overall, there are several processes through which aquatic organisms and humans may be exposed to plastic-associated chemicals. A more quantitative assessment of these processes will provide a better understanding of their relative roles in mediating the transfer of these chemicals from plastics to marine organisms and humans.

### Impacts on Marine Life

There are now thousands of publications on the impacts of large and small plastic particles on marine organisms. As with all other aspects of plastic pollution, impacts on marine animal health depend on the size, shape, and chemistry of the particles [41]. The most dramatic and visible effects occur with macroplastics; for large items of debris, the evidence for impacts is extensive and well documented [505]. These involve findings of entanglement by plastics and plastics in stomach contents of birds and large animals, often reported in the media. There is more evidence of negative effects from entanglement than from ingestion. This likely reflects the direct visibility of the damage associated with entanglement by macroplastics, which are external to the organism, as opposed to effects of ingestion, which require necropsy [505]. Kuhn and van Franeker [728] reviewed more than 700 studies of entanglement and ingestion in marine megafauna, seabirds, marine mammals, turtles, fish, and invertebrates, finding 914 species affected. The frequency and abundance of the visible ingested particles varied among taxa and was greater in some than in others; however, Wilcox et al. [724] modelled the data records and estimate that by 2050, 99% of seabird species will be affected by plastics.

There is a substantial body of evidence indicating effects of plastics on marine and aquatic organisms. The majority of the observations come from laboratory experiments, but there also is evidence of harm to organisms in the natural environment. Negative effects of all sizes of plastics have been demonstrated at most levels of biological organization from the scale of macromolecules to cells, tissues, organs, organisms, and assemblages of multiple species [505,506,507,510,845]. Although effects are not always found, in a recent meta-analysis of 139 lab and field studies, Bucci et al. [41] found that 59% of tested effects at all levels of biological organization were detected. Whether effects occur at a population level is uncertain, in part because it is extremely difficult to isolate the effects of plastics, compared to the myriad other stressors in the environment that may have population-level impacts. Nevertheless, there are studies that predict the potential for impacts at the population level, especially for species that are already threatened or endangered [846].

#### Particle Effects and Mechanisms of Micro and Nanoplastics

In contrast to the effects of macroplastics, the effects of MPs and NPs are less obvious, and the mechanisms of action are not yet well understood. There is a rapidly growing number of papers on the effects attributed to MP or NP particles themselves observed in experimental exposures, although a limitation is that many of the experimental studies used concentrations of plastics greater than those reported to occur in the environment [41,847,848,849]. Reports on impacts address diverse taxa, marine mammals to fish, crustaceans, and mollusks. Jacob et al. [850] reviewed studies on fish; Pisani et al. [851] examined studies on crustaceans; and Han et al. [852] did a meta-analysis of endpoints in aquatic vertebrates and invertebrates. The reported impacts include effects on survival, behavior, metabolism, and reproduction. Kogel et al. [514] reviewed effects observed in experimental studies with diverse taxa, considering multiple aspects of the particles as used, and noted that not all experiments are comparable. The reader is referred to these reviews for further details on the impacts. The text below considers types of health effects of the particles, and some of the mechanisms involved. Effects of associated chemicals are considered in the following subsection.

Given the broad molecular similarity across the biological spectrum, fundamental mechanisms inferred from experiments and observations with one species or with cells in culture are likely to apply to other species. Thus, while the focus of this section is on effects on marine species, the mechanisms considered here are drawn from studies in mammals and human cells as well as from marine or aquatic species. Some are discussed in more detail in the section on human health effects (Section 4).

MP and NP particle effects on cells, tissues and organisms depend to a large degree on the particle size, shape, and chemistry, as well as charge and concentration [41,504]. There still are uncertainties regarding the size ranges of particles classified as “microplastics” or “nanoplastics,” although there are suggestions for standardizing the terminology [521,737]. Commonly accepted sizes in the maximum dimension for MP particles are 1 µm to 5 mm, and for NPs as 1 to 100 nm or 1,000 nm [521]. Different size particles may differ in the *physical* effects related to the shape and structure of the particle, and how this can affect cells and cellular function.

There have been several reviews of NP effects *in vitro*, in diverse human cells in culture, and *in vivo*, in diverse model organisms [518,527,528,529,750,853,854,855]. Among commonly observed effects is enhanced production of reactive oxygen species (ROS) and an oxidative stress response, which can impair cellular and organ function in marine and other species [518,856]. Disruption of mitochondrial function and a role in ROS generation are seen in various human and other species cells in culture [857,858], The exact mechanisms of action on the mitochondria are not understood, but particles other than plastics are known to affect mitochondria. Mechanical stretching of the membranes could be involved in the effects on cells and on mitochondria [859].

Inflammation, suggesting an immune system reaction to the particles, has been observed in cells and organisms [860]. Consistent with this, changes in activation of genes involved in inflammation, including p38, mitogen-activated protein kinases, and various cytokines, have been observed [861]. Broader consequences of inflammation and oxidative stress can include GI, hepatic, reproductive, and neurotoxic effects, observed in rodents and in zebrafish [862,863,864]. In addition, inflammatory and other responses in gut and in liver and other tissues have been reported in invertebrates and fish [864,865]. The liver increasingly appears to be a target [866,867]. Metabolic effects suggest that NP may contribute to obesogenesis, possibly due to action of associated chemicals [868], including chemicals yet to be identified [869].

MPs in the GI tract may impact animal health in several ways, for example through intestinal blockage, triggering satiety in the absence of nutritional value (a maladaptive food choice referred to as an “evolutionary trap” [734]), or by interfering with the function of the animal’s microbiome [870,871]. The review by Jacob [850] and several primary studies have found that MPs and NPs cause alteration of gut microbiomes in fish [872], including adults and larval zebrafish [873,874], which could affect the function of the gut-brain axis. Evidence of effects on microbiomes is growing.

A difference between MPs and NPs related to particle properties is that the larger MPs reportedly either pass through the digestive tract and are excreted or accumulate therein, causing obstruction and potentially a false sense of satiety. NPs and smaller MPs apparently can cross cellular membranes in the gut [770]. NP also have been found to cross cell membranes of cells in culture [875]. Particles transferring from the gut to the blood may then distribute throughout the body, whether of vertebrate or invertebrate [763]. However, there is not yet a clear understanding regarding which particle sizes are more or less likely to pass from the gut into the circulatory system. In studies in marine and aquatic species smaller NPs were found to appear in or affect internal organs. An example in zebrafish showed that 50 nm particles appeared in blood and crossed the blood brain barrier and affected dopamine metabolism, while 100 nm particles did not [876]. It is also possible that some effects could result from the disruption of membranes and membrane function by particles [877]. Thus, the MP and NP particles themselves may elicit effects that compromise growth, behavior, or reproduction and other processes. Moreover, there are suggestions that transgenerational effects of NPs may occur [878,879]. Notably, the majority of the studies that address MP impacts experimentally have used virgin plastics at concentrations or doses far in excess of the concentrations in the ocean [41,847]. If bioaccumulation of the NP is proven, it could signal that a latency between exposure and effect may occur, as particles and their associated chemicals accumulate in the brain, for example, over time.

#### Effects and Mechanisms of Plastic-Associated Chemicals

*Chemical effects* result from the chemical composition of the particle that may include plasticizers or others added in synthesis, or chemicals associated with the particle, adsorbed from the environment. In nature, the effects of plastics will involve both particle and chemical effects. The studies showing effects of MPs or NPs on aquatic species in the laboratory seldom have examined chemical effects at the same time. As discussed above, plastics have been referred to as a “Trojan horse,” carrying the various chemicals associated with plastics, both additives and adsorbed chemicals, into organisms that acquire plastics via dietary or respiratory pathways, whether through gills or lungs [880,881,882]. Smaller particles that cross membranes may carry chemicals throughout the body. Larger particles that are ingested but do not cross membranes still may desorb or release additives in the gut, which then can be accumulated in the body.

As discussed above, the additives include plasticizers and stabilizers, with BPA, phthalates such as DEHP, and PBDEs as prominent examples. Adsorbed chemicals (Table 3.3) generally are POPs and other highly or moderately hydrophobic chemicals including PCBs, PCDDs, PAHs, pesticides, and others. There is a huge literature on the environmental occurrence of these compounds and their effects in aquatic and mammalian model organisms, and in animals in the ocean and Great Lakes. There is also extensive epidemiological information linking exposure to effects in humans, reviewed in Landrigan et al. [21] and in the Human Health section of this report (Section 4).

The effects in animal models (zebrafish, medaka, rodents) exposed to plastic-associated chemicals include neurodevelopmental disorders such as behavioral and cognitive effects (PCBs, PCDDs), immune dysfunction (PCBs, PCDDs, PBDEs), reproductive impairment (BPA, other chemicals), lipid metabolism defects (phthalates), cardiovascular disease (PCBs, PCDDs, PAHs), carcinogenesis (PAHs, PCBs), and population effects (endocrine disruptors, dioxin equivalents). The mechanisms for many of these effects are known to involve cytosolic and nuclear receptors that regulate genes determining hormonal action, growth and reproduction, cell proliferation and others.

Critical questions concerning effects in the ocean are whether—and where—there could be exposure to MPs or NPs at levels capable of eliciting effects like those observed experimentally [509,847,883], and what proportion of chemical exposure can be attributed to plastic sources. Immune system and reproductive effects have been observed in cetaceans, associated with PCBs in the tissues [884,885,886], developmental effects linked to population level effects have been seen in salmonids in the Great Lakes [887], and reproductive effects observed in birds have been linked to PCBs and pesticides [888]. An explicit link to plastics is seldom known, however, as there are many sources for such chemicals. Nevertheless, as we note above, in the case of additives, plastics may be a major source to the ocean, and to organisms: the bioaccumulation of plastic additives has been connected with plastic ingestion in whales, seabirds, fish, and a crustacean.

Data from mesopelagic fishes are providing information regarding exposure to chemicals from plastics. Analysis of a suite of additives and PCBs in myctophid fish from the mesopelagic zone within a gyre indicated that levels of PBDEs—but not other contaminants in these fish—were associated with the prevalence of plastic particles in the water within the gyre in one study [889], but a subsequent study did not show the same result [830]. In another study, the added risk of plastics with sorbed chemicals was negligible compared to that from chemicals sorbed to natural organic matter [890]. Whether passage through the environments from rivers to the ocean inevitably confers a chemical patina that can elicit effects apart from or in addition to the particles themselves is not clear. Moreover, as noted above, plastic-associated chemicals include many that also have other sources, and the contribution of those from plastics is not quantifiable in many cases. Many mechanisms are the same, and so the health effects attributable to the chemicals should reflect the combined exposure to those chemicals from all sources and their transformation products (see Box 3.3), and how particles and chemicals together contribute to impacts. Thus, whether chemicals in the water that sorb to plastic particles present a substantial risk to fish or other consumers is not fully resolved.

**Box 3.3 Microplastics (MPs) and Toxic Chemicals From Tire-Wear.**

Road transport is a major source of MP leakage to the environment—an estimated 2.7 megatons globally in 2019. Specific sources include tire abrasion, brake wear and eroded road markings [5]. Tire wear particles, in particular, are recognized as an important source of MPs to aquatic environments [310,583,584,586,891].

Tires were originally made from natural rubber but are now made from a combination of natural rubber and synthetic polymers to which are added sulfur (1–4%) to increase elasticity, carbon black (22–40%) as a filler, oil to improve wet-grip performance, and a number of other chemicals to augment durability. During use, contact between the tire and the road causes shear and heat with the generation of MP particles [583].

The amount of tire wear-related MPs released varies between countries in relation to traffic volume. Annual estimates range from 1.25–1.80 megatons in the US, 0.76 megatons in China, 0.29 megatons in Brazil, 0.29 megatons in India, 0.24 megatons in Japan, 0.13 megatons in Germany and 0.04–0.08 megatons in the UK [583].

Tire-wear particles contribute to the particulate matter in the air, including the PM_2.5_ and PM_10_ fractions that have demonstrated human health effects [892]. Inhaled tire-wear particles caused pulmonary fibrotic injury in mice [893], and toxic effects of tire-wear products have been reported in a human lung cell line [894,895]. Multiple chemicals used in tires are toxic and can be released to the environment; these include carbon black, which is possibly carcinogenic [374], toxic metal(loid)s such as cadmium, lead, nickel, and redox-active metals such as copper and zinc, and a variety of organic compounds [583,896,897]. No published studies have directly examined the human health hazards associated with exposure to tire-wear.

In the US, unexplained deaths in Pacific Northwest Coho salmon occurred annually over decades. This acute mortality coincided with storm water runoff in high-density traffic areas as salmon were returning to their freshwater breeding grounds. It was thus termed “urban runoff mortality syndrome.” Yet the causal agent remained unidentified. Recently, a transformation product of a tire rubber additive has been identified as the chemical responsible for this syndrome [585]. N-(1,3-dimethylbutyl)-N′-phenyl-p-phenylenediamine (6PPD) is an antiozonant added to the rubber used to make tires; it is widely used and added at substantial amounts (0.4 to 2%)[585]. After leaching from tire wear particles, it can be transformed in the environment to 6PPD-quinone. The quinone is extremely toxic to Coho salmon, which are exposed to it when they seek to reproduce in urban creeks that receive stormwater containing roadway runoff [585]. 6PPD -quinone was detected in storm water and roadway runoff at concentrations ranging from 0.016–2.29 µg/L, concentrations up to 24 times the median lethal concentration for Coho salmon (0.095 µg/L)[585].

Follow-up studies have shown species-specific differences in susceptibility to “urban runoff mortality syndrome” with high vulnerability in Coho salmon, white-spotted char, rainbow trout, and brook trout, but lower vulnerability in chum, other salmon species, zebrafish, arctic char, white sturgeon, and three crustacean species [585,898,899,900,901,902].

This case provides a compelling example of how additives in plastics can be transformed in the environment to novel toxic compounds and how such toxicity may remain unknown or unexplained for years. It also highlights the need for manufacturers to fully disclose the chemicals in their plastic products.

#### Effects in the Environment

**Figure 3.5:**
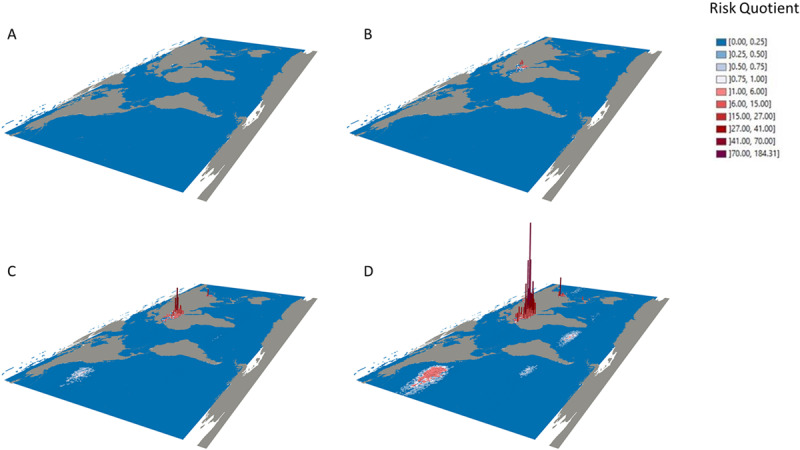
**Global risks of microplastic pollution based on worst case scenario** (unacceptable level (PNEC) = 7.99 *103 MP m^–3^) displayed in a four-panel plot, in which each panel corresponded to a specific year: 1970 **(A)**, 2010 **(B)**, 2050 **(C)**, and 2100 **(D)**. Cell specific (1° by 1°) risk estimates were calculated, and a 3D visualization of the data was generated. The risk estimates were represented in 3D as elevation values. As long as the risk quotient remains lower than the value of 1 (bluish tones), policy makers consider no risk due to MPs. In case that the risk quotient exceeds the value of 1 (reddish tones), there is a risk. Figure reproduced from (Everaert *et al.*, 2020) [904] (CC BY-NC-ND 4.0).

A recent modeling study suggests that, at present, harmful effects of MPs in the environment are likely to be restricted to locations where their abundance is relatively high and predicts that if environmental accumulation continues at the current rate, there are likely to be widespread ecological effects in the next 50–100 years (Figure 3.5) [903,904]. This future accumulation will add to what has been proposed as a global “toxicity debt”—the fragmentation of large plastics to MP and NP, which may exert greater toxicity over time due to the increase in these more toxic particles and the resulting increase in surface area and the potential release of associated chemicals [64,905].

##### Ecosystem-Level Effects

The potential for ecosystem-level effects of plastics is likely to vary among ecosystems. It has been suggested that some ecosystems may be more vulnerable [906]. While it is extremely difficult to assess changes in ecosystems such as many estuaries, coral reefs (see below), the Antarctic, and the deep ocean, the presence of plastic particles in, for example, deep sea fish [731,907,908] and in amphipods from the deepest parts of the ocean [909] indicate that ecosystem-level effects could occur globally.

Plastics may adversely impact ocean ecosystems through effects on a range of global processes and interactions affecting the natural flux of chemicals and energy in the environment [507,641]. Effects will vary across systems, e.g., comparing estuarine and open ocean systems, and the different species involved in energy transformation and transfer may respond differently to MP and NP. Factors that may influence this include polymer composition, particle size, associated chemicals, species feeding strategies, and others [910].

##### Effects on Phytoplankton and Primary Production

The effects of MP and material leached from plastic on phytoplankton have been reviewed [753]. Some studies have reported alterations in photosynthesis and primary productivity (the fixation of CO_2_ into organic molecules). Particles and especially leachates have adverse effects, although there is limited ability to generalize because of differences in species tested, polymer identity and size, and leached material. Effects more commonly seen include ROS generation and oxidative stress, and transcriptional alterations and effects on the proteome [911,912]. Both stimulation and inhibition of primary production have been observed. Some studies report increased primary productivity by organisms in the “plastisphere,” while hetero-aggregation and sinking of particles and cells may negatively affect productivity [912]. The potential link between MP effects on phytoplankton and oceanic carbon sequestration capacity are elaborated below, in Ocean Plastics and Climate.

##### Effects on Zooplankton and Energy Transfer

Microzooplankton comprise heterotrophic and mixotrophic organisms 20–200 µm in size, which include many protists, such as ciliates, dinoflagellates, and foraminiferans, as well as small metazoans, such as copepods and their developmental stages and some meroplanktonic larvae. We focus here on copepods as a representative and broadly important taxon.

Copepods (crustaceans in the subclass Copepoda) are among the most abundant primary consumers on earth, occurring in all waters; they form the base of food chains in the ocean. While Pisani et al. [851] reviewed plastics’ effects in crustaceans, copepods deserve special attention given their profound role in energy transfer. They also exemplify the complexities in dissecting effects in the ocean. Some copepod species generate flow fields to bring food particles in toward the mouth and then select particles by size and chemoperception. Inert particles can be rejected or consumed. Studies with various plastic particles indicate that copepods do consume plastic particles. Studies with a common species indicated that plastic beads or fibers alone were usually rejected after “tasting,” regardless of particle size or polymer type [747]. However, when particles were present together with prey diatoms, they were ingested together [913]. This implies that plastic particles may not be ingested by some species in regions where there are low densities of prey. Behavioral studies have suggested that there is a low risk of MP ingestion by planktonic copepods, although some species and other zooplankton do ingest particles alone, without regard to the presence of actual prey [914].

The duration of plastic particles’ time in the ocean may alter their acceptability to copepods. Polystyrene beads kept for several weeks in seawater were ingested by two calanoid copepod species at higher rates than fresh beads alone. The suggestion is that the microbial content of biofilms developing on the particles could make them more palatable [742].

Studies of impacts on copepods have shown varying results. In a laboratory study with the copepod *Acartia tonsa* investigators found that polystyrene microbeads affected growth and survival suggesting a decrease in population size that cumulatively could be substantial [915]. Large effects of PS MP particles were also observed on growth rates of a doliolid, an important gelatinous zooplankton species [916]. Zooplankton fecal pellets are important in carbon flux in the ocean (see below). Effects of plastic on fecal pellet size and composition have been observed. Decreased fecal pellet size implies less energy transfer to depths [915], while at the same time fecal pellets can vector MPs to the depths [614,917].

##### Effects on Coral Reefs

Coral reefs are among the most important biodiversity hotspots on our planet. Corals are the keystone species of coral reefs, and scleractinian corals secrete calcium carbonate skeletons that are the basis of reef structures. These organisms have adapted to live in nutrient-poor conditions by establishing a symbiosis with a wide range of microorganisms, including dinoflagellates of the Symbiodiniaceae family. Dinoflagellates are essential to coral health as they photosynthesize and transfer most of their photosynthates to the coral host for its own needs. When corals are exposed to stress, they tend to expel their dinoflagellate symbionts (a phenomenon referred to “coral bleaching”), and thus experience starvation. If bleaching continues for too long, corals will die.

Coral reefs also are among the most threatened ecosystems in the world and are affected by a range of global (e.g., warming, acidification) and local (e.g., overfishing, pollution) stressors. The combination of stressors potentially leads to greater impacts than a single factor. Currently, pollution from plastics and plastic additives such as biocides, flame-retardants, and plasticizers is a growing concern for the health of corals and other reef organisms [918,919]. Indeed, macroplastics and MPs have been consistently found in the water, sediments and organisms of all coral reefs studied, although little is known about the level of NPs [919]. The potential effects of MPs on corals may involve ingestion and direct exposure and the combined action of MPs and associated chemical contaminants, contributing to coral disease, and impacts on coral-Symbiodiniaceae symbiosis, with significant bleaching associated with NPs [920]. Some corals may mistake the plastics spiked with microbial surface biofilms as their natural food source [921]. Once ingested, MPs can induce a false sense of satiety and reduce natural heterotrophic feeding, although this effect depends on the species [921,922,923]. In addition to active ingestion of MPs by corals, passive adhesion to the coral structure surface may also impact coral health [921,922]. Laboratory studies have demonstrated that MP exposure (including active ingestion and passive surface adhesion) can influence the coral energetics, growth, and overall health, with consequences for feeding behavior, photosynthetic performance, energy expenditure, skeletal calcification, and even tissue bleaching and necrosis.

Although corals are the main reef builders and have attracted most of the scientific attention, they are not the only reef species affected by plastics. For example, all members of the planktonic and benthic species described above are also present in reefs and are likely affected by plastics. A recent review [919] summarized current knowledge of the effects of plastics on reef organisms.

If corals bleach and die, the whole reef ecosystem, and the services it provides to the billion humans living nearby, will disappear. Studies should thus continue assessing the actual risk of MNPs at relevant in situ concentrations and in conjunction with other anthropogenic stressors such as global warming to determine potential synergies these stressors may have.

### Ocean Plastics and Climate

Plastics contribute to GHG emissions during the production of and transport of fossil fuels that are plastic feedstocks, during the processing of feedstocks in plastic production, and during degradation and incineration of plastics (see Plastic Life Cycle, Section 2) [480]. The annual volume of plastic-associated GHG emissions is estimated to have been 1.9 Gt of CO_2_ in 2019 [14].

Plastics in the ocean have further potential impacts on climate through direct and indirect effects on the biogeochemistry of the planet. Direct effects include GHG emissions from plastic particles, primarily PET, as they degrade in the ocean [485], although the contribution from this relative to other sources of GHGs is likely to be very small [924].

#### Effects on Global Carbon Flux

As mentioned above, MP particles ingested by zooplankton, including copepods and others, can be eliminated in fecal pellets, which can alter the density of the pellets and thus the sinking rate of pellets and of marine snow that may incorporate the fecal pellets containing plastic particles [925]. The possible consequences include changing the rate of carbon export to the deep sea [614], which could impact the global carbon balance. The sinking marine snow also provides food to organisms in the midwaters and the deep ocean, and plastics in fecal pellets and marine snow can deliver plastics to those consumers [917]. Knowledge and understanding of the magnitude of plastic particles in marine snow in different parts of the ocean and the impact on the carbon export are badly needed.

Biofilms on plastic particles could influence the impacts of those particles in the ocean, potentially altering the consumption by zooplankton, conveying pathogens [754], altering the degradation of the particles [695,926], and increasing O_2_ consumption in remineralization, thereby reducing O_2_ availability for other processes or for efflux into the atmosphere [927]. Biofilms on the particles may affect their buoyancy and distribution [610,756], including accumulation in the sea surface microlayer [928,929].

The sea surface microlayer of ocean waters is composed of lipids and metabolites from microbes and is a site where heat and gases are exchanged between ocean and atmosphere [930]. Modeling and experiments suggest that plastics may be enriched in this layer, which could affect microbial growth and activity in the surface layer, and potentially affect air-sea gas exchange at the surface [929], which could modify CO_2_ uptake in the ocean, affecting this major sink for global CO_2_. MPs in the sea surface microlayer also may be ejected from the surface, with repeated settling and ejection leading to a “grasshopper” effect that can result in long-range ocean transport [931], adding to the surface microlayer and impacts in distant regions.

#### Phytoplankton and O_2_ Generation

Effects on phytoplankton that generate O_2_ via photosynthesis could be another mechanism through which ocean plastic affects climatic conditions [932]. Experimental studies have resulted in some, albeit conflicting, evidence for such impacts on marine phytoplankton. In a study of polystyrene leachate, the photosynthetic activity of four microalgal species was increased [933]. In contrast, materials leached from weathered PVC plastic particles were found to decrease the abundance of photosynthetic cells and to decrease photosynthetic efficiency [934]. However, in that same study populations of some heterotrophic bacteria were stimulated. Negative effects of leachates have been seen on *Prochlorococcus*, the most abundant photosynthesizer in the ocean [935].

As in impacts on other organisms, plastic effects on phytoplankton tend to depend on size and composition of the particles [911,912,936]. There are outstanding questions regarding the identity of leachate compounds that affect photosynthetic bacteria including *Prochlorococcus* [937], and the mechanisms of action on photosynthesizers, which may involve oxidative stress as in animals [911]. Importantly, as with effects on animals, the effects of plastics and/or leachates observed experimentally are obtained with concentrations orders of magnitude greater than the levels known or estimated in the environment [912]. The extent of such effects in the environment is thus uncertain [938]. There is a need to determine the degree to which plastics are affecting photosynthesis in the global ocean. This should include studies to determine how the effects of plastics and other stressors, including those associated with a warming ocean, might act together on primary producers [480].

### Plastics in Freshwaters

The impetus for much research on plastics, and in part for this report, (is the magnitude of the plastics problem in the ocean. However, the potential for ecological impacts of plastics is equally important in freshwaters, including lakes and rivers. As these are common sources of drinking water worldwide, the occurrence of plastics especially MP and NP) in lakes and rivers also potentially poses a more immediate exposure pathway to humans than plastics in the ocean. Searching the literature reveals that studies of MPs in freshwater systems have lagged studies in the ocean. Accordingly, there is need for attention on plastics in freshwaters.

MPs are detectable in water and sediments of lakes and rivers globally [939]. The levels in more heavily contaminated freshwaters tend to be higher than those in the ocean, consistent with proximity to terrestrial sources [940]. As expected, the numbers of particles detected vary greatly, by 8 or 9 orders of magnitude, depending on location of sampling, with greater levels in waters near urban areas [941,942]. In a study in China, levels were many orders of magnitude higher in water and in sediment near a “dumping river” in a heavily populated area than in a moderately urbanized environment [943]. Several studies have noted that PE, PP, PS, and PET, are common in both water and sediments [944].

Groundwater, which is a major source of drinking water for as much as 1/3 of the world’s population, is of increasing concern for plastic contamination [945]. In soil ecosystems, groundwater supports nearly all terrestrial plants, from grasslands to forests. MPs and plastics’ chemicals in groundwater thus present potential ecological and human health risks. Plastics can enter groundwater from farm operations, septic discharge, waste disposal sites, road run-off (tire particles), precipitation, and tidal inflow in coastal areas, and concentrations appear to exceed those in marine waters [946]. Reviews have shown multiple types of plastic and plastic particles in groundwater [946], although in many areas, fibers are most prevalent [947]. We might also note that chemicals associated with hydrocarbon extraction, some directed to plastic feedstock, can enter groundwaters [948]. There are suggestions that better analyses are needed [512] to address the many groundwater and plastics research needs [949].

Environmental and experimental studies of plastics in freshwater biota have concentrated mostly on fish, although studies have addressed taxa from microbes and algae to birds [950]. The studies of plastics in wild freshwater fish have examined samples from Europe, Asia, and North and South America. A full appreciation of the levels and extent of contamination is hindered by the small sample sizes in many studies [951]. Most of the studies have examined gut contents for plastics [952], which reveals little about the possibility of transfer to human consumers who eat the flesh. The presence of plastics in the gut also does not convey information about possible effects on the organisms. However, McIlwraith et al. [762] were able to detect the presence of a considerable number of particles in flesh and liver of a large proportion of the fish they surveyed. This indicates that some particles do translocate from the gut to the edible portions of freshwater fish and thereby could be transferred to humans.

Much of what has been reported above for effects in marine species can be expected to apply as well to freshwater fish. Indeed, in reviewing papers on effects of virgin MPs and NPs on fish, many of those studies included freshwater fish, model species as well as wild species [850]. The studies addressed types, shapes, exposure pathways and examined various life stages. As with marine species, however, most studies on effects in biota have been conducted with MP particle concentrations far greater than the concentrations measured in the environment, as much as 5–6 orders of magnitude higher [953,954].

The presence of plastics in drinking water from lakes and rivers has attracted growing concern. The key questions have to do with the levels and nature of plastics in specific drinking water sources, whether water treatment removes particles, or whether treatment adds particles. The levels detected in drinking water range by orders of magnitude (reviewed [512]). An investigation of type, quantity, size, and shape of MPs in drinking waters found that MP levels were low in water from a “high-performance” water treatment plant [955]. Treatment plants differ in performance, yet it appears that MP concentrations generally are lower in treated than in raw waters [956].

Detection of MP and NP contamination of freshwater biota shows similar results and raises similar questions as those in marine systems. That is, there is need for improved technology, and consistency in sampling and measurement, and reporting of the nature of particles.

### Conclusions, Recommendations, and Potential Implications for the Ocean and Human Health

Plastics are persistent contaminants that do not readily degrade in the environment. Large plastics and MPs have accumulated in all oceanic and freshwater environments, in lakes, rivers, polar ocean, and the deepest ocean trenches around the world. The impacts of large plastics entangling marine animals are visible, obvious, and disturbing. The impacts of MPs are less easily observed, but studies suggest that smaller MPs can adversely disrupt the physiology of plants, animals, and microbes. NP particles are less well studied in the environment, due to the inability to accurately measure them, but experimentally they have been shown to distribute in organisms to organs including the brain. Because of their small size and potential to transfer across tissues within organisms, NP exposure may pose a higher risk. In some studies, chemical additives found in plastics have been shown to affect heath and reproduction of aquatic organisms.

There are evidence gaps, but this document and other recent reviews conclude that there is sufficient evidence to act in order to reduce the rate of environmental accumulation. This conclusion is based partly on the evidence of harm demonstrated in laboratory studies, and projected increases in accumulation of plastics in the environment. For example, recent consideration of the need for binding restrictions by the ECHA concluded that there was already sufficient evidence of harm to stem the release of MPs into the environment. Similarly, the recent agreement to negotiate a legally binding international UN Plastics Treaty demonstrates a clear consensus that current design, use, and disposal of plastics is problematic and needs to change. The need for action to curb the escape of plastics to the environment is based on a need to protect aquatic life, biodiversity, and ecosystems upon which humanity depends for food, livelihood, and well-being.

#### Recommendations

In the published record of research on MNPs in the ocean, and in human health concerns, it is commonly mentioned that there are almost no data on smaller MNPs in the environment or tissues. This deficiency is largely due to the lack of reliable and cost-effective methods adequate for assessing the presence and abundance of these particles. We strongly recommend a concerted effort and funding to support the development of methods to allow detection of smaller MNPs in environmental media (water, sediment) and in tissues critical to effects (e.g., brain, gonad) and transfer to humans (e.g., fish muscle).

Information on the global distribution of plastics is currently fragmentary and depends on labor-intensive analyses. We strongly recommend international efforts to develop sensor technology to measure quantities of plastics as particles or debris, and deployment of such technology on robotic vehicles for ocean sensing of small particles and to broadly use satellite sensing to identify where plastic debris fields are changing. The information obtained may indicate where impacts may be greater, signaling need for intervention.

Effects of smaller MNPs in the environment are largely unknown; inferences are based on experimental studies most using doses far exceeding the plastics’ levels in the environment. We urge the support of studies to identify the impacts of small MPs and NPs on organisms in the environment itself, and at environmentally relevant doses in model systems.

#### Potential Implications for Human Health

**Figure 3.6:**
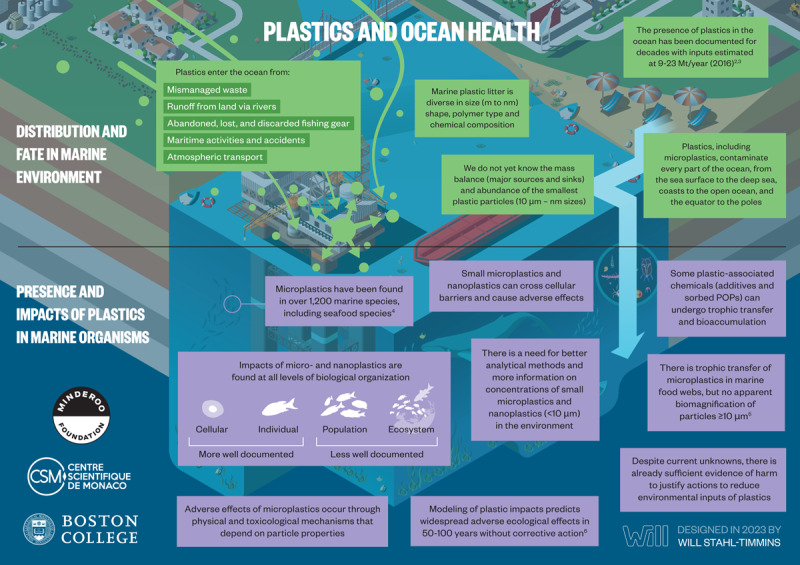
**Distribution, fate, and impacts of plastics in the ocean.** Plastics enter aquatic environments (marine and freshwater), undergo processes that determine their distribution and fate, and impact organisms and ecosystems in a variety of ways. Mt, Megatons; POPs, persistent organic pollutants. **References**: ^[1]^(Rochman *et al.*, 2019); ^[2]^(Lau *et al.*, 2020); ^[3]^(Borrelle *et al.*, 2020); ^[4]^(Santos, Machovsky-Capuska and Andrades, 2021); ^[5]^(Pitt, Aluru and Hahn, 2023); ^[6]^(Everaert *et al.*, 2020). *Credit*: Designed in 2022 by Will Stahl-Timmins.

The presence of plastics in the ocean and their adverse impacts (both demonstrated and potential) are important for the health of the ocean and the earth system more generally (Figure 3.6). In addition, plastics in the ocean have potential impacts on human health and well-being, as described in detail in the following section (Section 4). The potential links between ocean health and human health include:

1. Direct human exposure to plastics and plastic-associated chemicals occurs from consuming contaminated seafood. Billions of people depend on ocean for protein. Although for most people other sources of MP exposure may be more important, seafood could be a major route of exposure for some populations or for some types of plastic.
2. Direct human exposure also may occur from inhalation of plastics injected into the atmosphere, particularly in ocean spray near beaches, or in drinking water from lake and river sources. The levels from these sources are likely less than other atmospheric or water sources, such as indoor air and bottled water.
3. Plastic impacts in the ocean could affect human health indirectly, e.g., through effects on marine resources and climate-related processes, through degradation of ecosystem services (the different types of benefits that healthy ecosystems provide to humans and the environment) [849], and by impeding use of the ocean for healthful recreation. These indirect effects can include impacts on mental health, for example, through the psychological effects of marine litter [957].

Without dramatic change in production, especially of non-essential, single-use plastics, the abundance of plastics in the global environment, including the ocean, will increase. Likewise, the potential impacts of plastics on life in the ocean will increase. The prospect that increasing impacts will alter the role of the ocean in global and human health and wellbeing is inescapable. The human health effects, elaborated in the next section, and ocean health effects are inextricably linked.

## Section 4—The Impact of Plastics on Human Health

### Introduction

Plastic endangers human health and causes disease, disability, and premature death at every stage of its long and complex life cycle—from extraction of the coal, oil, and gas that are its main feedstocks; to transport, manufacture, refining, use, recycling, and combustion; and finally to reuse, recycling, and disposal into the environment [12]. Children are particularly vulnerable (see Box 4.1).

**Box 4.1 Plastics’ Impacts on Children’s Health.**

Infants in the womb and young children are two populations at particularly high risk of plastic-related health effects at every stage of the plastic life cycle. Because of the exquisite sensitivity of early development to hazardous chemicals and children’s unique patterns of exposure, plastic-associated exposures are linked to increased risks of prematurity, stillbirth, low birth weight, birth defects of the reproductive organs, neurodevelopmental impairment, impaired lung growth, and childhood cancer. Early-life exposures to plastic-associated chemicals also increase the risk of multiple noncommunicable diseases later in life.

The concept that noncommunicable diseases in adult life, such as cardiovascular disease, diabetes, cancer, and dementia, can result from adverse environmental exposures during fetal life or early infancy was elucidated in the early 2000s in the Developmental Origins of Health and Disease (DOHaD) concept [958]. This concept developed from the earlier work of David Barker and his colleagues and is based on long-term epidemiologic studies of adults who had been exposed to adverse environmental influences in utero and in early childhood [959]. The DOHaD construct provides an intellectual framework for conceptualizing the long-term impacts of plastic-associated chemicals on human health.

Plastics’ disproportionate impacts on children’s health are seen in communities near coal mines, oil wells, and fracking sites. They are seen in the low-income, largely minority “fenceline” communities adjacent to plastic production facilities. They are seen among children exposed to plastic during its use in homes, schools, and playgrounds. They are seen in children who live adjacent to plastic waste disposal sites in all countries and especially in the low-income countries to which so much of the world’s plastic waste is exported.

When policies for the reduction of plastics’ harms to human health are specifically designed to protect children’s health, they protect the health of all members of exposed populations.

**Figure 4.1:**
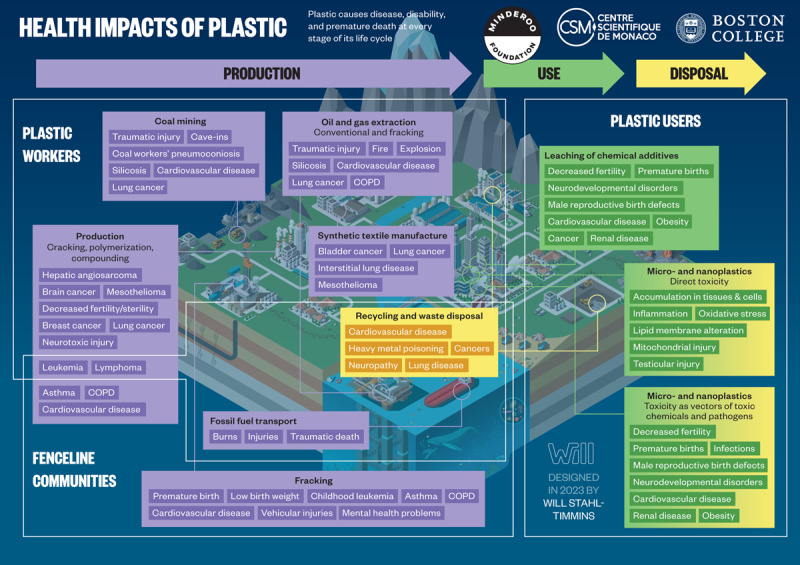
**Health impacts of plastic.** Plastic threatens and harms human health at every stage of its life cycle. COPD is chronic obstructive pulmonary disease. *Credit*: Designed in 2022 by Will Stahl-Timmins.

The purpose of this section, which parallels the structure of Section 2, is to trace plastics’ health impacts at each stage across its life cycle. These impacts are summarized in Figure 4.1.

### Production

#### Health impacts of extraction of carbon feedstocks

Fossil carbon derived from coal, gas, and oil is the raw material for more than 98% of all plastic and the main feedstock for most of the chemicals (petrochemicals) added to plastic [9,12]. Extraction of coal, oil, and gas by mining, conventional drilling, and unconventional drilling (hydraulic fracturing, or “fracking”) is associated with multiple harmful impacts on human health.

##### Coal mining

Coal mining is a physically hazardous occupation with high rates of acute and chronic injury and injury-related death. Coal mining is also responsible for chronic health impairment in miners. Coal dust inhalation can cause coal workers’ pneumoconiosis, silicosis, chronic obstructive airway disease, emphysema, and chronic bronchitis as well as cardiovascular disease [960,961,962]. Miners are exposed to diesel exhaust, a known cause of cardiovascular disease and lung cancer, from trucks and drilling equipment [94].

Coal mining can also cause disease in nearby communities. Coal dust inhalation by pregnant women in communities near mines increases the risk of acute lower respiratory tract infections in their children [962].

##### Oil and gas extraction

Oil and gas extraction are highly hazardous trades, and workers in these occupations are at elevated risk of disease and death caused by fire, explosion, blowouts, and physical injury. Work-related fatality rates in oil and gas development are 2.5 to 7.5 times higher than those in construction and general industry, respectively [94]. Offshore drilling operations are especially dangerous. Oil and gas field workers can be occupationally exposed to silica dust as well as to diesel exhaust from vehicles and drilling equipment [94].

##### Air and water pollution

Exposures to multiple toxic chemicals in air and water compound the physical hazards of coal, oil, and gas extraction and lead to increased risks of noncommunicable disease, disability, and premature death in workers as well as in residents of “fenceline” communities [963,964,965].

###### Air pollution

Air pollutants produced in coal, gas, and oil extraction include particulate matter (PM), NO_x_, sulfur oxides (SO_x_), carbon monoxide, hydrogen sulfide, and volatile C5-C9 hydrocarbons. NO_x_ and SO_x_ are respiratory irritants. Carbon monoxide and hydrogen disulfide can cause sudden death at high concentrations and chronic neurobehavioral impairment at lower levels of exposure [966].

###### Air pollution—Ozone

Ground-level ozone is formed in the air surrounding gas and oil extraction sites by the photochemical reaction of aerosolized hydrocarbons with NO_X_ [967,968]. Ozone is a respiratory irritant, and exposure is especially dangerous for children, the aged, and active adults who spend time outdoors. Ozone exposure can lead to asthma and chronic obstructive pulmonary disease [98]. Atmospheric concentrations of ozone in some gas fields are reported to be as high as those in urban areas. In Utah, the Department of Environmental Quality reported ozone levels in the heavily fracked Uinta Basin at levels 85% higher than US federal health standards in 2010 [969]. Gas field ozone haze can spread widely and has been measured as far as 300 km beyond drilling fields, thus presenting hazards for people in distant communities [967].

###### Air pollution—Particulate matter

Airborne PM pollution is extensive in coal, oil, and gas production and arises from multiple sources, including rock and coal dust from mining operations, diesel exhaust emissions from drilling rigs and transport vehicles, hydrocarbon emissions from wells, and flaring of natural gas [78]. Since 1995, airborne PM emissions from plastic across its life cycle have increased by 70%, with the greatest increases in the production phase [970,971].

PM air pollution causes disease and premature death in exposed workers and “fenceline” community residents. Fine particulates such as PM_2.5_ are the most dangerous airborne particulates because they are small enough to penetrate deep into the lungs, and in some instances, they can enter the bloodstream [972].

In adults, PM_2.5_ exposure increases the risk for cardiovascular disease, stroke, chronic obstructive pulmonary disease, lung cancer, and diabetes [973]. In infants and children, it increases risk for premature birth, low birth weight, stillbirth [974], impaired lung development, and asthma [975]. Prematurity and low birth weight are risk factors for cardiovascular disease, kidney disease, hypertension, and diabetes in adult life, while impaired lung growth increases risk in adult life for chronic respiratory disease [976].

Emerging evidence indicates that PM_2.5_ pollution is additionally associated with neurologic dysfunction in both adults and children. In adults, associations are reported between PM_2.5_ pollution and increased risk of dementia [977]. In children, air pollution is linked to loss of cognitive function (IQ loss), memory deficits, behavioral dysfunction, reductions in brain volume, and increased risks of attention deficit hyperactivity disorder (ADHD) and autism spectrum disorder [978].

Oxidative stress is a key initiating component in the cascade of pathophysiologic events triggered by PM_2.5_ exposure [979,980,981].

###### Air pollution—Volatile organic compounds

Multiple volatile organic compounds (VOCs) are released to the atmosphere by oil and gas extraction. They include benzene, 1,3-butadiene, tetrachloroethane, methane, ethane, propane, toluene, methanol, ethanol, formaldehyde, and acetaldehyde as well as *n*-hexane, styrene, methanol, and 2,2,4-trimethylpentane. Both workers and “fenceline” community residents are exposed to these hazardous air pollutants. VOCs can cause eye, nose, and throat irritation [982]; headaches; loss of coordination [983]; nausea; and damage to the liver, kidneys, and central nervous system [984]. Some, such as benzene, 1,3-butadiene, and formaldehyde are known human carcinogens and can cause leukemia and lymphoma. Others are associated with increased risk of neuropathy and asthma [985,986,987].

###### Water pollution

Leakage of hazardous chemicals from oil and gas extraction can result in pollution of surface water and groundwater with exposures of workers as well as community residents [31]. Chemical pollutants detected in water supplies near drill sites include 1,3-butadiene, tetrachloroethane, benzene, methane, ethane, propane, and toluene as well as methanol, ethanol, formaldehyde, and acetaldehyde [985,987]. Many of these chemicals are associated with human health effects, such as cancer, skin and eye irritations, and GI effects [93,988,989,990]. Indeed, exposure to contaminated water from oil and gas extraction has been shown to cause several health impacts, including neurological, GI, and dermatological effects [991].

##### Community health impacts of oil and gas extraction

Oil and gas development are associated with multiple adverse health effects in nearby communities [992,993]. Residents report foul-smelling air and burning eyes as well as coughing, migraines, dizziness, memory loss, and numbness in their hands, feet, arms, and legs. Diseases of livestock are reported, with cattle wasting and dying, and fewer calves born or surviving [991]. High rates of automobile accidents involving tanker trucks are reported in communities neighboring oil and gas extraction sites [94]. Increased cancers at various anatomic sites have been reported among adults in these communities [994] as well as in children (see Box 4.2).

**Box 4.2 Oil and Gas Extraction and Pediatric Cancer.**

Oil and gas are major feedstocks for plastic production. Many of the compounds used or produced in oil and gas extraction by drilling and fracking—most notably benzene, 1,3-butadiene, and formaldehyde—are known to cause leukemia and lymphoma in persons of all ages, including children [995]. Exposures to these materials during pregnancy and in early childhood are especially dangerous [996,997]. Epidemiological studies conducted among children born or living near fracking sites have found elevated rates of childhood cancer, especially leukemia, and congenital heart defects [996,998].

A 2017 registry-based study in Colorado found that children and young adults diagnosed with leukemia were four times more likely than controls to live in areas of extractive activity [996]. A Pennsylvania study found a nearly 2-fold increase over background in risk of acute lymphatic leukemia among children with at least one unconventional oil and gas development well within 2 km of their residence during the prenatal period and a 2.8-fold increase in risk of leukemia among children who had at least one unconventional oil and gas development well within 2 km of their residence at ages two to seven years [32].

#### Health impacts of fossil carbon transport

Coal, oil, and gas destined for use as plastic feedstocks are transported by pipeline, ship, rail, and road from mines and drill sites to chemical and plastic production sites. Because many plastic feedstocks are toxic, flammable, and explosive, all of these modes of transport are associated with hazards to human health and the environment, as was seen in the February 2023 rail car disaster in East Palestine, OH, USA.

##### Gas leaks

Fires and explosions are the major health hazards associated with fossil carbon transport, and most involve gas. The major components of gas, which vary with site of origin, are methane (60%–90%), ethane (0–20%), propane (0–20%), and butane (0–20%) [999]. All are flammable and explosive. The higher the pressure in a pipeline, the greater the danger of fire and explosion.

**Figure 4.2:**
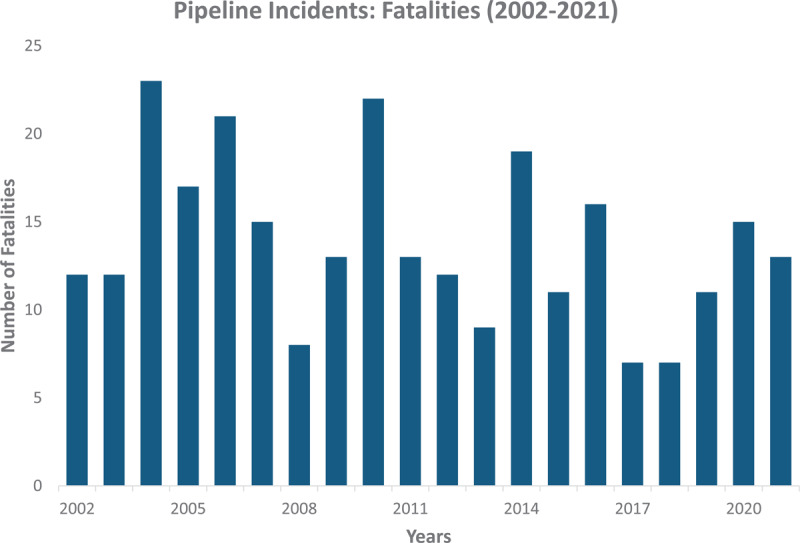
**Fatalities caused by pipeline incidents**. *Permission*: Pipeline and Hazardous Materials Safety Administration (no date) *Pipeline incident 20-year trends | phmsa*. Available at: https://www.phmsa.dot.gov/data-and-statistics/pipeline/pipeline-incident-20-year-trends (Accessed: 24 October 2022). Figure adapted from (Pipeline and Hazardous Materials Safety Administration (PHMSA), 2022) by Manuel Brunner (co-author).

Multiple gas pipeline explosions occur globally each year. In the US, the Pipeline and Hazardous Materials Safety Administration has collected data on more than 3,200 accidents deemed “serious” or “significant” since 1987 [1000]. (Figure 4.2)

Injuries, burns and deaths result from pipeline fires and explosions. Eighty explosions in a pipeline in Massachusetts in 2018 damaged 130 buildings, injured 23 people (including 2 firefighters), and killed 1 young man [1001]. Other notable explosions in the US have occurred in Armada Township, Michigan; Refugio, Texas; and Watford City, North Dakota. An April 2016 explosion in a high-pressure pipeline in Salem, Pennsylvania, created a crater 9 m wide, 15 m long, and 4 m deep; destroyed a house 60 m away; melted the siding off a house 300 m away; charred trees and telephone poles 1.6 km away; and hospitalized a man in his twenties with third-degree burns on over 75% of his body [1002].

Compressor stations, which are sited at intervals along pipelines to push gas forward, leak gas into surrounding communities. Additionally, the diesel engines that power compressors produce incessant noise and generate both particulate and hazardous air pollutants, notably benzene, 1,3-butadiene, and formaldehyde—all known human carcinogens [1003]. Pipelines, and compressor stations are disproportionately sited in low-income, minority, and marginalized communities—environmental justice communities—where they deepen social injustice while producing no local benefit [1004].

##### Oil leaks and spills

**Figure 4.3:**
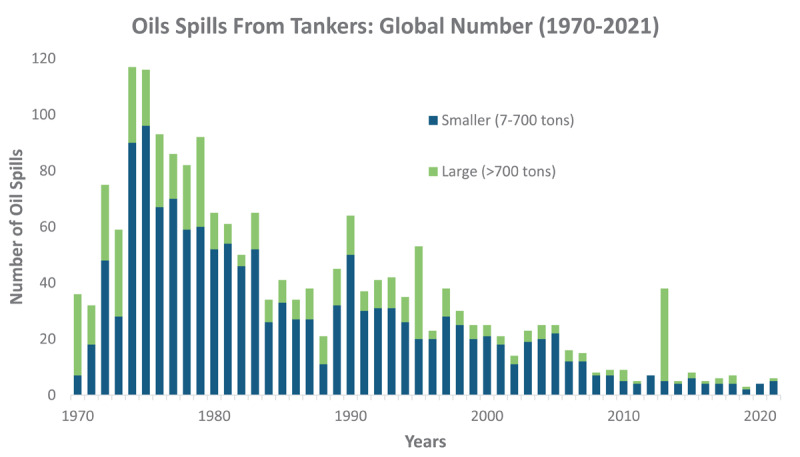
**Global number of oil spills from tankers from 1970–2021**. *Permissions*: no special permissions needed. Roser, M. and Ritchie, H. (2022) ‘Oil spills’, *Our World in Data* [Preprint]. Available at: https://ourworldindata.org/oil-spills (Accessed: 24 October 2022) and: ITOPF (2022). Oil tanker spill statistics 2021. ITOPF Ltd, London, UK. Figure adapted by Manuel Brunner (co-author).

Oil spills are numerous, (Figure 4.3) and associated with an increased prevalence of respiratory problems, neurological effects, genotoxicity, endocrine disruption, immune dysfunction, and mental health disorders in both cleanup workers and community residents [119]. All of these effects were seen after the Exxon Valdez oil spill in Alaska in 1989 and following the Deepwater Horizon oil spill in the Gulf of Mexico in 2010. Improper site management following leaks is associated with elevated atmospheric concentrations of methane, benzene, mercury, xylenes, *n*-hexane, and toluene [1005].

#### Health impacts of plastic production

##### Health impacts of cracking

**Figure 4.4:**
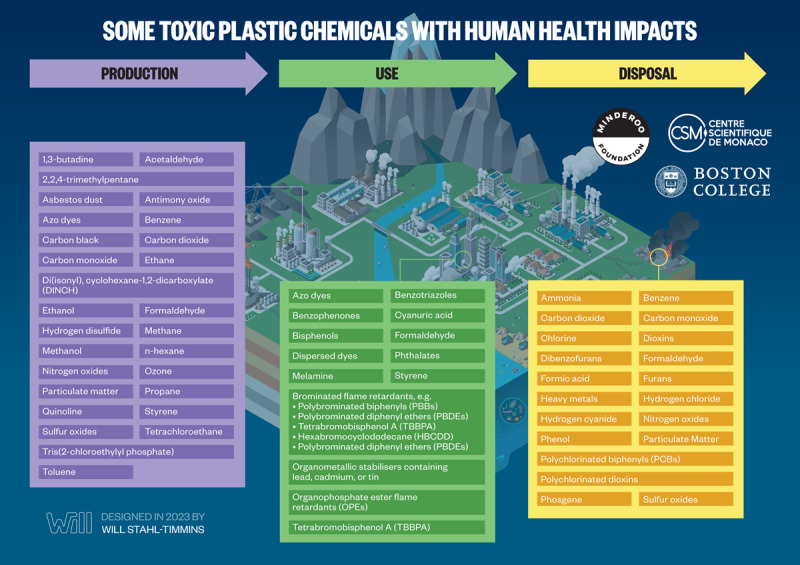
**Some toxic chemicals with human health impacts.** The human health impacts of the chemicals listed in each phase of the plastic life cycle are described in the text. *Credit*: Designed in 2022 by Will Stahl-Timmins.

Catalytic cracking of coal, oil, and gas to create ethylene and propylene feedstocks is the first step in plastic production. Cracking occurs in massive, highly energy-intensive chemical plants. These facilities generate multiple air pollutants, including carbon monoxide, NO_x_, SO_X_, VOCs, and PM (Figure 4.4). They also release substantial quantities of CO_2_ and methane, thus contributing substantially to climate change [159]. They endanger the health of chemical workers and residents of nearby “fenceline” communities [159]. They are often sited in environmental justice communities.

The principal health hazards to cracking workers result from their occupational exposures to toxic and carcinogenic petrochemicals. These include solvents (benzene, toluene, and xylene), 1,3-butadiene, and styrene [159]. Benzene and butadiene cause leukemias and lymphomas and are classed by the International Agency for Research on Cancer (IARC) as proven human carcinogens [132]. Styrene is neurotoxic and is classed by the IARC as a possible human carcinogen [1006]. Human toxicity impact potentials [1007] have been calculated across the cracking life cycle and include cancer, noncancer, and respiratory as well as environmental impact potentials of smog formation, freshwater ecotoxicity, and ozone depletion [159].

Risk of fire and explosion is high within refineries and cracking plants. From January 2000 to July 2013, 171 fires occurred in oil refineries in the US. Of these, about 8% led to explosions, which can result in serious injuries and death [1008]. At least 58 workers have died at oil refineries and cracking facilities in the US since 2005 [1009].

##### Health impacts of refinement, polymerization, compounding, and conversion

Many of the monomers, additives, and catalysts used to form plastic are highly toxic, and a number are carcinogenic [165]. These chemicals are released into workplace air in dust and vapor formed at virtually every stage of the production process—during handling and mixing of resins and additives, processing under heat and pressure, and finishing products after molding—and results in exposures of these chemicals to workers and to residents of nearby communities.

###### Occupational health hazards

Plastic production workers are occupationally exposed to toxic monomers (e.g., vinyl chloride, styrene, BPA, acrylonitrile, butadiene, ethylene, urethane); additives (e.g., phthalates, heavy metal stabilizers, flame retardants, hydrocarbons); dust, including airborne asbestos dust [965,1010,1011]; and hazardous vapors (Figure 4.4). Compared to nonexposed workers, chemical body burdens in epoxy-resin workers are high: acrylonitrile (11-fold elevation above background), styrene (5.5-fold elevation) [238], di(2-ethylhexyl) phthalate (DEHP; 2-fold elevation), and BPA (2-fold elevation) [1012]. Elevated body burdens were even observed in workplaces where airborne concentrations of hazardous vapors were within legally permissible limits, and they remained evident after one or more days away from work.

Toxic occupational exposures in plastic production workers are associated with a range of diseases:

- Men exposed to vinyl chloride monomer in liquid and vapor form in vinyl chloride polymerization plants have increased mortality from angiosarcoma of the liver, brain cancer, and connective and soft tissue cancers, with risks being highest in the most heavily exposed [27,1013].
- Workers in petroleum refinery and petrochemical plants have increased incidence of and mortality from mesothelioma due to airborne asbestos exposures [965] and are at increased risk of other asbestos-related diseases, such as asbestosis, lung cancer, and ovarian cancer [1014,1015].
- Benzene causes leukemias and lymphomas with strongly positive exposure-response relationships [28].
- Leukemias and lymphomas are caused by 1,3-butadiene [1016].
- Formaldehyde causes leukemias and lymphomas as well as respiratory cancers [1017].
- Styrene is neurotoxic and a possible human carcinogen [28].
- Women workers in plastic production are at disproportionate risk of reproductive harms. More than a doubling of breast cancer risk is reported among women working in automotive plastics manufacture and in synthetic textile manufacture. Increased breast cancer risks are also reported among men employed in plastics manufacture [238].
- Male infertility has been documented among workers in the plastics and rubber industries [1018].

###### Community health impacts

Petrochemical refining facilities, including cracking plants and plastic production facilities, are disproportionately located in low-income communities [1019]. In the US, over 530,000 people live within three miles of chemical refineries, with the majority being people of color and people living below the poverty line [1020]. People in these communities are exposed to hazardous chemical pollutants released from cracking plants into air, water, and soil. These pollutants include benzene, formaldehyde, toluene, and other hazardous air pollutants with recognized mutagenic, carcinogenic, and reproductive toxicity [99,1021,1022].

- Leukemia risk was found to be 30% higher among 187,585 residents of “fenceline” communities compared to residents of more distant communities in a large meta-analysis [1023].
- Lung cancer risk has been reported to be 19% higher among residents of communities near petrochemical plants than in the general population [1024].
- Asthma rates in both adults and children, lung and respiratory infections, and cardiovascular problems are all increased among individuals living near a petrochemical site and are attributed to inhalation exposures to VOCs and airborne PM [1025,1026].
- Rates of premature rupture of membranes [1027], premature births, and low birth weight babies are all elevated in “fenceline” communities and are attributed to prenatal exposures to hazardous chemicals [1028].

##### Health impacts of synthetic textile manufacture

Synthetic, plastic-based fibers are used in very large quantities to manufacture clothing, furniture, carpeting, tires, and myriad other consumer goods. Physical hazards of synthetic textile production include fire, noise, temperature, humidity, and unsafe machinery. Synthetic textile manufacture also results in occupational exposures to toxic chemicals and airborne dusts, including airborne plastic microfibers. Multiple diseases have been reported among workers in this industry [243]:

- Azo dyes derived from aromatic amines are genotoxic and highly carcinogenic as well as allergenic. They were among the first synthetic chemicals to be produced and were documented as early as 1895 to cause bladder cancer in dye workers [1029].
- Disperse dyes (small polar dye molecules) used to stain synthetic fibers are among the most common causes of textile allergy [246,247].
- Quinoline and its derivatives are used extensively in the manufacture of textile dyes. Some are skin irritants. Quinolines are classified by the IARC as “possibly carcinogenic to humans.” [1030]
- Exposure to airborne microfibers among workers producing “flock,” velvet-like and fleeced fabrics from recycled, pulverized, or cut nylon, polyester, polyolefins polyethylene (PE), and polypropylene (PP) are at increased risk of flock workers’ lung disease. This illness is characterized by fevers, asthma-like symptoms, lesions in the lower airways, interstitial fibrosis, extrinsic allergic alveolitis, chronic bronchitis, pneumothorax and chronic pneumonia, decreased lung function, and lung cancer [1031].
- Workers making car interiors from synthetic fabric coated with antinomy oxide and tris(2-chloroethyl) phosphate, a flame retardant, are exposed to dust and experience breathing problems [1032].

### Health Impacts of Plastics during Use

Plastics are used to produce an enormous range of industrial and consumer goods that include construction materials, electronics, medical equipment, and children’s toys. Virtually all plastics-based products contain a wide range of chemical additives, often in very large quantities. These additives, recently estimated to number more than 10,500, include plasticizers, stabilizers, dyes, and flame retardants [8]. Depending on the product, additives can comprise 5%–50% by weight of manufactured plastics [213]. Most additives do not form strong chemical bonds with the polymer matrix. They can therefore leach from plastic to contaminate air, water, and soil and expose humans [170].

Systematic reviews and meta-analyses were searched for on PubMed and Epistemonoikos [1033,1034]. Primary research articles were searched in the Medline and Embase databases using the Ovid search platform, with additional searches on PubMed. Where available, we focus on in vivo human health research with direct quantification of chemicals in biospecimens such as blood or urine. Where human health research was absent, additional searches were made for the animal and in vitro literature.

Most health hazards associated with plastics in the use phase result from exposures to toxic additives and other plastic-associated chemicals. For this reason, it is very important to consider additives in any discussion of the toxicity of plastics as well as in all negotiations on a Global Plastic Treaty.

Additional health hazards of plastics in the use phase result from ingestion and inhalation of (MNPs) formed through the erosion and breakdown of plastics. An emerging body of evidence indicates that MNPs may cause direct toxic effects due to their physical accumulation in cells and tissues [1035] and that they can also cause toxicity by acting as vectors, “Trojan horses,” that transport chemical additives, adsorbed chemicals, and pathogens into tissues and cells [882,1036]. Nevertheless, the contribution of MNPs to the transfer and bioaccumulation of adsorbed chemicals may be negligible compared to direct environmental transfer and bioaccumulation via prey and depends on multiple factors, such as the characteristics of the particles as well as the environmental milieu in which they are found [811,1037].

#### Health impacts of plastic additives

Exposures to plastic additives such as polychlorinated biphenyls (PCBs), BPA, phthalates, and BFRs are widespread, reflecting the great increases in plastic production and use in recent decades. In the US, the Centers for Disease Control and Prevention’s National Biomonitoring Program finds BPA and phthalates in the urine of nearly all people tested [1038,1039]. Children aged 6–11 years had the highest levels, followed by adolescents aged 12–19 years, non-Hispanic Blacks, and participants with lower-income levels.

Multiple sources of human exposure to plastic additives have been documented:

- Phthalates in concentrations above 0.1% of mass, i.e., above EU limits (EC No 552/2009), have been reported in nylon sheets, cot mattresses, and diaper changing mats [253].
- Bisphenols, BPA and BPS, have been detected in textiles, including those marketed for infants, at concentrations between 15 ng/g and 366 ng/g [249]. These concentrations are above a draft European Food Safety Authority recommendation for tolerable daily intake [252].
- Phthalates are found in high concentrations in textiles with polyvinyl chloride (PVC) prints used to make children’s clothing. Phthalate concentrations in these materials are reported to range from 1.4 mg/kg to 200,000 mg/kg (20% by sample weight) [250].
- Formaldehyde-releasing compounds and resins are used in permanent press fabrics to prevent creasing [1040].
- Textiles are increasingly treated with metal nanoparticles as antimicrobial agents (silver) or UV absorption (titanium). These materials can leak from the fabrics to expose workers and consumers [248].

##### Endocrine disruptors

A number of plastic additives act as EDCs—synthetic chemicals that “interfere with the synthesis, secretion, transport, metabolism, binding, or elimination of natural hormones responsible for homeostasis, reproduction, and developmental process.” [1041]

The endocrine signaling system regulates every aspect of early human development, including body growth, organ formation, and development of the brain, reproductive system, and immune system. For this reason, EDC exposures in early life—during pregnancy and in the first two years after birth—are extremely dangerous because they can cause changes within developing cells and organs that disrupt organ formation and increase risk of disease and disability in childhood and across the life span (see Box 4.1) [1042]. EDCs in a pregnant woman’s body can be transmitted to her child through the placenta during pregnancy and through breast milk during lactation. These chemicals can also cross the developing blood-brain barrier in young children, allowing toxic levels to accumulate in brain tissue [1043,1044]. Children’s vulnerability to EDCs is further magnified by their age-appropriate behaviors, such as hand-to-mouth activity, crawling, and persistent contact with soil [1045], all of which increase exposure, and by the immaturity of enzymatic pathways in their liver and kidneys, which prevent them from efficiently detoxifying and eliminating EDCs [1045].

Many EDCs are lipophilic, bioaccumulate in adipose tissue, and persist in the body, thus leading to continuing exposure [1041]. At a societal level, extensive exposure to EDCs that results in widespread disease, disability, and premature death can have substantial economic impacts that are the consequence of lifelong increases in health care costs and decreases in productivity [1046].

###### Reproductive toxicity of EDCs

EDCs such as BPA, PCBs, and phthalates can interfere with androgens and estrogens and thus disrupt sexual and reproductive development and function [1047]. The consequences are increased rates of premature births; increased frequency of birth defects, such as cryptorchidism and shortened anogenital distances in the reproductive organs of baby boys; increased risk for postnatal morbidity and mortality; and decreased fertility [1042,1047].

###### Metabolic toxicity of EDCs

Some EDCs interfere with the actions of the thyroid hormone, thus disrupting energy metabolism in cells throughout the body [1041]. Others, termed *obesogens*, reprogram the insulin-glucagon axis that regulates appetite and energy balance [1041,1047], thus increasing the risk for obesity and diabetes. In experimental animals as well as in epidemiologic studies, perinatal exposures to obesogenic EDCs such as BPA induce weight gain and body fat accumulation [1043,1048].

###### Cardiovascular and renal toxicity of EDCs

The metabolic changes initiated by obesogenic EDCs, including changes in serum lipid profiles and increased risk of hypertension, increase subsequent risk for cardiovascular disease and stroke [1049].

A major epidemiological study based on the NHANES survey in the general population of the US found that persons in the highest quartile of urinary BPA concentration had a significantly increased prevalence of myocardial infarction (odds ratio [OR] = 1.73, 95% CI = 1.11–2.69) and of stroke (OR = 1.61, 95% CI = 1.09–2.36) compared with persons in the lowest quartile [1050]. Additional epidemiological studies have found a positive relationship between BPA exposures and renal dysfunction [1051,1052].

Phthalate and PCB exposures are both associated with increased mortality from cardiovascular disease, and positive exposure-response trends are seen in these effects [1053].

##### Neurotoxicity

Some plastic additives are neurotoxic in addition to being endocrine disruptors. Infants in the womb and children in the first years after birth are particularly vulnerable to brain injury caused by these chemicals because their brains are rapidly growing and developing throughout the first thousand days of life and continue to develop through childhood and adolescence and into early adulthood (see Box 4.3) [1054,1055,1056]. Injury to the developing brain caused by neurotoxic plastic additives results in neurodevelopmental disorders, behavioral change, and diminished cognitive function (reduced IQ). An unresolved question is whether brain injury caused by toxic chemicals in early life increases risk for dementia and other neurodegenerative disorders in later life.

**Box 4.3 The Vulnerability of the Developing Human Brain to Neurotoxic Chemicals.**

An estimated one in six children in the US has a neurodevelopmental disorder, about 17% of all children [1057]. Prevalence rates of these disorders, including ADHD, autism spectrum disorder, cognitive impairment (IQ loss), dyslexia, reduced academic performance, behavioral changes, and reductions in brain volume appear to be increasing [1058,1059]. Toxic chemicals in the environment, including chemicals added to plastic, are important causes of neurodevelopmental disorders in children [1059,1060].

The brain and nervous system begin to develop in the first month of pregnancy with the formation of a thin strip of cells—the neural plate—along the dorsal side of the embryo [1061]. The cells in the neural plate are the precursors of the brain and spinal cord. Over the course of pregnancy, these cells divide and multiply at a very rapid rate, and by the time a baby is born, the brain contains approximately 100 billion neurons [1062]. As they are dividing and multiplying, neurons migrate from the positions where they are formed to their final destinations. As they migrate, the neurons form dense networks of connections with one another [1061]—an estimated 2,500 connections per neuron by the time of birth [1062]. Each connection must be precisely established, and redundant neurons and connections are pruned away through programmed cell death, or apoptosis. Almost 50% of the neurons present in the brain at birth have undergone apoptosis by adolescence [1061]. All of these developments are highly interdependent, and optimal brain development requires that each step must occur in its proper sequence.

A consequence of the great complexity of human brain development is exquisite vulnerability to toxic chemicals and other harmful environmental exposures [1042,1043,1045,1047]. Exposures to even minute doses of toxic chemicals during critical developmental stages can have lifelong consequences for brain function and neurological health [1058,1060,1063]. These windows of vulnerability are unparalleled in adults.

Multiple toxic chemicals have been found to induce adverse neurological effects in children at levels previously thought to be safe and that produce no adverse outcomes in adults [206,1060,1064,1065,1066,1067,1068]. Brain damage caused by exposure to toxic chemicals early in life may be chronic, irreversible, and difficult to treat [1058]. Prevention of exposure is the most effective strategy for safeguarding the developing human brain against toxic chemicals [1058,1069,1070]. Dr David Rall, former director of the US National Institute of Environmental Health Sciences, famously stated that “if thalidomide had caused a 10-point loss of IQ instead of obvious birth defects of the limbs, it would probably still be on the market [1071].”

###### Neurotoxicity of PCBs

Prenatal exposures to PCBs are associated with lower IQ and difficulties with motor skills, attention, and memory [1072,1073,1074,1075,1076].

###### Neurotoxicity of phthalates

Prenatal exposure to phthalates has been associated with increased risk for autism spectrum disorder [1077,1078,1079]. Certain phthalates are associated with poorer motor skills in children [1068,1079]. Prenatal exposures to phthalates are also associated with aggression, depression, conduct and attention problems, externalization of problems [1064], and diminished executive functioning in preschool children of both sexes [1064]. Mothers with higher exposure to phthalates during pregnancy are three times more likely than other women to have children diagnosed with ADHD [1065].

Phthalates act as anti-androgens in the developing central nervous system, disrupting the normal sexual differentiation of the brain. This finding may explain the sex-specificity of disruption in brain function in children exposed to phthalates, such as the observation that the exposure of baby boys to phthalates has been associated with less male-typical play behavior [1066]. Phthalates also interfere with estrogen synthesis and thyroid hormone production, both of which exert powerful influence on brain development. Prenatal BPA exposure is reported to influence hypothalamic morphology, concentrations of total testosterone, and sex-dependent neurological behaviors [1080].

A study to assess the impact of prenatal phthalate exposure on cognitive function conducted among 328 inner-city mothers and their children found that children whose mothers had the highest concentration of certain phthalates had significantly lower scores on perceptual reasoning (by 3.9 IQ points) and verbal comprehension (by 4.4 IQ points), respectively [1067]. A follow-up study of the children at age seven years showed similar results, where children born to women above the 75th concentration percentile for phthalates scored 6.6 and 7.6 IQ points lower than their less heavily exposed peers. These findings are consistent with those of other studies [1079] and suggest that the adverse impacts of prenatal phthalate exposure on cognitive function can persist into the early school years, with negative implications for academic performance and future earnings potential.

###### Neurotoxicity of BPA and BPA substitutes

Multiple and diverse adverse emotional and behavioral outcomes are associated with both in vitro and childhood BPA exposure [1081]. Maternal BPA exposure during pregnancy has been found to increase hyperactivity and aggression in two-year-old girls [1082]. In boys, prenatal BPA exposure is associated with increased aggression and emotional reactivity at ages three to five years [1083] and with increased depression and anxiety at age seven years. One study found much poorer scores across several behaviors in boys than girls, including withdrawal, depression, rule breaking, defiance, and conduct problems. These findings suggest the impacts of BPA exposure on the developing human brain may be sexually dimorphic [1084].

Studies in experimental animals confirm the findings from human studies, similarly finding neurodevelopmental and behavioral abnormalities following BPA exposure. Examples include locomotor deficits, anxiety-like behavior, and declarative memory impairments that persisted into old age following prenatal exposure [1085] and hyperactivity, anxiety-like behaviors, and elevated dopamine levels following exposure to BPA [1080]. A review found that BPA induces aggression, anxiety, cognitive deficits, and learning memory impairment [1080].

Studies to elucidate the possible mechanisms underlying these behavioral changes have found that BPA exposure during critical windows of development, such as breastfeeding and organogenesis, upregulates dopamine receptor function, while exposure at other time points does not have this effect [1080]. BPA exposure has been found to increase DNA methylation in rodents, and this change can be passed to future generations [1080]. Increasing evidence suggests that BPA substitutes can induce similar toxicity (see Box 4.4).

**Box 4.4 The Problem of Regrettable Substitution.**

The concept of regrettable substitution—the replacement of toxic chemicals in plastics with new materials that were never assessed for toxicity and subsequently found to be toxic—is exemplified by the flame retardant additives previously described. Without more robust, legally mandated premarket evaluation processes and without systems in place for early postmarket detection of unforeseen adverse effects, there is a risk for further regrettable substitutions as markets shift away from chemicals with established toxicity, such as BPA and phthalate plasticizers, to other bisphenol analogs and alternative plasticizers [197]. Similar to the chemicals they replace, these substitutes are able to leach out from plastic products, leading to environmental contamination and human exposure [197], and there is an urgency to have a process to identify potential hazards. We consider some selected examples here.

***Di-isononyl cyclohexane-1,2-dicarboxylate (DINCH)*.** DINCH is commonly used to replace other phthalate plasticizers, such as DEHP and di-isononyl phthalate [1086], most frequently in food packaging products, toys, and medical devices [1087]. DINCH is oxidized to various metabolites in the human body, and these metabolites have been found to activate human nuclear receptors in vitro and thus interfere with hormonal activity [1086]. In vitro studies have shown that DINCH can alter lipid metabolism in steroidogenic cells and Sertoli cells and induce oxidative stress in the Sertoli cell line, indicating its potential to damage the endocrine and reproductive systems [1088]. Additional in vitro studies have found that DINCH exposure is associated with enhanced inflammatory responses in human macrophages and enhanced cellular stress [1089]. Studies conducted in zebrafish larvae have indicated that DINCH can impact transcriptional profiles, lipid metabolism, and behavior [1090].

Human epidemiological research confirms widespread exposure to DINCH [1091,1092,1093,1094,1095,1096,1097,1098,1099]. In the last seven years, a small number of epidemiological studies have now started to evaluate association with human health outcomes, with a particular focus on reproductive outcomes. Adverse associations have been observed between DINCH exposure and multiple outcomes, including oxidative stress in men [1093] and pregnant women [1095], sperm epigenetics [1092], in vitro fertilization outcomes [211,1091], risk of preterm birth related to preconceptual and or prenatal exposure [1099], and fibroids in women [1096]. More robust evaluation is however limited by sensitivity of the techniques used to detect exposure, resulting in low detection rates in most of these studies.

***Di(2-ethylhexyl) adipate (DEHA)*.** DEHA belongs to a second group of replacement plasticizers, but again, it has endocrine activity with potential for endocrine disruption, specifically with respect to metabolic and reproductive function, at least at high dose. In rat studies, high-dose intravenous injections of DEHA were shown to decrease the animals’ appetite and weight gain, increase liver weight in females, and decrease thymus weight in both males and females, indicating the compound’s disruptive effect on metabolism. In vitro studies demonstrate that DEHA exposure can alter mitochondrial activity in steroidogenic cells, disrupting the ability of these cells to synthesize sex hormones [1088]. In silico experiments show that DEHA is able to bind the ligand-binding pocket of human sex hormone–binding globulin, subsequently preventing sex hormones from binding and therefore interrupting normal endocrine function [212]. While DEHA exposure has been confirmed in human observational studies, there are not yet any observational studies to directly evaluate safety at current levels of exposure, including in terms of endocrine effects seen in animal and in vitro studies [1100].

***Acetyl tributyl citrate (ATBC)*.** ATBC is a plasticizer from a third group, the citrate esters. As with DINCH and DEHA, however, ATBC again has endocrine activity with potential for endocrine disruption, specifically with respect to reproductive function. ATBC is able to bind the ligand-binding pocket of human sex hormone–binding globulin in a similar manner to DEHA and thus has the potential to disrupt human sex hormone regulation [212]. ATBC was additionally found to have anti-estrogenic and anti-androgenic effects in the uterotrophic assay and steroidogenesis assays, respectively [1101]. Further in vitro studies using mouse cells demonstrated that exposure to ATBC reduced the viability of Leydig and fibroblast cells, with a greater effect demonstrated on Leydig cells, indicating the anti-androgenic effects of ATBC [1102]. In vivo studies have demonstrated that ATBC can slightly impair liver function in rats and interfere with the animals’ reproduction and development [1103]. There are not yet any observational studies to have directly evaluated human safety, including in terms of endocrine effects seen in vitro [1100].

***Bisphenol S (BPS) and bisphenol F (BPF)*.** BPS and BPF are chemically similar analogs of BPA, with similar application. In vivo and in vitro studies additionally demonstrated that they have endocrine-disrupting effects similar to those of BPA [1104]. Zebrafish assays studying BPA, BPS, and BPF have shown that all three chemicals can influence estrogenic activity [1105]. Similarly, studies in pigs have revealed that BPS can interfere with meiotic activity in oocytes [1106]. BPA, BPS, BPF, and bisphenol AF were all found to genetically alter steroidogenesis in H295R steroidogenic cells [1107]. Disruption of the thyroid hormone stimulating pathway as a result of BPA, BPS, and BPF exposure has also been documented in tadpoles both in vivo and in vitro, revealing the potential capacity of bisphenols as metabolic disruptors [1108]. A recent scoping review identified 21 human epidemiological studies on BPA and bisphenol analogs [1109]. BPS and BPF exposure have been associated with metabolic problems, such as increased risk of type 2 diabetes; pregnancy and reproductive issues, such as increased risk of late-term birth for girls; and skin sensitivity [1109].

###### Neurotoxicity of BFRs

BFRs, including polybrominated biphenyls (PBBs), polybrominated diphenyl ethers (PBDEs), hexabromocyclododecane (HBCDD), and tetrabromobisphenol A (TBBPA), are added to consumer plastic products such as furniture, carpets, drapes, and consumer electronics to reduce flammability. A number of these compounds have been shown to be neurotoxic [181,184,189,192,197,1110,1111,1112].

The toxicity of PBBs was discovered through investigation of a large contamination episode that occurred in Michigan in 1973 and resulted in the deaths of thousands of dairy cattle and other farm animals as well as widespread contamination of milk and other dairy products, beef, pork, lamb, chicken, and eggs. Human exposure was extensive [1113].

To study the human health effects of PBB exposure, a cohort of around 4,000 people—the Michigan Long-Term PBB study—was formed. It included workers at the chemical plant where the mislabeling had occurred, farm families with varying degrees of exposure, and members of the general public. The study found some evidence linking high PBB exposure to increased risk of breast cancer, lymphoma, and cancer of the digestive system [1114,1115]. Spontaneous abortion rates and increased weight loss were also seen in the Michigan cohort [1116]. Early-life exposure to PBBs has been shown to be associated with developmental neurotoxicity [1117]. In everyday exposure scenarios, PBB has been associated with increased risk of papillary thyroid cancer in women [1118], type 2 diabetes [1119,1120], and deep infiltrating endometriosis [1121].

PBDEs can leach from furniture, draperies, and electronics and settle into house dust [1122]. PBDEs in house dust can include both newer PBDEs as well as older and more highly toxic phased-out members of the class that are still present in older furniture [191,1110]. PBDEs in house dust are a source of exposure for young children who crawl on the floor and exhibit age-appropriate behaviors, such as frequent touching of objects and hand-to-mouth activity [1110]. Some of the highest PBDE body burdens in the world have been reported in California children, because California’s Fire Safety Law Technical Bulletin 117 requires that furniture and baby and other household products contain very high levels of PBDEs [1111].

To investigate the developmental neurotoxicity of PBDEs, a 2013 California study undertaken within the Center for the Health Assessment of Mothers and Children of Salinas cohort analyzed the relationship between pre- and postnatal PBDE exposure and neurological functioning in children [1111]. Researchers found that both gestational and early childhood exposure to PBDEs were associated with adverse neurobehavioral development, including shortened attention span and poorer fine motor coordination at five and seven years of age, and a decrease in verbal and full-IQ scale at seven years [1111].

A 2010 longitudinal study analyzed PBDE levels in 210 cord blood specimens and assessed neurodevelopmental effects in children up to 72 months. Researchers found that children with higher cord blood concentrations of BDEs 47, 99, or 100 scored lower on mental and physical development tests such as IQ [1110]. Other epidemiological studies have found negative associations between prenatal PBDE exposure and impaired cognitive function, attention problems, anxious behavior, and increased withdrawal as well as with reductions in psychomotor development index and lower full-scale IQ performances [1123]. In these studies, PBDE congeners 47, 99, and 100 have consistently been associated with lower cognitive functioning [1124].

###### Neurotoxicity of organophosphate ester (OPE) flame retardants

OPE flame retardants are structurally similar to organophosphate pesticides of known neurotoxicity, such as chlorpyrifos [192]. Human studies on the neurotoxicity of OPFRs have reported associations between early-life exposure to OPEs, decreased childhood IQ, and disrupted internalizing and externalizing behaviors [188]. In further studies, associations have been found between higher prenatal levels of OPE metabolites and poorer performance on cognitive tests, more frequent withdrawal and attention problems, and hyperactivity. Higher prenatal OPE metabolite levels were associated with poorer IQ scores at seven years of age and with more hyperactive behaviors [1125].

###### Stabilizers

Stabilizers are a class of additives that protect plastic from degradation by oxidation, light, and heat [179]. ECHA’s “Mapping Exercise—Plastic Additives Initiative,” [214] classified stabilizer chemicals as “light stabilizers” (N = 17), “heat stabilizers” (N = 27), “antioxidants” (N = 26), and “other stabilizers” (N = 22). We follow this categorization here.

A second approach to categorizing stabilizers involves hazard assessment by chemical class. Groh et al. estimated “harmonized hazard scores” for six groups of stabilizer chemicals based on (1) environmental effects, (2) human health hazards, (3) endocrine disruption (based on the EU REACH, the UNEP and the WHO), (4) PBT properties, and (5) very persistent/very bioaccumulative properties [213]. Each of the six stabilizer groups listed (tin, organophosphite, hindered phenol, benzophenone, benzotriazole, and “other”) had high hazard scores on at least one of the above three criteria [213].

Many stabilizers are additionally used in consumer products other than plastic (e.g., in household and personal care products) or have other major sources of human and environmental exposure.

###### Light stabilizers

Light stabilizers, also called UV stabilizers or photo stabilizers, may be added to plastics either to protect the plastic itself or—when used in plastic packaging materials—to protect goods within plastic packaging against photodegradation. Major classes of UV stabilizers include benzophenones (BzPs) and benzotriazoles.

BzPs act as UV absorbers, with widespread use in sunscreens, cosmetics, and other personal care products [215,216], and are added to plastics and textiles to protect them from photodegradation [216,217].

BzPs can readily penetrate the skin and therefore be easily absorbed [1126]. A recent experimental study in humans showed that cotton clothing that had been exposed to elevated concentrations of BzP-3 in the air (4.4 microg/m^3^) for 32 days acted as an exposure route [1127]. After wearing the clothing for three hours, both the parent BzP-3 and its metabolite BzP-1 were detected in urine [1127].

BzPs are also released from food packaging materials [223,457], and exposure has been linked to dietary sources such as frozen food, instant noodles, and instant coffee in a 2016 Korean study, with urinary concentrations being higher in younger cosmetics users and lean women [1128].

BzPs in personal care products have long been recognized as causing allergic contact dermatitis, with BzP-3 being the most common cause [215]. Systemic health impacts associated with exposure to BzPs in human epidemiological studies include endometriosis, renal function, oxidative stress markers in pregnant women, and disordered emotional-behavioral development in preschool children following prenatal exposure [1126,1129]. BzP-3 exposure is associated with decreased birth weight in girls and increased birth weight in boys; in addition, gestational age in both boys and girls is reduced [1130,1131].

Benzotriazoles are another class of high-volume chemicals used as UV stabilizers. They have been shown to present in plastic bottle caps, food packaging, and shopping bags [221]. Benzotriazoles are highly lipophilic, bioaccumulative, and persistent in the environment and have been detected in human blood, breast milk, and urine [221], including urine from the general population of seven countries (China, Greece, India, Japan, Korea, Vietnam, and the US) [220].

The human health effects of benzotriazoles have been investigated in only one cohort, which reported associations between three benzothiazone stabilizers (1-H-benzotriazole, tolytriazole, and xylyltriazole) and elevated estrogens and testosterone in the urine of pregnant women [1132]. In the same cohort, Chen et al. report association between benzothiazones and cord blood mitochondrial DNA copy number [1133].

Some benzothiazones appear to be endocrine disruptors that activate the aryl hydrocarbon receptor [1134,1135], and/or exhibit anti-androgenic, estrogenic, or anti-estrogenic activity at human sex hormone receptors [221,1136].

In January 2021, the benzothiazone UV-328 was found to fulfill all persistent organic pollutant (POP) criteria under the Stockholm Convention, and in September 2022, the POPs Review Committee to the Stockholm Convention recommended that UV-328 be listed in Annex A to the Convention [222].

Other light stabilizers include carbon black (a UV absorber, with additional application as a pigment and filler in black plastics) [179]; triazine UV absorbers [224]; metal chelate UV quenchers, such as nickel chelates [179,225]; and hindered amine quenchers [179,225]. Eleven triazine UV stabilizers have recently been detected in human breast milk alongside benzotriazoles and BzPs [226], in addition to detection in household dust and air. Except for carbon black, which is a known human carcinogen, the health effects of these compounds have been little studied [223,457].

###### Antioxidants

Classes of antioxidants in plastics include hindered phenols [179,227], aromatic secondary amines [179,228], and organosulfur compounds (thioethers) [179,231].

Hindered phenol antioxidants are primary antioxidants that act as hydrogen donors and may be used alongside secondary antioxidants [179]. They include butylated hydroxytoluene (BHT), which is used in a large number of products, including food, cosmetics, pharmaceuticals, and fuels as well as plastics and rubbers [232]. Human exposure is primarily via diet and also occupationally via inhalation in workers handling BHT [1137]. Its metabolite (BHT-acid) has been detected in 98% of samples in the German Environmental Specimen Bank, with median levels being slightly higher in women than men [232], and in 78% of samples in a study of urine samples from China, India, Japan, Saudi Arabia, and the US [1138].

Toxicological studies of BHT have reported carcinogenicity and reproductive toxicity [1138] as well as immunosuppression when administered to rats along with other food preservatives [1139]. BHT administration in pregnant mice resulted in maternal weight loss, reduced implantation sites, and failure of the uterine lumen to close [1140]. Endometrial decidual markers were reduced along with upregulation of serum estrogen, progesterone, estrogen-receptor-a, and the progesterone receptor [1140]. There are fewer data available on other hindered phenols. However, 9 of 14 hindered phenols listed as antioxidant plastic additives in the ECHA mapping exercise have been shown to migrate from food contact plastics [223,457]. Epidemiologic studies of the possible adverse health effects of BHT are lacking.

Despite reports of adverse health effects in toxicological studies, and the lack of epidemiological data regarding the safety of population-wide exposure, BHT is approved as a food additive in the US up to concentrations of 0.02% (w/w) of total lipid content of food [1141].

Organophosphite antioxidants are secondary antioxidants that decompose hydroperoxides released by oxidative processes to unreactive products, but they are themselves converted to OPEs in that process [1142]. They have an additional role as heat stabilizers [214]. Both organophosphite additives and their OPE products have been detected in indoor dust and have thus been recognized as an important novel source of OPEs [1142]. Tris(nonylphenyl)phosphite, an organophosphite antioxidant used in plastic food packaging and demonstrated to leach from those materials [223,457], can a be hydrolyzed (in the presence of acids within packaged food or by gastric acid following ingestion) to nonylphenol [1143,1144], which are known endocrine disruptors [1145].

Other antioxidants used in plastics include aromatic secondary amines (primary antioxidants) [179,228] and thioesters (secondary antioxidants) [179,231], including bis(4-(2,4,4-trimethylpentan-2-yl)phenyl)amine, distearyl thiodipropionate, and di-octadecyl-disulfide, listed in the ECHA mapping exercise [214]. We did not find any human exposure or health data for these three chemicals.

###### Heat stabilizers

Heavy metal stabilizers are especially used in PVC, which has particular sensitivity to heat [179]. These include organometallic compounds containing lead, cadmium, or tin [179].

Lead in the form of lead salts has a long history of use as a PVC stabilizer. Concerns about widespread leaching of lead and its toxicity have triggered a proposal under REACH to restrict PVC products containing equal to or greater than 0.1% lead in the EU [1146]. Nevertheless, legacy plastic products, such as PVC piping, children’s toys, and recycled plastics, are still a major concern as sources of environmental lead and other metal contamination [1147,1148,1149]. High concentrations of lead have also continued to be detected in children’s plastic toys in a number of countries in Asia, Africa, and South America [1150].

Lead damages human health at even the lowest levels of exposure; the WHO has determined that no level of lead in blood is safe [1151,1152,1153]. Lead is toxic to multiple organs in children, most notably the brain and nervous system [1151]. It is additionally associated with hypertension in children [1154,1155,1156], and in adults, increasing evidence indicates that lead is an important risk factor for cardiovascular and renal disease [1157].

Cadmium (stearate or laurate) was previously used to stabilize PVC conferring heat stability and weatherability. The use of cadmium salts as stabilizers in plastics is now minimal and was less than 1% of total consumption in 2009 [1158,1159]. Cadmium was also used as a pigment in plastics, particularly for brightly colored products such as toys, and in ceramics and paint [1160].

Cadmium is toxic at low levels, and systematic reviews have documented associations between cadmium exposure and increased all-cause mortality, cancer mortality, and cardiovascular disease [1161,1162].

Organotins remain widely used as heat stabilizers and are used also to make antifouling paint for ships [179]. They are dispersed extensively in the environment, especially the ocean, and in humans [236,1163]. Dietary intake is considered the main route for human exposure [1163,1164].

Organotins are associated with endocrine and metabolic disturbances, including appetite regulation in toxicological studies [1165,1166]. Animal and in vitro human studies have revealed the association of organotins with oxidative stress and inflammatory responses [1167]. Organotins have been shown to affect fertility and reproductive function [1168], and in human infants, placental organotin concentrations are associated with increased risk of congenital cryptorchidism [1169].

As far as other heat stabilizers, nonmetallic organic stabilizers are increasingly used in place of metal stabilizers due to environmental and health concerns [179]. These are typically based on phosphites [179,214]. Phosphite esters are discussed in the “Antioxidants” section above.

##### Melamine

Melamine-formaldehyde resins, commonly referred to as “melamine,” are thermoset aminoplast plastics produced as copolymers of melamine and formaldehyde, sometimes blended with urea-formaldehyde resins. Key consumer applications include tableware, laminate surfaces on furniture, and inside coatings of food cans [1170]. Degradation of melamine-formaldehyde resin leads to the release of melamine and formaldehyde [1171,1172], and in some instances cyanuric acid [1173].

The human health impacts of high-dose, acute exposure to melamine were seen in a tragic episode of adulteration of infant milk formula with melamine in China. The main clinical consequences were renal stones and associated renal damage in young children [1174,1175].

Subsequent research has shown that lower-level exposures to melamine in the general population through melamine-formaldehyde resins may be associated with broader health impacts [1176,1177,1178], notably more renal toxicity. In a series of Taiwanese studies, melamine exposure was associated with renal stone formation (urolithiasis) in adults [1179], increased renal damage in patients with urolithiasis [1180,1181], and progressive decline in renal function in patients with chronic kidney disease [1182]. Studies in the US indicate that melamine exposure levels in the general adult [1183,1184] and child [1184,1185] populations are similar or higher than those seen in the Taiwanese studies and approach the levels seen in melamine factory workers in Taiwan [1186]. These general population studies find evidence of association between urinary melamine and diminished renal function in adults [1183] and between urinary melamine and cyanuric acid levels and markers of renal damage in children [1185].

#### Health impacts of microplastic and nanoplastic particles (MNPs)

Fragmentation of plastic through use or following disposal leads to the production and release into the environment of millions of MP particles (1–5,000 µm in diameter) [2,1187], which can further degrade over time into NPs; <1 µm in diameter [528,1188]. Additionally, there are primary MNPs, materials that are intentionally manufactured at micro or nano size for use in domestic and biomedical products, and these materials can also leak into the environment [1189,1190,1191]. MNPs are ubiquitously present in the environment today and have been detected in air [1192,1193], soil [271], and water supplies [1194,1195,1196]. However, knowledge of the safety of MNPs is limited by the fact that most experimental studies use standard particles that are most often PS, spherical, and of a known size, whereas environmental MNPs to which humans are exposed are highly diverse in terms of the type of plastic and additives therein, as well as their size, shape, and adsorbed chemicals [171].

##### MNP exposure

Humans are exposed to MNPs via multiple routes [780,1197,1198], with ingestion and inhalation being the main pathways [393,780]. Exposure to MNPs and their associated chemicals and pathogens can occur by direct ingestion and inhalation or via consumption of contaminated food products, such as fish [1199,1200] and wheat [1201] (see Table 3.2).

###### MNP ingestion exposure

Ingestion is a major route of human MNP exposure [788]. Humans are exposed to MNPs via consumption of contaminated food products, such as fruits and vegetables [1202], seafood [1203,1204,1205] (see Section 3), table salts [1206,1207,1208], drinking water [1209,1210], and other daily consumables [1208,1211], or consumption of drinks containing MNPs leached from plastic products, such as plastic bottles [1212,1213], tea bags [1214], and coffee cups [1215], during use. Additional exposure can occur via accidental ingestion of personal care products containing MNPs, such as toothpaste [1203,1208,1216].

There is a broad range of uncertainty in current estimates of human MNP exposure via ingestion [1217]. Estimates of typical exposures range from a high of 0.1–5 g/week (average exposure via ingestion only) [1218] to a low as 4.1 µg/week (median exposure in adults via both ingestion and inhalation) [530]. Key challenges to exposure estimation include combining data across studies based on mass with those based on count and heterogeneity in the polymers measured, technique used, and range of particle sizes reported upon.

Once ingested, MNPs travel through the digestive tract. They can accumulate within the gut, be excreted, or be absorbed. MPs have been detected in human colonic mucosal samples [1219], and several studies have reported MNPs in human stools [1203,1208,1220,1221,1222].

MNPs can be absorbed from the digestive tract via several pathways, depending on their size, including endocytosis by epithelial cells, transcytosis via M-cells or across tight junctions of the epithelial cell layer, and persorption via epithelial gaps formed after shedding of enterocytes from villous tips [1187,1223], with estimates of uptake of spherical PS MNPs being 10% for 60 nm MNPs after five days of daily oral gavage in rats [1224] and 6.16%, 1.53%, and 0.46% for MNPs of sizes 50, 500, and 5000 nm, respectively, 24 hours after a single oral gavage in mice [1035]. Local effects of MNP on gut permeability may also increase uptake. MNP-induced oxidative stress has been shown to cause intestinal epithelial cell apoptosis and increased gut permeability in mice, leading to increased MNP absorption and biodistribution in several organs [1035].

###### MNP inhalation exposure

Inhalation of MNPs released to indoor and outdoor air during the manufacture and use of products such as synthetic textiles, synthetic rubber tires, paint, and plastic covers is a second important exposure route for systemic uptake [393,780]. In evaluation of airborne particles, it is those with an aerodynamic diameter of less than 10 μm, and especially those of less than 2.5 μm, that are most likely be inhaled into lower airways rather than deposited in upper airways and ultimately swallowed [1225], although microfibers of up to 250 μm in size have been found in human lungs [1226]. Therefore, depending on their size and shape, once inhaled, MNPs can be trapped by the lungs’ lining fluid and be expelled with sputum or swallowed, or they can be deposited deeper in the lungs [780]. Upon contact with the alveolar epithelium, they can translocate into epithelial cells and macrophages via diffusion, direct cellular penetration, or active cellular uptake [780].

Humans are estimated to inhale between 26 and 170 airborne MNPs of varying sizes per day [255,783]. In humans, MNPs of multiple sizes and shapes have been detected in the sputum [1227] and bronchoalveolar lavage fluid [1228] as well as lung tissue obtained from surgical resections [1226,1229,1230] and autopsies [393]. The abundance of MNPs in human lungs has been shown to increase with age [1228,1229].

###### MNP dermal exposure

Dermal contact is another potential route of MNP exposure. Dermal exposure can occur via MNPs present in indoor dust, microfibers from textiles [1197,1231], and in a range of personal care and cosmetic products, such as face and body scrubs [1232,1233,1234,1235], shower gels [1236], and shampoo [427].

Dermal contact is considered the least significant route of MNP exposure in terms of potential for systemic uptake [1237] because absorption via skin is believed to be minimal for MNPs greater than 10–100 nm in diameter [1238]. PS NPs (≥20 nm) have been shown to accumulate in hair follicles of pig skin [1239,1240] and only penetrate the outermost layer of pig skin, even with a modestly compromised skin barrier [1240]. In contrast, studies using ex vivo human skin have shown that 750–6,000 nm PS MNPs can penetrate the skin via the hair follicles [1241]. Rare events of deeper penetration of smaller PS MNPs (20–200 nm) into viable epidermis at sites of high focal particle aggregation have also been detected in ex vivo human skin tissue [1242,1243].

###### MNP transplacental exposure

A route of exposure unique to infants is transplacental transfer of maternal MNPs during pregnancy, with a number of studies now reporting extensive detection of MNPs in human placental tissue following delivery [1244,1245,1246,1247]. Transplacental transfer is supported by detection of MNPs in human meconium samples [1244,1248] and by ex vivo human studies [1249,1250]. In vivo rodent studies have similarly confirmed the transfer of 20 nm rhodamine-labeled spherical PS MNPs from the maternal to the fetal circulation [1251].

###### MNP exposure risks for children

Infants are exposed to MNPs in infant feeding bottles [1252], infant formula [1253], and expressed breast milk stored in plastic containers [1253,1254]. Young children are exposed via age-specific behaviors, such as hand-to-mouth activity [251]. As a result, infant exposure to MNPs is likely to be greater than that of adults. Indeed, concentrations of MNPs reported in infant stools are an order of magnitude greater than in adults [1248]. The potential role of these early exposures in increasing risk for disease in later life is not yet known [1255,1256,1257].

###### MNP exposures via biomedical products and procedures

Several studies have detected ultra-high molecular weight PE MNPs in periprosthetic tissues in patients with joint replacements [771,1258,1259]. Veruva and colleagues [1260] showed strong correlations between the number of wear MNPs and macrophages and the level of inflammatory markers in periprosthetic tissue samples from patients with total disk replacement. Importantly, by inducing an inflammatory reaction [1261] mediated by macrophages [1262,1263], wear MNPs from prostheses can lead to periprosthetic osteolysis [1264,1265].

###### MNP toxicity

Studies of human health effects of MNPs are limited by the lack of a standardized method for quantifying MNP exposure, as they remain in early stages of development [1266,1267,1268]. As a consequence, most health evaluation is currently based on data from animal and in vitro studies [1269,1270], and we include that evidence here.

###### Gastrointestinal toxicity of ingested MNPs

Pathways for local toxicity of ingested MNPs in the gut include direct effects on gut epithelium and local uptake and indirect effects through actions in the lumen. A recent study found significantly higher concentrations of MNPs in stools of adults with inflammatory bowel disease compared to healthy individuals, and MNP concentration positively correlated with inflammatory bowel disease symptom severity [1271]. While these data are cross-sectional and reverse causality may be possible, there is mechanistic support from in vivo animal studies [1272], with MNPs ranging in shape and polymer type and size ranging from 0.1 μm to 5 mm shown to induce intestinal inflammation in a number of animal models (zebrafish, goldfish, and mice). In addition, in vitro studies have shown that exposure to MNPs can induce oxidative stress [1273], reduce mitochondrial membrane potential [1273], and reduce viability of Caco-2 human gut cells [1273,1274]. Overall, MNP exposure is suspected to be a digestive hazard to humans by adversely impacting the colon and small intestine, cell proliferation and cell death, chronic inflammation, and immunosuppression [1275].

MNPs can also have indirect effects on the gut through action within the lumen, with MNPs reported to induce gut microbial dysbiosis in mice [1276]. Similarly, in vitro experiments with Caco-2 human gut cells have shown that 30–140 μm PE MNPs altered the gut microbial population, which inhibited the following microbial metabolite pathways: the pentose phosphate metabolism pathway, which has an antioxidant effect in the gut; fructose and mannose metabolism, which may overload their absorption by the gut, leading to increased risk of obesity; and synthesis of secondary bile acids, which is crucial for GI function [1273].

###### Pulmonary toxicity of inhaled MNPs and microfibers

Synthetic fabrics, tires, and other fiber-based plastic products can release MP fibers to the environment through their use and disposal. MP fibers are also produced intentionally, and their manufacture can result in occupational exposures to production workers (see Section 2). Scientific evidence for adverse health effects of MP fibers is mainly related to exposures during textile manufacture [248].

Extensive studies of the toxicity of inhaled microfibers have been conducted in nylon flock workers. These studies are reviewed in the “Health impacts of synthetic textile manufacture” section. Health effects include elevated risks of a range of respiratory symptoms [1277,1278,1279], decreased lung function [1228], accumulation in pulmonary tissue [1230], and stomach and esophageal cancers [1280].

In vitro studies have shown that PS MNPs can be absorbed by human lung cells (25–100 nm) [1281,1282,1283] and can induce negative cytotoxic effects, including increased pro-inflammatory markers (25 nm–2.17 μm) [1282,1283,1284,1285], oxidative stress (40 nm–2.17 μm) [1281,1282,1284,1285], mitochondrial disruption (50 nm) [1282], genotoxicity (50 nm) [1281], altered cell morphology (1 μm and 10 μm) [1286], and decreased cell viability (25 nm–2.17 μm) [1282,1283,1284,1285,1287], cell proliferation (40 nm–10 μm) [1285,1286], and cell metabolic activity and cohesion between cells (1 μm and 10 μm) [1286]. In vivo rodent studies have shown that exposure to 64–535 nm PS microspheres increase pro-inflammatory markers [1288,1289] and that MNP exposure can cause pulmonary inflammatory cell infiltration, bronchoalveolar macrophage aggregation (1–5 µm commercial synthetic polymer microspheres) [1290], impaired lung function, and increased heart weight (0.10 μm PS microsphere) [1289].

###### Dermal toxicity of MNPs

In vitro human studies have shown internalization of fluorescently labeled PS MNPs (200 nm) by human keratinocytes and induction of several cytotoxic effects by PE MNPs (100–400 nm) and MNPs isolated from commercial face scrubs (30–300 nm), including oxidative stress, reduction in cell viability at high concentrations, and inhibition of cell proliferation [1291]. However, to date, there are no studies assessing human dermal exposure to MNPs and its potential health effects in vivo [1197].

###### Placental toxicity of MNPs

There have been few studies on health effects of transplacental MNP exposure. However, experimental studies in rodents have shown that maternal exposure to 5 μm spherical PS MNPs can permanently alter metabolism in the F1 and F2 generations, which increases their risk of metabolic disorder [1292,1293]. In humans, the presence of MNPs in the placenta have been associated with several ultrastructural alterations, such as narrowing of fetal capillary and changes in mitochondrial and endoplasmic reticulum morphology in human placenta [1246].

###### Systemic toxicity of MNPS

When they enter the body via any pathway, MNPs can be transported to all the organs through the circulation [1294,1295]. In mice, 50 nm and 500 nm spherical PS MNPs absorbed through the gut accumulated in the heart, liver, lungs, spleen, kidneys, testis, epididymis, and brain [1035]. In pregnant rats, 20 nm spherical PS MNP absorbed though the lungs accumulated in the maternal lungs, heart, and spleen and also translocated through the placenta to accumulate in the fetal heart, liver, lungs, kidneys, and brain [1251].

In humans, MNPs have been detected in several internal organs, including the para-aortic lymph nodes, liver, and spleen [771,1296]. Data on human tissue-specific effects following systemic MNP exposure is derived from in vitro human studies, with experimental MNP exposure aiming to model that which should enter systemic circulation [1297]. Key recent studies have shown that spherical PS MNPs can induce cytokine and chemokine production in mast cells and peripheral blood mononuclear cells (0.46–10 μm) [1298]; induce morphologic alterations, changes in gene expression, and apoptosis in microglia (0.2 μm and 2 μm) [1299]; and accumulate in human kidney proximal tubular epithelial cells, where they induce inflammation, mitochondrial dysfunction, endoplasmic reticulum stress, and autophagy (2 μm) [1300]. PE beads of 10–45 μm can have genotoxic effects in peripheral blood lymphocytes [1301], and several types of MNP beads can destabilize lipid membranes in red blood cells by mechanical stretching (0.8 μm PS, 1 μm and 10 μm PE, and 1 μm and 8 μm polymethylmethacrylate) [859].

In experimental animals, studies of MNP toxicity have generated abundant information [1302]. A recent systematic review reported induction of inflammation, oxidative stress, and several reproductive impacts of MNP exposure in rodents due to their accumulation in the ovaries and testes [1270]. For example, spherical PS MNPs disrupted the blood-testis barrier (38.92 nm–10 μm), induced testicular atrophy (38.92 nm and 100 nm), reduced sperm count (0.5–10 μm), increased ovarian collagen and fibronectin (0.5 μm), and impaired germ cell development in rodents (0.1–10 μm) [1270]. Indeed, a recent rapid systematic review concluded that MNPs may be a hazard to the human reproductive system [1275]. In addition to reproductive impacts, a recent scoping review by da Silva Brito and colleagues [863] reported metabolic and endocrine disruption, hepatotoxicity, and neurotoxic effects related to MNP exposure in rodents. Therefore, accumulation of MNPs in internal organs can potentially lead to serious health impacts.

#### Mechanisms of MNP toxicity

Potential mechanisms of MNP toxicity include effects due to physical properties such as a particle size and shape; effects related to chemical properties of the polymer; effects related to leaching of additives and other chemicals from MNPs; effects related to toxic chemicals that adsorb to the surfaces of MNPs; and effects related to pathogenic microbes that can adhere to the surfaces of MNPs to form a biofilm [20,641,752,1303].

##### MNP toxicity—Physical and material toxicity

Since the majority of MNPs originate from the breakdown of a wide range of larger plastics, they come in many different sizes and shapes (e.g., spheres, irregular, fibers) and are composed of many different polymers and additive chemicals [20,1037]. These factors determine the properties and bioavailability of individual MNPs [1037,1304] and therefore influence their toxicity (see systematic review [1305]).

MNPs appear able to exert direct toxicity related to their size, shape, and chemical composition. This direct toxicity is a relatively recently recognized phenomenon [20,1303]. It is separate from the toxicity that results from exposures to monomers and additive chemicals that leach from MNPs [861,1306].

Smaller [1307,1308,1309], irregular-shaped MNPs [861,1310] with sharp edges [1307,1310,1311] have been shown to have the highest toxicity in in vivo animal (e.g., decreased reproductive success and body length of *Daphnia magna*) and in vitro human studies (e.g., increased release of pro-inflammatory cytokine and ROS and reduced cell viability). The use of control particles in experimental studies has elucidated that the effects of MNPs (e.g., reproductive toxicity in *Daphnia magna* and antioxidant activity in mussels) are likely related to the plastic material itself and are not a result of particle exposure per se [1306,1309,1312].

The toxic effects of MNPs have additionally been found to be polymer dependent. PVC MNPs showed the greatest particle toxicity in an in vivo study in *Daphnia magna* (highest reproductive toxicity) [1306] and in an in vitro study using primary human monocyte and monocyte-derived dendritic cells (greatest increase in pro-inflammatory cytokine release) [861]. Importantly, this effect of polymer has been found to be independent of leached chemicals, with an independent effect of certain leached chemicals also demonstrated [861,1306].

An observation of possible relevance to understanding the direct toxicity of MNPs is that metal(-oxide) nanoparticles, similar in size to NPs, have been shown to target the central nervous system. In several animal studies, these very small particles were found able to cross the blood-brain barrier and enter the brain through olfactory nerve endings, resulting in altered neurotransmitter levels, acetylcholinesterase inhibition, oxidative stress, neuroinflammation, cell damage, and death [1295].

##### MNPs as a vector for toxic additives and monomers

MNPs can cause toxicity by releasing toxic chemicals such as monomers, plasticizers, flame retardants, antioxidants, and UV stabilizers from their plastic matrix into cells, tissues, and body fluids [1313,1314]. This has been termed the “Trojan horse” effect [882,1036] (see Section 3), and although it may not play a major role in most habitats [1037], risks to human health from this source are unknown [811].

##### MNPs as a vector for environmental toxins and pathogens

MNPs can cause toxicity through their ability to adsorb toxic chemicals and pathogens from the surrounding environment and transport these materials into cells and tissues [1035,1223,1224,1225,1228]. Toxic chemicals detected on the surface of MNPs include POPs such as PCBs [818,1315,1316], PFAS [1317], and PAHs [507,818,1201,1315,1318,1319]. The adsorption capacity of MNPs depends on their surface area, with smaller particles having higher active surface area, and also on the polymer type for both environmental pollutants [818,1320,1321] and microorganisms [1322].

MNPs can also act as a vectors for microorganisms [272,1323,1324], including human pathogens [1325,1326] containing antibiotic resistance genes [1327,1328,1329]. Pathogenic bacteria such as *E. coli* have been found on plastic pellets on bathing beaches [1330,1331]. A recent human study showed that the presence of pathological microbes in bronchoalveolar lavage fluid was associated with higher MP concentrations [1228]. MNPs can thus play a role in facilitating the emergence of infectious diseases [1332].

### Health Impacts of Plastic Waste

An estimated 400 Mt of plastic waste are generated globally each year, and this volume is increasing in parallel with annual increases in plastic production, especially in the production of short-lived and single-use plastics. Less than 10% of plastic waste is recycled, in contrast to recycling rates of 65%–70% for paper and cardboard and 90% for glass [1333]. Most plastic waste is discarded in landfills, incinerated, or exported to LMICs, where it threatens the health of approximately two billion people [362]. A particularly hazardous component of plastic waste is electronic waste (e-waste).

#### Health impacts of landfilled plastic

Plastic discarded in landfills accumulates in enormous quantities, especially in LMICs [1334]. Mismanaged plastic waste that escapes from landfills clogs waterways and disfigures beaches. Plastic waste can also catch fire, exposing nearby residents to toxic combustion products. As it breaks down, plastic waste can generate MNPs and leach toxic additives into surface water and groundwater (see Section 2).

#### Health impacts of unmanaged plastic incineration

Combustion of plastic in open pits generates copious amounts of particulate air pollution as well as multiple hazardous air pollutants that include polychlorinated dioxins and dibenzofurans (PCDD/Fs), PCBs, and heavy metals. In Indian cities, the burning of waste and plastic accounts for 13.5% of all PM_2.5_ air pollution and is linked to an estimated 5.1% of lung cancer cases (total 5,000 per million population) or 255 cases per million [367].

#### Health impacts of “waste-to-energy”

Thermal conversion, or pyrolysis, of waste plastic via incineration in waste-to-energy facilities results in the generation of a wide range of hazardous chemicals, including chlorine, hydrogen chloride, and phosgene (mustard gas); hydrogen cyanide; and ammonia as well as formic acid, formaldehyde, benzene and its derivatives, phenol, and PCDD/Fs [321]. The main sources of these toxic combustion chemicals are PVC and condensation polymers such as PURs, PA, and phenyl-formaldehyde resins.

Human exposure to toxic chemicals produced by thermal conversion of waste plastic occurs through dietary intake, inhalation of contaminated air, soil and dust ingestion [1335], and dermal contact [1336], with dietary intake accounting for 60%–99% of total PCDD/F intake [1336].

- PCDD/Fs ingested through agricultural products have been linked to endocrine disruption, damage to the immune system, reproductive disorders, neurodevelopmental disorders, and physical growth deficits in “fenceline” communities surrounding an e-waste recycling facility in Vietnam [1337]. PCB and PBDE exposures in communities near this facility were implicated in cognitive deficits and genotoxicity [1337].
- PCB, PCDDs, and PBDE exposures have been correlated with changes in thyroid function, weight loss, skin and mucosal pigmentation, periorbital edema, gingival hyperplasia, and abnormal skull calcification in children living near a computer e-waste recycling site in China [1338].
- Airborne VOC exposures near a plastic reprocessing plant in Japan were associated with a significantly increased prevalence of mucocutaneous and respiratory symptoms, including sore throat, eye discharge, eye itch, eczema, and sputum [1339].
- In Greece, a fire in a plastic recycling plant resulted in an increase in PCDD/F levels in the surrounding air, which was associated with a 13% increase in 30-year cancer risk, and even higher lifetime cancer risk, in neonates exposed in utero or viabreastmilk [1340].

#### Electronic waste (e-waste)

Electronic waste comprises a significant portion of discarded plastic (see Section 2). Over 50 Mt of e-waste are generated annually, most in high-income countries. E-waste consists of discarded computers, mobile phones, televisions, and appliances and contains great quantities of plastic. Planned obsolescence is a major driver. Only 17.4% of discarded electronics is recycled, and 7%–20% is exported to LMICs [1341], where it accumulates in enormous deposits, such as those in Agbogbloshie, Ghana [1342]; Latin America; and various locations in south China [1343]. The WHO reports that more than 18 million children, some as young as five years old, are employed in e-waste recycling, where they are exposed to lead, mercury, and PCDDs under horrificconditions [1344].

Plastics commonly used in electronics manufacture and found in e-waste are acrylonitrile butadiene styrene, PS, PC, PVC, PE, and PP. In addition, more than 200 different types of flame retardants are used in electronics production and can constitute 15% by weight of electronic products. These flame retardants can be chlorinated or brominated, phosphorus-based, and aluminum trihydrate and its derived inorganic trihydrates. Metals, either in plastic or from other sources, are also present [348].

Associations between e-waste exposures and compromised thyroid function have been reported, although results were inconsistent. Exposure to metals in e-waste was associated with lower forced vital capacity in eight- and 9-year-old boys. Pregnancy outcomes included consistent associations with increased spontaneous abortions, stillbirths, and premature births as well as reduced birth weight and birth length. Physical growth (i.e., height, weight, and body mass index) was also stunted. Increased lead levels in blood were associated with low scores on neonatal behavioral neurological assessment. Increased frequencies of DNA damage and micronucleated and binucleated cells were also seen in peripheralblood [346].

### Harms to Human Health of Climate Change Caused by Plastics

Plastic is a contributor to climate change [479,480,481]. In 2019, global greenhouse gas (GHG) emissions across the plastics life cycle were estimated to be 1.8 Gt of CO_2_e, approximately 3.7% of current global GHG emissions [5]. The greatest fraction of these emissions arises in plastic production. GHG emissions from plastic are projected to increase to 4.3 Gt CO_2_e by2060 [14].

Myriad catastrophic environmental events are associated with climate change, and all have potential to significantly impact human health [307]. They include the direct effects of heat and extreme weather conditions and events; indirect ecosystem-mediated effects, such as expanded ranges of disease vectors [1345,1346] and effects on food systems; and effects mediated by socioeconomic pathways, such as increased poverty, sociopolitical tension and/or conflict, and population displacement [1347]. The WHO considers climate change the gravest threat to human health in the 21st century, having the potential to undo all the progress made over the past 50 years in human development, global health, and povertyreduction [307].

Studies investigating the human health impacts of climate change have increased exponentially in number [1347] and unequivocally indicate that climate change has deleterious impacts on mortality; infectious diseases; respiratory, cardiovascular, and neurological illnesses and diseases; mental health; nutrition; pregnancy and birth outcomes; skin diseases and allergies; occupational health and injuries; and health systems [1345,1348,1349]. Despite these great gains in knowledge, critical gaps remain in the evidence base, and further investigation is required [1345,1347].

The effects of climate change disproportionately impact populations that have contributed least to the problem [1346], and they particularly threaten society’s most vulnerable and disadvantaged groups. These include women and children, older adults, individuals with underlying health conditions, poor communities and low-income countries, ethnic minorities and Indigenous populations, and migrants and displaced persons [307,1350,1351].

Climate change has the potential to amplify the health impacts associated with plastic across its life cycle. For example, the increasing frequency and intensity of climate-related extreme weather events and flooding have the potential to exacerbate the spread of plastic in the natural environment [480] and to accelerate the spread of MNPs and the release into the environment of plastic additives [481]. Rising sea levels have the potential to increase the amount of ocean plastic pollution [1352]. Storms have the potential to damage warehouses that store raw materials and finished plastic products, thereby causing unintentional plastic leakage into the environment. Wildfires, which are occurring with increased frequency and severity [1353,1354], result in the combustion of plastic materials, especially when they reach urbanized environments. This, in turn, releases copious amounts of air pollutants, including PM, ozone, carbon monoxide, NO_x_, and VOVs [1354] as well as PCDDs and PCDFs s, mercury, and PCBs [1355]. Increasing ocean acidification, seawater salinity, and higher temperatures combined with UV light exposure accelerate the rate at which plastics fragment and release additives into the environment [513,1356,1357,1358,1359].

Plastic pollution is enhancing the spread of vector-borne diseases by increasing the number of sites available for mosquito breeding [1360]. Bacteria, including pathogenic bacteria, can colonize MPs in the sea [1325,1361,1362] and may travel vast distances, expanding their geographic range [1363]. Rising sea surface temperatures have been found to increase the virulence and capacity for the marine pathogen *Vibrio parahaemolyticus* to adhere and form biofilms onplastics [1364].

### Conclusion

Plastic causes disease, disability, and premature death at every stage of its long and complex life cycle—from extraction of the coal, oil, and gas that are its main feedstocks; to transport, manufacture, refining, use, recycling, and combustion; and finally to reuse, recycling, and disposal into the environment. In this section of the Minderoo-Monaco Commission on Plastics and Human Health, we have summarized current information on the nature and magnitude of these hazards and also identified gaps in knowledge where additional research is needed to characterize and quantify plastics’ risks to human health.

At every stage of the plastic life cycle, infants in the womb and children are the populations at highest risk. Measures taken to protect the health of children and other highly vulnerable groups, such as workers, residents of “fenceline” communities, and Indigenous populations, are ethically and morally well justified and have the added benefit of protecting the health of entire populations.

## Section 5—Quantifying the Health-Related Economic Costs of Plastic Production, Use and Disposal

### Introduction

The harms to human health and the environment caused by the production, use, and disposal of plastics impose significant economic costs on individuals, society, and governments. These include costs of illness; productivity losses resulting from disease, disability, and premature death; and costs resulting from damages to ecosystems.

The goal of this section is to quantify the burden of disease and estimate the health-related costs associated with the production, use, and disposal of plastics. To undertake these analyses, we relied on three data elements: data on exposures to plastic-related hazards in at-risk populations; data on incidence and prevalence of plastic-related health outcomes in these populations; and dose-response functions relating plastic-associated hazards to adverse health outcomes.

**Figure 5.1:**
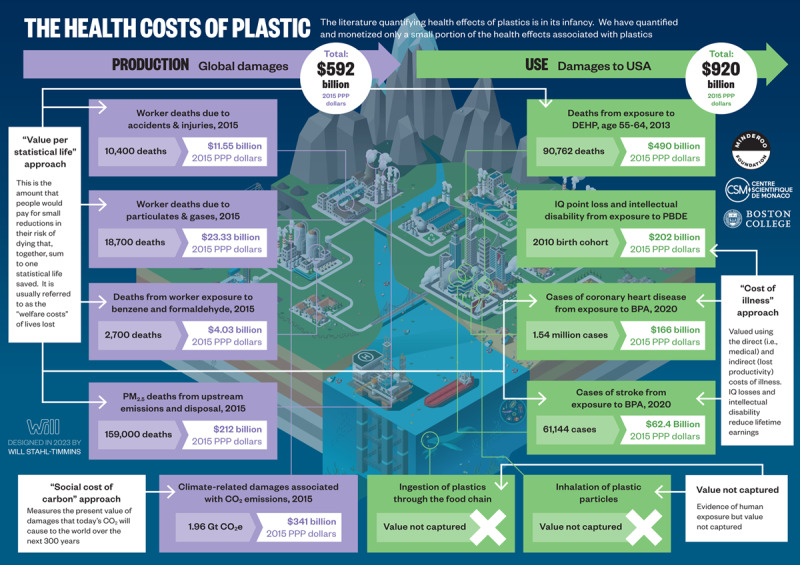
**Health costs of plastic.** Plastic causes significant harm to humans as well as the environment across all stages of its life cycle. Quantifying the human health disease burden and economic costs associated with plastic production, use, and disposal is a complex, and at times difficult, endeavor. Conducting high-quality epidemiological studies will greatly benefit this emerging field of research. PPP, purchasing power parity; PM_2.5_, particulate matter with a diameter of 2.5 micrometers or less; CO_2_, carbon dioxide; Gt, Gigatons; CO_2_e, carbon dioxide equivalent; DEHP, di(2-ethylhexyl) phthalate; PBDE, polybrominated diphenyl ether; BPA, bisphenol A. *Credit*: Designed in 2022 by Will Stahl-Timmins.

We first estimate the costs of health impacts associated with plastic production—for example, the costs of occupational disease and premature death in workers producing plastics, and the health-related costs of air pollution exposure in communities adjacent to oil and gas extraction and plastic production facilities. Next, we estimate some of the health-related costs associated with plastic disposal. We also present estimates of the costs of mortality and other impacts associated with emissions of CO_2_ and other GHGs from plastics production (Figure 5.1).

Estimating the disease burden and the economic costs associated with plastics use is more challenging. Most of this disease burden appears to result from exposures to plastic additive chemicals, and the epidemiological literature on these additives, many of which are endocrine disruptors, carcinogens, and neurotoxicants, is still in its infancy, although rapidly expanding. Estimates of population exposures to these chemicals exist primarily for countries in Europe and North America. We are therefore able to estimate and value the health impacts of some EDCs and neurotoxic chemicals found in plastics for these countries but not for the rest of the world.

We sought additionally to quantify the burden and costs of disease and death caused by MNP particles. Epidemiologic studies of morbidity and mortality associated with MNP exposures are, however, rare, due to limited measurement of these particles in the general population. We therefore do not quantify or value these health effects.

We note that the economic costs of harms to human health and the environment caused by plastics are often not borne by plastics manufacturers or fossil fuel companies. Instead, most of these costs are externalized and are borne by governments, businesses, and individual citizens, locally as well as globally.

### Health Costs of Plastics Production, use and Disposal

#### Health costs of local air pollution

Fossil fuels provide the feedstock used to make plastics and are also the main source of the energy used in plastics production. Plastics production is highly energy-intensive: 87% of fossil fuels used in plastics production are combusted [13], resulting in air pollution emissions (PM_2.5_, NO_x_, and SO_x_) as well as in the generation of GHGs. Plastics disposal (e.g., incineration, pyrolysis, and landfilling) results in additional air pollution and GHG emissions. Multi-regional input-output models have made it possible to trace the location of plastics production facilities and associated fuel use from the extraction of fossil carbon, through their combustion in the production process, and in the incineration and landfilling of plastics.

The local air pollution impacts of plastics production are greatest in the regions and countries where fossil fuel extraction and transportation, resin production, and manufacturing are concentrated, often in low-income countries. Table 5.1 presents estimates of the workforce engaged in plastics production and disposal in 2015 by geographic location and stage of production. Seventy-five percent of the global plastics workforce in 2015 were located in Asia—41% in China and 35% in the rest of Asia. Only 5% were located in the EU and 2% in the US. The other four areas listed—the rest of the Americas, the rest of Europe, Africa, and the Middle East each accounted for no more than 6% of the global plastics workforce.

**Table 5.1:** **Global workforce in employed plastic production (unit: 1,000 Full Time Equivalents).** RoW refers to rest of world.


	UPSTREAM CHAIN	RESIN PRODUCTION	MANUFACTURING	RECYCLING	INCINERA-TION	LAND-FILLING	TOTAL WORKFORCE	TOTAL (UNIT: %)

EU	4,090	1,184	755	199	73	28	6,329	5.79%

US	1,950	438	37	48	33	21	2,527	2.31%

China	31,300	11,900	63	1,090	8	45	44,405	40.65%

RoW Asia	24,600	12,262	517	40	36	23	37,478	34.31%

RoW Americas	3,527	2,626	263	7	7	130	6,560	6.01%

RoW Europe	3,308	1,265	654	30	25	251	5,533	5.07%

RoW Africa	4,732	571	129	0	29	28	5,490	5.03%

RoW Middle East	668	232	7	0	4	6	917	0.84%

Total	74,175	30,477	2,425	1,414	216	531	109,238	100.00%


*Source:* Cabernard *et al.*, 2022 and authors’ calculations.

The types of fossil fuels burned to produce plastics and associated emissions reflect the location of production. The world’s largest consumers of coal—China, followed by India and Indonesia—are also major plastic producers. Thus, in 2015 coal constituted 44% of fossil fuel used in plastics production globally followed by oil 40%, and natural gas 8%. The sulfur and ash content of coal, the sulfur content of oil, and the extent to which pollution control equipment is used in extraction and production processes determine the amounts of primary PM emitted. Emissions of SO_2_ and NO_x_ determine secondary particulate formation.

Cabernard et al. [13] provide estimates of exposure to ambient PM_2.5_ pollution and associated health effects throughout the plastics production process. Specifically, they estimate PM_2.5_ intake fractions [1365,1366] associated with the burning of different fuels used to produce (and dispose of) plastics, by country. Estimates of the number of deaths associated with PM_2.5_ exposures are based on concentration-response functions from the Global Burden of Disease study [970,1367,1368] and reflect deaths due to ischemic heart disease, stroke, chronic obstructive pulmonary disease, lower respiratory infection, and lung cancer among adults 30 years of age and older.

Mortality estimates associated with plastic, by country group for 2015, are presented in Table 5.2. Cabernard et al. [13] estimate that local air pollution from plastics production and disposal resulted in 159,000 deaths globally in 2015, of which 99% are associated with plastics production. Seventy-nine percent of these deaths occurred in Asia—31% in China. Only 6% occurred in the USA and the EU combined. Geographic patterns of these deaths reflect differences in ambient PM pollution levels associated with plastics production, differences in population density surrounding production facilities, and differences in baseline mortality associated with cardiorespiratory diseases of persons living near these fatalities. The overall pattern is, however, clear: deaths associated with ambient PM pollution from plastics production occur where production is taking place: primarily in low- and middle-income countries (LMICs).

**Table 5.2:** **Deaths attributable to ambient particulate matter (PM) air pollution resulting from plastics production and disposal.** RoW refers to rest of world.


	ENTIRE LIFE CYCLE OF PLASTICS	DISPOSAL (DIRECT IMPACTS OF INCINERATION AND LANDFILLING)	TOTAL (UNIT: %)

EU	6,474	49	4.06%

US	3,540	80	2.22%

China	48,900	629	30.66%

RoW Asia	76,512	59	47.97%

RoW Americas	6,290	11	3.94%

RoW Europe	4,216	264	2.64%

RoW Africa	8,700	33	5.45%

RoW Middle East	4,860	10	3.05%

Total	159,491	1,134	100.00%


*Source:* Cabernard *et al*. (2022), Cabernard (Personal Communication, 2022) and authors’ calculations.

#### Health impacts on workers

Workers involved in plastics production are exposed to a variety of hazards in addition to ambient PM_2.5_ pollution.^1^ These include toxic and carcinogenic chemicals used to produce plastics, such as benzene, formaldehyde, and vinyl chloride as well as gases and PM inside factories. These workers are also at high risk of occupational death and injury. The Global Burden of Disease study [1369] estimates occupational fatalities for various categories of hazards, by country. Those most relevant to plastics production include exposure to benzene, exposure to formaldehyde, exposures to PM and gases, and deaths due to injuries. To measure occupational deaths associated with plastics production, we assume deaths are related to the proportion of the workforce in plastics production. We multiply total worker fatalities in a category by the ratio of workers involved in making plastics to the size of the country’s workforce [1370].

Estimates of worker fatalities in plastics production in 2015 due to exposure to benzene, formaldehyde, injuries, PM, and gases are presented in Table 5.3. The largest category of fatalities are deaths due to PM and gases (18,713 globally in 2015), followed by deaths due to injuries (10,410). Occupational exposures to benzene were responsible for an estimated 1,705 deaths, and formaldehyde exposures for 1,030 deaths. The distribution of total worker deaths by region/country generally parallels the distribution of plastics workers in Table 5.1, but there are differences that reflect differences in workplace safety across countries and differences in the proportion of the workforce employed in plastics production.

**Table 5.3:** **Occupational deaths attributable to plastics production, global, 2015.** PM, particulate matter; RoW, rest of world.


	BENZENE	FORMALDEHYDE	INJURIES	PM AND GASES	TOTAL	TOTAL (UNIT: %)

EU	139	18	209	498	864	2.71%

US	96	12	114	261	483	1.52%

China	383	398	3,939	10,985	15,705	49.30%

RoW Asia	526	378	4,356	5,876	11,136	34.95%

RoW Americas	249	90	601	421	1,362	4.27%

RoW Europe	77	19	400	380	876	2.75%

RoW Africa	143	85	713	258	1,200	3.77%

RoW Middle East	91	31	77	34	233	0.73%

Total	1,705	1,030	10,410	18,713	31,857	100.00%


*Source:* GBD 2016 Occupational Risk Factors Collaborators (2020) and authors’ calculations.

It is important to emphasize that the data presented in Table 5.3 are almost certainly underestimates of the full burden of occupational deaths associated with plastics production. These estimates do not reflect deaths that occur after long latency periods, such as cancer deaths due to vinyl chloride monomer exposure, or cancers of long latency associated with carcinogen exposures in the extraction of fossil fuels.

#### Health and other impacts of plastic-related GHG emissions

In 2015, plastics production created 1.96 Gt of CO_2_ and other GHGs [13]—almost 2 Gt of CO_2_e. While representing only around 3.7% of total GHG emissions in 2015, it is not unreasonable to assume that a similar proportion of the GHG effects on climate and human health could be attributed to production of plastics. The health impacts of GHG emissions, which occur through their effects on the earth’s climate now and in the future, are numerous. They include impacts of temperature changes on mortality and morbidity due to cardiorespiratory disease (fewer hospitalizations or deaths in the winter, and more in the summer); impacts of changes in temperature and precipitation on disease and death from vector-borne diseases; effects of climate on health mediated through floods, droughts and food insecurity [1371,1372]. Among the best studied are the impacts of temperature on mortality [1373,1374,1375]. Estimating the health impacts of current CO_2_ emissions requires estimating their future impacts on the climate and the effects of climate changes on health. Because of the long residence times of CO_2_ and other GHGs in the atmosphere, both estimates require predicting what the world will look like—in terms of population, gross domestic product (GDP), and GHG emissions—over the span of many decades. Recent estimates of the social cost of carbon (SCC) [1376,1377] address this complexity. They reflect the anticipated impacts of CO_2_ emissions on changes in temperature and the impacts of temperature changes on mortality throughout the world from the present to the year 2300. These estimates suggest that increases in deaths during the summer will outweigh reductions in deaths during the winter.

The SCC measures not only the net impacts of temperature on mortality—it also includes estimates of the net impacts of temperature on agriculture, energy consumption and damages due to sea-level rise. In 2022, the US EPA [1376] released estimates of the SCC that include the present value of impacts for multiple categories of damages based on Rennert et al. [1377], the Climate Impact Lab [1378], and Howard and Sterner [1379]. The EPA’s estimate of the SCC corresponding to one ton of CO_2_ released in 2020 is $190 (2020 USD).

Using 2020 United States dollars (USD), the US Environmental Protection Agency estimates that each ton of CO_2_ released to the environment results in damages to human health and well-being (the social costs of carbon, SCC) that cause economic losses of $190 (USD).

#### Valuation of premature mortality associated with plastics production

Deaths associated with plastics production can be valued using the *human capital approach*—the value of output lost when a person dies prematurely—or using the *Value per Statistical Life* (VSL)—the amount that individuals will pay for small reductions in risk of death that together sum to one statistical life. We follow the World Bank [1380,1381] in valuing deaths associated with air pollution—in this case, deaths from plastics production—using the VSL. We refer to these as the “welfare costs” of premature deaths.

Because the deaths associated with plastics production occur throughout the world, we transfer estimates of the VSL from the OECD to individual countries using per capita Gross National Income (GNI) and an income elasticity of the VSL equal to one [1380,1382]. This is equivalent to using a VSL equal to 100 times per capita GNI of whichever country it is applied to. We express all damages in 2015 international dollars (Int$).^2^

The global welfare costs of the premature deaths associated with plastics production in 2015 were over $250 billion (2015 Int$)^3^ (Table 5.4). The welfare costs of ambient PM_2.5_ deaths (212 billion 2015 Int$) were 5.4 times greater than the welfare costs of occupational deaths (39 billion 2015 Int$), reflecting the fact that ambient PM_2.5_ deaths were five times as numerous as occupational deaths among workers in plastics production. Sixty-four percent of welfare costs occurred in Asia (35% in China). The EU and USA accounted for 20% of these costs.

**Table 5.4:** **Economic costs of deaths attributable to ambient particulate matter (PM) air pollution and occupational exposure resulting from plastics production, global, 2015.** PPP, purchasing power parity; RoW, rest of world.


	VALUE OF PM DEATHS (IN 2015 PPP BILLION)	VALUE OF OCCUPATIONAL DEATHS (IN 2015 PPP BILLION)	TOTAL VALUE (2015 PPP BILLION)	TOTAL (UNIT: %)

EU	24.42	3.11	27.54	10.98%

US	19.13	2.61	21.74	8.67%

China	66.30	21.29	87.59	34.94%

RoW Asia	64.94	8.03	72.98	29.11%

RoW Americas	8.76	1.60	10.36	4.13%

RoW Europe	8.15	1.32	9.47	3.78%

RoW Africa	8.22	0.47	8.69	3.47%

RoW Middle East	11.83	0.48	12.31	4.91%

Total	211.75	38.92	250.67	100.00%


*Source:* Authors’ calculations.

The welfare cost of the 1.96 Gt of CO_2_ emissions from plastics production in 2015 is $341 billion (2015 Int$), using the US EPA’s estimate of the SCC.

#### Valuation of health impacts of plastics use

Bisphenols, phthalates, brominated compounds, and other EDCs and neurotoxic chemicals are used in very large quantities in plastics production. These chemicals can leach out of plastics and can enter the human body through ingestion, inhalation, or dermal absorption. Bisphenols such as BPA are found in food packaging. PBDE is a flame retardant used in furniture and in children’s clothing that can be ingested from contaminated dust. DEHP, a phthalate, is used in industrial food processing and may be ingested in processed food. All of these chemicals have been found through epidemiological and toxicological studies (reviewed in Section 4) to be endocrine disruptors.

A growing epidemiological literature links endocrine disruptors to adverse reproductive outcomes, neurological development, morbidity, and mortality. Extrapolating these results to the general population requires estimates of population exposure to these chemicals. In the USA, the NHANES provides these estimates for some endocrine disruptors. Comparable data are available for Canada, and for some countries in the EU, but are not readily available for India or China. We therefore illustrate the impacts of three endocrine disruptors—PBDE, BPA, and DEHP—on health outcomes in Canada, the EU, and the US.

#### Impacts of PBDE on intellectual development

Longitudinal studies have linked PBDE levels in the blood of pregnant women to decreases in their children’s IQ [1110,1111,1123], finding that higher maternal levels of PBDE are associated with lower IQ scores in children, and negatively associated also with other neurodevelopmental and behavioral indices. Chen et al., Eskenazi et al., and Herbstman et al. [1110,1111,1123] all find statistically significant negative relationships between levels of PBDE congeners (BDE-47, BDE-100, and BDE-153) in mothers’ blood during pregnancy (indicating prenatal fetal exposure) and children’s IQ. In Chen et al. [1123], a one-unit increase in log_10_ BDE-47 is associated with a 4.5-point reduction in IQ at age five (CI = 0.1–8.8).

The results of these longitudinal studies have been used to estimate IQ losses in the 2010 birth cohorts in the US, the EU, and Canada (see Table 5.5). Attina et al. [1384] apply these results to the 2010 birth cohort in the US using the distribution of BDE-47 in the female population of child-bearing age from NHANES. They estimate that PBDE exposure resulted in a loss of almost 10 million IQ points based on Chen et al. [1123]. This number doubles using the dose-response results in Herbstman et al. [1110]. Bellanger et al. [1385] perform a similar analysis using estimates of PBDE in cord blood for the EU. PBDE concentrations are much lower in the EU and Canada than in the US due to bans on the use of PBDE in those countries, and result therefore in much smaller IQ losses than in the USA.^4^ In Canada, estimated IQ losses range from 347,000 to 927,000 points [1386].

**Table 5.5:** **Economic costs of IQ Loss and intellectual disability resulting from polybrominated diphenyl ether (PBDE) exposure in EU, USA, and Canada.** PPP, purchasing power parity; GDP, gross domestic product.


GEO GROUP	SCENARIO	IQ POINTS LOST	LOST ECONOMIC PRODUCTIVITY (IN 2015 PPP BILLION $)	ATTRIBUTABLE INTELLECTUAL DISABILITY	COST OF INTELLECTUAL DISABILITY (IN 2015 PPP BILLION $)	TOTAL COSTS (IN 2015 PPP BILLION $)	TOTAL COSTS (IN % OF GDP)

EU	PBDE (base)	837,685	8.30	3,156	2.75	11	0.06%

EU	PBDE (high)	1,926,158	19.09	7,705	6.72	26	0.13%

USA	PBDE (base)	9,818,493	145.77	43,268	56.51	202	1.11%

USA	PBDE (high)	19,035,078	282.61	98,769	129.00	412	2.26%

Canada	PBDE (base)	373,628	4.19	1,607	1.59	6	0.36%

Canada	PBDE (high)	926,055	10.40	4,493	4.44	15	0.93%


*Source:* Honeycutt *et al*. (2004); Bellanger *et al*. (2015); Attina *et al*. (2016); Malits, Naidu and Trasande (2022) and authors’ calculations.

Intellectual disability is defined by IQ levels below 70 points. Estimates of numbers of cases of intellectual disability associated with PBDE appear in Table 5.5. These range in number from over 43,000 in the US (base case) to 3,200 in the EU (base case) and 1,600 in Canada (base case).

We estimate the economic cost of lost IQ points in terms of foregone earnings over the lifespan of exposed children, and we estimate the cost of cases of intellectual disability using medical costs. There is a large literature linking IQ to earnings both directly and indirectly—i.e., by affecting educational attainment and labor force participation. We assume that one IQ point reduces the present value of lifetime earnings by 1.1% [1383], noting that recent studies have used values between 0.9% [1387] and 1.4% [1388]. Our estimate of lifetime earnings (Grosse, Krueger, and Mvundura [1383] include both market earnings and non-market output.) Using a discount rate of 3% and discounting lifetime earnings to birth yields a loss per IQ point of $14,847 2015 Int$ for the 2010 birth cohort in the US. This value is transferred to each country in the EU and to Canada based on the ratio of per capita GNI (2015 Int$) in the transfer country to per capita GNI (2015 Int$) in the US.

The present value of lost productivity associated with PBDE exposure for the 2010 birth cohort (base case) is $145 billion (2015 Int$) for the US, $8.3 billion (2015 Int$) for the EU and $4.2 billion (2015 Int$) for Canada (See Table 5.5) These numbers double using higher published estimates of IQ loss. We use estimates of the present value of the medical (direct and indirect) costs of intellectual disability for the US from Honeycutt et al. [1389], which we transfer to other countries using the ratio of per capita GNI (2015 Int$) in the transfer country to per capita GNI (2015 Int$) in the US. Adding these costs to IQ productivity losses yields economic costs of PBDE exposure to the 2010 birth cohort of $202 billion (2015 Int$) in the US—or 1.1% of GDP. Costs are significantly lower in Canada (0.36% of GDP) and the EU (0.06% of GDP), reflecting the lower levels of PBDE exposure in women in those countries.

#### Impacts of BPA on cardiovascular disease

Epidemiological studies over the past decade have explored associations between BPA and cardiovascular disease [1050,1390,1391,1392]. Three of these studies use data from multiple waves of the NHANES survey to link urinary BPA concentrations to various cardiovascular outcomes: coronary heart disease (CHD), ischemic heart disease, congestive heart failure and stroke. Cai et al. [1050], using a sample of 9,139 adults from the 2003–2014 waves of NHANES, find that the natural logarithm of BPA level (ng/mL) (ln BPA) in American adults is associated with increased prevalence of any cardiovascular diseases with an OR of 1.16 (CI: 1.04–1.29). Examining specific causes of deaths, ORs are 1.19 (CI: 1.02,1.39) for CHD and 1.20 (CI: 1.05, 1.37) for stroke. Moon et al. [1392] use waves 2003 through 2016 of NHANES to estimate impacts of ln BPA (ng/mL) on cardiovascular outcomes using Propensity Score Matching. They estimate ORs of 1.17 (CI: 1.08–1.27) for any cardiovascular diseases; 1.13 (CI: 1.04–1.24) for ischemic heart disease; and 1.13 (CI:1.01–1.26) for stroke.

We use the Propensity Score Matching results from Moon et al. [1392] together with the distribution of urinary BPA levels in the adult US population in the 2003–2016 waves of NHANES to calculate the fraction of CHD and stroke attributable to BPA in the US. We conservatively apply an OR of 1.13 to CHD and an OR of 1.13 to stroke. Our calculations indicate that 7.64% of the prevalence of each outcome is attributable to BPA exposure. Based on the prevalence of CHD (affecting 20.1 million adults in 2020) [1393] and stroke (affecting 795,000 adults in 2020) [1394], we estimate that 1.54 million cases of CHD and 60,738 cases of stroke in the US in 2020 were attributable to the plastic additive BPA.

We value the economic costs of these cases using estimates of the direct costs of illness from the American Heart Association [1395], and we value mortality attributable to BPA using the VSL used in previous analyses. Converting the medical costs of a case of CHD and a case of stroke to 2015 Int$ yields annual costs of $7.85 billion (2015 Int$) for cases of cardiovascular disease and $2.41 billion (2015 Int$) for cases of stroke associated with BPA. We value the 29,247 CHD deaths and 10,634 stroke deaths attributable to BPA exposure using a VSL of 5.4 million 2015 Int$. In the US, the total costs attributable to BPA are $165.9 billion 2015 Int$ for CHD and $62.4 billion 2015 Int$ for stroke.

#### Impacts of phthalates on mortality

Several recent studies in the US have investigated the relationship between phthalates and mortality [1271,1396]. Trasande, Bao, and Liu [1052] estimate the impact of phthalate metabolites, focusing on high-molecular weight metabolites and DEHP, on all-cause and cardiovascular mortality in the US. High-molecular weight phthalates are used in food wraps and intravenous tubing; DEHP is used in industrial food processing. Using data from the 2001–2010 waves of NHANES, the authors associate urinary phthalates with mortality data through 2015. Specifically, they estimate Cox proportional hazard models relating the natural logarithm of urinary phthalate concentrations to cardiovascular and all-cause mortality for 55- to 64-year-olds. They find that the natural logarithm of high-molecular weight phthalates is significantly related to all-cause mortality (OR = 1.14; CI: 1.06–1.23), as is the logarithm of DEHP (OR: 1.10 CI: 1.03–1.19). Applying the DEHP results to the 2013 cohort of 55- to 64-year-olds yields an estimated 90,762 deaths attributable to DEHP levels in 2013 or approximately 5.2% of all deaths in that year.

We value the 90,762 deaths using the same VSL as in previous analyses—5.4 million (2015 Int$). This implies a welfare cost of $490 billion (2015 Int$) for deaths attributable to DEHP exposure in the US in 2013.

### Conclusion

We have quantified some of the externalized costs associated with plastics production and use—costs that are not borne by the industries responsible for them, but instead are imposed on governments, businesses, and persons throughout the world, without compensation. The particular externalities on which we have focused are the health-related global impacts of pollution of ambient air and workplace air due to plastics production, and the impacts of endocrine disruptors and neurotoxicants associated with plastics use in the US. The value of these externalities is summarized in Table 5.6. The total cost of the health impacts of plastics production in 2015 that we are able to quantify is $250 billion (2015 Int$), more than the GDP of New Zealand or Finland in that year [1370].

**Table 5.6:** **Summary of disease burden and economic costs attributable to plastic production and use.** CO_2_, carbon dioxide; PPP, purchasing power parity; PM, particulate matter; PBDE, polybrominated diphenyl ether; CHD, coronary heart disease; BPA, bisphenol A; DEHP, di(2-ethylhexyl) phthalate; CO_2_e, carbon dioxide equivalent; Gt, Gigatons.


	DEATHS, CASES, IQ POINTS LOST OR CO_2_ EMISSIONS	VALUE (IN 2015 PPP BILLION)

**Upstream Health Effects**		

Global PM deaths in 2015	159,491 Deaths	211.8

Global Occupational deaths in 2015	31,857 Deaths	38.9

Sub-total	191,348 Deaths	250.7

**Health Effects of Plastics Use in the USA**		

Lost IQ Points (PBDE)	9,818,493 Points	145.8

Cases of Intellectual Disability (PBDE)	43,268	56.5

Cases of CHD (BPA)	1,540,000	165.9

Cases of Stroke (BPA)	60,738	62.4

Deaths (DEHP)	90,762	490.0

Sub-total	–	920.6

**Social Cost of Carbon – Plastics Production**		

Global CO_2_e emitted during plastics production	1.96 Gt CO_2_e	341.0


*Source:* Authors’ calculations.

The annual emissions of CO_2_ and other GHGs from plastics production, amounting to 1.96 Gt of CO_2_e in 2015, are more than the combined CO_2_ emissions of Brazil and Indonesia [1397]. Using the US EPA’s estimates of the SCC, we estimate that the annual cost of these emissions is $341 billion (2015 Int$).

To examine the health impacts of plastics use, we examined the disease burden and economic costs associated with EDCs and neurotoxic plastic additive chemicals, PBDE, BPA, and DEHP in the US. We limited this analysis to the USA because estimates of serum and urinary concentrations of these endocrine disruptors are available for the general US population and not available in most other countries. We estimate that the total cost of the health impacts due to these endocrine disruptors in the US in a single year is over $920 billion (2015 Int$). Estimates suggest that over 90% of exposure to these substances comes from plastics [1398].

What are the solutions to these externalized costs? Reducing local air pollution from plastics production, which relies heavily on the combustion of coal and oil, will require either a transition to clean, renewable energy or the use of pollution control devices to limit emissions of PM, NO_x_, and SO_2_. Reducing CO_2_ emissions will require moving from fossil fuel combustion to renewable sources of energy though various means, including economic incentives such as carbon trading rights, or through internalizing pollution costs through penalties and pollution taxes. If these policies were implemented, they would help reduce pollutant emissions from plastics production and from other manufacturing sectors.

The incorporation of hazardous chemicals into plastic production is part of the larger problem of inadequate regulation of chemicals in consumer goods. Differences in the regulation of chemicals in the EU vs. the US are illustrated by the differential impacts of PBDEs on intellectual disability and lost IQ points in Table 5.5. The losses in IQ points and associated lifetime productivity losses are much lower in the EU, where PBDEs have been regulated for many years, than in the US, where the impact of these endocrine disruptors remains substantial. Although we cannot produce similar estimates in other countries, due to lack of data on exposure of the general population, results from the US suggest that the externalized disease burden and economic costs associated with plastic use require much closer examination.

## Section 6—Social and Environmental Justice

### Introduction

**Figure 6.1:**
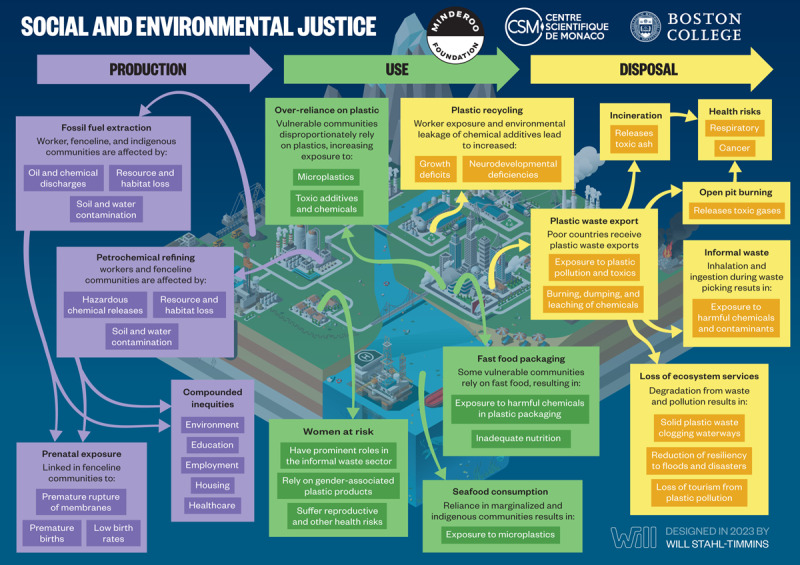
**The impact of plastic on social and environmental justice**. *Credit:* Designed in 2022 by Will Stahl-Timmins.

**Figure 6.2:**
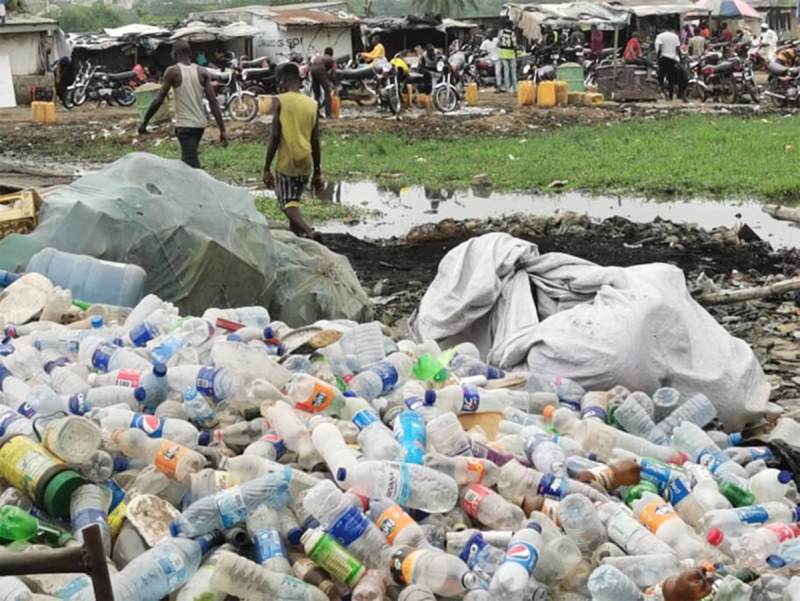
**Informal waste pickers – transit storage site in Ogun State, Nigeria**. *Credit:* Adetoun Mustapha and Korede Out.

Plastics’ health consequences fall disproportionately on the poor, minorities, the marginalized, and people in the Global South. Groups at especially high risk of disease, disability, and death caused by plastic, its feedstocks, its components, and its waste are people of color; Indigenous populations; fossil fuel extraction workers; chemical and plastic production workers; informal waste and recovery workers; persons living in “fenceline” communities adjacent to fossil fuel extraction, plastic production, and plastic waste facilities; and children (see, e.g., Figure 6.1 and Figure 6.2).

The purpose of this section of the Minderoo-Monaco Commission on Plastics and Human Health is to increase understanding of these disproportionate impacts and devise solutions that identify and address the needs of vulnerable groups. This requires looking at the problem of plastic pollution through the lenses of both social justice and the more specific issue of environmental justice (SEJ). See Box 6.1.

**Box 6.1 Definition of Social and Environmental Justice (SEJ).**

*Social justice* strives to fairly distribute resources, opportunities, and privileges in society, regardless of an individual’s background and status [1399]. *Environmental justice* is defined as follows:

The fair treatment and meaningful involvement of all people regardless of race, color, national origin, or income, with respect to the development, implementation, and enforcement of environmental laws, regulations, and policies [1400].

SEJ are inherently linked because issues in the environment have direct impacts on certain people and groups, including those living in or directly dependent on particular environments. When people do not have equitable access to resources that could relieve environmental stressors, it becomes a social justice issue. Both SEJ necessitate the following:

Cultural norms and values, rules, regulations, behaviors, policies, and decisions that support sustainable development, so that people can interact with confidence that their environment is safe, nurturing, and productive [1401].

SEJ addresses both “procedural” injustice, the unequal access to information and role in decision-making, and “distributive” injustice, which is the unequal distribution of burdens on certain groups and people [1402].

### Social and Environmental Justice

Social and environmental injustice flow from a belief, common in much of modern society, that “natural and human resources exist for exploitation, commodification and control, and to fuel economic growth.” [1403] Such beliefs and narratives are created and perpetuated by economic, political, and social groups “to concentrate power and wealth, which necessarily requires oppression of the masses and the marginalized.” [1403] They are grounded in the concept that some lives are more important than others. Those beliefs take no cognizance of the view that Earth is a shared inheritance, a “Common Home” whose “fruits are meant to benefit everyone.” [46]

The concepts of SEJ relate not only to the negative consequences of pollution but also to the exclusion of certain groups from participating “meaningfully in the leadership and composition of the environmental movement and related decision-making processes.” [1402] To reverse such injustice, SEJ focuses on the equitable distribution of both the environmental resources and burdens so that no one group of people bears a disproportionate share of the negative consequences resulting from industrial operations and/or government policies. SEJ may also consider equity and the rights of the environment [1404].

Until recently, less attention has been given to environmental justice in marine and coastal environments, where environmental harms, such as plastic and chemical pollution, are worsening and exceeding planetary boundaries through pollution and toxic waste, plastics and marine debris, climate change, fishery declines, ecosystem degradation, and biodiversity loss [1405,1406]. These concerns are now being framed as “ocean equity,” “ocean justice,” and “blue justice” and include the impacts of pollution and climate. The focus of this emerging area is the need to address equity and justice in ocean governance and management [1405,1406].

It is essential that any global agreement on plastic pollution, such as the Global Plastics Treaty [1407], address SEJ and that it include remedies to reduce the disproportionate impacts of plastics and related issues of climate change on coastal and ocean-dependent communities. These points are noted in Case Study 6.1, which focuses on the disproportionate impacts of plastic pollution and climate change on small island states. If solutions to the plastic crisis and the climate crisis are to be sustainable, they must benefit people and advance SEJ.

**Case Study 6.1 The Intersection of Climate Change and Plastics Pollution on Small Islands: An Amplifier of Social and Environmental Injustice.**

The intersection of plastics and climate change issues on small islands amplifies social and environmental injustice through complex linkages. Islands are on the front lines of climate change, experiencing extreme weather events, sea-level rise, brackish water inundation of freshwater sources, and other existential stressors [1408,1409,1410].

Island nations and states also experience substantial amounts of plastic washing up on their shores, including swaths of microplastics (MPs) [1410]. This pollution has negative implications for aesthetics, fisheries, and water quality. At the same time, island nations are most vulnerable to sea-level rise and will have to plan for managed retreat because of global climate change [1412,1413,1414].

Small island states contribute little to anthropogenic climate change yet are major recipients of its effects. The least developed countries contribute about 1% of carbon dioxide (CO_2_) emissions [1415], and similarly, the small islands classified as “small island developing states” by the United Nations (a mix of least developed countries and middle-income countries) contribute less than 1% of such emissions [1416,1417,1418]. In contrast, global plastics production, use, and disposal are sizable contributors to climate-driving greenhouse gas (GHG) emissions.

Plastic production relies almost exclusively on fossil fuel feedstocks, and by 2050, it is forecast to account for 20% of total oil consumption [73]; plastics-associated GHG emissions could reach 15% of the global carbon budget that same year [7,483]. Plastics products themselves emit GHG when exposed to sunlight [485], emphasizing the need for a life cycle approach to solutions [23].

Outcomes of plastics production, use, and disposal are growing threats to island communities, including to human health, at every stage of the plastics life cycle [9,1419]. Small Islands worldwide are disproportionately affected by plastics [1419,1420]. This inequitable burden results from a confluence of circumstances [1411].

For example, small islands serve as “strainers” of distal plastics concentrated and transported by ocean currents [1419]. These islands also have insufficient waste infrastructure to manage plastic waste generated locally [1421,1422] and experience plastic debris from all fisheries sectors (commercial, recreational/touristic, subsistence fishing, and aquaculture) [1411,1423]. The cultural and economic reliance on seafood in Oceania, including by marginalized or Indigenous communities, also poses a threat from MP pollution in seafood, particularly where dietary alternatives are not readily accessible [23,1424].

The intersection of climate change and plastic pollution is amplifying social and environmental injustice on small islands. Developing, implementing, and refining solutions to decrease social and environmental injustice on small islands can provide scalable solutions applicable to continental “islands” and ultimately our planet “island”—Earth. Such solutions are urgently needed by humanity to arrest the existential threat of climate change and address a less commonly recognized driver of GHG emissions: plastics production, use, and disposal.

### Plastic Pollution and SEJ

**Figure 6.3:**
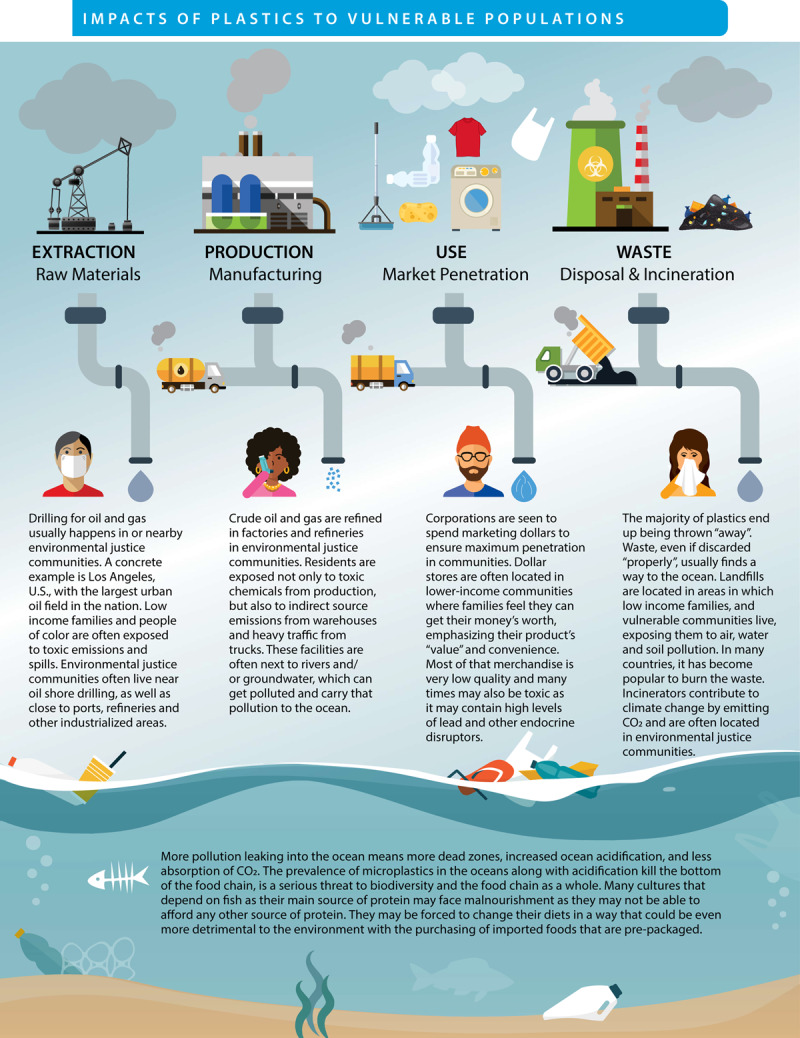
**Impacts of plastic to vulnerable populations.** As depicted in this figure from (UNEP and Azul, 2021), vulnerable groups and populations are adversely affected by plastic pollution (which includes intentional and unintentional leakage of plastic and chemical additives to the environment), throughout the entire life cycle of plastics, beginning with extraction and production, through its market penetration and uses, to plastic waste management and disposal. *Original source:* (UNEP and Azul, 2021).

Plastic-related pollutants intersect with SEJ across human health, environmental health, economics, and human rights [1402]. The nexus between these areas and SEJ is complex and tangled. As noted in Sections 2 and 3, plastic-related pollutants take many forms and are generated across the plastics life cycle, from fossil fuel extraction—the feedstock for virtually all plastic—production and manufacturing, to product use, and finally to intentional and unintentional dumping, littering, or unregulated disposal or discharge; managed disposal via landfills, incinerators, or chemical recycling facilities; and leakage from managed systems [23,64,1355,1425,1426] (see Figure 6.3).

The adverse effects of plastic pollution and climate change are widespread but currently felt most keenly in certain geographies and among certain groups and populations least responsible for the pollution who lack the power or resources needed to address the problem [1402,1427]. As a result, plastic pollution has been described as a new form of “colonialism.” [1428] Plastic pollution creates dire inequities for people, the environment, and the countries and prevents individuals and societies from flourishing [1427]. Environmental injustices occur at local, national, and global scales, including injustices occurring between the regions of the Global North and Global South: “Global North nations continue dumping waste in both domestic and global ‘pollution havens’ where the cost of doing business is much cheaper, regulation is virtually non-existent, and residents do not hold much formal political power.” [1429]

### Understanding the True Costs and Impacts of Plastics

Plastics are produced and manufactured inexpensively for the global market with harms and costs unaccounted for (or “externalized”), resulting in the diminished health and well-being of marginalized and vulnerable communities and the degradation of the environments and resources upon which these communities depend. The indirect social and environmental costs of plastics production and manufacturing are thus borne by poor or disempowered communities and peoples [7,1430,1431] and are not represented nor included in the costs paid by consumers. The full economic costs associated with plastic-related pollution include impaired ecosystems; reduction of their associated benefits, sometimes referred to as “ecosystem services”; and the still incompletely quantified costs of impacts on human health as noted by this Commission [1331,1432,1433]. These costs (referred to as “externalities”) are excluded from the plastic sector’s current accounting or responsibilities and shifted onto governments, taxpayers, and citizens without compensation; thus, they are disproportionately experienced by marginalized and vulnerable communities [1402,1434,1435,1436].

For example, communities in low-lying island nations are experiencing substantial amounts of plastic washing up on their shores, including swaths of MPs [1411]. See Case Study 6.1 for more information. This has negative implications for aesthetics, fisheries, and water quality—all of which are sensitive to environmental change. Concurrently, island nations are particularly vulnerable to sea-level rise impacts that result from global warming and will have to plan for managed retreat from the lands where they have lived for centuries [1412,1413,1414]. Advancing SEJ will require a reckoning by the plastics sector and by consumers of these externalities (both from climate change and plastic pollution) across the plastics life cycle.

Investigations of plastic-related pollution and social and environmental injustice are increasing, including the documentation of harmful emissions associated with plastics production and disposal. Growing legal and financial risks associated with the generation of plastic pollution may provide further impetus for change, particularly as public awareness and oppositiongrows [1398].

Section 4 of this Commission, addressing human health, underscores the need to reduce production of plastic at its source to protect human health and well-being and identifies the health consequences of plastic on the poor, minorities, the marginalized, and the people of the Global South. The fact that plastic pollution is also pervasive in high-income countries despite adequate and widely accessible waste management infrastructure underscores that the countries of the Global South cannot solely resolve the problems of plastic pollution through improved plastic waste management [540,1437]. While increasing waste management may lessen the impacts of the problem, it alone cannot solve it. Indeed, as Sections 2 and 4 emphasize, plastic is not just a waste issue but also a health issue across the plastics life cycle, which includes exposure from everyday use.

Understanding the SEJ implications of plastic-related materials and pollutants requires isolating how each stage of the plastics life cycle affects vulnerable people and groups. Here, we introduce and explore these impacts with the aim of elevating SEJ in discussions and solutions surrounding each stage of the plastics life cycle. Addressing both the urgency and complexity of these problems at all scales requires a specific set of solutions that maintains focus on the disadvantaged, marginalized, and excluded and supports the empowerment of those who suffer inequities. Specific health and environmental risks associated with each of these phases is presented in greater detail in Sections 2 (The Plastic Life Cycle and Its Hazards to Human Health) and 4 (The Impact of Plastics on Human Health).

#### SEJ issues associated with extraction of raw materials

Exploration and extraction of materials used to create plastics have disproportionately harmful effects on marginalized and vulnerable communities at scales from local to global [23,1401,1435]. These range from impacts of oil extraction on Indigenous peoples reliant on vulnerable and threatened natural resources for survival [78] to environmental and health effects of pollution from the extraction and processing of petroleum products on communities sited near chemical facilities, also known as “fenceline” communities [1430] or “sacrifice zones.” [1438,1439]

Specific activities associated with extraction include mining, fracking, and drilling for the oil, coal, and gas used to formulate essentially all (about 98%) plastics produced today [23]. For example, Section 2 details the contaminated water impacts in Nigeria in the Niger delta from oil pollution and gas flaring activities. The siting of these facilities and related exposures from activities conducted at these facilities affect many people and communities already at risk and are exacerbated in locations with absent or weak laws or enforcement of environmental or health protections. Many such locations are found in the Global South and on Indigenous lands, such as South Sudan and the Amazon basin. However, poor and vulnerable locations in the Global North are not immune, such as the infamous “Cancer Alley” in the US Gulf region, which accounts for about 25% of US petrochemical production [1402](see Box 6.2).

This harm directly affects the health of individuals and exposed groups and drives the destruction of habitats and the biodiversity needed for the survival of groups and populations. Specific drivers of these impacts include illegal or uncontrolled discharge of oil and chemicals, construction and related activities that destroy or disturb habitats and species, contamination of soil and water sources, and equipment failures and dangerous working environments for those employed in such activities. These same affected groups also commonly suffer from procedural inequities, lacking access to guidance or information while having little to no standing in decision-making; this is especially true of Indigenous groups [1402,1440].

#### SEJ issues associated with production and manufacturing

Plastics production and manufacturing are fraught with SEJ issues. The disproportionate siting of production and manufacturing facilities as well as of pipelines and compressor stations in minority and disadvantaged communities is at the literal forefront of these issues, with repercussions that cascade across human health, economics, and human rights [23,1402,1430,1439]. Poorer communities are commonly selected as sites for plastic manufacturing [1402,1437].

Environmental burdens resulting from plastic production and manufacturing are unjustly borne by marginalized and vulnerable communities worldwide across the Global North and South [1402,1435]. “Fenceline” communities and housing (in proximity or immediately adjacent to production and manufacturing plants) are often touted as providing affordable housing and the promise of local employment, while failing to acknowledge the risks associated with exposure to documented harmful emissions [9,1430]. Adverse health impacts in “fenceline” communities can compound existing inequities, including in education for individuals with few to no economic options to move or exert political influence [1439].

Chemical additives, most of them petrochemicals, used in the production and manufacture of plastics are major drivers of exposure risk [73,1431]. Nearly all plastics are derived from fossil fuels, and when plastics production facilities and fossil fuel refineries are concentrated geographically for manufacturing efficiencies, it amplifies SEJ inequities [9]. See Box 6.2 formore information.

**Box 6.2 Plastic Pollution Hotspots.**

One plastic pollution hotspot is the infamous “Cancer Alley” area along the southern Mississippi River bracketed by New Orleans and Baton Rouge, Louisiana [1436]. This largely minority black region experiences the highest cancer rates in the US [1434,1441]. In one community, resident cancer rates are overwhelmingly higher than the national average, putatively attributed to toxic emissions from a nearby synthetic neoprene factory, among other petrochemical producers [1434,1442]. These disproportionate health and exposure impacts were specifically cited in a 2022 judicial decision denying a permit for a plastic production facility in the Cancer Alley parish of St. James, Louisiana [1440].

These hotspots generate exposure to both nearby residents and workers at these facilities. As an example, in the same region, which is also repeatedly impacted by hurricanes and other disasters, the National Institute of Environmental Health Science Gulf Long-Term Follow-Up Study (GuLF Study) followed a cohort of 32,608 adults involved in oil spill response and cleanup following the 2010 *Deepwater Horizon* oil spill disaster. The study found strong associations between exposure to natural and other hazards and mental health impacts (perceived stress, distress, depression, anxiety, and post-traumatic stress disorder [PTSD]) [1443].

Concentrations, or “hotspots,” of plastics production plants and refineries are of SEJ concern worldwide [1402]. The greatest sources of plastic production, and foci of many studies, is the Global North [3,63,1434,1436]. In 2020, while Asia led the world in global plastics production at 52%, the three largest global producers were China at 32%, North America at 19%, and Europe at 15% [63]. The remainder of Asia, excluding Japan, produced 17%, the Middle East 7%, and Latin America 4%, with the Commonwealth of Independent States and Japan each at 3% of production [63].

#### SEJ issues associated with plastics use and market penetration

Plastics are inexpensive to produce and highly accessible, with broad use and market penetration, but this convenience comes at a cost that industry neither bears nor calculates. Substantial uncounted indirect costs (“negative externalities”) associated with the everyday use of plastics, particularly associated with chemical exposures from plastic, are borne by vulnerable communities and the environment [23,1431,1444].

Although plastic use is prevalent among lower-income communities, gross domestic product (GDP) is a key driver of plastics use globally, with plastic use rising as nations increase their economic capacity and attainment. For example, an individual in the US uses 255 kg of new plastic every year on average, while the average person in sub-Saharan Africa uses less than one-tenth that amount [5]. As Africa’s population and economy grow and transform over the coming century, its plastic consumption is expected to increase exponentially [14,1444]. Considering the negative health and environmental impacts associated with all stages of the plastics life cycle, the correlation of increasing GDP and plastic use warrants attention on the global stage.

Examples of uses and market penetration that increase SEJ concerns surrounding plastics and related chemical pollution include plastic water bottles, plastic packaging in general, and products and plastic packaging from fast-food restaurants and discount stores.

- Plastic water bottles provide needed drinking water while increasing the risk of MP exposure in vulnerable communities.

**Figure 6.4:**
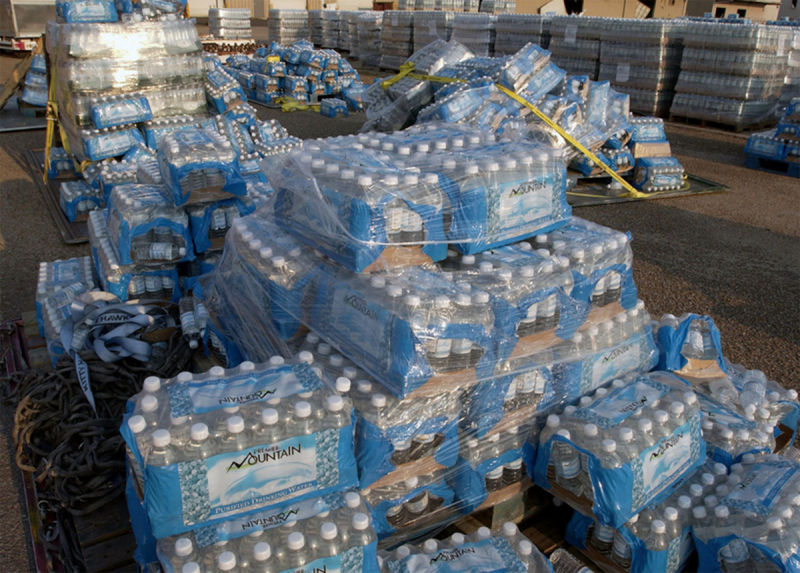
**Bottled drinking water to support Hurricane Katrina personnel in New Orleans, Louisiana**. *Credit:* MSGT Michael E. Best, USAF.

Plastic water bottles are used worldwide to provide much-needed drinking water. Globally, around two billion people lack access to safely managed drinking water at home [1445]. In some cases, local governments fail to deliver potable water to their constituents and instead rely on bottled water as the primary source of water consumption [1402]. This increases the risk of MP and plastic additive exposure in vulnerable communities because toxic additives are leachable and migrate out of products (Section 2). One study found that of 259 total bottles processed from nine different countries, 93% showed some sign of MP contamination of the water [1212]. The use of plastic-bottled water in response to natural hazards, both in the Global North and South, will likely rise given the increasing frequency and intensity of storms and associated hazards(see Figure 6.4).

- Plastic packaging contains a range of harmful chemicals that interfere with hormonal and reproductive systems in women and men.

As discussed in Section 4, use of plastics in packaging can expose users to a range of harmful chemicals, including bisphenol A (BPA), phthalates, and PFAS, all of which are used in common items such as bottles and processed food containers [1444,1446]. These chemicals can interfere with hormonal systems, damage children’s developing brains, and increase the risk of cancer [1431]. Women have an additional risk of exposure through feminine hygiene products and household items, exacerbating existing gender-related inequalities [1402]. A growing body of evidence shows that women experience reproductive disorders (including infertility and miscarriage) when frequently exposed to such chemicals [1402,1446].

- The ubiquity of fast-food restaurants and discount stores in poorer communities creates increased exposure to plastic packaging and products and associated chemicals and impacts.

Consumption of fast foods is another source of disproportionate use and exposure to harmful chemicals in vulnerable populations. Fast food served at high temperatures in plastic packaging enables harmful chemicals to migrate into the food [1431]. One study found that many popular fast foods contain an abundance of ortho-phthalates; these plasticizer chemicals are established endocrine disruptors that can increase risk of diabetes, obesity, and cardiovascular disease and also impair fertility [1447]. Individuals who frequently consume high-fat, high-salt fast-food meals are especially vulnerable to such plasticizer exposures, including lower-income minority communities living in food deserts with limited healthy food options [1447,1448].

Market penetration of plastic products is high in vulnerable and poorer communities because of business strategies and investments. For example, in the US, “dollar stores” are more likely to be found in lower-income neighborhoods, where families seek to maximize their budget and where offerings emphasize the perceived value and convenience of plastic. However, most of the merchandise is low quality and may contain toxic chemicals, such as high levels of lead and other endocrine disruptors [1402].

Despite these challenges, the widespread use and market penetration of plastics can also provide an opening for the use of consumer purchasing power to drive market innovation. By investing in reusable products and refusing single-use plastic options, consumers can collectively advocate for a more circular economy [1402]. Unfortunately, lower-income groups have less opportunity and fewer sustainable options, and even if available, they cannot always afford the better option, undermining their ability to drive market change [1402].

As societies transition toward circularity, it will be important for sustainable living practices and necessary resources to be widely accessible across economic and social conditions. Accessibility, and other SEJ parameters, need to be explicitly incorporated in solutions, as noted below.

#### SEJ issues associated with plastics waste: Leakage and management, including disposal, exports, and incineration

Waste disposal, management, and leakage and plastic waste exports have a wide range of harmful impacts, particularly on vulnerable and poorer populations. Lower-income countries, such as countries in South and Southeast Asia, bear the brunt of plastic waste mismanagement, while higher-income countries are responsible for most of the plastic production and use [23,24,407,1449].

In the Global North, plastic waste may be incinerated, recycled, sent to managed landfills, or exported to countries without adequate waste management, but in the Global South, access to organized waste management systems is indeed limited. Many cities and governments rely on incineration, or plastic is dumped into uncontrolled landfills that may leak into surrounding environments [4,9,23]. Large amounts of toxic ash by-product from incineration are another critical concern, as a large body of evidence links incineration by-products to cardiovascular diseases, cancers, stroke, and respiratory and other illnesses [9].

The piling and burning of plastic waste in dumps, as well as in towns and villages, seriously affects people living in “fenceline” communities, as particulate matter (PM), PCDDs, and PCDFs and toxic gases are released into the air as plastic burns [1438,1449,1450]. In addition, in poorer communities, plastics may be burned as fuel (heat or cooking) or as a means of disposal [1432]. The implications for human health from these practices are difficult to document, given the lack of data on exposure of the most vulnerable individuals to this range of pollutants, as described in Sections 2 and 4.

**Figure 6.5:**
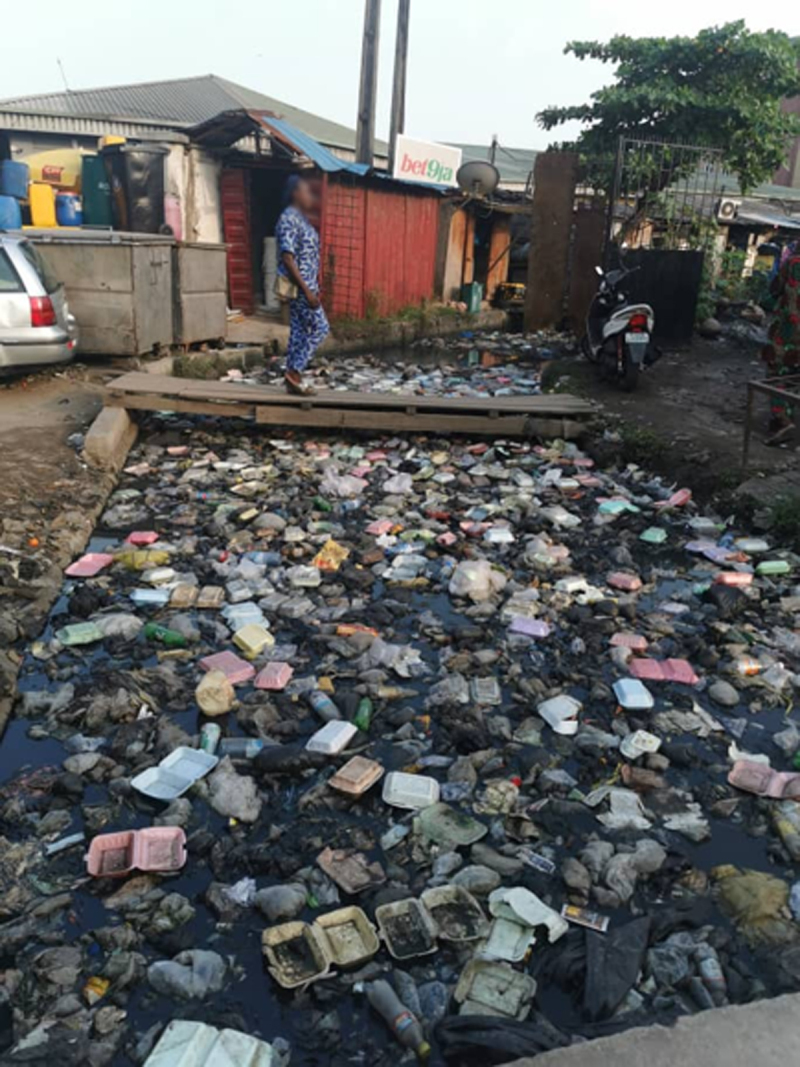
**Single use plastic waste clogging open drains in Makoko, Lagos, Nigeria**. *Credit:* Adetoun Mustapha and Korede Out.

Additional, and often unforeseen, consequences of plastic pollution on these communities can include flood risks from drainage systems clogged with solid waste that ends up in waterways, such as seen in Figure 6.5; increases in vector-borne diseases; and a reduction in tourism due to degraded environments with plastic and other wastes, which is exacerbated in poorer communities without adequate solid waste management systems [24,407,1332,1432,1451].

**Figure 6.6:**
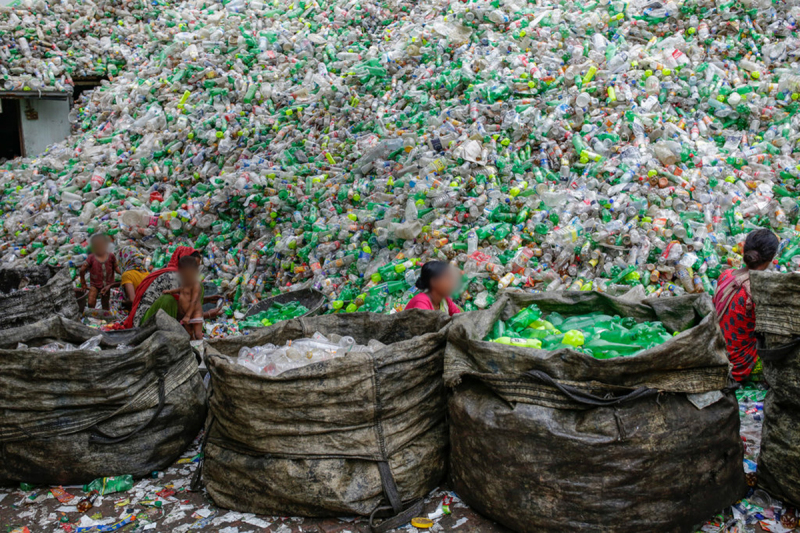
**Female workers sort out plastic bottles for recycling in a factory in Dhaka, Bangladesh**. *Credit:* Abir Abdullah/Climate Visuals Countdown.

People who work in recycling and with recycled plastic (see health risks of recycling in Sections 2 and 4) as well as in the informal waste sector, such as waste pickers, are especially vulnerable to the impacts of plastic pollution (Box 6.3), and the informal sector is growing, influenced by climate change impacts and urbanization [1452]. In developing countries, informal waste pickers perform much of the vital role of reducing the large amount of waste (especially plastic waste) in landfills and open dumpsites, where open burning often takes place [1452] (see Figure 6.6). Informal waste-picking activities also provide important social benefits, serving as opportunities for people who have few or no marketable skills and education and no alternative sources of income to survive. However, as described in Sections 2 and 4, these workers, many of whom are young children and pregnant women, are heavily exposed to harmful chemicals and contaminants through inhalation and ingestion during waste picking [373].

**Box 6.3 Gender Injustice and Impacts on Women Waste Pickers.**

Of the estimated 20 million waste pickers worldwide, the majority are women from socially and ethnically marginalized communities [1453,1454,1455]. Unjustly, women waste pickers are often “invisible” or disrespected in their societies; they work long hours in unhealthy conditions and earn lower wages compared to men [1454,1456,1457]. There is limited attention paid to the occupational health issues and social harms experienced by women waste pickers, but a few key studies from around the world report significant impacts on women as a result of consistent exposure to toxic plastic and electronic waste (e-waste), many of which contain known endocrine disruptors [1458,1459,1460,1461].

Trade in waste also creates SEJ issues. Wealthy countries that produce more waste than they can recycle at home ship much of that plastic waste to low-income and middle-income countries (LMICs) for legal or illegal disposal or putative recycling [1449]. In addition, the island nation of Indonesia and small countries like Vietnam and Malaysia that accept these materials and process them for a fee do not have systems to manage all the plastic waste they import, let alone the ability to manage the river- and ocean-borne plastic pollution that reaches their shores and coastal waters from distal and local sources [23].

Leaked plastic waste and its toxic chemical additives can be transferred through the food chain and across trophic levels [23]. Exposure to MPs via seafood is likely to be greater for populations that depend on seafood for nutrition [9,23]. Marine plastic pollution also causes negative impacts on the fisheries sector, including reduced revenues or increased costs [1462]. Vulnerable communities who rely on fisheries as a main source of income or for subsistence may be particularly affected (Case Study 6.1).

### Embedding SEJ in Solutions to the Plastics Problem

Decision makers need to prioritize and establish policies in national and international action plans to address the long-neglected and disproportionate environmental and health impacts of plastic pollution on poor, marginalized, and voiceless populations and groups [1463]. To date, the failure to do so has been due to the long latency of many pollution-related health impacts, insufficient information about pollution’s enormous economic and social costs, the vested interests of large industry, and the belief—widely held but thoroughly discredited—that pollution is an unavoidable consequence of economic development [1380].

Policies and actions to reduce plastic waste will also need to address the important social, environmental, and economic roles of high-risk groups, including workers through formal recognition of their existing roles in the waste sector and actions to reduce both worker health risks and poverty [1452]. Finally, solutions need to recognize and reduce the full cost (including “externalities” not currently considered in decision-making) associated with plastic pollution, especially to vulnerable or marginalized groups, and the environment, upon which we all ultimately depend.

Solutions will need to encompass mechanisms to resolve both distributive and procedural inequity in each stage of the plastics life cycle. This includes addressing power inequities, reducing disproportionate burdens on vulnerable and disadvantaged communities (including ensuring accessibility of those solutions), and considering the role of human agency and individuals’ choices by promoting empowerment. Principles for addressing the procedural inequities and promotion of empowerment include the following:

1. Rights to information and full disclosure of the toxic chemicals in plastic as well as the shedding potential of MPs
2. Appropriate training for workers
3. Public participation in decision-making
4. Opportunities for input and partnership for all
5. Ensuring involvement and guidance for stakeholders and vulnerable communities in environmental decision-making processes [1402]

In alignment with the identified SEJ principles and numerous statements from civil society and worker groups, the Intergovernmental Negotiating Committee (INC) urged delegates to address SEJ in the Global Plastics Treaty at the first meeting (INC-1) [1464]. Additionally, the Office of the High Commissioner on Human Rights’ statement to the INC-1 about key human rights considerations for the Global Plastics Treaty informs some of the critical tasks for embedding SEJ in solutions. Recommendations included promoting the human right to a clean, healthy, and sustainable environment; safeguarding the rights of those who suffer the most from plastic production and pollution; holding businesses accountable to remediating or protecting against human rights harms; and transitioning toward a chemically safe circular economy that address all stages of the plastics life cycle [1465].

The emerging concept of ocean equity similarly asserts the need for attention to many dimensions of equity from the beginning to the end of any decision-making process, as in Figure 6.7 from Bennett (2022): (1) recognitional equity, (2) procedural equity, (3) management equity, (4) distributional equity, (5) environmental equity, and (6) contextual equity. This framework would also be particularly applicable to the issues associated with plastic pollution even beyond coastal and ocean-dependent communities.

**Figure 6.7:**
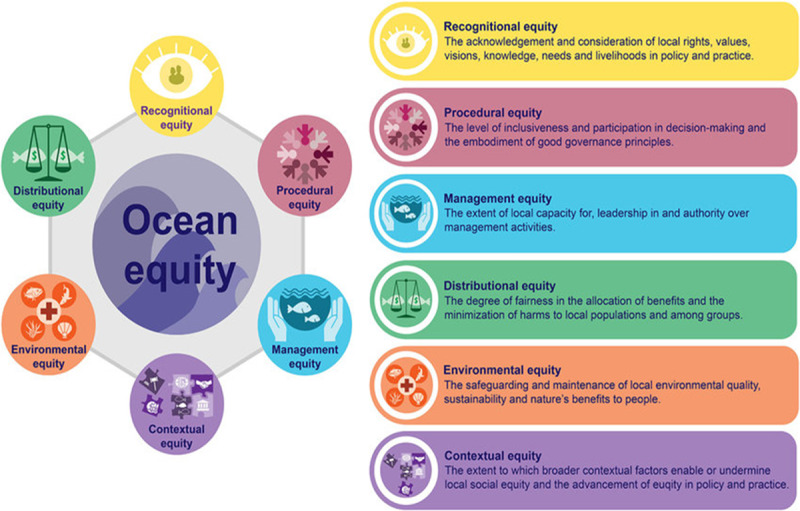
**Ocean equity is comprised of several distinct dimensions.** Bennett (2022) describes, and depicts in this figure, a range of equity considerations (which include many aspects of procedural and distributional equity) relevant to those working in coastal and marine conservation. These equity dimensions may also provide a useful framework for embedding social and environmental justice (SEJ) in decisions and processes relevant to plastic production and pollution at the local to global scale – even beyond coastal and marine areas. *Original source:* (Bennett, 2022).

#### A key inequity: Lack of funding for SEJ research

A lack of long-term and sustained funding hampers our understanding and limits development of solutions to plastics-associated SEJ problems. Proportionally less funding for marine conservation, including marine pollution and marine science, goes to developing countries, particularly Africa [1466], which may contribute to limited knowledge regarding social justice–related plastic issues in areas of the Global South. Little research has been conducted on plastic pollution and impacts on human health, ecosystems, economies, and SEJ in LMICs compared to high-income countries. For example, with about 2.5 Mt of plastic waste annually, Nigeria ranks ninth globally among countries with the highest contributions to plastic pollution [24], and more than 88% of the plastic waste generated in Nigeria is not recycled but flows to lagoons and the ocean [1467,1468,1469]. A systematic review of academic studies on plastic pollution in the environment in Nigeria conducted by Yalwaji et al. (2022) [1469] shows that as of May 30, 2021, there were only 26 such studies in Nigeria, compared to 62 peer-reviewed studies on the Arctic Ocean [1470]. Between 1987 and September 2020, there were only 59 studies on plastic pollution in the African aquatic environment [1471].

Due to resource constraints and the lack of research prioritization in LMICs, most LMICs’ governments have failed to allocate meaningful resources for research. Lack of research funding in LMICs has several implications for generating high-quality evidence to inform policy and practice [1472] and impedes achievement of the UN Sustainable Development Goals. Regarding marine-focused funding for conservation priorities such as pollution, grant making has historically allocated a sizable proportion of funding to global initiatives (40%) and work focused on North America (32%). Over the past decade, an increased proportion of marine funding (15%) was allocated to Asia. Funding to Africa remains limited at less than 3% of philanthropic funding [1466].

Currently, much of the literature on environmental justice issues related to plastics focuses only on local to national scales and is US-centric [1402]. Future studies should enhance focus on international case studies to better comprehend the range of environmental justice implications from global dependence on plastics [1402]. A much wider evidence base is required regarding environmental justice and equity. Transdisciplinary research that demonstrates the multifaceted interplay among the plastics life cycle, environment, and society and how the nexus of these elements produces inequities within and between countries is necessary to drive global strategies and policy interventions. Implementation research that demonstrates just and equitable outcomes with the potential to reduce burdens experienced by communities most impacted by marine plastic pollution are also necessary. Research to better understand and quantify the potential health impacts of plastic pollution to humans (see Section 5, Economic Impacts) and aquatic and terrestrial ecosystems is key for science and data-driven solutions.

#### SEJ solutions

##### Primary SEJ solution: Reduce plastic pollution and health risks at the source and hold producers accountable

While it is important to act at all stages of the plastic life cycle and at all scales, from local to global, the economic and health costs of plastic pollution on vulnerable populations require that reducing the sources of plastic pollution and health impacts (including reducing production and addressing the health risks of additives and MPs) be preeminent in the hierarchy of SEJ solutions. This includes ensuring that burdens are removed from those least responsible and ensuring that responsibility lies with those who create and profit from plastic pollution. Addressing pollution and other problems at their source will be not only efficient but also effective, and it will ensure responsibility is borne by producers, or “perpetrators.” [1406,1473] Expert reports have shown that “reducing plastics production, consumption and improving waste management to turn off the tap of plastics pollution is easier than attempting to clean up ocean plastics,” as well as being more cost-effective [1402,1407,1437,1474]. Increasingly, courts are also recognizing that responsibility lies with plastic producers, manufacturers, and transporters under both statutory and common law, creating new impetus for “polluter pays” solutions [1398,1475].

Moreover, an approach that assigns much greater legal and financial responsibility to the plastic industry for previously externalized costs aligns well with the 2021 recommendation of the UN Special Rapporteur on Toxics, in UN General Assembly Report A/76/207, that plastics be addressed using a “human rights–based approach,” citing the specific impacts of plastic on the following vulnerable populations [1476]:

- Workers in the petrochemical and plastics manufacturing industries and waste pickers;
- Children, who when exposed to hazardous substances in the plastics cycle suffer a violation of their rights to life, health, physical integrity, and a toxic-free environment;
- Women, who are politically underrepresented in decision-making processes;
- People of African descent, who endure proximity to a higher concentration of hazardous waste facilities, contaminated sites, and dumping grounds;
- Indigenous peoples, whose lands are contaminated through exploitation of fossil fuels, which comprise the bulk of plastic feedstock;
- Coastal communities inundated with marine plastic litter;
- People living in poverty, who often reside close to chemical industries and are on the receiving end of the global plastic waste flow; and
- Future generations, whose ability to enjoy their human rights and a healthy environment is being threatened.

The UN General Assembly’s declaration in 2022 that a “clean, healthy and sustainable environment” is a human right confirms obligations to address plastic pollution where it begins, with production [1477].

##### Key SEJ solutions: Procedural equity

In addition to addressing distributive inequity, which is the disproportionate impact of harm to specific peoples and communities, there is a need to institute procedural equity in government and nongovernment sector decision-making across the plastics life cycle. Action in five key areas will be needed to advance procedural solutions to SEJ plastics pollution issues: (1) equitable and inclusive participation, (2) full economic cost estimation, (3) transparency and access to information, (4) acknowledge and address societal roles, and (5) fill data and funding gaps.

###### Equitable and inclusive participation

Governments and private sector actors should adopt the participatory approach to ensure transparent processes and meaningful participation of affected communities in decision-making [200]. Such an approach would include the following elements:

- Identify the objective of the intervention and the socioeconomic and environmental context of implementation; this information can inform the effectiveness of interventions, the timing for such action, and relevant stakeholders;
- Identify and engage key stakeholders in a participatory process to ensure a complete assessment of costs and benefits relevant to all stakeholders and a more equitable sharing of costs; this includes environmental, social, and economic costs (as well as benefits) of “conservation solutions,” e.g., waste-to-energy facilities;
- Include environmental justice principles, such as prior informed consent and rights to information, into processes and legislation at all levels;
- Monitor and report progress toward implementation of such policies or directives to ensure transparency and accountability; and
- Establish mechanisms for affected communities and individuals to seek legal redress and accountability, e.g., via legislation or common law.

###### Full economic cost estimation

To fully inform decision-making with SEJ considerations, economic quantification methods must capture the full economic cost of an action over time and across groups (see, e.g., [200] and Section 5, Economic Impacts). A broader public understanding of the full costs associated with plastics is essential to engender actions needed to remedy social and environmental injustice associated with plastics production, use, and disposal.

Many costs and benefits can be difficult to quantify—particularly indirect costs, nonmonetary costs, monetary benefits, and nonmonetary benefits—but other methods exist and should be employed for quantifying costs and benefits, such as the following:

- Cost-effectiveness analyses—first used in public health [1478];
- Ecosystem services valuation to estimate the value of nonmonetary costs and benefits of plastic pollution interventions [1433]; and
- Recognition that a lack of standardization across approaches may create challenges for comparing values across studies and contexts [1479].

###### Transparency and access to information

Lack of equal access to information and knowledge places workers and underserved groups at a significant disadvantage in decision-making on plastic pollution. Of the utmost importance is advancing the access of such individuals and groups to a range of relevant, evidence-based information, including the state-of-the-science and procedural requirements and legal rights. Some examples of actions that would address transparency and access to information follow:

- Create a science body to inform and advise negotiations on the Global Plastics Treaty (currently proposed);
- Provide information and education to raise awareness of the sources and impacts of plastics, including human and environmental health impacts of chemical and plastic pollution;
- Provide advice and education on alternatives to plastic, including benefits and disadvantages, and how to obtain such alternatives;
- Ensure facility workers and affected communities understand and receive their legal and other rights with respect to hazard exposure and other plastics-mediated impacts; and
- Invest in local education on plastic pollution cleanup campaigns to empower impacted communities to be part of decision-making processes.

###### Acknowledge and address societal roles

Any process should recognize and accommodate economic and societal roles in legacy to novel systems to reduce plastic pollution. Examples of such recognition and accommodation might include the following:

- Integrate the informal waste and recovery sector into formal waste management channels to provide better working conditions and jobs and reduce exposure to toxic conditions.
- Address human rights and quality of life issues in affected communities, including providing reparations where appropriate. For example, California legislation enacted in 2022 will require plastic producers to pay US$500 million a year for 10 years starting in 2027 for environmental mitigation and to address harms to disadvantaged, low-income, and rural communities [45].
- Address the livelihoods of those relying on marine resources where plastic pollution has negatively impacted trade of toxic MNP-contaminated foods, including seafood.

###### Fill data and funding gaps

It is imperative to invest resources to identify and fill major gaps in funding and to generate knowledge in key areas critical to equitable decision-making. This may include the following:

- Given extant literature on SEJ and plastics focuses on local to national scales and is US-centric, focus future research on international case studies and scales to enhance our understanding of the range of environmental justice implications due to global dependence on plastics [1402];
- Facilitate and pursue epidemiological studies and air-quality studies focused on waste pickers and those who burn plastics in their business or home settings;
- Obtain more information and longitudinal studies about “fenceline” communities through natural and social science investigations, ideally using a transdisciplinary focus;
- Gain a broader understanding and definition of economic and social costs of pollution-related disease to overcome assumptions that pollution is an “unavoidable consequence of economic development” [1463]; and
- Determine the efficacy of various proposed solutions in different geographies and socioeconomic conditions and establish the trade-offs among solutions and the potential for unintended consequences.

##### Key SEJ solutions: Stage-specific and distributional equity

In addition to the foci previously discussed, stage-specific actions will also be necessary to mitigate the specific SEJ issues associated with the plastics life cycle. Examples of such actions are outlined below.

###### Extraction, production, and manufacturing stages

- Reduce plastic production and implement health protective standards for plastic, especially for MPs and chemical additives, to protect groups vulnerable to plastic impacts, such as those identified by the UN High Commissioner on Human Rights.
- Require equitable siting of extraction and processing operations through meaningful participation in decision-making and community and other stakeholder access to scientific and other relevant information.
- Create safety zones in vulnerable communities, establishing a minimum distance, or setback, for extraction sites located near people and communities. For example, the state of California recently passed SB 1137, a bill to protect the public health of California’s “fenceline” communities by creating a minimum health and safety distance of 3,200 feet between sensitive receptors (such as a residences, schools, childcare facilities, playgrounds, hospitals, and nursing homes) and oil and gas production wells [1480].

###### Use and market penetration stage

- Address price differentials for plastics and alternative products and ensure substitutes are accessible, recognizing current plastics’ pricing fails to reconcile externalities;
- Advance, improve, and regulate plastics products’ labeling regarding recycling and other disposal routes that more accurately reflects end-of-life options, or lack thereof, of products, e.g., the (non)availability of recycling of certain polymers and the availability of composting facilities in communities, states, and nations;
- Ensure accessibility of more benign substitutes, e.g., multiuse, refillable containers and infrastructure that obviates use of avoidable and problematic plastic sachet-type products; and
- Establish fuel alternatives for those who burn plastic in their homes (e.g., the UN Clean Cookstoves initiative).

###### Waste stage (including leakage and management)

- Establish and enforce producer responsibility for the full life cycle costs and impacts of plastics products and packaging, with an emphasis on responsibility for costs and impacts affecting underserved communities and regions.
- Involve the informal waste sector, a critical stakeholder group exemplified by waste pickers, in the design and development of activities and strategies to address plastic pollution. For example, the recently launched Just Transition Initiative coordinated with the International Alliance of Waste Pickers and other stakeholders will ensure that the concerns of waste pickers are amplified and addressed in the proposed Global Plastics Treaty [1481].
- Empower the informal plastics waste sector by connecting waste pickers with potential buyers to create transparency in the value chain that facilitates pickers earning fairer wages and better working conditions [42].
- As recommended by the United Nations Environment Programme (UNEP) and Azul (2021) [1402], countries should restrict importation of toxic plastics in cooperation with the Basel Convention and through the passage of more restrictive legislation on substances of concern [1402]. National authorities and stakeholders should collaborate on international policies, agreements, and initiatives related to waste export and management [1402].
- Support and employ the informal local waste sector (e.g., waste pickers) with fair wages, improved economic status, and worker protections in a transition to safer recycling and management systems for plastic waste [42].
- Explore international policies prohibiting open burning of various types of waste to reduce toxic exposures and emissions.

### Conclusion

Adequately addressing SEJ with respect to plastics pollution will require honest consideration of the following:

- The reasons we produce plastic;
- The decision makers and those absent or excluded from decision-making;
- The resources needed to obtain plastic products;
- The market forces and drivers that lead to the production and consumption of plastic (e.g., the use of bottled water);
- Who influences and controls the above dynamics; and
- Who and what suffers where plastic products and their derivatives accumulate and negatively affect all forms of life and ecosystems.

However, such change will invoke predictable resistance by those benefiting from the status quo, including powerful individuals, governments, and corporations, especially the integrated multinational fossil carbon corporations that both produce coal, oil, and gas and manufacture plastics and plastic additives. Groups with acquired privileges and power—whether individuals, organizations, or multinational corporations—will resist critical examination of current plastics production and use [46,1482]. Thus, governments and society will need to give extra attention to SEJ in the designing of durable and equitable strategies. Critically, action is necessary at all levels. A case study from Indonesia provides examples of how government, business, and other actors can all participate, and it provides some hope for future action—once SEJ inequities are addressed (Case Study 6.2).

**Case Study 6.2 Indonesia’s Response to Plastic Waste.**

**Figure 6.8:**
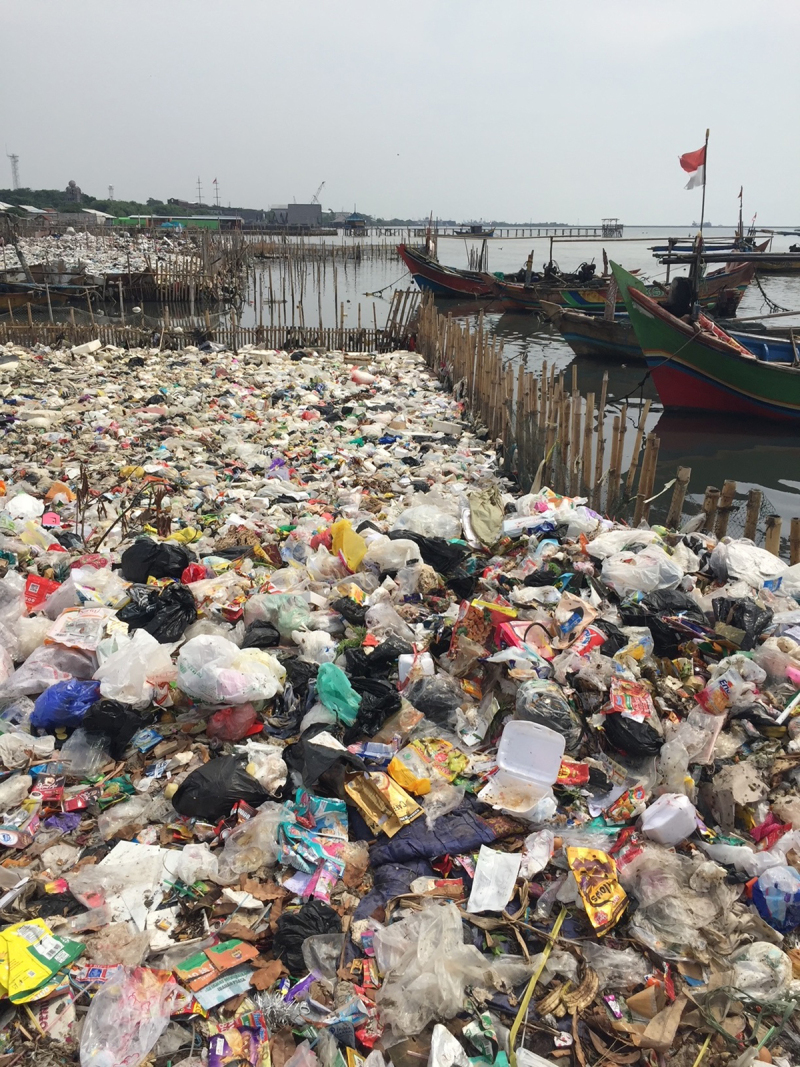
**Trash next to a waterway in Indonesia**. *Credit:* Credit to Richard C. Thompson, University of Plymouth.

Both on land and in the ocean that encircles its islands, Indonesia is currently experiencing a trash catastrophe (Figure 6.8). The second-largest source of the vast amount of oceanic plastic garbage is Indonesia. The nation and its citizens suffer negative economic effects because of this waste. Currently, barely 10% of the 6.8 Mt of plastic trash generated in Indonesia (including imported plastic trash) each year makes it to recycling facilities. The ocean receives about 625,000 tons of plastic waste each year. Waste management is lacking or inadequate across large areas of Indonesia, resulting in informal dumping and burning of litter on a substantial scale. Where management exists, landfills are frequently located relatively close to residential areas, and contaminated effluent can leak into those areas and impede the growth of surrounding crops.

Contaminated effluent as well as large quantities of solid plastic waste enter rivers and impact the people whose livelihoods depend on them. Due to the harm caused to marine life by plastic pollution in the waters, the fishing sector also suffers [22,1483]. Videos showing trash-filled beaches in popular tourist locations like Bali have gone viral, alarming the tourism sector and potentially damaging Indonesia’s economy. The possible effects of this high pollution on tourism are a source of concern. Fortunately, the problem has been acknowledged, and measures exist to deal with Indonesia’s plastictrash issue.

*Actions by individual and community*

People, organizations, and the government are mobilizing to solve and lessen Indonesia’s plastic trash crisis. The first step is to become aware of the issue. Local Indonesians have made a big contribution to organizing campaigns and raising public awareness.

For example, Melati and Isabel Wijsen established the environmental nonprofit Bye Bye Plastic Bags when they were just 12 and 10 years old, respectively [1484]. Bye Bye Plastic Bags has become one of the largest environmental nonprofits in Bali and is helping to educate children on the environmental harm of plastics. Another individual, Mohamad Bijaksana Junerosano, founded the social enterprise Waste4Change [1485]. It educates the populace on sorting and sustainably managing waste.

Initiatives to clean up the community have also gained popularity recently. Simple and efficient ways to engage people are beach cleanups. For a one-day beach cleaning in August 2018, that also brought attention to the garbage situation, more than 20,000 individuals organized in 76 places around Indonesia [1486].

*Action by the government*

Both local and national levels of government have taken the most important steps to end the crisis of plastic waste in Indonesia. The island of Bali banned all single-use plastics at the end of 2018 [1487]. The capital of Jakarta also banned single-use plastic bags in its shopping centers and street markets in 2020 pursuant to Governor’s Regulation No. 142/2019 [1488]. Internationally, the UK has invested over US$5 million in research to help identify solutions, including in Indonesia via the Pisces Partnership [1489].

Indonesia’s national government has rolled out a very ambitious plan to end the plastic waste problem. It aims to minimize marine plastic waste by 70% by 2025 and be entirely rid of plastic pollution by 2040. Indonesia created five action points to make it easier to meet these overall goals [1490,1491]:

1. Reduce or replace plastic use by avoiding single-use plastic packaging;
2. Rethink the designs of plastic products and packaging to allow for multiple use and recycling;
3. Double the current plastic waste collection of 39% to 80% by 2025;
4. Double current recycling capacity by investing in infrastructure capable of processing an additional 975,000 tons of plastic annually; and
5. Develop or expand on proper waste disposal infrastructure that can process an additional 3.3 Mt of plastic waste annually.

Though reducing plastic waste in Indonesia and its oceans is a challenge, ordinary people and the government of Indonesia are taking proactive steps. Hopefully, these efforts will have a positive impact on livelihoods, the economy, and the health of people. The future looks brighter for a cleaner Indonesia.

## Section 7—Findings and Recommendations

### Introduction

Plastics are ubiquitous in modern societies. They have supported breakthroughs in fields as diverse as medicine, electronics, aerospace, construction, food packaging, and sports.

It is now clear, however, that current patterns of plastic production, use, and disposal are not sustainable and are responsible for significant harms to human health, the environment, and the economy as well as for deep societal injustices.

While there remain gaps in knowledge about plastics’ harms and uncertainties about their full magnitude, the evidence available today demonstrates unequivocally that these harms are already great and that they will increase in magnitude and severity in the absence of urgent and effective intervention [34]. Manufacture and use of essential plastics may continue. But reckless increases in plastic production, especially increases in the manufacture of an ever-increasing array of unnecessary single-use plastic products, that take no heed of health or environmental consequences must be curbed. Global intervention against the plastic crisis is needed now, because the costs of failure to act will be immense.

The good news is that many of plastics’ harms can be avoided via better practices of production, design of alternative, less toxic materials, and decreased consumption. Plastics’ harms to human health, the environment, and the global economy can be mitigated by building on the same cost-effective strategies that international bodies and governments at every level have used for 50 years to prevent and control air, water, soil, and ocean pollution [21,1380].

Contrary to the oft-heard tropes that pollution is the unavoidable price of progress and that pollution control destroys economies, a review by the *Lancet* Commission on Pollution and Health clearly demonstrates that actions taken by governments to prevent and control pollution have, in fact, yielded large positive returns on investment [9]. Thus, every dollar invested in air pollution control in the USA since passage of the Clean Air Act in 1970 has yielded a return of $30 (USD) (range, $4–88) [1492]. These gains resulted from the substantially increased economic productivity of a healthier, longer-lived population and from reductions in the costs of health care associated with pollution-related disease. Likewise, the removal of lead from gasoline in the USA reduced children’s blood lead levels by 95% and has generated an estimated economic benefit of $200 billion (USD) in each year’s annual birth cohort since 1980—an aggregate benefit in the past 40 years of over $8 trillion USD [1493]. This large economic gain resulted from the population-wide increase in children’s cognitive function (IQ scores), creativity, and productivity that followed widescale reduction in lead exposure.

### Main Findings

This Commission has four major findings:

#### Main Finding #1

Current practices for the production, use, and disposal of plastics cause great harms to human health and the global environment, and they are not sustainable. These harms arise at every stage across the plastic life cycle and are described in Sections 2 and 4 of this Commission. They include human health impacts such as developmental neurotoxicity, endocrine disruption, and carcinogenesis. In the ocean (Section 3), plastics’ harms extend far beyond the visible and well-recognized damages of beach litter, contaminated mid-ocean gyres, and physical injury to marine species and include extensive injury to marine ecosystems. Plastic production results in GHG emissions equivalent to nearly 1.96 Gt of CO_2_e annually that contribute to climate change.

The main driver of plastics’ worsening harms is an almost exponential and still accelerating increase in global plastic production. More than half of all plastics ever produced have been manufactured since 2002. Plastics’ harms are further magnified by low rates of recovery and recycling—less than 10% globally—and by the long persistence of plastic waste in the environment. The result has been the accumulation since 1950 of nearly 6 Gt of plastic waste that now pollutes every corner of the planet [3].

#### Main Finding #2

The thousands of chemicals in plastics—monomers, additives, processing agents, and NIAS—are responsible for many of plastics’ known harms to human and planetary health (Section 2). These chemicals leach out of plastics, enter the environment, cause pollution, and result in human exposure (Section 4). In the environment and in the bodies of living organisms, many plastic-associated chemicals can undergo chemical transformation to form breakdown products and metabolites, some of which are highly toxic and contribute further to plastics’ harms.

Plastic manufacturers disclose little information on the identity, chemical composition, or potential toxicity of plastic chemicals at the time of entry to market and in most countries are under no legal obligation to do so. Both the complexity and the lack of transparency regarding the chemical composition of plastics has led to the current situation in which publicly funded epidemiologic research must attempt to discover possible health impacts of plastic-associated chemicals, but only after these chemicals have been released to market and resulted in potentially widespread human exposure.

#### Main Finding #3

The economic costs of plastics’ harms to human health and the global environment are very high (Section 5). We estimate that in 2015 the health-related costs of plastic production exceeded $250 billion (2015 Int$) globally, and that in the US alone the health costs of disease, disability, and premature death caused by just three plastic-associated chemicals (PBDE, BPA, and DEHP) exceeded $920 billion (2015 Int$). The cost of GHG emissions from plastic cause economic harms that we value at $341 billion (2015 Int$) annually.

These costs, large as they are, underestimate the full costs of plastics’ impacts on human health and the environment. All of these costs are externalized by the petrochemical and plastic manufacturing industries, and they are borne by individual citizens, taxpayers, and their governments without compensation.

#### Main Finding #4

The health, environmental, and economic harms caused by plastics disproportionately affect vulnerable and at-risk populations—the poor, people of color, and Indigenous populations as well as fossil fuel extraction workers; plastic production workers; informal waste and recovery workers; persons living in communities adjacent to fossil fuel extraction, plastic production, and plastic waste facilities; and children [1494,1495,1496,1497,1498,1499,1500] (Sections 4 and 6). These disparate harms are seen in countries at every level of income, including high-income countries [1494,1497,1499]. They are seen globally in the export of vast quantities of plastic waste, including plastic-laden e-waste from high-income to low-income countries, where this waste accumulates in open tips and landfills, pollutes air and water, degrades vital ecosystems, befouls beaches and estuaries, damages fisheries, and harms human health, especially children’s health [1495,1498]. SEJ principles require reversal of these inequitable burdens to ensure that no group bears a disproportionate share of plastics’ harms and that those who benefit economically from plastics bear their fair share of its currently externalized costs.

### Recommendations for Policy Makers

This Commission’s strongest recommendation is that the Intergovernmental Negotiating Committee (INC) for the Global Plastics Treaty develop and implement a strong and comprehensive, legally binding Treaty that ensures urgent action and effective interventions at an international scale across the entire life cycle of plastics to end plastic pollution, pursuant to the mandate set forth in the March 2022 resolution of the UNEA [37]. Progress toward development of this Treaty is already underway, and the first meeting of the INC took place in Punta del Este, Uruguay, in late 2022 [1501,1502].

International measures to curb plastic production and pollution are critical because the harms to human health and the environment caused by plastics, plastic-associated chemicals, and plastic waste transcend national boundaries, are planetary in their scale, and have disproportionate impacts on the health and well-being of people in some of the world’s poorest nations. A powerful and effective Treaty, consistent with fundamental principles of precaution, would build on models already elaborated in existing multilateral environmental agreements [1503,1504].

Experience with accelerated timelines for such other agreements as the Ottawa Land Mine Convention [1505,1506] and the Nuclear Weapons Prohibition Treaty [1507] suggest that the timeline proposed in the UNEA resolution, for completion of Treaty development by the end of 2024, is realistic.

The Commission notes that effective implementation of the Global Plastics Treaty will require coordinated action at the global, national, regional, and local levels. This Commission encourages national, regional, state and local policymakers to be involved in the negotiations on the Treaty, including to support evaluation of the efficacy and feasibility of measures proposed for inclusion in the Treaty as negotiations proceed. National and local policymakers are uniquely well-positioned to pilot test and assess the efficacy of harm reduction strategies, and their experience can provide valuable guidance and real-world grounding to the treaty deliberations.

#### 1. Addressing Unsustainable Production

##### 1.1 Global Cap on Plastic Production

**This Commission recommends that a global cap on plastic production be a central provision of the Global Plastics Treaty**.

Given the great and growing magnitude of the harms caused by plastics to human and planetary health [1508,1509] (Sections 2, 3, 4, and 6), the substantial and still undercounted economic costs resulting from those harms (Section 5), and the enormous increases in plastic production projected for coming decades (Section 2), this Commission is of the considered opinion that a cap on plastic production is well-justified, much needed, timely—and importantly—the most effective harm-reduction strategy.

The great power of a global cap on plastic production is that it will reduce the volume of plastics and plastic waste at its root source. It will slow the current massive global buildout of plastic production infrastructure. It will help put the world on track to end plastic pollution by 2040, a target put forth by the High Ambition Coalition to End Plastic Pollution [1510].

Like the phase-out of ozone-depleting chlorofluorocarbons under the Montreal Protocol [1511] and the removal of lead from gasoline [1512], a global cap on plastic production will have far-reaching benefits for planetary and human health. As was the case with both of those interventions, a cap on plastic production can be expected to have salutary public policy impacts by encouraging industry to develop new technologies and substitutes for existing uses.

Complementary, “downstream” control strategies such as enhanced plastic recovery, recycling, and reuse need also to be encouraged. Experience indicates, however, that these approaches are inherently less effective than “upstream” prevention and that without curbing continuing unsustainable production of plastics, they may be expected to continue to lag behind for the foreseeable future (Section 2) [57,577].

This Commission recognizes that for a global cap on plastic production to be effective, the Treaty will need to include a roadmap that stipulates targets and timetables, and the Treaty includes national contributions that are binding. Targets and timetables for LMICs will likely need to be less stringent than those for high-income countries, to facilitate a just transition.

##### 1.2 Bans on Unnecessary, Avoidable and Problematic Plastic Items

**This Commission recommends that a provision banning or severely restricting manufacture and use of unnecessary, avoidable, and problematic plastic items, especially single-use items, be included in the Global Plastic Treaty**.

As is described in Section 2, manufacture of single-use plastic products accounts for 35–40% of current plastic production, and this fraction is growing rapidly. Inclusion in the Global Plastic Treaty of a provision restricting manufacture and use of unnecessary single-use plastics will help curb current unsustainable increases in plastic production and slow the accumulation of plastic waste. The Montreal Protocol and the Stockholm Convention both provide precedents on how such a ban or restriction could be structured under the Global Plastic Treaty.

Many countries, states, and cities have already successfully imposed bans on some single-use plastic items [1513,1514,1515,1516]. Additional strategies for limiting use of single-use plastics at the national and local levels are suggested in the UNEP report, *Single-Use Plastics: A Roadmap for Sustainability* [1432].

This Commission suggests that manufactured MPs such as microbeads in cosmetics (see Box 7.1) be considered as a target for banning under the Global Plastics Treaty.

**Box 7.1 Manufactured microplastic particles—“microbeads”.**

**Manufactured microplastic (MP) particles.** Manufactured MP particles, often called “microbeads” are now intentionally added to many personal care products and cosmetics, including sunscreen, shampoo, makeup, and deodorants as well as in other commercial and consumer products [1233].

These products contribute directly to environmental contamination and human exposure yet their perceived benefit to society is trivial [1517]. To counter these materials’ potential hazards to human health and the environment, several countries including New Zealand, Canada, UK, and the Republic of Korea and have banned plastic microbeads in cosmetics and personal care products [1518]. In the US, the Microbead-Free Waters Act of 2015 prohibits the manufacture, packaging, and distribution of rinse-off cosmetics containing plastic microbeads [1519]. In 2019, the European Chemicals Agency proposed a sweeping restriction on the use of MPs in all types of EU market products. A year later, the agency’s Committee for Risk Assessment recommended an additional ban on all MPs utilized in infill for artificial turf fields [1520]. The final EU rule on intentionally added MPs in products is scheduled to be released soon [1518].

The Nordic Council of Ministers recommend minimizing MP releases at every stage of the plastic life cycle [1521].

#### 2. Addressing Harm Caused by Chemicals in Plastics

##### 2.1 Inclusion of Chemicals in Scope

**This Commission strongly recommends that the scope of the Global Plastics Treaty extend beyond MPs and marine litter to include all of the many thousands of chemicals incorporated into plastics** [8,43,1522] **(Section 2)**.

Thousands of chemicals, including monomers, additives, and NIAS, are incorporated into plastics during manufacture and are integral components of plastic products, macroplastic waste, and MP particles. Many of these chemicals are responsible for a very great part of the harms to human health and the environment caused by plastic. Over 2,400 plastics chemicals have hazard ratings that are of high concern. Most of the rest have never been assessed for their potential impacts on human and ecosystem health. Plastics chemicals include neurotoxicants, human carcinogens and endocrine disruptors. As is documented in Section 4, plastics chemicals are especially dangerous for infants in the womb, young children, and pregnant women.

##### 2.2 Establishment of Health Protective Standards for plastic-associated chemicals

**This Commission recommends the establishment of health-protective standards for plastic-associated chemicals under the Global Plastics Treaty, including global rules to be implemented at national level, requiring testing of all polymers and plastics chemicals for toxicity before they enter markets**.

The incorporation of hazardous chemicals into plastic production is part of the larger problem of inadequate regulation of chemicals in consumer goods. Health-protective standards for plastic-associated chemicals and their associated reporting obligations should address five major areas: sustainable design; mutual acceptance of data; transparency on the chemical composition and toxicity of plastic-associated chemicals; and systems for human biomonitoring and post-market surveillance [1523].

- **Sustainable Design**: If plastics are to continue to provide benefit to humanity while not harming human and planetary health, they need to be fundamentally redesigned to use sustainable, non-toxic, and more circular materials. Mandatory health-protective standards applied to all ingredients in plastic—both polymers and other plastics—are a mechanism for achieving this goal.
  These standards need to prohibit the incorporation into plastics of toxic chemicals and chemicals of known concern for either humans or the environment. The current practice of allowing new plastic-associated chemicals to enter markets without full disclosure and comprehensive toxicity testing is dangerous and unsustainable. It has time and again resulted in widespread disease and premature death [1524]. The INC’s remit in the UNEA Resolution specifically calls for it to promote sustainable design. International regulatory cooperation for chemicals is essential to harmonize chemical assessments with the aim of minimizing risks [1525].
- **Mutual Acceptance of Data**: The Mutual Acceptance of Data system is a framework whereby OECD chemical safety guidelines developed in one adhering country must be accepted in all adhering countries and is helping to minimize regulatory divergencies, and the framework facilitates work-sharing with a recent case-study indicating significant cost-benefit estimated at ~3 million Euros [1525]. In addition to OECD member countries, seven non-OECD member countries (Argentina, Brazil, India, Malaysia, Singapore, South Africa, and Thailand) are participating [1525].
  To avoid conflict of interest, it is important that all toxicity testing of plastic-associated chemicals be performed by independent laboratories and not by the chemical manufacturing industry or by laboratories financed by the industry. Such an independent testing program could be supported by user fees paid to national governments by chemical and plastic manufacturers, as is done today in registration of pharmaceutical and food chemicals [1526].
- **Transparency**: Mandatory disclosure regimes are needed to require labeling of the full chemical composition of all plastics products, including resins, polymers, additives, monomers, and adjuvant chemicals as well as information on transformation products of these materials. The UNEA mandate addresses this question, calling upon the INC to consider the provision of policy-relevant scientific information and assessment related to plastic pollution in its drafting of the Global Plastics Treaty.
- **Traceability**: Mandatory disclosure on composition should be complemented by the systematic collection of data on the specific chemical signatures of specific plastic products, which can be made publicly available so that waste can be tracked, traced, and effectively managed.
- **Human Biomonitoring**: Even with adequate premarket toxicity testing, it is never possible to fully know all of the short- and long-term health risks that may be associated with plastic-associated chemicals until wide-scale human exposure has occurred. Health protective standards for plastics chemicals should therefore be complemented by mandatory systematic biomonitoring and post-market surveillance of plastic chemical exposures in human populations, as is already routinely done in the pharmaceutical and food industries [1526]. These biomonitoring programs should cover LMIC and vulnerable populations as well as populations in high-income countries. Effective biomonitoring will require surveillance for plastic-associated chemicals as well as for their degradation and biotransformation products. It will also require a sustainable funding mechanism, which could be funded by industry through chemical registration fees, as is routine practice in the pharmaceutical and food chemical industries.

##### 2.3 Reducing the Complexity of Plastic Products

**This Commission recommends inclusion in the Global Plastics Treaty of mechanisms for reducing the complexity of plastic products**.

Currently, many different chemicals are used to perform similar functions in plastic production. This, together with a lack of coordination among manufacturers, has resulted in an enormous proliferation of different types of plastic. This complexity poses great challenges to downstream control efforts, including recovery and recycling. These problems are compounded by the lack of any system to trace the identities and levels of the chemicals present in specificplastic products.

To address this issue, the UNEA resolution specifically identifies the need to address the design of products and materials

…so that they can be reused, remanufactured or recycled and therefore retained in the economy for as long as possible, along with the resources they are made of, and of minimizing the generation of waste, which can significantly contribute to sustainable production and consumption of plastics.

This Commission notes that several suggestions on how to achieve product streamlining have recently been put forth [1510]. They include developing design standards for plastics to simplify their chemical composition and increase compatibility with end-of-life management [43], for example, through removing toxic chemicals from plastics and replacing them with more environmentally friendly materials, redesigning clothing to reduce fiber shedding and redesigning tires to reduce PM and microfiber release.

#### 3. Addressing Harmful Externalities

##### 3.1 Mandatory Extended Producer Responsibility (EPR) Frameworks

**This Commission recommends inclusion in the Global Plastics Treaty of requirements for Parties to enact and enforce legislation and policy frameworks on EPR**.

The Treaty could prescribe minimum global standards for EPR frameworks to be implemented at the national level by treaty parties. EPR legislation makes plastic producers and the manufacturers of plastic products legally and financially responsible for the safety and end-of-life management of all the materials they produce and sell. By making plastic producers responsible for costs that until now they have externalized and shifted onto governments, taxpayers, and the general public, the goal of EPR is to incentivize change within industry. By analogy, liability protocols have been adopted for releases of hazardous substances [1527], wastes [1528], and genetically modified organisms [1529] under other major multilateral conventions.

At a minimum, EPR programs require producers of plastic products to either take back these products at the end of their useful life (with take back encouraged in some instances through deposit fees) or to cover costs of waste management and clean-up (see examples in Boxes 7.2, 7.3, 7.4) [1530]. For example, Canada has had a national EPR policy in place since 2009 that has been implemented in at least five provinces [1531]. Container deposit laws in multiple countries provide additional examples of successful EPR strategies (Box 7.4).

**Box 7.2 Extended producer responsibility (EPR) and e-waste.**

**EPR and E-Waste.** Electric-powered and electronic products contain large quantities of many types of plastic. Substantial opportunities exist to reduce both plastic production and the generation of e-waste by requiring that all electric-powered and electronic products be repairable, that their components be reusable, and that manufacturers take their products back at the end of their useful life for reuse, remanufacturing, recycling, or safe disposal.

Such requirements will make manufacturers of electric-powered and electronic products responsible for the costs of e-waste handling that they currently externalize and shift on to state and local governments and vulnerable populations. It will also make them responsible for the large volumes of e-waste that they currently send to landfills in high-income countries and into e-waste dumpsites in LMICs.

Product design standards that require easy disassembly of all electric and electronic products and the repair and reuse of components are key to achieving this goal. They should include standardization of the plastics used in electrical appliances and electronic goods. The European Commission has already proposed design requirements mandating most of these features [1532].

“Right to Repair” laws are a further strategy for reducing e-waste. These laws prohibit manufacturers from placing limitations on access to repair materials such as parts, tools, diagnostics, and programming such as firmware. In July 2017, the European Parliament approved a recommendation that Member States should pass laws giving consumers the right to repair their electronics. Likewise, the British government introduced a “Right to Repair” law that went into effect on July 1, 2021.

**Box 7.3 Extended producer responsibility (EPR) for abandoned, lost, and discarded fishing gear (ALDFG).**

**EPR and ALDFG.** Abandoned, lost, or discarded fishing gear constitutes a significant portion of ocean plastics [551]. Entanglement in ALDFG is well known to endanger marine mammals, turtles, seabirds, and some fishes and poses a threat to ecosystems [505,567,722]. ALDFG may also serve as an ongoing source of marine leakage of harmful chemicals and MPs [1533,1534].

Several EPR provisions pertaining to fishing gear are already in place in the EU, where Directive 2019/904 requires producers of fishing gear containing plastic to cover the costs of 1) the collection of waste gear and 2) awareness raising measures to prevent and reduce the abandonment of gear at sea. EU Member States are required to monitor and assess compliance [334]. The measure was built on earlier directives, such as that of 2008 obliging national minimum annual collection rates of gear containing plastic for recycling [1535] and of 2009 requiring that gear be tagged with an external identification number, and that the master of the vessel attempt to retrieve lost gear as soon as possible, reporting losses to authorities of the flag state within 24 hours [1536].

An international program of EPR can incorporate and expand on these provisions, for instance by implementing a universal system of equipment registration, and by marking registered gear with acoustic transponder tags, which can be used to ensure that lost gear is tracked and, when possible, retrieved. Such tags are relatively inexpensive and are already in use in fisheries in Southwest England [1537]. Commercial fishers who use fish aggregating devices employ similar devices, and these have been successfully utilized in demonstration projects by bodies such as the European Climate Infrastructure and Environment Executive Agency to locate and retrieve lost nets [1538,1539].

Technologies such as these, in combination with deposit schemes or regulations that impose financial penalties for discarded or lost equipment, can introduce stronger systems of accountability. Drawing on the EU’s minimum annual collection rates, international requirements can be instituted requiring manufacturers to offer take-back credits, and to ensure that new gear be made using a percentage of recycled materials. A certification or labelling scheme can be initiated to identify products made from recycled fishing gear, thus conferring a higher value [1537].

**Box 7.4 The effectiveness of bottle deposit laws.**

**Container Deposit Laws.** Deposit laws in multiple countries for bottles, cans and other containers provide an example of a highly successful EPR strategy implemented at national level. Under EC Directive 2019/904, EU Member States are encouraged to establish bottle bills that incentivize the collection and reuse of containers, including plastic bottles [334]. Such bills are already in place in many countries worldwide [1540].

In 2022, the 11 Canadian provinces with deposit laws had an average return rate of 74%, the 13 EU countries averaged 90%, and the 13 jurisdictions in Oceania averaged 69%. All of these rates are far higher than the global average plastic recycling rate of about 9%. Across the US, polyethylene terephthalate (PET) plastic beverage containers without a deposit were recycled at a rate of only 17%, in contrast to a nationwide average recycling rate of 57% for PET bottles with a deposit [1541]. In 2019, Oregon and Michigan—the two US states with 10¢ deposits—had overall redemption rates of 86% and 89% respectively. In contrast, states such as Massachusetts and Connecticut, which have 5¢ deposits, had redemption rates of 43% and 44%.

In the European Union, the five best performing Member States with deposit schemes for PET bottles (Germany, Denmark, Finland, the Netherlands, and Estonia) reached an average collection rate for PET of 94% in 2014 [1532].

A striking example of the benefit that could have been delivered by EPR is seen in the case of plastic microbeads in cosmetics. The patent for the use of plastic microbeads in cosmetics was filed decades ago. If EPR been in place at that time it would have likely prevented the hundreds of thousands of tons of totally avoidable environmental contamination that have resulted from use of these materials.

EPR programs can be coordinated with national and state strategies to reduce virgin plastic production. The ultimate goal of this suite of interventions is to make the plastic supply chain more circular and less linear, thus reducing need for virgin plastic production and slowing the accumulation of plastic waste.

##### 3.2. Explore Listing Plastic Polymers as Persistent Organic Pollutants (POPs) under the Stockholm Convention

**This Commission encourages inclusion in the Global Plastic Treaty of a provision calling for exploration of listing some plastic polymers as POPs under the Stockholm Convention** [187].

Plastics meet many of the cardinal criteria for listing as POPs:

- All plastic polymers are synthetic chemicals (Section 2).
- Macroplastics as well as MNPs are resistant to natural degradation and can persist in the environment for many decades [1542,1543]. Persistent materials such as plastics will continue to accumulate in the environment for as long as they are released, reaching high levels. Toxic effects that would not otherwise be evident could emerge [1544].
- Plastic can be transported over long distances, especially through the oceans (Section 3).
- Although the larger MP particles (>10 µm) that result from the environmental breakdown of plastic waste appear not to undergo biomagnification themselves, there is potential for bioaccumulation and biomagnification of the smaller MP and NP particles (<10 µm), which cannot yet be reliably measured.

Based on the foregoing considerations, this Commission urges the Parties to the Stockholm Convention to consider listing of some plastic polymers as POPs and urges the INC expressly to call for exploration of this action.

This Commission makes two further observations in regard to the Stockholm Convention:

- A great strength of the Stockholm Convention is that it reaches into domestic production and use. It consequently is a superior model for the Global Plastics Treaty than either the Basel or Rotterdam Conventions, which primarily address international trade. Under the Stockholm Convention, domestic production, use, importation, and exportation of listed POPs can be eliminated or restricted. The 2013 Minamata Convention on Mercury [1545] is another comprehensive instrument, which requires parties to implement policies and measures to control mercury throughout its life cycle, including reductions across various products, processes, and industries where mercury is used, released, or emitted.
- The Stockholm Convention also has limitations that should be overcome in the new Global Plastics Treaty. For example, the process of listing new substances is slow and cumbersome, bound by a consensus decision-making process, often influenced by least-common-denominator results. Thus, since entry into force in 2004, only 20 new substances or groups of substances have been listed, most recently in 2022.

#### 4. Addressing the disproportionate impacts on vulnerable and at-risk populations

##### 4.1 Consideration of Vulnerable and At-Risk Populations in Developing Globally Binding Controls

**This Commission recommends that protection of human health and well-being, and especially protection of the health of vulnerable and at-risk populations, be a paramount consideration in developing globally binding controls addressing the harms caused by plastics under the Global Plastics Treaty**.

The UNEA resolution on the Global Plastics Treaty specifically identifies human health in its preamble. Building on this foundation, the operational provisions of the Global Plastics Treaty should be crafted to assure that protection of human health is a central goal of the instrument. The UNEA remit specifically instructs the INC to adopt a full life cycle approach to addressing the health impacts of the global plastics problem.

The groups at greatest risk of harms to health caused by plastic across the life cycle are infants, children, pregnant women, workers, Indigenous populations, and persons living in “fenceline” communities adjacent to plastic industries (Sections 4 and 6). Protection of the health of these vulnerable and at-risk groups is ethically well justified, and measures crafted to protect their health will safeguard the health of entire populations.

Consistent with the directive of the UNEA resolution, it is essential that affected communities, civil society organizations, Indigenous populations, environmental justice organizations, the scientific community, faith-based organizations, and LMIC representatives such as waste-pickers contribute to the treaty negotiations. People living near plastic production and waste facilities in both high-income countries and LMICs have a particularly important voice that needs to be heard and factored into decision-making. Their participation will help ensure that the Global Plastics Treaty includes measures specifically designed to address the disproportionate harms that plastics impose on these populations. Liberal standards for admission of accredited non-governmental observers to negotiations under UN auspices for major conventions on climate, biodiversity, international trade in wastes, POPs, stratospheric ozone protection, and others is now the global good practice standard.

##### 4.2 Strengthening Restrictions on Transnational Export of Plastic Waste

**This Commission recommends a strong interface between the Global Plastics Treaty and the Basel and London Conventions to support ongoing management of hazardous plastic waste**.

The provisions of the Global Plastics Treaty will need to build upon and reinforce the work of the Basel Convention on the Control of Transboundary Movements of Hazardous Wastes and Their Disposal.

Having entered into force in May 1992, the Basel Convention [1546] was designed to reduce the movement of hazardous waste between nations, and specifically to discourage wealthier countries from exporting unsafe waste to lower-income nations. In 2019, this Convention was updated by the Plastic Waste Amendment, which modified three annexes to include certain plastic wastes [1528]. This amendment requires prior written consent of the importing country for most categories of plastic waste, including e-waste. A companion amendment [1547] has the effect of banning the export of contaminated and highly mixed plastic waste from the OECD countries, the EU, and Lichtenstein to LMICs.

Despite the strong and well-crafted provisions of the Basel Convention and its Plastic Waste Amendment, massive amounts of plastic waste continue to flow into the world’s least developed countries where it degrades environments, harms human health, and deepens social injustices.

To address this continuing crisis, this Commission recommends that the Global Plastics Treaty include a provision calling for collaboration with the Basel Convention to strengthen enforcement of Basel Convention’s regulations and increase awareness among national leaders of their power to refuse unwanted shipments of plastic waste under the provisions of the Basel Convention.

Additionally, recognizing that The Basel Convention is primarily limited in scope to international trade in wastes, this Commission notes that the Global Plastics Treaty needs to penetrate to the domestic level, establishing globally agreed-upon standards for production, use, and disposal of plastics within countries and not only on shipments that enter into international trade.

The London Convention and Protocol [1549] can and should be mobilized further to address the problem of the dumping of plastics into the marine environment.

The Rotterdam Convention [1548] creates a similar prior-informed-consent procedure for chemicals and pesticides in international trade, potentially including many plastics or their precursors and starting materials. The parties to the Rotterdam Convention should consider a plastics amendment analogous to that under the Basel Convention. Even without such an instrument, the Rotterdam Convention can be mobilized to address an important additional component of the global plastics problem.

#### 5. Creation of a Permanent Science Policy Advisory Body

The governing UNEA resolution directs the INC to consider “the possibility of a mechanism to provide policy-relevant scientific and socioeconomic information and assessment related to plastics pollution.”

Currently, in parallel with negotiations on the plastics treaty, the global community is working on establishing an intergovernmental, independent science-policy panel on chemicals, waste and pollution prevention, with the ambition to establish it by 2024. This body will need to interface closely with the Plastics Treaty and appropriate frameworks for that interface should be established. Negotiators may consider whether the science-policy functions for the Plastics Treaty could be realized through this new science-policy panel on chemicals, waste, and pollution prevention, or would be better served by a dedicated science-policy body established under the Treaty (or some combination of both).

**This Commission recommends establishment of a dedicated Permanent Science Policy Advisory Body for the Global Plastics Treaty, with core functions of providing scientifically rigorous, unbiased advice to the Treaty implementation through close engagement, on an ongoing basis, with the global academic community, national research institutes, and national and local policy makers**.

Such a body should be transdisciplinary in scope and include expertise in the natural, economic, and social sciences as well as regional expertise and Indigenous knowledge. It will be essential that this body is shielded from special interests.

The overall priority of such a dedicated Permanent Science Policy Advisory Body could be to guide Treaty Parties in evaluating which solutions are most effective in reducing plastic production and consumption, curbing the generation of plastic waste, enhancing plastic waste recovery and recycling, and ensuring proper disposal of plastic waste. This Body could also assess trade-offs among proposed solutions, evaluate unintended consequences to interventions, and evaluate safer alternatives to current plastics (see Box 7.5).

**Box 7.5 Independent evaluation of evidence on the efficacy of solutions is critical for the Global Plastics Treaty.**

**The Need for Independent Evaluation of Evidence on the Efficacy of Solutions.** It will be essential that solutions proposed to the plastics crisis be subject to careful review and due diligence to avoid “regrettable substitutions.” Some examples of inadequately vetted solutions that have been found, on review, to aggravate global plastics crisis include:

- Carrier bags marketed as “biodegradable” that fail to explain the context required for meaningful degradation to occur, and that actually remain fully functional after several years at sea or buried in soil [1550].
- Devices that claim to reduce the release of microfibers from laundering, but fail to deliver any significant reductions [1551].
- Devices marketed for the removal of litter from ports and harbors, that fail to remove much plastic, but instead capture large quantities of seaweed and kill juvenile fish [1552].

It is critical that we learn from these mistakes and take a far more precautionary approach, e.g., based on EPR. This transition needs to start now; it needs to be evidence-based; and it needs to be enshrined in all approaches intended to address the issue of plastic production [60].

It is also critical to adopt an evidence-based approach to identify which aspects of the plastic crisis are best addressed by actions at an international versus the national level. A case study is seen in the release of MP fibers from textiles. Three main intervention points exist to reduce microfiber shedding:

1) increasing the availability and quality of wastewater treatment [1553],
2) fitting filters to washing machines [1551], and
3) reducing the shedding of fibers via design changes to fabric and yarn [1554].

Options 1 and 2 are more attractive in the Global North because they can be implemented at a national scale. For example, new legislation in France will mandate filters on washing machines. By contrast, Options 1 and 2 will not be very effective in the Global South, where many populations do not have the benefit of washing machines or advanced wastewater treatment. Option 3, improved fabric design, which could be mandated by international legislation under the Global Plastics Treaty, appears to be effective in reducing microfiber shedding in all countries at every level of income, because 50% of all microfiber emissions occur while garments are being worn rather than while they are being washed [1555]. Improved fabric design therefore has far greater potential to address the issue of microfiber shedding than any “downstream” solution [1555].

Specific functions of this Body could be to:

- Track global and national trends in plastic production, recycling, transnational export, environmental leakage, and plastic-associated GHG emissions;
- Coordinate, assist, and provide guidance in the crafting of national EPR legislation/policy frameworks;
- Provide ongoing guidance to Treaty parties for the refinement of health-protective standards for plastic-associated chemicals;
- Provide guidance on effective strategies for plastic waste recovery, recycling, and disposal;
- Summarize information on the heretofore externalized and undercounted health and environmental costs of plastic production and pollution, including impacts on the marine environment;
- Examine the health and environmental costs of products proposed as replacements for plastics to assure that they entail no net increases in production, use and disposal externalities; and
- Support robust aquatic, land, and air-based monitoring programs for primary and secondary MPs.

#### 6. Additional Recommendations for Harm Reduction

This Commission is very clear in our view that the most effective strategies for slowing the accumulation of plastic waste and reducing the harms associated with plastics across its life cycle are “upstream” solutions that address the root causes of the plastic crisis. They include:

- Reducing plastic production through an agreed-upon production cap;
- Enacting and enforcing EPR legislation;
- Banning production and sale of non-essential single-use plastics; and
- Redesigning plastics to simplify their chemical composition and increase compatibility with end-of-life management [43].

These “upstream” solutions need to be supplemented by complementary, “downstream” strategies such as effective recovery, recycling, and reuse.

The Commission makes the following comments regarding some existing and emerging, expensive, and unproven downstream waste management methods from the perspective of minimizing harm to human health and the environment:

- **Open burning of plastic waste in landfills and municipal waste dumps is the most dangerous approach to plastic waste disposal and should be prohibited in all countries under the Global Plastic Treaty.** Open burning of plastic releases metals and toxic chemicals, including lead, mercury, arsenic, PM_2.5_, carbon monoxide, NO_x_, arsenic, PCDD/Fs, PCBs, PBBs, PAHs, and pyroplastics (a complex mixture of plastic transformation products) to air, water, soil, and ultimately the ocean [679,1355]. The ash resulting from plastic burning is also toxic and contains many of these same pollutants, which can leach into the soil and contaminate groundwater [384].
- **The full scope of risks associated with chemical and thermal conversion (also known as “advanced recycling,” “pyrolysis,” “gasification,” “hydrothermal conversion,” and “waste-to-energy” processes) remain unknown.** All of these technologies involve super-heating or burning plastic waste in furnaces or kilns, or they use chemical reactors to break chemical bonds and produce smaller molecules. At present, these are mostly small-scale enterprises of limited capacity and unproven reliability that are able to cope with only a small fraction of the millions of tons of virgin plastic produced each year [322]. “Upcycling” is a term used by some to describe depolymerization of plastic waste into smaller molecules that can be used to make the same or different higher value materials—higher value than those resulting from mechanical recycling. While these approaches seem promising, none of them are yet proven effective at any useful scale.

This Commission notes the following risks associated with these technologies:

- Heating and burning of plastics that contain chlorine (e.g., PVC) and bromine (e.g., BFRs) can release highly toxic halogenated dioxins and furans and other hazardous pollutants such as benzene into the atmosphere through their stacks unless combustion temperatures and waste gas filtration devices are meticulously well maintained at all times [1556]. The residue that remains after combustion may also contain these toxic chemicals.
- Chemical and thermal conversion operations are disproportionately sited in low-income communities and communities of color, where they exacerbate environmental injustices [1557].
- Because they require high heat, chemical, and thermal conversion processes are energy-intensive with high energy costs and potential to produce abundant GHG emissions [1558,1559].

This Commission advises countries to approach these emerging, expensive, and unproven technologies with great caution and to undertake thorough, independent assessments of their environmental impacts prior to any adoption. Any implementation of these technologies should proceed step-wise and be closely scrutinized.

A final consideration in regard to chemical and thermal conversion is that investments into these costly yet unproven technologies will divert funding away from proven effective strategies. Investments made in chemical and thermal recycling thus have potential to derail efforts to address the root causes of the global plastics crisis.

### Recommendations for Research

While much actionable information is already available on plastics’ hazards to human and planetary health, gaps in knowledge remain and additional research is needed to better safeguard health.

Governments will likely support much of this research using public funds. An additional source of support could be a carbon tax levied against the industries that produce the coal, oil, and gas feedstocks used in plastic manufacture. Such a tax would complement EPR fees paid by the manufacturers of plastic products to prevent the accumulation of plastic waste. Like EPR, it would have the effect of making the producers of fossil carbon responsible for the harms to human health and the global environment caused by the materials they produce and sell.

#### 1. Operational Research

**This Commission recommends urgent investment by national governments and major foundations in research into practical solutions to the global plastic crisis**.

This research will need to determine which solutions are most effective and cost-effective in the context of particular countries and assess the risks, benefits, and trade-offs of proposed solutions. It will need additionally to evaluate any potential unintended consequences of interventions. This Commission recommends that there be close coordination between research on these topics at the national and international level and the Permanent Science Policy Advisory Body that this Commission suggests be established under the treaty.

#### 2. Oceanographic and Environmental Research

**This Commission recommends increased oceanographic and environmental research to**:

- Better measure and understand the concentrations and health impacts of plastics <10µm on marine species;
- Monitor levels of plastic regardless of size (macro-, micro- and nano-) or origin (primary or secondary) and assess their fate throughout the global ocean (see Section 3); and
- Better measure and monitor the impacts of plastic-associated chemicals on marine species.

#### 3. Biomedical and Human Research

**This Commission recommends increased research on the human health effects of plastic-associated chemicals (see Section 4) to**:

- Better assess human exposure to a wider range of plastic chemcials;
- Develop enhaced toxicologial techniques, including high-throughput and *in silico* techniques, to screen currently untested plastic-associated chemicals for toxicity;
- Undertake longitudinal biomonitoring studies to assess plastic chemical exposure in human populations within both developed countries and LMICs, and including high-risk populations such as plastic production workers, residents of “fenceline” communities, and waste-pickers; and
- Undertake longitudinal epidemiological studies in human populations within both developed countries and LMICs, including high-risk populations to assess the impacts of plastic chemical exposures on human health.

**This commission also recommends increased research on the potential human health effects of MNPs (see Section 4) to**:

- Refine and validate existing measurement techniques and develop novel techniques for quantifying both MPs and NPs in biological and environmental media;
- Develop rigorous contamination control methodologies and equipment for use during sample collection, storage, processing and measurement;
- Undertake longitudinal biomonitoring studies in human populations, including high-risk populations, to assess MNP exposure; and
- Undertake longitudinal observational studies in human populations, including high-risk populations, to assess the impacts of MNPs exposure on human health.

### Recommendations for Education and Outreach

#### 1. Education

**This Commission recommends that plastics researchers and policymakers engage educators as partners to inform the next generation of consumers and decision-makers about the health and environmental impacts of plastics**.

Because plastics’ manufacture, use, disposal, and pollution are globally pervasive and growing, it is imperative that today’s youth be educated about the plastic world that they will inherit and have to address as the consumers and leaders of tomorrow. Youth need to have as keen an appreciation of the dangers of chemical and plastic pollution as they do of climate change. Educators can instill in students the importance of a healthy body and a healthy environment and help students grow into well-informed consumers and members of society who have the power to make change with their individual voices, choices, and actions.

#### 2. Outreach to Impacted Communities

**This Commission recommends that plastics researchers and policymakers engage their local communities to provide accurate information about the plastics life cycle, the health and environmental impacts of plastics, and the alternatives to unnecessary plastic products**.

Although plastics are materials upon which modern society is heavily reliant, substantial outreach is required to inform the general populace about the dangers of pervasive use of unnecessary plastic products and the negative impacts these products have on human and environmental health from production through use, disposal, and pollution of the environment. It is critical that accurate and timely information be shared, and that concrete, actionable alternatives are co-created according to the specific needs and circumstances of each local community.

### Recommendations for the Medical, Public Health, and Scientific Communities

**This Commission recommends that the medical, public health, and scientific communities take an active role in reducing use of unnecessary single-use plastic and raising awareness about impacts of plastic-associated chemicals on human and environmental health**.

While the oceanographic and marine biology communities have been aware of plastic’s negative impacts on the environment since the 1970s [1,490], the medical and public health communities have until now not been widely aware of plastics’ impacts on human health [284,1560]. This is not surprising, given that the science in this area is still emerging and the majority of reports on plastics’ hazards have appeared in environmental and oceanographic journals and in government agency publications not widely read by physicians, nurses, and public health professionals. Moreover, plastic and chemical pollution have been worsening quietly, while the world’s attention has been focused on climate change.

Today, however, as knowledge of plastics’ many harms to human and planetary health increases and becomes more widely available, physicians, nurses, and public health professionals have an opportunity to lead the global effort to reduce these hazards and to protect the health of their patients.

Health professionals can educate themselves and their patients about plastics and their hazards. They can take a leadership role in reducing plastic use and plastic waste generation in hospitals and health care facilities. The health care sector generates substantial volumes of plastic waste, and depending on the medical facility, plastics comprise between 20–65% of all waste generated [284]. While some plastics are essential in health care, many are not, and careful review of inventories and supply chains has potential to greatly reduce plastic use. Examples include going back to cotton rather than plastic sheets and using sterilizable, reusable surgical instruments instead of single-use plastic devices.

Public health agencies can increase support for toxicological and epidemiological research into the health hazards of plastics and plastic additives. They can launch and support large-scale, multi-year human biomonitoring programs and observational studies. They can increase their educational offerings on chemical and plastic pollution.

Medical societies and public health organizations are uniquely well positioned to educate elected officials about plastics’ harms to human health and the environment and to advocate governments at every level to reduce plastic production, use, and disposal to protect the health of their patients. They can advocate for such goals as reductions in plastic production; moratoria on fracking and on the construction of cracker plants, pipelines, and compressor stations; restrictions on single use plastics; and passage of EPR legislation.

By pointing out to elected officials the increasingly well-documented links between plastics and harms to human health, and by noting that actions to control plastic production will also help control climate change, prevent pollution, and save tax dollars, doctors, nurses, public health professionals, and scientists are in a powerful position to oppose the forces that call for endless, unchecked increases in plastic production. These trusted advocates are uniquely well positioned to catalyze enduring action to safeguard human health, protect the planet, and advance the common good.

### Conclusion

The Minderoo-Monaco Commission on Plastics and Human Health finds that plastics are both a boon to humanity and a stealth threat to human and planetary health. Plastics convey enormous benefits, but current linear patterns of plastic production, use, and disposal with little attention paid to sustainable design or safe materials and a near absence of recovery, reuse, and recycling are responsible for grave harms to health, widespread environmental damage, great economic costs, and deep societal injustices. These harms are rapidly worsening.

While there remain gaps in knowledge about plastics’ harms and uncertainties about their full magnitude, the evidence available today demonstrates unequivocally that these impacts are great and that they will increase in severity in the absence of urgent and effective intervention at global scale. Manufacture and use of essential plastics may continue. But reckless increases in plastic production, and especially increases in the manufacture of an ever-increasing array of unnecessary single-use plastic products, need to be curbed.

Global intervention against the plastic crisis is needed now, because the costs of failure to act will be immense.

## Additional File

The additional file for this article can be found as follows:

10.5334/aogh.4056.s1 Supplementary file. Abbreviations and Glossary.

## Footnotes

The intake fractions used to measure premature mortality due to ambient PM_2.5_ reflect outdoor exposures in rural areas, rather than indoor exposures.

The present discounted value of lifetime output, both market and non-market, in the US, is approximately 1.375 million (2015 Int$) [1383] for a 40–44 year-old, about 25% of our transferred VSL for the US ($5.42 million 2015 Int$).

Eighteen of the countries for which we have PM_2.5_ deaths do not have VSL estimates because their per capita GNIs are not published by the World Bank. These countries account for 8,575 deaths, whose value is omitted from Table 5.4.

To put these numbers in context, the sizes of the 2010 birth cohorts are: US (4.0 million); EU (5.2 million); Canada (0.38 million).
