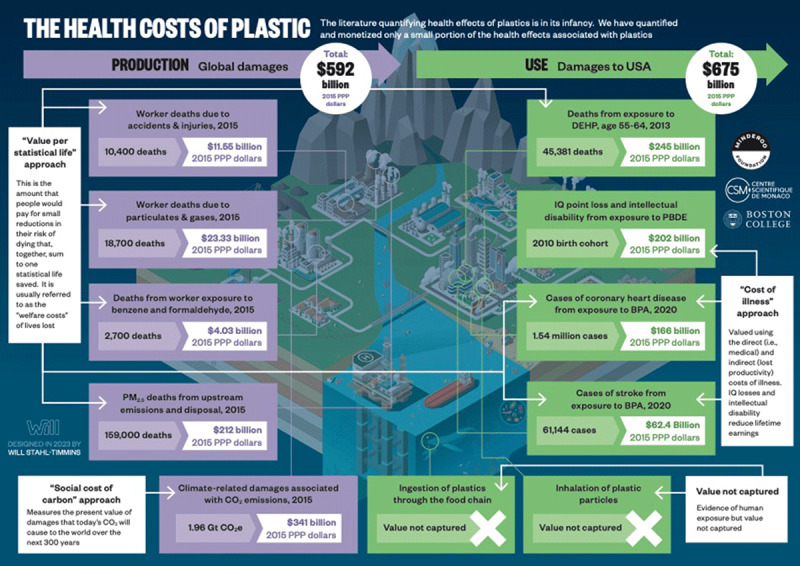# Correction: The Minderoo-Monaco Commission on Plastics and Human Health

**DOI:** 10.5334/aogh.4331

**Published:** 2023-10-11

**Authors:** Philip J. Landrigan, Hervé Raps, Maureen Cropper, Caroline Bald, Manuel Brunner, Elvia Maya Canonizado, Dominic Charles, Thomas C. Chiles, Mary J. Donohue, Judith Enck, Patrick Fenichel, Lora E. Fleming, Christine Ferrier-Pages, Richard Fordham, Aleksandra Gozt, Carly Griffin, Mark E. Hahn, Budi Haryanto, Richard Hixson, Hannah Ianelli, Bryan D. James, Pushpam Kumar, Amalia Laborde, Kara Lavender Law, Keith Martin, Jenna Mu, Yannick Mulders, Adetoun Mustapha, Jia Niu, Sabine Pahl, Yongjoon Park, Maria-Luiza Pedrotti, Jordan Avery Pitt, Mathuros Ruchirawat, Bhedita Jaya Seewoo, Margaret Spring, John J. Stegeman, William Suk, Christos Symeonides, Hideshige Takada, Richard C. Thompson, Andrea Vicini, Zhanyun Wang, Ella Whitman, David Wirth, Megan Wolff, Aroub K. Yousuf, Sarah Dunlop

**Affiliations:** 1 Global Observatory on Planetary Health, Boston College, Chestnut Hill, MA, US; 2 Centre Scientifique de Monaco, Medical Biology Department, MC; 3 Economics Department, University of Maryland, College Park, US; 4 Global Observatory on Planetary Health, Boston College, US; 5 Minderoo Foundation, AU; 6 Monterey Bay Aquarium, US; 7 Biology Department, Boston College, US; 8 University of Hawai’i Sea Grant College Program, US; 9 Beyond Plastics, Bennington College, US; 10 Université Côte d’Azur; 11 Centre Hospitalier, Universitaire de Nice, FR; 12 European Centre for Environment and Human Health, University of Exeter Medical School, UK; 13 Centre Scientifique de Monaco, Marine Biology Department, MC; 14 Norwich Medical School, University of East Anglia, UK; 15 Biology Department, Woods Hole Oceanographic Institution, US; 16 Woods Hole Center for Oceans and Human Health, US; 17 Department of Environmental Health, Universitas Indonesia, ID, US; 18 Research Center for Climate Change, Universitas Indonesia, ID; 19 College of Medicine and Health, University of Exeter, UK; 20 Department of Marine Chemistry and Geochemistry, Woods Hole Oceanographic Institution, US; 21 Department of Biology, Woods Hole Oceanographic Institution, US; 22 United Nations Environment Programme, KE; 23 Department of Toxicology, School of Medicine, University of the Republic, UY; 24 Sea Education Association, US; 25 Consortium of Universities for Global Health, US; 26 Nigerian Institute of Medical Research, Lagos, Nigeria; 27 Lead City University, NG; 28 Department of Chemistry, Boston College, US; 29 University of Vienna, Austria and University of Plymouth, UK; 30 University of Massachusetts Amherst, US; 31 Laboratoire d’Océanographie de Villefranche sur mer (LOV), Sorbonne Université, FR; 32 Chulabhorn Research Institute (CRI), TH; 33 School of Biological Sciences, The University of Western Australia, AU; 34 Biology Department and Woods Hole Center for Oceans and Human Health, Woods Hole Oceanographic Institution, US; 35 Superfund Research Program, National Institutes of Health, National Institute of Environmental Health Sciences, US; 36 Laboratory of Organic Geochemistry (LOG), Tokyo University of Agriculture and Technology, JP; 37 International Marine Litter Research Unit, University of Plymouth, UK; 38 Theology Department, Boston College, US; 39 Technology and Society Laboratory, WEmpa-Swiss Federal Laboratories for Materials and Technology, CH; 40 Boston College Law School, US

**Keywords:** plastic life cycle, human health, ocean health, microplastics, plastic additives, environmental health

## Abstract

This article details a correction to: Landrigan PJ, Raps H, Cropper M, et al. The Minderoo-Monaco Commission on Plastics and Human Health. *Annals of Global Health*. 2023; 89(1): 23. DOI: https://doi.org/10.5334/aogh.4056.

## Correction

In Landrigan, Raps, and Cropper et al. [1] our estimate of annual DEHP-associated mortality in the United States in Chapter 5 of the Minderoo-Monaco Commission on Plastic and Human Health—90,762 deaths per year among 55–64-year-olds—is based on data reported in Table 4, “Phthalate-attributable mortality and associated lost economic productivity in the US, 2014” of the article by Trasande, Liu and Bao [2].

On re-examination of the Trasande et al. article, and examination of the source data for the population of 55–64 year olds from the US Census Bureau, it appears that their estimate, interpreted to be an annual estimate, is based on population data for both 2013 and 2014 combined; hence their estimate of 90,762 annual deaths attributable to DEHP and our corresponding cost estimate of $490 billion is the sum of two years of deaths and corresponding damages.

An updated version of Figure 5.1 below displays adjusted estimates of the dollar sums and deaths from 2013 alone, based on the re-examination of Trasande et al.

**Figure:**