# Supplementary material for: The Minderoo-Monaco Commission on Plastics and Human Health
Source: Ann Glob Health. 2023 Mar 21;89(1):23. doi: 10.5334/aogh.4056 (PMC10038118; doi:10.5334/aogh.4056)
Supplement: Supplementary file. — Abbreviations and Glossary. [file agh-89-1-4056-s1.pdf]

ABBREVIATIONS AND GLOSSARY

ALDFG	abandoned, lost, or otherwise discarded fishing gear
ATBC	acetyl tributyl citrate
BBP	butyl benzyl phthalate (also commonly abbreviated as BBzP)
BFRs	brominated flame retardants
BHT	butylated hydroxytoluene
BPA	bisphenol A
BPF	bisphenol F
BPS	bisphenol S
BTEX	benzene, toluene, ethylbenzene and xylene
BUVS	benzotriazole-type UV stabilizer
BzP	benzophenones
CHD	coronary heart disease
CO_2	carbon dioxide
CO_2e	carbon dioxide equivalent
DEHA	di(2-ethylhexyl) adipate
DEHP	di(2-ethylhexyl) phthalate
DINCH	di-isononyl cyclohexane-1,2-dicarboxylate
DINP	di-isononyl phthalate (also commonly abbreviated as DNP)
ECHA	European Chemicals Agency
EDCs	endocrine disrupting chemicals
EPA	Environmental Protection Agency
EPR	extended producer responsibility
EU	European Union
GDP	gross domestic product
GHG	greenhouse gas
GI	gastrointestinal
GNI	gross national income
Gt	gigaton (1 billion metric tons)
HBCDD	hexabromocyclododecane
HDPE	high-density polyethylene
Int$	international dollar (equivalent to US dollar)
INC	Intergovernmental Negotiating Committee
IV	intravenous
LDPE	low-density polyethylene
LMIC/s	low-and-middle income country/ies
LNG	liquified natural gas
MNP	micro- and nano-plastic particles
MPs	microplastics
Mt	megaton (1 million metric tons)
NHANES	National Health and Nutritional Examination Survey
NHS	National Health Service
NIAS	non-intentionally added substances
NO_x	oxides of nitrogen
NPs	nanoplastics

OECD	Organisation for Economic Co-operation and Development
OPEs	organophosphate esters
OPFRs	organophosphorus flame retardants
OR	odds ratio
PA	polyamide
PAHs	polycyclic aromatic hydrocarbons
PBBs	polybrominated biphenyls
PBDEs	polybrominated diphenyl ethers (also commonly abbreviated as BDE)
PBT	persistence, bio-accumulation potential and toxicity (or persistent, bioaccumulative and toxic)
PC	polycarbonate
PCBs	polychlorinated biphenyls
PCDDs	polychlorinated dibenzo-p-dioxins
PCDD/Fs	polychlorinated dioxins and dibenzofurans
PE	polyethylene
PET	polyethylene terephthalate
PFAS	per- and poly-fluoroalkyl substances
Pg	petagram
PLA	polylactic acid
PM	particulate matter (number after PM indicates size in microns. $PM_{2.5}$ are particulates with a diameter of 2.5 micrometers or less and PM_{10} are particulates with a diameter of 10 micrometers or less)
POPs	persistent organic pollutants
PP	polypropylene
PS	polystyrene
PUR	polyurethane
PVC	polyvinyl chloride
REACH	Registration Evaluation Authorization and Restriction of Chemicals
ROS	reactive oxygen species
RoW	rest of world
SCC	social cost of carbon
SEJ	social and environmental justice
SO_2	sulfur dioxide
SO_x	oxides of sulfur
TRL	technology readiness level
UN	United Nations
UNEA	United Nations Environmental Assembly
UNEP	United Nations Environment Programme
UV	ultraviolet
VOCs	volatile organic compounds
VSL	value per statistical life
WHO	World Health Organization

Additives	Chemicals that are incorporated into the plastic polymer matrix to impart a wide range of properties such as durability (antioxidants, light stabilizers, heat stabilizers, biocides and flame retardants), strength (glass fibres and carbon fibres), flexibility (plasticizers) and color.

Adsorbed chemicals	Chemicals from the surrounding environment attracted to and accumulating on the surface of a material
Bioaccumulation	Increased concentration of a chemical in a biological organism over time during its lifespan, compared to the chemical's concentration in the surrounding environment.
Biodegradation	Breakdown of materials by microorganisms, such as bacteria and fungi, into environmentally acceptable products such as water, carbon dioxide, and biomass.
Biomagnification	Increased concentration of a chemical due to environmental and food exposure with increasing trophic level.
Carbon feedstocks	Carbon-containing raw materials and/or fuel used in manufacturing products, typically derived from fossil fuels such as coal, gas and oil.
Environmental justice community	A neighborhood or community subjected to a disproportionate burden of environmental hazards and/or experiences a significantly reduced quality of life due to environmental harms and risks relative to surrounding or comparative communities.
Fenceline community	A neighborhood that is within a distance from a company or industrial project short enough to be directly affected by the noise, odors, chemical emissions, traffic, and operations of the company.
Macroplastics	Typically categorized as plastics >5 mm in size. Includes mesoplastics.
Microplastics	Plastic particles <5 mm in size.
Monomers	Small molecules that can react together or with other monomer molecules to form a larger polymer chain or a three-dimensional network polymer in a process called polymerization.
Nanoplastics	Plastic particles typically defined either as <1 µm or <100 nm.
Plastic chemicals	Includes monomers, polymers, chemicals added to assist manufacturing and processing of the plastic materials (processing aids), chemicals added to impart properties to the plastic materials (additives), as well as non-intentionally added substances such as impurities, by-products, breakdown products and contaminants.
Plastics	Synthetic materials that have the capability of being molded or shaped. They are made of polymers and a number of plastic chemicals.
Polymers	Natural or synthetic substances composed of very large molecules, called macromolecules, that are multiples of simpler chemical units called monomers.
Primary microplastics	Defined by the Organisation for Economic Co-operation and Development (OECD) as manufactured plastic particles smaller than 5 mm in diameter.
Ton	Metric ton i.e., 1000 kg (2204 lbs - pounds).
